# Environmental Toxicant Induced Epigenetic Transgenerational Inheritance of Prostate Pathology and Stromal-Epithelial Cell Epigenome and Transcriptome Alterations: Ancestral Origins of Prostate Disease

**DOI:** 10.1038/s41598-019-38741-1

**Published:** 2019-02-18

**Authors:** Rachel Klukovich, Eric Nilsson, Ingrid Sadler-Riggleman, Daniel Beck, Yeming Xie, Wei Yan, Michael K. Skinner

**Affiliations:** 1 Center for Reproductive Biology, School of Biological Sciences, Washington State University, Pullman, WA 99164-4236 USA; 2 Department of Physiology and Cell Biology, University of Nevada, Reno School of Medicine, Reno, NV 89557 USA

## Abstract

Prostate diseases include prostate cancer, which is the second most common male neoplasia, and benign prostatic hyperplasia (BPH), which affects approximately 50% of men. The incidence of prostate disease is increasing, and some of this increase may be attributable to ancestral exposure to environmental toxicants and epigenetic transgenerational inheritance mechanisms. The goal of the current study was to determine the effects that exposure of gestating female rats to vinclozolin has on the epigenetic transgenerational inheritance of prostate disease, and to characterize by what molecular epigenetic mechanisms this has occurred. Gestating female rats (F0 generation) were exposed to vinclozolin during E8-E14 of gestation. F1 generation offspring were bred to produce the F2 generation, which were bred to produce the transgenerational F3 generation. The transgenerational F3 generation vinclozolin lineage males at 12 months of age had an increased incidence of prostate histopathology and abnormalities compared to the control lineage. Ventral prostate epithelial and stromal cells were isolated from F3 generation 20-day old rats, prior to the onset of pathology, and used to obtain DNA and RNA for analysis. Results indicate that there were transgenerational changes in gene expression, noncoding RNA expression, and DNA methylation in both cell types. Our results suggest that ancestral exposure to vinclozolin at a critical period of gestation induces the epigenetic transgenerational inheritance of prostate stromal and epithelial cell changes in both the epigenome and transcriptome that ultimately lead to prostate disease susceptibility and may serve as a source of the increased incidence of prostate pathology observed in recent years.

## Introduction

Prostate disease is very common in older men in North America with 50% of men between the ages of 50 and 60 having evidence of pathologic benign prostatic hyperplasia (BPH)^1^. The incidence of prostate cancer has been increasing worldwide in the past decades with prostate cancer now being the second most common neoplasia in men^2–4^. While some of this increase can be attributed to an aging population other factors such as toxicant exposures and epigenetic transgenerational inheritance of disease susceptibility appear to be of importance. For the purposes of this article prostate pathology is referred to when abnormal histopathological changes are observed while the term disease is used when a specific prostate disease such as BPH or cancer is referenced.

Epigenetics is defined as “molecular factors and processes around the DNA that regulate genome activity independent of DNA sequence, and that are mitotically stable”^5^. Epigenetic factors include histone modifications, DNA methylation, non-coding RNAs (ncRNAs), RNA methylation and chromatin structure^6^. Epigenetic transgenerational inheritance is defined as the “germline transmission of epigenetic information and phenotypic change across generations in the absence of any continued direct environmental exposure or genetic manipulation”^5^. As an example, if an F0 generation pregnant mother is exposed to an environmental toxicant then the F1 generation fetus, and the developing germ cells in the fetus that will produce the F2 generation, are also directly exposed. Therefore, the subsequent F3 generation is the first unexposed transgenerational generation in which one can evaluate transgenerational inheritance. Epigenetic changes can be induced by environmental factors such as nutrition or toxicant exposure and are an important mechanism by which organisms change their gene expression in response to their environment. While transgenerational epigenetic changes must be inherited via germ cells (i.e. sperm or eggs), it is the epigenetic changes that these germ cells induce in the early embryo and embryonic stem cells that then promote an altered epigenome and transcriptome in all derived somatic cells of the individual. This can later in life lead to disease susceptibility in tissues and organs. Therefore, disease development in organs such as the prostate gland can be due to ancestral exposures and epigenetic inheritance^7^.

The prostate’s epithelium is responsible for contributing secretions to semen. Prostatic epithelial cells contain a large endoplasmic reticulum and golgi apparatus, as well as many secretory granules. There are multiple tubuloalveolar glands in a prostate that are lined by prostatic epithelium. These glands are separated from each other by adjacent prostatic stroma. The stroma of the prostate is considered to be the interstitial tissue and is made up of smooth muscle cells, blood vessels, fibroblasts, and nerves. These mesenchymal stromal cells are believed to work in unison with the epithelial cells to maintain prostate physiology and expel secretions to the semen^8^. In the current study the cell type isolated as prostatic stroma is primarily mesenchymal fibroblasts.

The prostate develops from the urogenital sinus (UGS) which branches to form the prostate in response to androgens, with the prostatic buds appearing in rats at embryonic E18-E19 and the majority of prostate branching occurring postnatally (reviewed in^9^). Rodent prostates have three prostatic lobes consisting of the anterior prostate, the dorsolateral prostate, and the ventral prostate, which has the most extensive branching. In the current studies prostatic cells are only isolated from the ventral prostate and the histopathology analysis is focused on the ventral prostate. After prostate growth is complete in the adult organism, the epithelium of the prostate has low levels of proliferation and cell death which maintain a constant prostate size in the presence of androgens. The epithelial-stromal ratio is also believed to be critical in determination of final prostate size (reviewed in^9^).

Smooth muscle cells in the prostate are known to have androgen receptors and are believed to regulate epithelial cells through androgen signaling^10^. High levels of testosterone have been shown to induce proliferation of prostate stromal cells^11^. A constant source of androgens is required to maintain a healthy prostate and is essential throughout development. In addition, estrogenic compounds can also have a wide variety of effects on the developing prostate (reviewed in^12^).

Although a variety of environmental toxicants following direct exposure have been associated with prostate pathology and disease^13,14^, few studies have investigated transgenerational effects in later generations not exposed *in utero* or neonatally. Initial studies of the ability of environmental toxicants to promote the epigenetic transgenerational inheritance of prostate pathology and disease showed that ancestral exposure to the agricultural fungicide vinclozolin increased rates of prostatic epithelial atrophy, cystic hyperplasia and prostatitis in the transgenerational F3 and F4 generations^15,16^. These effects were accompanied by transgenerational changes in mRNA expression in F3 generation ventral prostate epithelial cells, as determined by microarray analysis^16^. Associated epigenetic changes in these cells were not investigated at that time. In the current study, transgenerational changes to the epigenome of ventral prostate epithelial and stromal cells are characterized in F3 generation rats after ancestral vinclozolin exposure, compared to controls. Stromal-epithelial cell interactions are critical for normal prostate development and function, so abnormal interactions can lead to prostate diseases, including cancer^17–21^. The transgenerational epigenetic changes investigated in the current study involve changes to DNA methylation that have previously been associated with ancestral toxicant exposures in germ cells^22,23^ and somatic cells^24,25^. Additionally, epigenetic transgenerational changes in expression of mRNAs and non-coding RNAs (ncRNAs) are characterized for both prostatic epithelium and stroma.

Noncoding RNAs (ncRNAs) are any type of RNA whose functions are distinct from sequence complementarity and that are not involved in classic messenger RNA expression or translation. It is currently believed that long noncoding RNAs (lncRNAs) are responsible for maintaining epigenetic memory through regulation of DNA methylation, chromatin remodeling, histone modifications, or affecting transcription and translation by altering transcript stability (reviewed in^26^). A recent study has shown that the sperm of transgenerational males that were ancestrally exposed to DDT have differentially expressed lncRNAs^27^. Small noncoding RNAs have also been shown to have a role in epigenetic transgenerational inheritance. In *C*. *elegans* an increased sncRNA population was induced upon starvation and persisted until the F3 generation, resulting in longer lifespans^28^. There are many different kinds of small, noncoding RNAs that are found in the spermatozoa, and both the large (>200 nucleotide (nt)) and the small (<200 nt) noncoding RNAs have been found to have differential expression throughout spermatogenesis (reviewed in^29^).

Elucidation of epigenetic and gene expression changes that occur in the prostate after ancestral exposure to an environmental toxicant provides insight into the molecular etiology of the epigenetic transgenerational inheritance of prostate disease. These observations also improve our understanding of the risk factors (i.e. ancestral exposures) that must be considered when investigating the increasing incidence of prostate disease in the human population.

## Results

### Prostate Pathology Analysis

Pregnant F0 generation rats were treated with vinclozolin or control vehicle from days 8–14 of gestation, as described in Methods. Ventral prostatic tissue was harvested from the transgenerational F3 generation males at postnatal 18–21 days of age. Ventral prostate epithelial and stromal cells were isolated and analyzed so as to characterize DNA methylation, ncRNA expression and mRNA gene expression as described in Methods. Additional F3 generation vinclozolin lineage and control lineage rats were aged to one year and their ventral prostates subjected to histopathological evaluation to assess prostate pathology.

**Figure 1:**
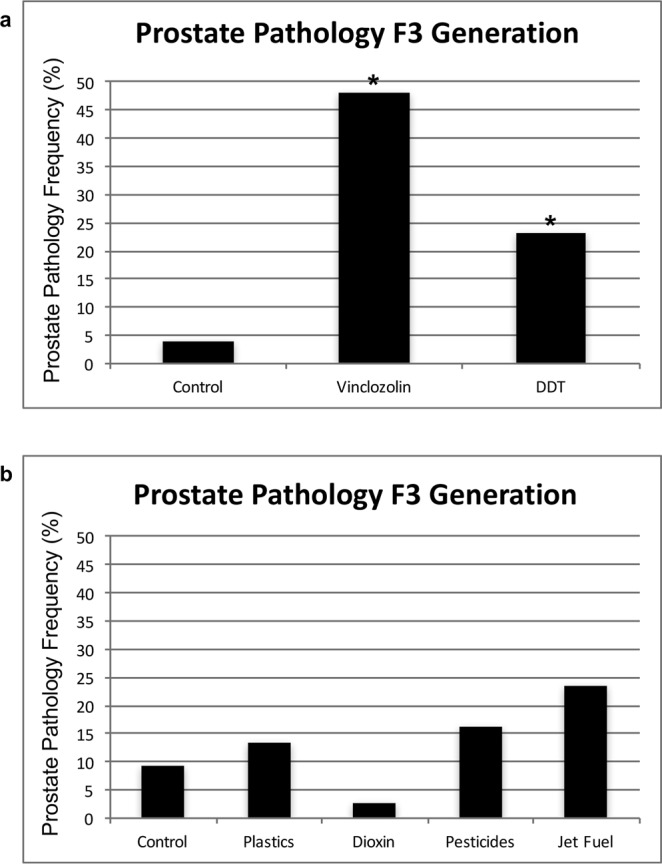
Prostate pathology frequency. (**a**) Transgenerational prostate disease in F3 generation control (n = 26), vinclozolin (n = 27) and DDT (n = 39) lineage males at 1 yr of age. The (*) indicates statistical significance of p < 0.05. (**b**) Transgenerational prostate disease frequency from previous studies in control, plastics^34^, dioxin^35^, pesticides^36^ and jet fuel^37^ lineage males at 1 yr of age (n = ~25 each). Histopathology analyses were performed using similar methods as with the current study. No statistical differences from control disease frequency were observed.

Prostate histopathology was defined as the presence of prostatic epithelial atrophy, epithelial hyperplasia, and/or as the presence of vacuoles in glands at rates two standard deviations above those found in controls (see Methods). There was a significant increase in prostate histopathology in transgenerational F3 generation vinclozolin lineage rats at one year of age (n = 27) compared to F3 generation controls (n = 26)^30^ (Fig. 1a). Representative prostate histopathologies are presented in Supplemental Figure S1. Several degrees of prostatic hyperplasia are presented, due to the previous observations that minor epithelial cell growth is considered normal^31^. A recent study also demonstrated a significant prostate pathology increase in F3 generation DDT lineage males (n = 39)^32^.

### DNA Methylation Analysis

**Figure 2:**
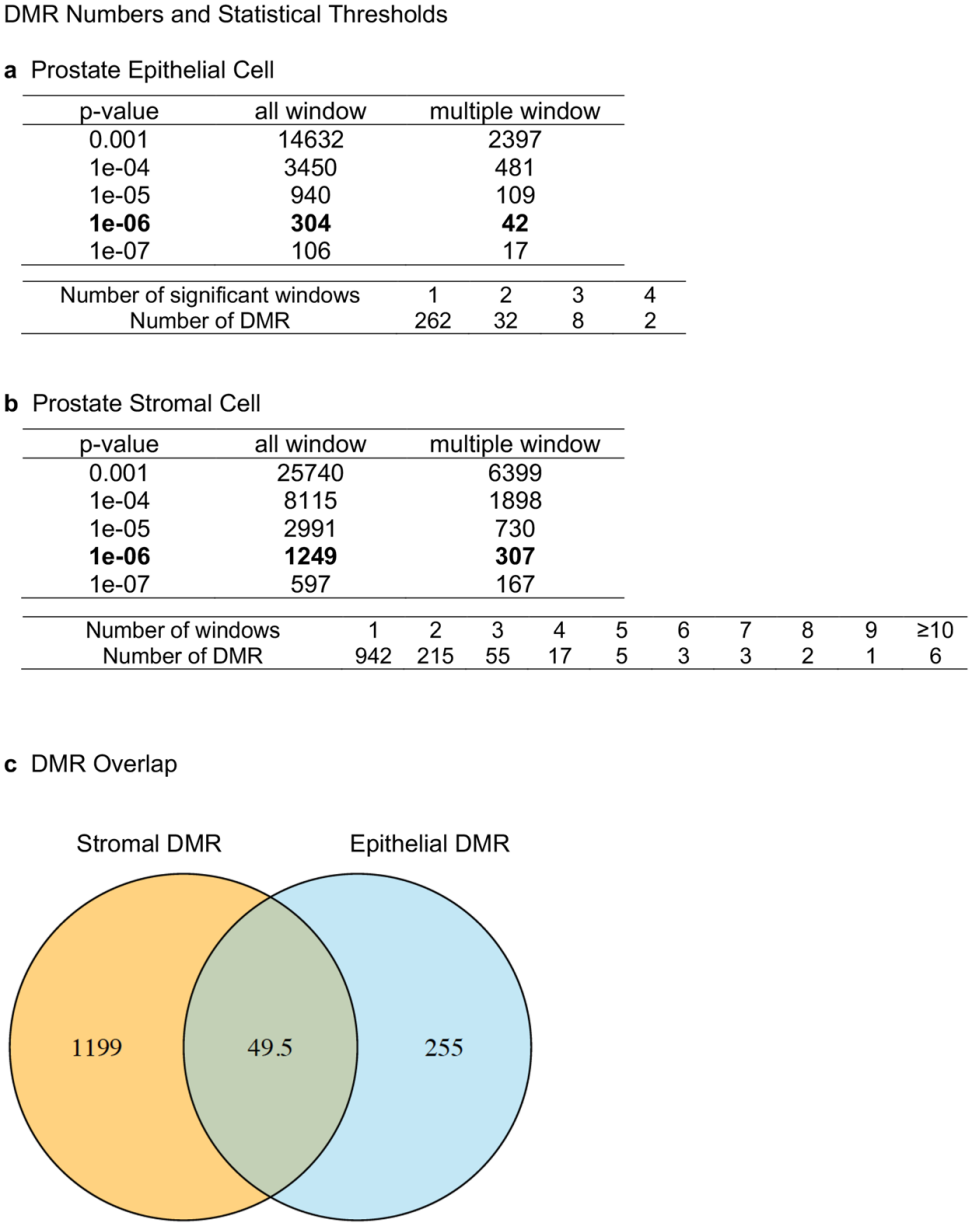
DMR identification. The number of DMRs found using different p-value cutoff thresholds is presented. The all window column shows all DMRs. The multiple window column shows the number of DMRs containing at least two significant neighboring windows. At the base of each table is presented the number of DMRs with each specific number of significant windows at p < 1e-06. (**a**) Prostate epithelial cell F3 generation DMRs p < 1e-06. (**b**) Prostate stromal cell F3 generation DMRs p < 1e-06. (**c**) Venn diagram showing the number of DMRs in common between prostate stroma and epithelium at p < 1e-6. Three pools of each prostate cell type with n = 6–11 different animals in each pool were used, as outlined in Methods.

**Figure 3:**
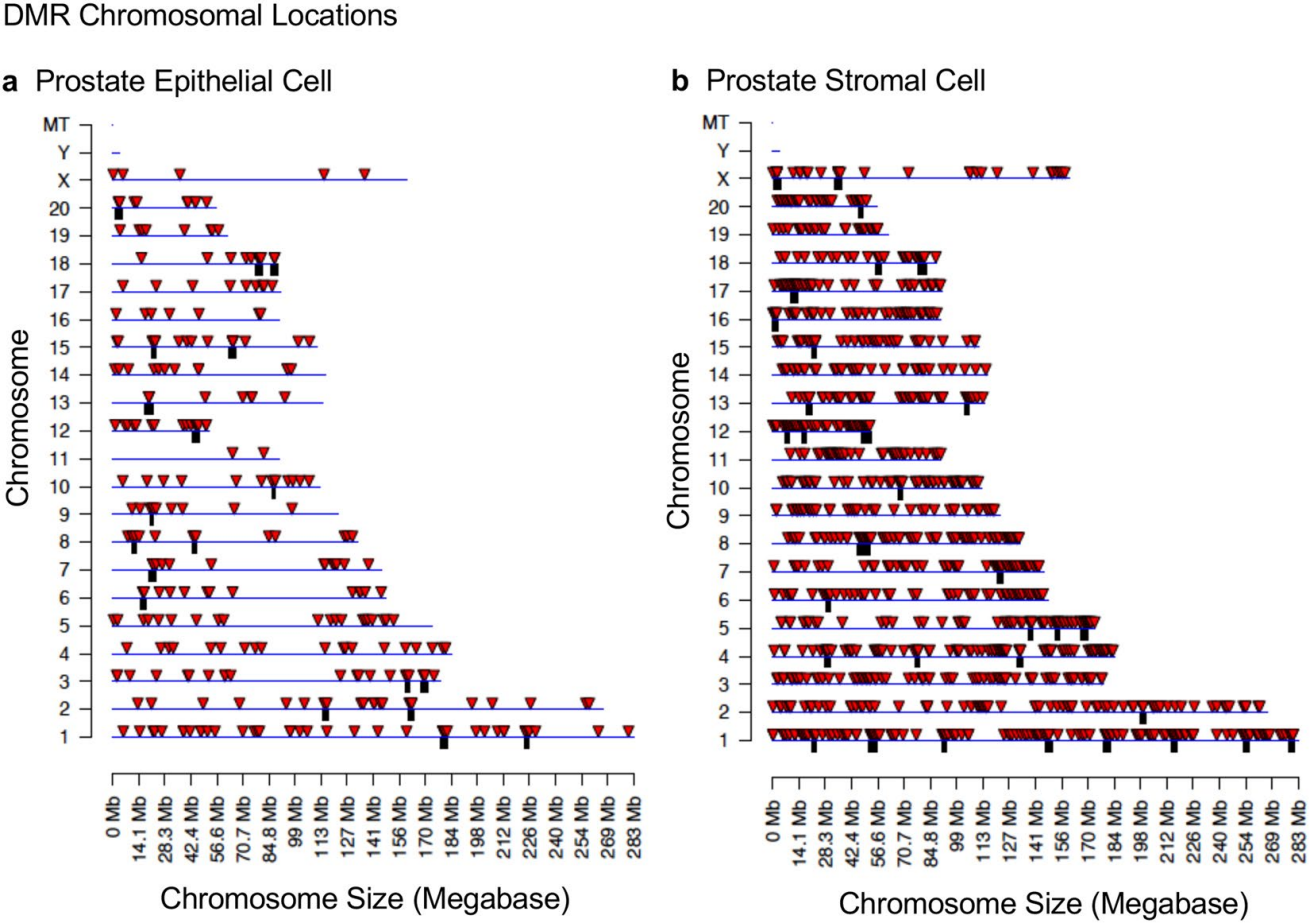
DMR chromosomal locations. The DMR locations on the individual chromosomes for all DMRs at a p-value threshold of p < 1e-06. (**a**) Prostate epithelial cells. (**b**) Prostate stromal cells. Red arrowheads indicate positions of DMR and black boxes indicate clusters of DMR.

Differences in sites of DNA methylation (*i*.*e*. Differential DNA Methylation Regions, DMRs) between the F3 generation control and vinclozolin lineage rats were characterized for both ventral prostatic epithelial cells and stromal cells using methylated DNA immunoprecipitation (MeDIP) followed by next generation sequencing for a MeDIP-Seq procedure and bioinformatics techniques as described in Methods. The DMRs are assessed in 100 bp windows of genome sequence and associated with altered read number following the sequencing. A number of p-value statistical thresholds were assessed and presented. The p-value selected allowed a more balanced comparison between groups and high statistical stringency. A false discovery rate (FDR) of 0.1 or less was associated with the majority of DMRs from these p-values selected. In prostate epithelial cells there were 304 DMRs at a p-value of p < 1 × 10^−6^, of which 42 DMRs comprised multiple neighboring genomic windows (Fig. 2a). A list of these DMRs is presented in Supplemental Table S1. In prostate stromal cells there were 1249 DMRs at a p-value of p < 1 × 10^−6^, of which 307 DMRs were comprised of multiple neighboring genomic windows (Fig. 2b). A list of these DMRs is presented in Supplemental Table S2. At p < 1 × 10^−6^ there were 50 DMRs in common between stromal and epithelial cells (Fig. 2c) and the list of these DMRs is presented in Supplemental Table S3. The DMR genomic location, statistics and ratio of vinclozolin/control fold change indicates an increase or decrease in DNA methylation, Supplemental Tables S1–S3. Approximately 50% of the DMRs had an increase in DNA methylation and the rest a decrease. The chromosomal locations of the DMRs were examined. The DMRs were present on all chromosomes except the Y chromosome and mitochondrial DNA (Fig. 3a,b). The red arrowheads identify the DMRs and black boxes clusters of DMRs.

**Figure 4:**
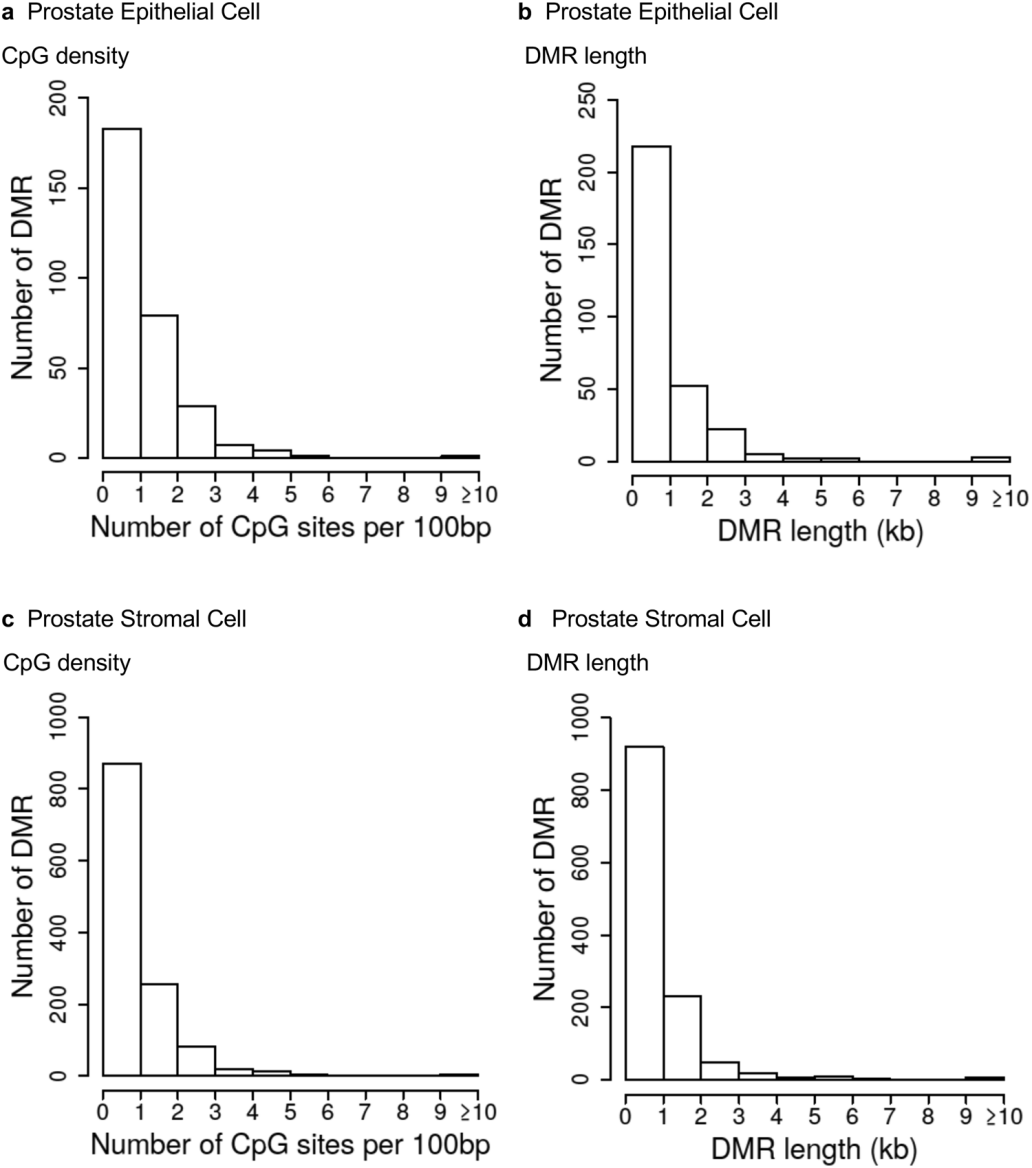
DMR genomic features. (**a**,**c**) The number of DMRs at different CpG densities for all DMRs at a p-value threshold of p < 1e-06. (**b**,**d**) The DMR lengths for all DMRs at a p-value threshold of p < 1e-06. (**a**,**b**) Prostate epithelial cells. (**c**,**d**) Prostate stromal cells.

Examination of the characteristics of the genomic sites where DMRs reside shows that most DMRs are present in areas having an average of 1 or 2 CpG sites per 100 base pairs (Fig. 4a,c). A CpG is a cytosine residue adjacent to a guanine residue on the DNA and the cytosine bases are methylated. This indicates that most of the DMRs identified occur in areas of low CpG density termed CpG deserts^33^. Most DMRs for both prostate epithelial cells and stroma cells were shown to be less than one kilobase (kb) in length (Fig. 4b,d). Characteristics of individual DMRs are presented in Supplemental Tables S1–S3.

### Non-Coding RNA Analysis

**Figure 5:**
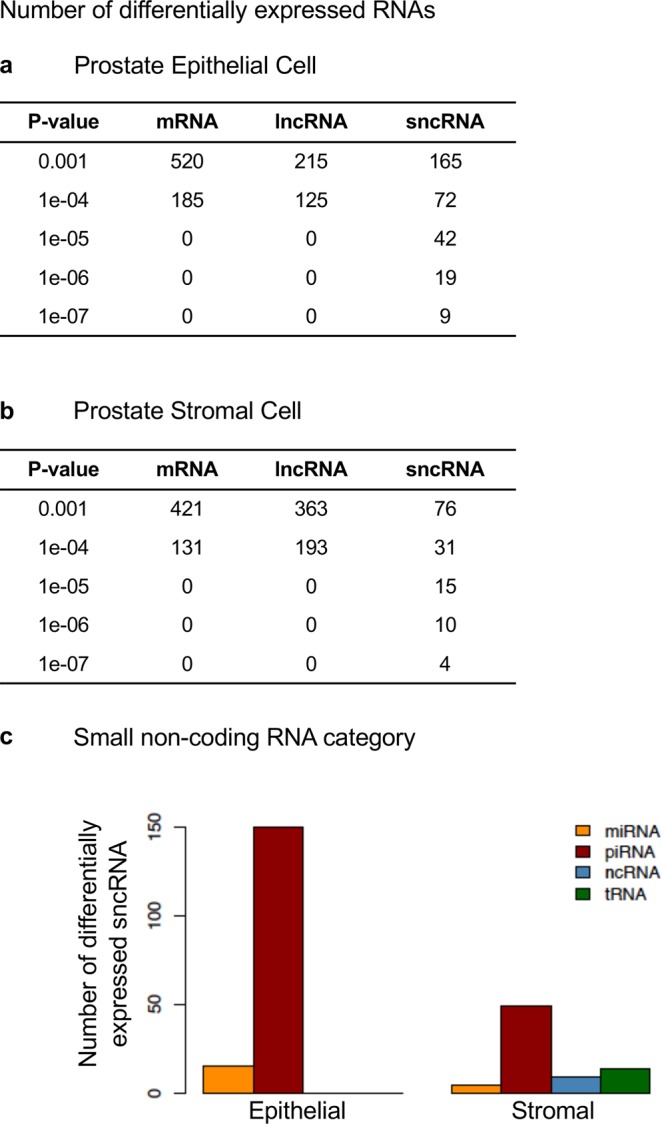
Differential expression of mRNA and noncoding RNAs between the control and vinclozolin lineages in prostate epithelial (**a**) and stromal (**b**) cells. (**c**) Categories of differentially expressed small, noncoding RNA at P < 0.001. Three pools of each prostate cell type with n = 6–11 different animals in each pool were used, as outlined in Methods.

The differentially expressed mRNA, long ncRNA (lncRNA) and small ncRNAs (sncRNA) were determined using RNA-seq data, comparing vinclozolin lineage ventral prostate stromal and epithelial cells to control cells. The numbers of differentially expressed RNAs of different classes at different p-value statistical thresholds are presented for both prostate epithelium and stroma (Fig. 5a,b, respectively). A significance level of p < 0.001 was chosen for subsequent analysis. Specific locations of differentially expressed ncRNAs for the prostate epithelium and stroma are presented for sncRNA in Supplementary Tables S4–S6; for lncRNA in Supplementary Tables S7–S9; and for mRNA in Supplementary Tables S10–S12, respectively. In both epithelial and stromal cells mRNA had the highest number of differentially expressed transcripts (520 vs 421, respectively), followed by those categorized as lncRNA. There were about twice as many differentially expressed sncRNAs in the epithelial prostate cells as there were in stromal prostate cells (165 vs 76, respectively). Differentially expressed sncRNAs were subsequently broken down into categories by type with both epithelial and stromal prostate cells having piRNA as the most numerous category of sncRNAs (Fig. 5c). Interestingly, sncRNAs other than those of the miRNA and piRNA classes were not differentially expressed in the epithelial cells, but small tRNA fragments were differentially expressed in the stromal cells. Therefore, the different classes of sncRNAs are apparently affected differently in the same tissue in different cell types, indicating that ancestral exposures can have different effects on different cell types.

**Figure 6:**
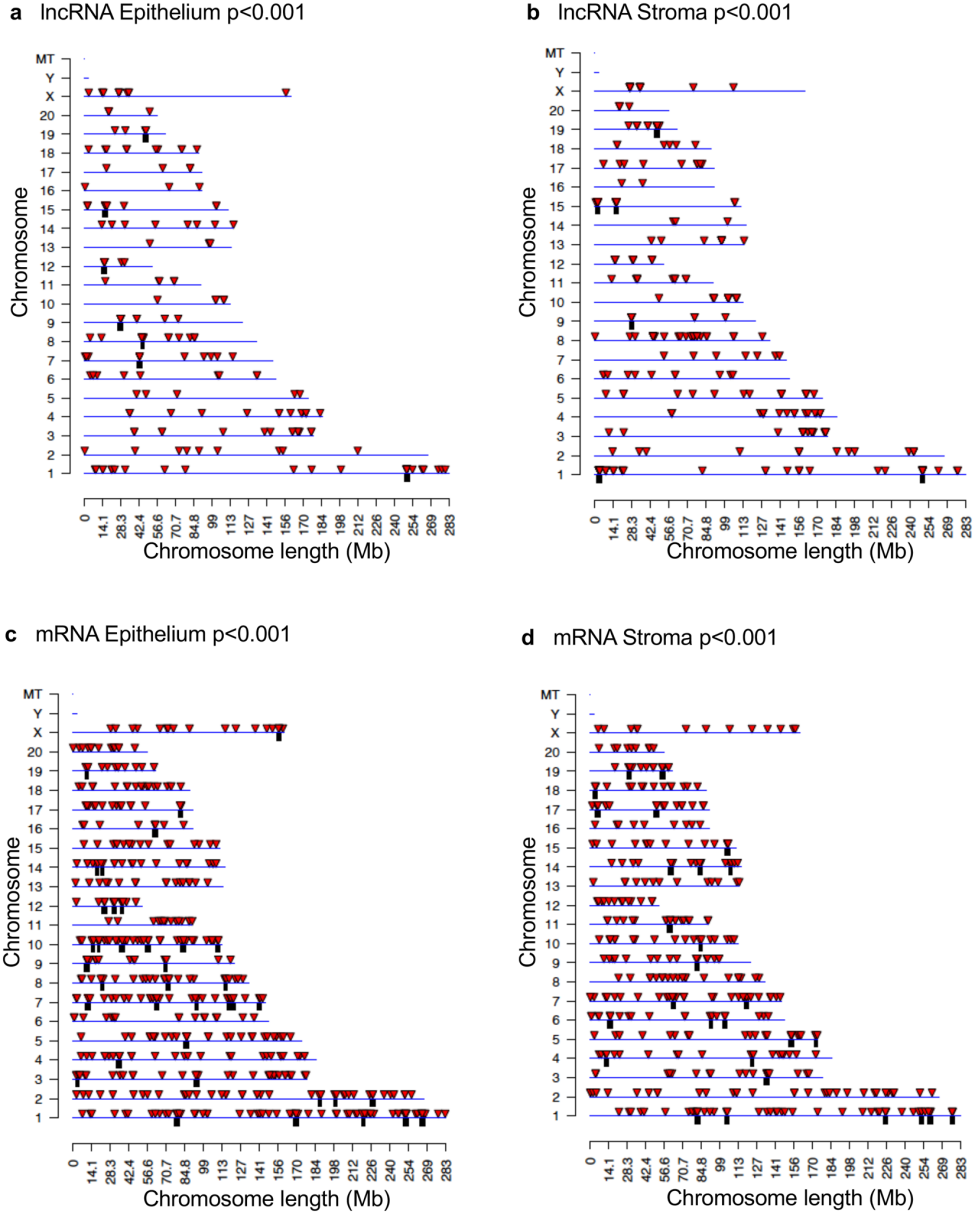
Chromosomal locations of differentially expressed large RNAs. Long, noncoding RNAs from the epithelium (**a**) and stroma (**b**). mRNAs from the epithelium (**c**) and stroma (**d**). Red arrows indicate individual large RNAs, while black boxes indicate clusters. P < 0.001.

**Figure 7:**
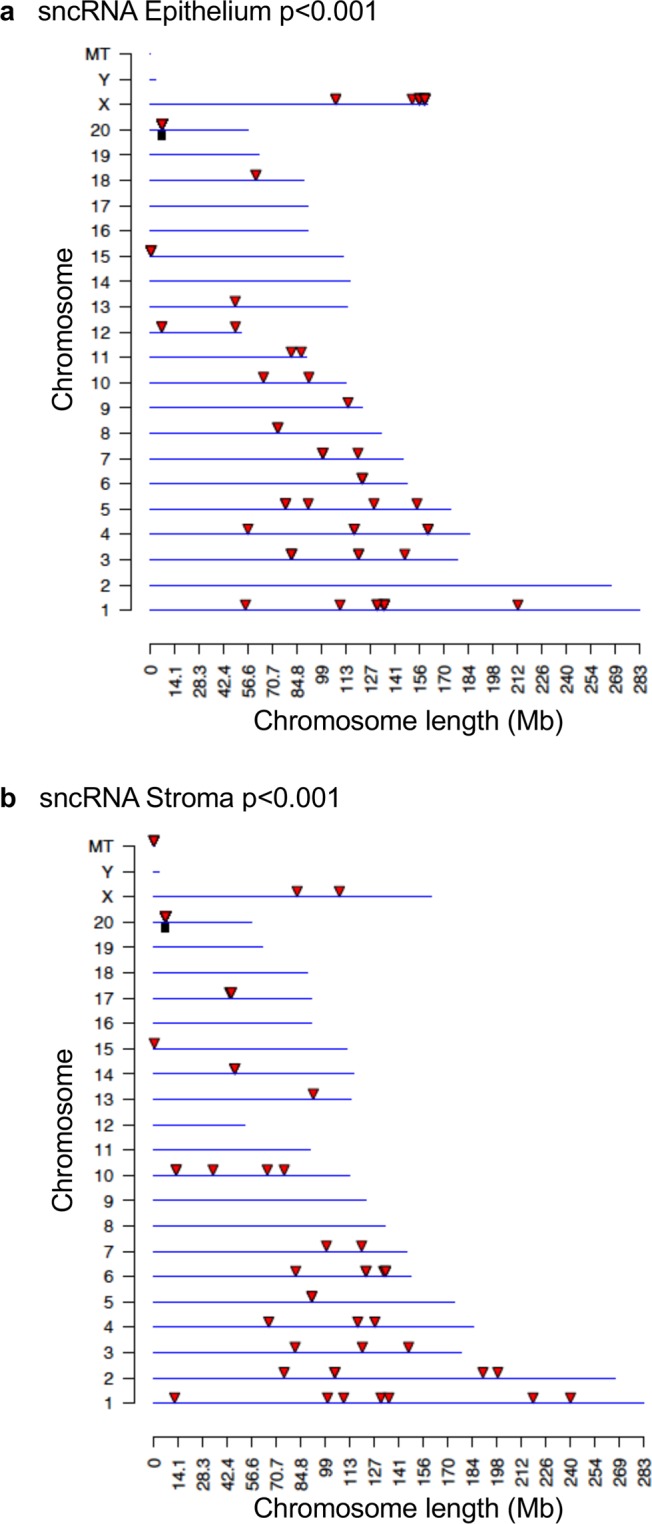
Chromosomal locations for differentially expressed small, noncoding RNAs from the epithelium (**a**) and stroma (**b**). Red arrows indicate individual sncRNAs, while black boxes indicate clusters. P < 0.001. There are 9 differentially expressed sncRNA with unknown locations from the epithelium and 4 from the stroma (Supplemental Table S4).

Chromosomal locations of differentially expressed mRNA and ncRNA were analyzed. The differentially expressed lncRNA from both the epithelial (Fig. 6a) and stromal (Fig. 6b) cell types were present on all chromosomes except for the Y chromosome and mitochondrial DNA. This was also the case for the differentially expressed mRNAs from both epithelium and stroma (Fig. 6c,d, respectively). Chromosomal locations of differentially expressed sncRNAs are presented in Fig. 7 and Supplemental Tables S4–S6. Only the stromal cell line had a differentially expressed sncRNA on the mitochondrial (MT) chromosome.

**Figure 8:**
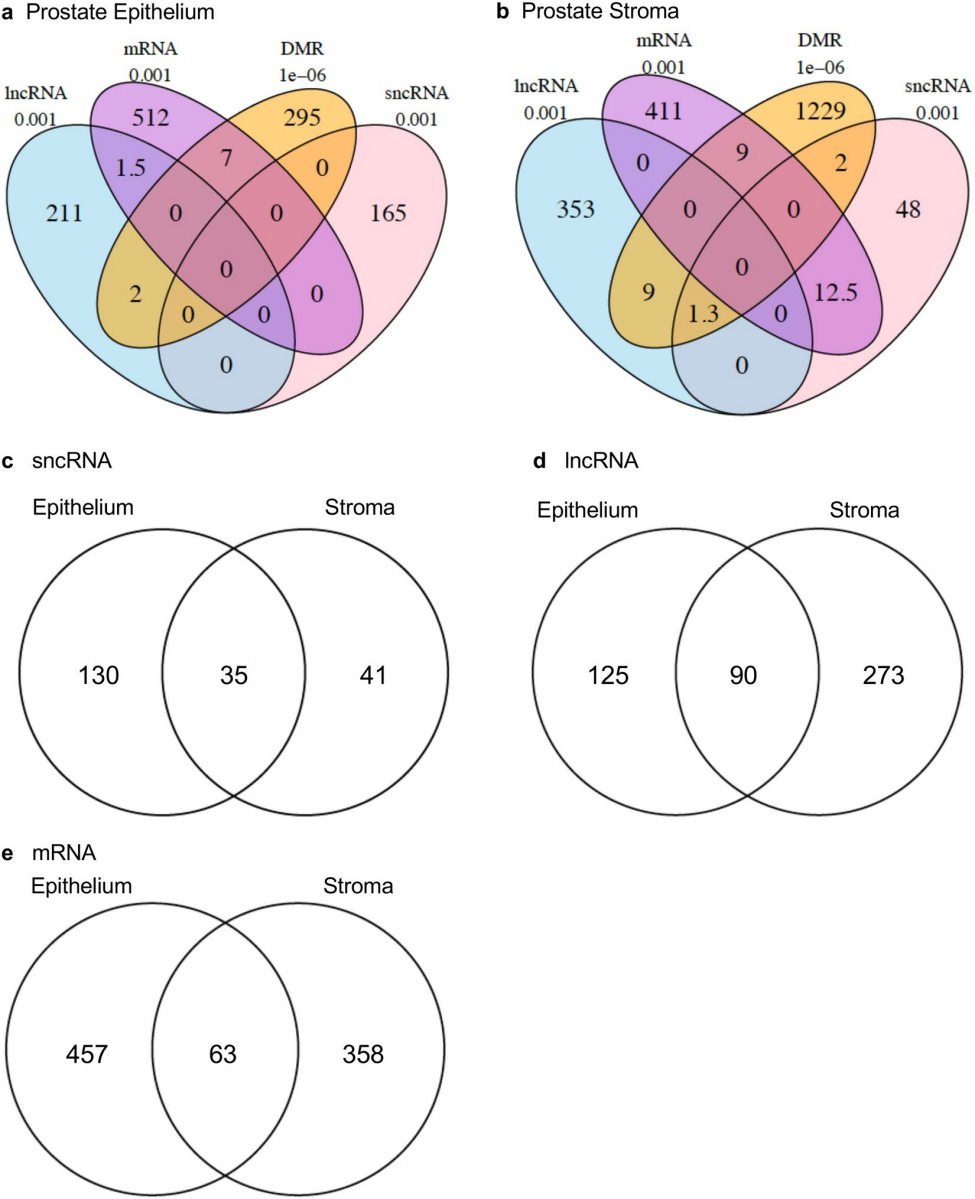
Overlaps of the DMRs (p < 1e-6) with the differentially expressed noncoding RNAs (p < 0.001) in the prostate epithelium (**a**) and the prostate stroma (**b**). Overlaps of differentially expressed RNAs between epithelium and stroma for sncRNA (p < 0.001) (**c**), lncRNA (p < 0.001) (**d**) and mRNA (p < 0.001) (**e**).

All differentially expressed transcripts and epigenetic modifications within both the epithelium and the stroma were compared to assess overlaps (i.e. lncRNA, mRNA, sncRNA, and DMRs). In the ventral prostate epithelium (Fig. 8a), DMRs overlapped individually with 2 lncRNA transcripts and 7 mRNA transcripts. Very little overlap (1–2 transcripts) was observed between the lncRNA and the mRNA, and no overlap was observed with any of the sncRNAs. In contrast, in the prostate stroma there was a larger overlap of 12.5 transcripts between the mRNA and the sncRNA, and no overlap between the lncRNA and the mRNA (Fig. 8b). The stromal cell DMRs overlapped with 9–10 transcripts each of both the mRNA and the lncRNA, but none were in common to all three.

Noncoding RNA transcripts were compared between prostate epithelial and stromal cells (Fig. 8). Of the 165 differentially expressed sncRNA transcripts present in epithelium and the 76 differentially expressed sncRNA transcripts present in stroma there were 35 in common (Fig. 8c and Supplemental Table S7). Similarly, of the 215 differentially expressed lncRNA transcripts present in epithelium and the 363 differentially expressed lncRNA transcripts present in stroma there were 90 in common (Fig. 8d and Supplemental Table S8).

### Gene Association Analysis

**Figure 9:**
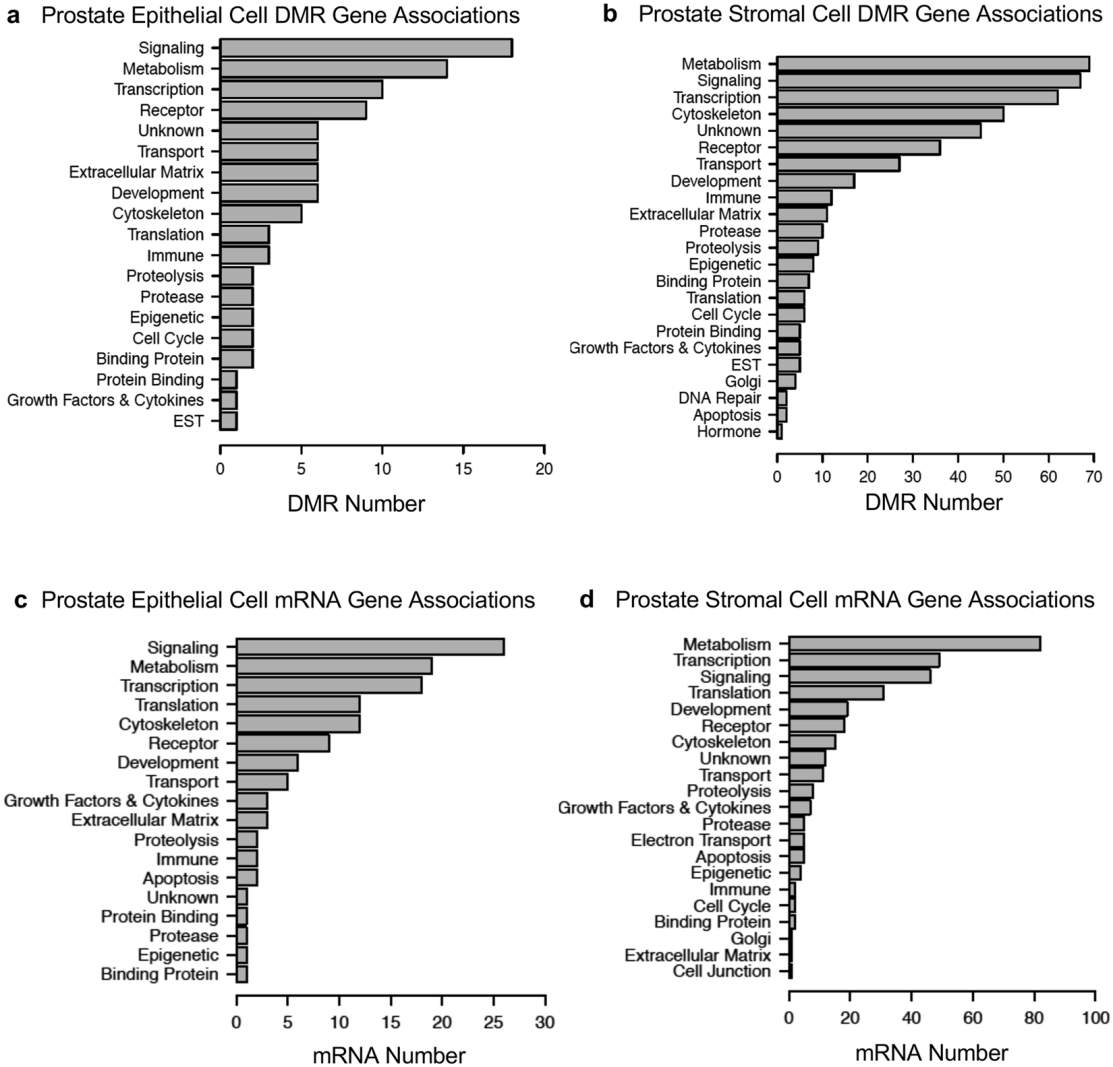
Gene functional categories for DMR associated gene categories. (**a**) Prostate epithelial cell and (**b**) Prostate stromal cell. Genes within 10 kb proximity to DMR were categorized as to function and the number of DMR associated genes in each category presented. DMRs are at a p-value of ≤ 1e-6. Differentially expressed mRNA gene categories for (**c**) Prostate epithelial cell and (**d**) Prostate stromal cell. Differentially expressed mRNA genes (p < 0.001) were categorized and the number of genes in each category presented.

Some DMRs occurred in the vicinity (within 10 kb) of known genes, Supplemental Tables S1–S3. This 10 kb window allows the flanking regions of the gene, such as the promoter, to be considered. These DMR associated genes were categorized and evaluated for potential function. For both prostate epithelial cells and stroma cells the DMR associated genes were most often related to signaling, metabolism, transcription and receptor functions (Fig. 9a,b).

Differentially expressed mRNA transcripts in prostate epithelial and stromal cells were categorized and evaluated for potential function. In epithelium the highest number of differentially expressed genes had functions related to metabolism, transcription, translation, signaling and development (Fig. 9c). Similarly, in stromal cells the highest number of differentially expressed genes had functions related to signaling, metabolism, transcription, translation, cytoskeleton and development (Fig. 9d).

**Figure 10:**
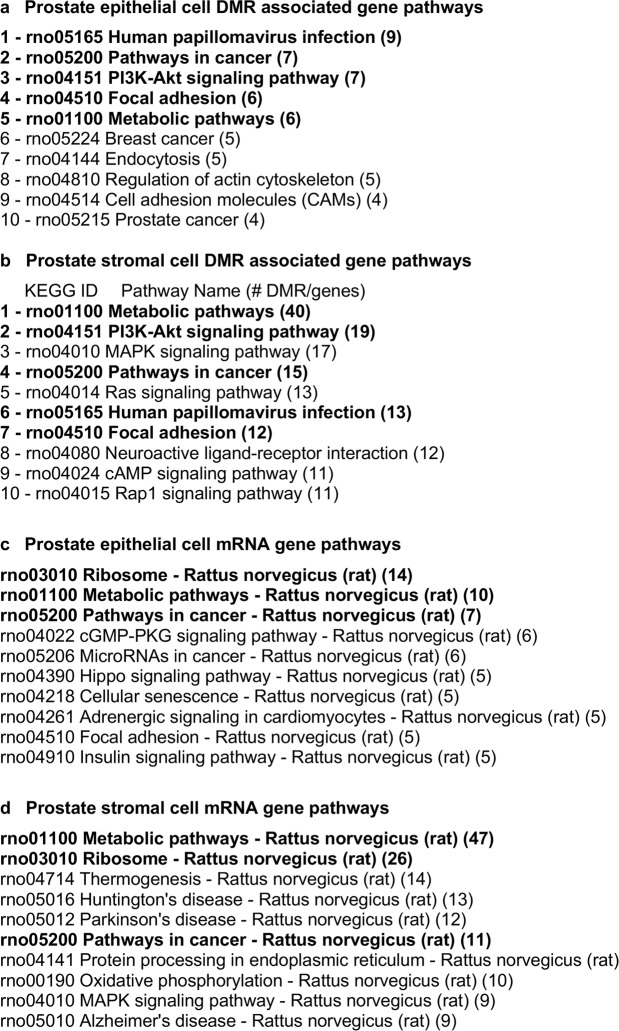
Gene pathways for DMR associated gene pathways. (**a**) Prostate epithelial cell. (**b**) Prostate stromal cell. DMR associated genes were surveyed for their presence in known physiological pathways (KEGG pathways). The number of DMR associated genes present in each pathway is indicated. Bold indicates common pathways between the cell types. Differentially expressed mRNA gene pathways in (**c**) Prostate epithelial cell and (**d**) Prostate stromal cell. Differentially expressed mRNA genes were surveyed for their presence in known physiological pathways (KEGG pathways). The number of mRNA genes present in each pathway is indicated. Bold indicates common pathways between the cell types.

The lists of differentially expressed DMRs and mRNAs were also compared to well-characterized physiological pathways in the KEGG database (http://www.kegg.jp/kegg/kegg2.html). Those pathways having the most DMR associated genes and differentially expressed mRNAs are presented in Fig. 10. Among the KEGG pathways containing DMR associated genes from epithelial cells and those from stromal cells there were five pathways (bolded) in common (Fig. 10a,b). Similarly, for differentially expressed mRNAs three of the pathways are in common between the epithelial cells and stromal cells (Fig. 10c,d). The ‘Pathways in Cancer’ KEGG pathway is presented in Supplemental Figure S2 and shows the DMR associated genes and differentially expressed mRNAs from both epithelium and stroma within this pathway, featuring several different signaling cascades. The extracellular matrix (ECM) and growth factor – cytokine signaling were the most predominant components of the pathway affected, Supplemental Figure S2.

Differentially expressed mRNA transcripts were compared between prostate epithelial and stromal cells (Fig. 8e). Of the 520 differentially expressed mRNA transcripts present in epithelium and the 421 differentially expressed mRNA transcripts present in stroma there were 63 in common (Supplemental Table S8).

A final analysis identified genes previously associated with prostate disease (cancer and BPH) in the literature and compared them to the results of the current study. A list of 159 genes that have previously been shown to be associated with prostate disease are presented in Supplemental Table S13. Several genes previously associated with prostate disease were also identified as affected in the current study: Fli1 (stromal DMR), Egf (epithelial DMR), Dgk2 (stromal differentially expressed mRNA), Snai2 and Cxcl1 (epithelial differentially expressed mRNA). Therefore, several genes previously shown to be associated with prostate disease were present in the DMR or differentially expressed mRNA lists.

## Discussion

The results of these studies indicate that ancestral exposure to the toxicant vinclozolin induces an epigenetic transgenerational increase in susceptibility to prostate pathology in F3 generation rats. These results are in agreement with previous studies which found a transgenerational increase in rates of prostatic epithelial atrophy, cystic hyperplasia, and prostatitis in the transgenerational F3 and F4 generations^15^ after exposure of F0 generation pregnant rats to vinclozolin. These effects were accompanied by transgenerational changes in mRNA expression in F3 generation ventral prostate epithelial cells^16^. Prostate diseases, which include benign prostatic hyperplasia and prostate cancer, are common in aging men^1–4^. Observations suggest that ancestral exposures to toxicants and epigenetic transgenerational inheritance may contribute to the development of prostate disease in men today.

Interestingly, a number of previous transgenerational studies have shown no ventral prostate histopathology or disease detected following plastic derived compound exposures (bisphenol A (BPA) and phthalates (DBT & DEHP))^34^, dioxin (TCCD)^35^, pesticide permethrin and insect repellent N,N-Diethyl-meta-toluamide (DEET)^36^, jet fuel hydrocarbons^37^, or methoxychlor^38^ exposures, Fig. 1b. Therefore, observations suggest ancestral exposure specificity in the ability to induce the transgenerational inheritance of prostate disease. There was also no increase in prostate histopathology in the directly exposed F1 or F2 generation vinclozolin lineage rats compared to controls^30,39^. This indicates that there was a transgenerational increase in susceptibility to prostate pathology and disease in rats ancestrally exposed to vinclozolin. A variety of compounds, for example phthalates, can promote prostate disease in the F1 generation after direct *in utero* or developmental exposure^40,41^. However, these are not examples of transgenerational inheritance of pathology to an unexposed generation^42^.

Changes in DNA methylation were observed in F3 generation vinclozolin lineage ventral prostate epithelial and stromal cells compared to the control lineage. The sites of these DMRs were in genomic regions of relatively low CpG density “CpG deserts”^33^. This finding is consistent with previous work in which transgenerational DMRs in sperm were most often found in regions of low CpG density after ancestral toxicant exposure^34–38^. Changes in DNA methylation can affect genome activity and gene expression in concert with other epigenetic factors. DMRs were found in both epithelial and stromal cells that were associated (within 10 kb to include the promoter) to genes, raising the possibility that these genes might be epigenetically regulated. An investigation of the putative functions of DMR associated genes revealed signaling, transcription, development and receptor genes to be predominant. These classes of genes are important for the stromal-epithelial interactions that are necessary for normal prostate function and dysregulation may promote prostate disease. Similarly, several DMR associated genes were present in a ‘Pathways in Cancer’ KEGG pathway (Supplementary Fig. S1). Observations suggest that their abnormal expression might promote prostate cancer susceptibility. There were only a few DMRs that overlapped with differentially expressed mRNA transcripts including 7 in prostate epithelium and 9 in stroma (Fig. 8). Considering that the differentially expressed mRNAs were evaluated in epithelial and stromal cells collected from young animals with healthy prostates, further epigenetic changes in aging animals may be required to increase disease susceptibility.

Examination of the noncoding RNAs showed that the two prostate cell types, epithelium and stroma, had very different classes of differentially expressed ncRNAs. While the prostate epithelium had more differentially expressed sncRNAs compared to the stroma (165 vs 76), none of the differentially expressed epithelial sncRNAs overlapped with the other alterations. This is in contrast to the stromal sncRNAs, where 12 sncRNAs were shown to overlap with stromal mRNAs. This is almost 16% of the total differentially expressed sncRNAs, one of which was also found to be located on the mitochondrial DNA. As sncRNAs are known to affect gene expression, it is likely that one mechanism by which epigenetic transgenerational inheritance affects stromal cells in the prostate is through sncRNAs. Unlike the stroma, the prostate epithelium did not have any differentially expressed sncRNA overlap with either lncRNA or mRNA. In contrast, epithelial DMRs were found to overlap with both differentially expressed mRNAs and lncRNAs. This suggests that the mechanism by which epigenetic transgenerational inheritance affects prostate epithelium involves control of gene expression by DNA methylation and lncRNAs. In the future, it will be necessary to determine the exact gene targets of these epigenetic modifications to determine further mechanisms by which prostate diseases occur due to ancestral exposure to toxicants.

Differential expression was compared between prostate epithelial and stromal cells for sncRNA, lncRNA and mRNA (Fig. 8). There were relatively high numbers of differential expression in common between epithelium and stroma for each RNA class, but less overlaps between RNA classes or DMR within a single cell type. The reasons for this are unclear, but it is possible that this is an artifact of the limited degree of annotation and mapping of sncRNAs and lncRNAs in the rat genome. Interestingly, there was also a relatively high degree of overlap of differentially expressed mRNAs between epithelium and stroma (Fig. 8e). Therefore, these overlaps may be a manifestation of ‘epigenetic control regions’ regulating groups of genes, as proposed by Skinner *et al*.^43^. Epigenetic control regions are portions of the genome that are up regulated or down regulated as a block by epigenetic factors. Those genes present within the region, if expressed in a particular cell type, may be similarly regulated. The relatively high overlap of differentially expressed mRNA transcripts between epithelium and stroma may be a reflection of this phenomenon.

It is interesting to note that epimutations are present in prostatic epithelium and stroma even at 18–21 days of age, which is long before any visible signs of prostate histopathology or disease are detectable. This indicates that the underlying factors that can contribute to an adult-onset disease like prostate disease can be present early in life. Further changes that occur in aging animals could then activate these epimutations and lead to gene dysregulation that promotes prostate disease. The differentially expressed mRNAs present in young prostatic tissue and the DMR associated genes that may alter expression as animals age are parts of known pathways affecting cell adhesion, gene translation and signaling pathways involved with cancer (Fig. 10). These genes included receptors, growth factors and extracellular matrix components that could be important to the cell-cell communication that is necessary for normal prostate function^18,20,21^ (Fig. 9, Supplemental Tables S10 and S11). Several regulated growth factor and receptor mRNAs (Kitlg, Lif, Ntrk3, Fgf9, Cxcl1 and Mif) have been implicated in prostate cancer^44–49^ and normal prostate function^50^. Additional genes identified as affected transgenerationally in this study have also been previously associated with prostate disease including Fli1, Egf, Dgk2 and Snai2 (Supplemental Table S13). It is interesting to note that both rats and men have a delayed prostate disease onset occurring in later life (in rats over 1 year of age and over 50 years old in men), with the prevalence of prostatic lesions in both groups being very similar at approximately 50%^1–4^.

In summary, these studies show that exposure to the environmental toxicant vinclozolin can promote the epigenetic transgenerational inheritance of susceptibility to prostate disease. Prostate epithelial and stromal cells from young vinclozolin lineage animals had epigenetic changes in DNA methylation and ncRNA expression, as well as in mRNA gene expression. These changes likely contribute to the dysregulation of the prostate gland that occurs in later life. Future studies need to investigate if similar mechanisms are at work in human males who have adult-onset BPH or prostate cancer. Ancestral exposures and epigenetic transgenerational inheritance need to be considered in the molecular etiology of prostate disease.

## Methods

### Animal studies and breeding

Female and male rats of an outbred strain Hsd:Sprague Dawley®™SD®™ (Harlan) at about 70 to 100 days of age were fed ad lib with a standard rat diet and ad lib tap water for drinking. To obtain time-pregnant females, the female rats in proestrus were pair-mated with male rats as previously described^30^. The sperm-positive (day 0) rats were monitored for diestrus and changes in body weight. If pregnant, then on days 8 through 14 of gestation^51^, the females were administered daily intraperitoneal injections of vinclozolin (100 mg/kg BW/day, Chem Services, Westchester PA, USA) or dimethyl sulfoxide (vehicle) as previously described^22^. This pharmacological level dose of vinclozolin was used because it is known to result in transgenerational epigenetic effects, and so results can be compared with previous studies^15,16^. Treatment groups were designated ‘vinclozolin’ and ‘control’ lineages. The gestating female rats treated were considered to be the F0 generation. The offspring of the F0 generation rats were the F1 generation. If litters of pups were larger than 11 pups, then litters were culled down to 10 pups in the first week after birth. Litter sizes ranged from three to 11 pups, and were not different between F3 generation treatment groups (data not shown). Non-littermate females and males aged 70–90 days from F1 generation control or vinclozolin lineages were bred to obtain F2 generation offspring. The F2 generation rats were bred to obtain F3 generation offspring without using sibling or cousin breedings to avoid inbreeding. Only the pregnant F0 generation rats were treated directly with vinclozolin. The control and vinclozolin lineages were housed in the same rooms with lighting, food and water as previously described^5,15,22^. All experimental protocols for the procedures with rats were pre-approved by the Washington State University Animal Care and Use Committee (IACUC approval # 6252) and all experiments were performed in accordance with relevant guidelines and regulations.

### Tissue harvest and histology processing

As previously described^30^, rats at 12 months of age were euthanized by CO_2_ inhalation and cervical dislocation for tissue harvest. Ventral prostates were removed and fixed in Bouin’s solution (Sigma) followed by 70% ethanol, then processed for paraffin embedding by standard procedures for histopathological examination. Tissue sections (5 µm) were cut and were stained with H & E stain and examined for histopathologies.

### Histopathology examination and disease classification

Prostate histopathology criteria included the presence of vacuoles in the glandular epithelium indicating epithelial cell loss or death, an atrophic epithelial layer affecting at least one third of a microscopic gland, and hyperplasia of prostatic epithelium as previously described^16,30,52^ (Supplemental Figure S1). Minor hyperplasia of an extra cell layer is difficult to distinguish from epithelial stratification, and is a normal prostate phenomenon^31^ and was not counted as a histopathology. Significant hyperplasia and localized epithelial cell growth was considered histopathologies (Supplemental Fig. S1e,f). For each rat the number of histopathological abnormalities in each of the above categories was counted in one complete section of ventral prostate cut on a horizontal plane below the bladder and including left and right portions of the ventral prostate. A cut-off was established to declare a tissue ‘diseased’ based on the mean number of histopathological abnormalities in each category plus two standard deviations from the mean of control tissues by each of the three individual observers blinded to the treatment groups. This number was used to classify rats into those with and without prostate pathology in each lineage. A rat tissue section was finally declared ‘diseased’ only when at least two of the three observers marked the same tissue section ‘diseased’. Results were expressed as the proportion of affected animals and were analyzed using Fisher’s exact test. Prostates from twenty-six control lineage rats from ten different litters, twenty-seven vinclozolin lineage rats from eleven different litters, and thirty-nine DDT lineage rats from ten different litters were evaluated.

### Prostatic epithelial and stromal cell collection

The ventral prostate epithelial and stromal cells were isolated as previously described^53^. For the control-lineage animals prostate tissue was collected in 3 groups with group 1 comprising 9 rats, group 2 comprising 7 rats and group 3 6 rats. For the vinclozolin -lineage animals prostate tissue was collected in 3 groups as well, comprising 9, 10 and 11 rats. In each group the tissues from the individual rats were combined and processed for isolation of epithelial and stromal cells according to the protocol. Briefly, ventral prostates were removed from 19–21 day old rats and cleaned of fat, then digested in 50 ml Hank’s Buffered Salt Solution (HBSS) with 0.5 mg/ml collagenase type II (Sigma C1764) and 66 μg/ml DNAse (Sigma DN25) with agitation at 37° for up to 4 hours depending on digestion progress. After gravity settling for 10 min. the supernatant containing the stromal cells was removed. The supernatant was centrifuged at 30xg for 4 min. to pellet contaminating epithelial cells, and then the supernatant centrifuged again at 190xg for 6 min to pellet the stromal cells. This wash is repeated 1–2 more times. To clean the epithelial cells from the original gravity settled pellet, the pellet is resuspended in HBSS, centrifuged at 30xg for 4 min., and the supernatant discarded. This wash is repeated 1–2 more times. For each group the final pellets for epithelial and stromal cells were divided for DNA and RNA isolation and frozen at −80 degrees for further processing. This resulted in 3 epithelial and 3 stromal samples for DNA isolation, as well as 3 epithelial and 3 stromal samples for RNA isolation, for both vinclozolin and control treatment groups.

### DNA Isolation

Genomic DNA was prepared as previously described^54^. The cell pellet was resuspended in 820 μL DNA extraction buffer (0.05 M Tris HCl, pH 8; 0.01 M EDTA; 0.5% SDS) and then 80 μl proteinase K (20 mg/ml) added. The sample was incubated at 55 °C for 2–3 hours under constant rotation. Then 300 μl of protein precipitation solution (Promega A795A) was added, the sample mixed thoroughly and incubated for 15 min on ice. The sample was centrifuged at 17,000xg for 30 minutes at 4 °C. One ml of the supernatant was transferred to a 2 ml tube and 2 μl of Glycoblue cryoprecipitant (Thermo Fisher Scientific AM9516) and 1 ml of cold 100% isopropanol were added. The sample was mixed well by inverting the tube several times then incubated at −20 °C for at least one hour. After precipitation, the sample was centrifuged at 17,000 × g for 20 min at 4 °C. The supernatant was discarded without disturbing the (blue) pellet. The pellet was washed with 70% cold ethanol and incubated at −20 °C for 20 minutes. Samples were centrifuged for 10 min at 4 °C at 17,000 × g and the supernatant discarded. Pellet was air-dried at RT (about 5 minutes), then resuspended in 100 μl of nuclease free water. Concentration of the resulting DNA was determined in the NanoDrop.

### Methylated DNA Immunoprecipitation (MeDIP)

Genomic DNA was used for MeDIP as previously described^30,54^. Briefly, genomic DNA extracted from the tissue pools (as described above) was diluted if necessary to the appropriate volume of 130 μl and then fragmented in the Covaris M220 using the manufacturer’s 300 bp program. Sizing was confirmed on a 1.5% agarose gel. DNA was diluted with TE buffer to 400 μl, heat-denatured for 10 min at 95 °C, then immediately cooled on ice for 10 min. 100 μl of 5X IP buffer and 5 μg of antibody (monoclonal mouse anti 5-methyl cytidine; Diagenode #C15200006) were added, and the DNA-antibody mixture was incubated overnight on a paddle rotator at 4 °C.

The following day 50 μl of pre-washed anti-mouse IgG magnetic beads (Dynabeads M-280 Sheep anti-Mouse IgG; Life Technologies 11201D) were added to the DNA-antibody mixture, incubated for 2 h on a rotator at 4 °C, and then the DNA-antibody-bead mixture was washed with 1xIP buffer 3 times using a magnetic rack. The washed sample was resuspended in 250 μl digestion buffer (5 mM Tris PH8, 10.mM EDT4, 0.5% SDS) with 3.5 μl Proteinase K (20 mg/ml)) and incubated for 2–3 hours on a rotator at 55°. After this incubation the DNA was cleaned up with buffered Phenol-Chloroform-Isoamylalcohol and chloroform. To the aqueous phase supernatant 2 μl of Glycoblue (20 mg/ml) (Invitrogen AM9516), 20 μl of 5 M NaCl and 500 μl ethanol were added and the DNA precipitated at −20 °C for >1 hour.

The DNA precipitate was centrifuged at 17,000xg for 20 min at 4 °C and the pellet washed with 500 μl cold 70% ethanol. The pellet was air-dried at RT (about 5 minutes) then resuspended in 20 μl H_2_O or TE. DNA concentration was measured using a Qubit (Life Technologies) with ssDNA kit (Molecular Probes Q10212).

### MeDIP-Seq Analysis

As previously described^30,54^, the MeDIP DNA was used to create libraries for next generation sequencing (NGS) using the NEBNext® Ultra™ RNA Library Prep Kit for Illumina® (NEB #E7530S) (San Diego, CA) starting at step 1.4 of the manufacturer’s protocol to generate double stranded DNA. After this step the manufacturer’s protocol was followed. Each sample received a separate index primer. NGS was performed at WSU Spokane Genomics Core using the Illumina HiSeq 2500 with a PE50 application, with a read size of approximately 50 bp. Six libraries were run in one lane of the sequencing cell with at least 40 million reads per pool.

### mRNA and ncRNA isolation

Total RNA (mRNA, lncRNA, rRNA, tRNA, and sncRNA) was extracted from purified prostate cells (n = 3 pooled samples of epithelium and 3 of stroma for each treatment group) using either the Trizol reagent (Thermo Fisher) or mirVana miRNA isolation kit (Life Technologies) following the manufacturer’s protocol with some modifications. Control lineage prostate cells were stored as a cell pellet at −80 °C until extraction with the mirVana kit. Cell pellets were manually homogenized and heated to 65 °C for 10 minutes after lysis buffer was added. The manufacturer’s protocol was then resumed. Vinclozolin lineage prostate cells were suspended in 1.2 mL of Trizol and stored at −80 °C until use. The manufacturer’s protocol was followed with the exception of increasing the amount of isopropanol added to 1 mL at the RNA precipitation step to recover the small RNA. RNA from both lineages was eluted in 50 μL of water with the addition of 0.5 μL murine RNase inhibitor (NEB).

Quality control analysis for both lineages was performed by running the RNA on an RNA 6000 Pico chip on the Agilent 2100 Bioanalyzer (Agilent). The Qubit RNA HS Assay Kit (Thermo Fisher) was used to determine RNA concentration.

### mRNA and ncRNA sequencing

As previously described^39^, large mRNA and noncoding RNA libraries were constructed from total RNA using the KAPA RNA HyperPrep Kit with RiboErase (KAPA) according to the manufacturer’s protocol, with some modifications. Barcodes and adaptors were from NEBNext Muliplex Oligos for Illumina. Prior to PCR amplification, libraries were incubated at 37 °C for 15 minutes with the USER enzyme (NEB). PCR cycle number was determined using qPCR with the KAPA RealTime Library Amplification kit before final amplification. Size selection (200–700 bp) was performed using KAPA Pure beads (KAPA). Quality control was performed using Agilent DNA High Sensitivity chips (Agilent) and concentration was determined using Qubit dsDNA high sensitivity assay (Thermo Fisher). Libraries with different barcodes were pooled (10 samples per pool by equal RNA content) and loaded onto an Illumina HiSeq 4000 sequencer on a paired-end 100 bp flow cell. Bioinformatics analysis was used to separate mRNA libraries from ncRNA libraries and to determine differential expression for each RNA class (see ncRNA bioinformatics section).

Small RNA libraries were constructed using the NEBNext Multiplex Small RNA Library Prep Set for Illumina and were barcoded with the NEBNext Multiplex Oligos for Illumina. After amplification, purification and size selection was performed using the KAPA Pure beads at 1.3x and 3.7x ratios following the manufacturer’s instructions. Final size selection (115–160 bp) was performed using the Pippin Prep 3% gel with marker P (Sage Science). Quality control was performed using Agilent DNA High Sensitivity chips (Agilent) and concentration was determined using Qubit dsDNA high sensitivity assay (Thermo Fisher). Libraries were pooled using equal RNA content and concentrated using 2.2x KAPA Pure beads and were loaded onto an Illumina HiSeq 4000 sequencer and sequenced with a single-end 50 bp flow cell. A customized primer was used to sequence the sRNA libraries: 5′-ACA CGT TCA GAG TTC TAC AGT CCG A-3′. Bioinformatics analysis was used to determine differential expression (see ncRNA bioinformatics section).

### DMR Statistics and Bioinformatics

As previously described^30^, the basic read quality was verified using summaries produced by the FastQC program. The data was cleaned and filtered to remove adapters and low-quality bases using Trimmomatic^55^. The reads for each MeDIP sample were mapped to the Rnor 6.0 rat genome using Bowtie2^56^ with default parameter options. The mapped read files were then converted to sorted BAM files using SAMtools^57^. To identify DMRs, the reference genome was broken into 100 bp windows. The MEDIPS R package^58^ was used to calculate differential coverage between control and exposure sample groups. The edgeR p-value^59^ was used to determine the relative difference between the two groups for each genomic window. Windows with an edgeR p-value less than an arbitrarily selected threshold were considered DMRs. The DMR edges were extended until no genomic window with an edgeR p-value less than 0.1 remained within 1000 bp of the DMR. CpG density and other information was then calculated for the DMR based on the reference genome. DMR clusters were identified as previously described^60^ (Supplemental Tables S1–S12).

DMRs were annotated using the biomaRt R package^61^ to access the Ensembl database^62^. The genes that overlapped with DMR were then input into the KEGG pathway search^63,64^ to identify associated pathways. The DMR associated genes were then sorted into functional groups by consulting information provided by the DAVID^65^, Panther^66^, and Uniprot databases incorporated into an internal curated database (www.skinner.wsu.edu under genomic data). All molecular data has been deposited into the public database at NCBI (GEO # GSE118447, SRA # PRJNA480506) and R code computational tools available at GitHub (https://github.com/skinnerlab/MeDIP-seq) and www.skinner.wsu.edu.

### ncRNA statistics and bioinformatics

As previously described^27^, the small ncRNA data were annotated as follows: Low- quality reads and reads shorter than 15nt were discarded by Trimmomatics (v0.33). The remaining reads were matched to known rat sncRNA, consisting of mature miRNA (miR- Base, release 21), precursor miRNA (miRBase, release 21), tRNA (Genomic tRNA Database, rn5), piRNA (piRBase), rRNA (Ensembl, release 76) and mitochondrial RNA (Ensembl, release 76) using AASRA pipeline with default parameters. Read counts generated by AASRA were statistically normalized by DESeq2.

The long ncRNA data were annotated as follows: Trimmomatics (v0.33) was used to remove adaptor sequences and the low-quality reads from the RNA sequencing data of the large RNA libraries. To identify all the transcripts, we used HiSAT2 (v2.1.0) and StringTie (v1.3.4d) to assemble the sequencing reads based on the Ensembl_Rnor_6.0. The differential expression analyses were performed by Cuffdiff. The coding and the non-coding genes were primarily annotated through rat CDS data ensembl_Rnor_6.0. The non-annotated genes were extracted through our in-house script and then analyzed by CPAT, indicating the true non-coding RNAs.

## Supplementary information

Supplemental Material
