# Supplementary material for: Environmental Toxicant Induced Epigenetic Transgenerational Inheritance of Prostate Pathology and Stromal-Epithelial Cell Epigenome and Transcriptome Alterations: Ancestral Origins of Prostate Disease
Source: Sci Rep. 2019 Feb 18;9:2209. doi: 10.1038/s41598-019-38741-1 (PMC6379561; doi:10.1038/s41598-019-38741-1)
Supplement: Supplementary file 1 — Supplemental Material [file 41598_2019_38741_MOESM1_ESM.pdf]

Environmental Toxicant Induced Epigenetic Transgenerational Inheritance of Prostate Pathology and Stromal-Epithelial Cell Epigenome and Transcriptome Alterations: Ancestral Origins of Prostate Disease

Rachel Klukovich[2+], Eric Nilsson[1+], Ingrid Sadler-Riggleman[1], Daniel Beck[1], Yeming Xie[2], Wei Yan[2++], Michael K. Skinner[1++]

[1]Center for Reproductive Biology
School of Biological Sciences
Washington State University
Pullman, WA, 99164-4236, USA

[2]Department of Physiology and Cell Biology
University of Nevada, Reno School of Medicine
Reno, NV, 89557, USA

(+) Co-first authors
(++) Co-senior authors / Corresponding authors

Supplemental Material

Supplemental Figure and Table Legends

Supplemental Figure S1. Representative rat ventral prostate histopathology from F3 generation vinclozolin lineage. (A) Normal prostate with single cuboidal to columnar epithelial layer for each gland. (B) Prostatic epithelial atrophy demonstrating flattened glandular epithelium (black arrow). (C) Epithelial layer vacuoles indicated by blue arrowheads showing empty spaces where epithelial cells have died or are missing. (D) Prostatic epithelial pseudo-stratification or hyperplasia (white arrow) showing multiple layers of epithelium were not counted as an abnormality. (E & F) Abnormal prostate hyperplasia (white arrows) were counted as histopathologies. White arrows showing where multiple layers of epithelium and hyperplasia are present. Black arrows indicate regions of epithelial atrophy. Scale bar = 100 μm.

Supplemental Figure S2. Kegg pathways in cancer. The blue circles indicate DMR association and red circles mRNA association.

Supplemental Table S1. Site table vinclozolin DMR prostate epithelium 1e-06.

Supplemental Table S2. Site table vinclozolin DMR prostate stromal 1e-06.

Supplemental Table S3. Overlap DMR epithelium and stromal.

Supplemental Table S4. Site table sncRNA vinclozolin epithelium 0.001.

Supplemental Table S5. Site table sncRNA vinclozolin stromal 0.001.

Supplemental Table S6. Site table sncRNA overlap epithelium vs. stromal 0.001.

Supplemental Table S7. Site table lncRNA prostate epithelium 0.001.

Supplemental Table S8. Site table lncRNA prostate stromal 0.001.

Supplemental Table S9. Site table lncRNA prostate stromal vs. epithelium overlaps 0.001.

Supplemental Table S10. Site table mRNA prostate epithelium 0.001.

Supplemental Table S11. Site table mRNA prostate stromal 0.001.

Supplemental Table S12. Site table mRNA prostate epithelium vs. stromal overlaps p<0.001.

Supplemental Table S13. Prostate disease associated genes from literature [67-85].

Supplemental Figure S1

Representative Prostate Histopathology

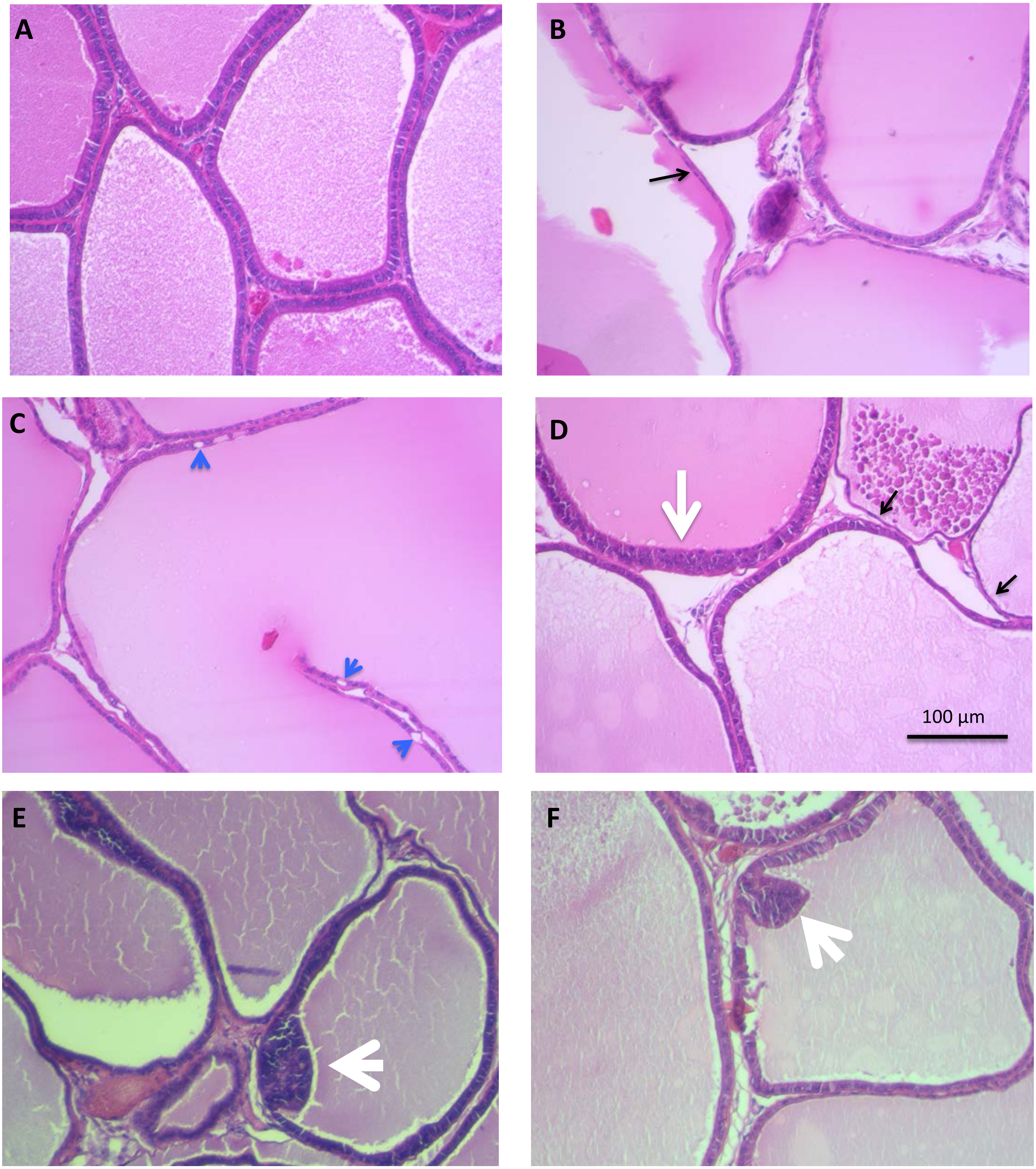

Supplemental Figure S2

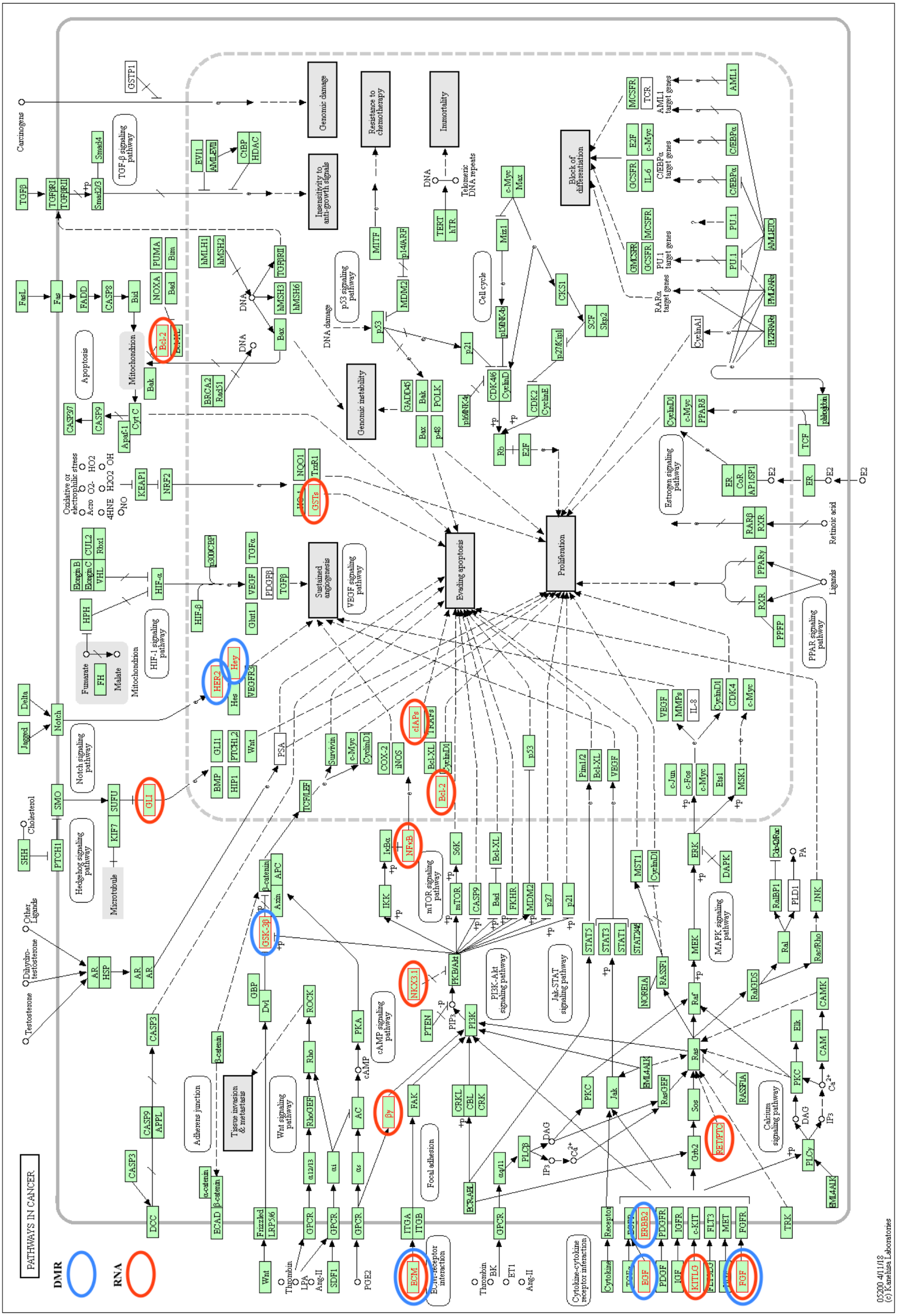

Supplemental Table S1
Prostate Epithelial Cell DMR List (p<10-6)

DMR Name	Chr	Start	Length	# SigWin	minP	Ratio Vin/Con	CpG #	CpG Density	Annotation	Category
DMR1:5738301	1	5738301	200	2	3.26E-15	0.045	0	0	SNORA17	
DMR1:14646701	1	14646701	1100	1	2.10E-07	0.349	6	0.5455		
DMR1:21877701	1	21877701	1100	1	3.40E-07	0.409	5	0.4545		
DMR1:22736601	1	22736601	400	1	2.19E-19	0.254	2	0.5	Slc18b1	
DMR1:26631101	1	26631101	300	2	1.27E-08	5.043	1	0.3333		
DMR1:38338201	1	38338201	900	2	1.61E-08	3.442	6	0.6667		
DMR1:38431701	1	38431701	1200	1	6.35E-08	0.506	9	0.75	pRNA	
DMR1:41961301	1	41961301	1200	1	3.14E-08	11.966	3	0.25	Syne1	Development
DMR1:47830701	1	47830701	400	1	2.61E-07	0.431	1	0.25		
DMR1:47883201	1	47883201	700	2	3.14E-08	0.298	10	1.4286		
DMR1:51417501	1	51417501	1300	1	6.15E-07	0.406	2	0.1538		
DMR1:55498001	1	55498001	300	1	1.05E-09	4.22	2	0.6667		
DMR1:70607201	1	70607201	800	1	2.81E-07	2.421	5	0.625		
DMR1:76761801	1	76761801	300	1	9.20E-10	0.218	2	0.6667	LOC100912485	
DMR1:78435101	1	78435101	300	1	4.57E-07	4.735	6	2	Npas1	Transcription
DMR1:79664801	1	79664801	700	1	4.98E-07	3.112	7	1	Mill1;5S_rRNA	
DMR1:96331501	1	96331501	800	1	7.34E-07	4.255	3	0.375		
DMR1:96344001	1	96344001	1000	1	6.71E-07	2.314	8	0.8		
DMR1:101589701	1	101589701	300	1	2.19E-07	0.349	1	0.3333	Fgf21;Fut1	Metabolism
DMR1:105581201	1	105581201	300	1	3.30E-07	0.35	1	0.3333	Nell1	Development
DMR1:116265801	1	116265801	1500	2	1.85E-07	2.288	20	1.3333		
DMR1:131238801	1	131238801	300	1	7.25E-07	0.407	0	0		
DMR1:143988101	1	143988101	300	1	4.48E-07	3.587	2	0.6667		
DMR1:159567101	1	159567101	400	1	8.96E-07	2.915	3	0.75		
DMR1:179199801	1	179199801	1900	1	1.35E-10	3.484	24	1.2632		
DMR1:179416901	1	179416901	2900	1	6.34E-07	2.418	24	0.8276		
DMR1:179687901	1	179687901	2500	2	2.76E-07	1.737	38	1.52		
DMR1:179697001	1	179697001	1700	1	6.23E-08	3.53	15	0.8824		
DMR1:180242101	1	180242101	2300	1	4.83E-09	3.24	36	1.5652		
DMR1:181560001	1	181560001	5300	1	6.20E-07	2.692	100	1.8868		
DMR1:197244801	1	197244801	400	1	8.76E-07	0.512	2	0.5	LOC361646	
DMR1:201861201	1	201861201	500	1	9.50E-07	0.349	5	1	AC123083.1	
DMR1:209021701	1	209021701	1200	1	8.35E-07	3.109	15	1.25		
DMR1:212412301	1	212412301	4200	1	1.10E-07	2.606	48	1.1429		
DMR1:212438801	1	212438801	600	1	9.66E-07	2.773	2	0.3333		
DMR1:223899701	1	223899701	1500	1	1.26E-07	3.505	35	2.3333		
DMR1:224072801	1	224072801	3500	1	3.35E-08	1.893	58	1.6571		
DMR1:225355401	1	225355401	200	1	1.00E-07	0.26	5	2.5		
DMR1:226232001	1	226232001	400	1	2.60E-07	5.35	8	2	Fads1	Metabolism
DMR1:229552801	1	229552801	300	2	5.29E-09	0.316	2	0.6667	Glyatl2	
DMR1:263684201	1	263684201	900	1	1.88E-07	0.506	1	0.1111	Dnmbp	EST
DMR1:280046301	1	280046301	700	1	1.44E-08	0.249	0	0		
DMR2:13591401	2	13591401	100	1	6.64E-07	4.012	2	2	Ccnh	Cell Cycle
DMR2:20994801	2	20994801	2500	1	9.63E-07	2.917	54	2.16		
DMR2:49066801	2	49066801	500	1	5.12E-08	0.49	5	1		
DMR2:68828601	2	68828601	100	1	1.04E-07	0.127	0	0	AABR07008655.1;Egf	Growth Factors & Cytokines
DMR2:94194201	2	94194201	400	1	1.63E-07	0.433	2	0.5		

DMR2:104058801	2	104058801	200	1	8.07E-07	7.562	2	1	Pde7a	Signaling
DMR2:114916901	2	114916901	100	1	6.54E-07	3.057	0	0		
DMR2:114943301	2	114943301	4200	2	5.82E-09	2.643	38	0.9048		
DMR2:116260401	2	116260401	100	1	7.98E-07	3.643	0	0	Samd7	
DMR2:134802501	2	134802501	400	1	1.50E-07	3.466	4	1		
DMR2:136116401	2	136116401	600	1	4.75E-08	0.169	7	1.1667		
DMR2:141753001	2	141753001	1300	1	6.06E-07	0.371	9	0.6923		
DMR2:141950101	2	141950101	700	2	4.10E-08	0.495	4	0.5714		
DMR2:145253701	2	145253701	200	1	7.66E-12	0.253	0	0		
DMR2:146483401	2	146483401	300	2	3.19E-09	0.254	3	1		
DMR2:161037301	2	161037301	700	2	3.55E-11	2.673	11	1.5714		
DMR2:161901901	2	161901901	1000	1	8.70E-09	2.057	10	1		
DMR2:162151301	2	162151301	2300	1	6.13E-08	5.368	29	1.2609		
DMR2:162414201	2	162414201	1300	2	1.35E-09	2.173	12	0.9231		
DMR2:195076801	2	195076801	1000	1	1.60E-07	2.803	2	0.2		
DMR2:206098801	2	206098801	500	1	3.26E-07	0.226	0	0	Syt6	Transport
DMR2:226852901	2	226852901	1400	1	6.20E-08	0.243	5	0.3571		
DMR2:256984001	2	256984001	300	1	8.29E-07	0.351	0	0	Ifi44l	Immune
DMR2:258729201	2	258729201	2000	1	7.19E-08	2.907	34	1.7		
DMR3:2243801	3	2243801	400	1	3.43E-07	0.524	0	0	Pnpla7	Metabolism
DMR3:2358201	3	2358201	500	1	6.37E-07	0.482	0	0		
DMR3:9264201	3	9264201	100	1	4.63E-07	4.55	1	1	Aif1l;Lamc3	Signaling;Extracellular Matrix
DMR3:23694201	3	23694201	200	2	7.25E-15	0.067	2	1		
DMR3:40739901	3	40739901	1200	1	1.41E-07	3.1	35	2.9167		
DMR3:41320201	3	41320201	300	1	4.26E-08	2.236	3	1		
DMR3:52071801	3	52071801	1200	1	1.36E-13	3.234	23	1.9167	Csrnp3	Unknown
DMR3:60951001	3	60951001	300	1	4.68E-07	0.538	2	0.6667		
DMR3:60963601	3	60963601	400	2	2.17E-10	11.608	6	1.5		
DMR3:64021701	3	64021701	300	1	2.40E-07	5.868	10	3.3333	Ccdc141	Cytoskeleton
DMR3:123623501	3	123623501	300	1	1.46E-07	0.327	3	1	Siglec1	Extracellular Matrix
DMR3:132612001	3	132612001	400	2	1.93E-08	0.399	0	0	Sptlc3	Metabolism
DMR3:133507101	3	133507101	300	1	9.67E-07	2.309	2	0.6667		
DMR3:139692901	3	139692901	400	1	2.58E-07	0.239	0	0	Slc24a3	Transport
DMR3:150569601	3	150569601	200	1	4.12E-07	0.422	0	0	Asip	Signaling
DMR3:159097301	3	159097301	500	1	2.82E-09	0.277	10	2	LOC103694889	
DMR3:159547401	3	159547401	1700	1	7.66E-07	4.495	33	1.9412		
DMR3:160779101	3	160779101	2100	1	3.31E-08	0.227	13	0.619	Slpil3	
DMR3:168670901	3	168670901	800	1	1.86E-08	7.667	6	0.75		
DMR3:168952801	3	168952801	300	1	6.78E-08	0.127	8	2.6667		
DMR3:168987001	3	168987001	400	1	5.07E-07	0.352	2	0.5		
DMR3:169465901	3	169465901	2300	1	6.80E-07	3.407	36	1.5652		
DMR3:174392401	3	174392401	800	2	1.83E-08	3.259	11	1.375		
DMR4:7597201	4	7597201	300	1	3.18E-07	0.318	1	0.3333		
DMR4:26098601	4	26098601	200	1	4.06E-08	6.706	1	0.5	Cdk14	Cell Cycle
DMR4:30466001	4	30466001	300	1	5.64E-07	0.461	1	0.3333	Asb4	Protein Binding
DMR4:32781301	4	32781301	200	1	9.80E-07	2.576	0	0		
DMR4:53250101	4	53250101	200	1	7.17E-07	0.34	1	0.5	AABR07060084.1	
DMR4:58622401	4	58622401	400	1	2.52E-07	8.238	4	1		
DMR4:71623201	4	71623201	200	1	6.09E-07	9.448	4	2	Gstk1	Binding Protein
DMR4:77401101	4	77401101	11200	1	1.10E-08	0.607	368	3.2857	AABR07060519.1	
DMR4:80765501	4	80765501	300	1	7.61E-07	0.351	3	1		

DMR4:115226301	4	115226301	1000	1	1.40E-07	6.508	5	0.5	Actg2;AC115473.1	Cytoskeleton
DMR4:125209901	4	125209901	2300	1	9.90E-07	2.96	29	1.2609	AABR07061448.2	
DMR4:126365101	4	126365101	300	1	4.99E-08	0.333	0	0		
DMR4:129949701	4	129949701	300	1	6.13E-08	5.035	3	1		
DMR4:147435301	4	147435301	800	1	1.29E-07	0.503	5	0.625		
DMR4:158928401	4	158928401	600	2	2.53E-10	0.307	5	0.8333		
DMR4:164695001	4	164695001	1100	1	3.39E-08	0.409	9	0.8182	Ly49s4;Ly49i2	Receptor
DMR4:173488601	4	173488601	300	3	4.62E-13	0.016	9	3		
DMR4:179152301	4	179152301	400	1	1.27E-09	0.451	1	0.25		
DMR4:180738701	4	180738701	600	1	3.98E-07	9.265	7	1.1667		
DMR5:491101	5	491101	1900	1	2.07E-07	3.358	14	0.7368		
DMR5:2614901	5	2614901	300	2	2.73E-07	2.353	29	9.6667	Rdh10;AABR07046713.1	Metabolism
DMR5:16658001	5	16658001	400	1	9.06E-07	4.333	7	1.75		
DMR5:19302001	5	19302001	200	1	1.62E-07	4.059	3	1.5	Ubxn2b	Proteolysis
DMR5:24896101	5	24896101	600	1	9.83E-07	3.768	5	0.8333	RGD1559441	Extracellular Matrix
DMR5:31779601	5	31779601	500	1	1.51E-09	28.04	5	1	Mmp16	Proteolysis
DMR5:45410601	5	45410601	500	1	1.04E-07	5.045	4	0.8		
DMR5:57391201	5	57391201	400	1	2.68E-07	0.354	1	0.25		
DMR5:60200801	5	60200801	400	1	1.06E-07	0.423	2	0.5	Pax5	Transcription
DMR5:111459301	5	111459301	1000	1	1.55E-09	5.135	15	1.5		
DMR5:118328601	5	118328601	300	1	1.38E-07	0.42	1	0.3333		
DMR5:121133101	5	121133101	3000	1	6.67E-07	2.733	72	2.4		
DMR5:124241401	5	124241401	600	1	7.05E-08	3.289	5	0.8333	Dab1	Signaling
DMR5:135836401	5	135836401	1300	1	4.47E-07	0.486	16	1.2308	Zswim5;AABR07073141.1	Transcription
DMR5:137138701	5	137138701	1100	1	8.53E-07	0.332	15	1.3636		
DMR5:138699301	5	138699301	2700	1	6.51E-07	0.4	15	0.5556	Guca2b	Signaling
DMR5:140941101	5	140941101	1000	1	8.18E-08	5.837	4	0.4	Heyl	Transcription
DMR5:147047301	5	147047301	600	1	7.25E-07	0.312	1	0.1667		
DMR5:148504701	5	148504701	1100	1	3.08E-07	0.404	7	0.6364	AABR07050017.2;AABR07050017.1;SCARNA16	
DMR5:152428601	5	152428601	200	1	5.23E-07	0.199	6	3	Umodl;Catsper4	Transport
DMR6:16260701	6	16260701	2300	1	7.16E-08	3.973	22	0.9565		
DMR6:16595801	6	16595801	3400	1	4.77E-07	2.472	48	1.4118		
DMR6:17053501	6	17053501	700	3	1.03E-07	4.793	16	2.2857		
DMR6:25293501	6	25293501	300	1	1.56E-07	3.591	4	1.3333	Srd5a2	Metabolism
DMR6:31062601	6	31062601	9300	2	3.77E-08	0.598	169	1.8172		
DMR6:38923701	6	38923701	800	1	5.93E-07	2.199	42	5.25		
DMR6:51253101	6	51253101	700	1	1.24E-07	0.392	2	0.2857	Hbp1	Transcription
DMR6:52528101	6	52528101	400	1	4.20E-07	4.918	11	2.75	Atxn7l1	Development
DMR6:65095701	6	65095701	200	1	7.04E-07	0.336	0	0	Stxbp6	Receptor
DMR6:130196601	6	130196601	800	1	9.38E-07	3.173	4	0.5	AABR07065476.2;AABR07065476.1	
DMR6:133952401	6	133952401	300	1	6.99E-07	6.413	4	1.3333		
DMR6:134504101	6	134504101	1100	2	3.28E-09	0.514	26	2.3636		
DMR6:137030301	6	137030301	400	1	1.40E-07	6.191	8	2		
DMR6:145762801	6	145762801	1000	1	1.74E-09	7.246	9	0.9	Cdca7l	
DMR7:21704301	7	21704301	1600	1	7.56E-07	4.393	8	0.5		
DMR7:21714101	7	21714101	2500	4	4.88E-08	1.979	38	1.52		
DMR7:21906601	7	21906601	1200	1	9.08E-07	3.231	4	0.3333		

DMR7:23624201	7	23624201	300	1	3.30E-07	0.328	15	5	Syn3	Development
DMR7:26840601	7	26840601	800	2	1.42E-08	5.398	5	0.625	Chst11	Metabolism
DMR7:30748101	7	30748101	1500	1	3.69E-07	7.084	5	0.3333	Anks1b	Receptor
DMR7:53502501	7	53502501	200	1	2.43E-08	2.801	1	0.5		
DMR7:114696001	7	114696001	200	1	5.47E-07	6.738	3	1.5	AABR07058406.1	
DMR7:115169901	7	115169901	600	1	6.62E-08	5.066	4	0.6667		
DMR7:119494001	7	119494001	900	1	3.38E-07	0.352	4	0.4444	Ncf4	Development
DMR7:120510801	7	120510801	300	1	5.30E-07	0.295	6	2	Baiap2l2;Pla2g6	Receptor;Metabolism
DMR7:121631101	7	121631101	200	2	1.59E-08	0.37	6	3		
DMR7:125729701	7	125729701	100	1	7.34E-07	0.244	0	0	Arhgap8	Signaling
DMR7:138862001	7	138862001	1000	1	2.31E-08	0.347	10	1		
DMR8:8224501	8	8224501	300	1	2.88E-07	0.472	10	3.3333	Cntn5	Extracellular Matrix
DMR8:10620301	8	10620301	3500	1	1.16E-07	3.577	21	0.6		
DMR8:10628701	8	10628701	900	1	1.15E-07	2.092	8	0.8889		
DMR8:12109201	8	12109201	800	1	1.46E-07	0.663	2	0.25	Maml2	Transcription
DMR8:14259101	8	14259101	600	2	8.22E-13	0.543	26	4.3333	Slc36a4	Transport
DMR8:23010501	8	23010501	600	1	2.75E-07	0.45	0	0	Ccdc151;Prkcsh	Signaling
DMR8:43681601	8	43681601	1200	1	9.92E-07	2.959	28	2.3333	Olr1323	
DMR8:43875701	8	43875701	1700	1	5.76E-07	3.085	73	4.2941	Olr1337	Receptor
DMR8:45106101	8	45106101	300	1	5.80E-07	0.488	0	0	RGD1309108	Unknown
DMR8:84748701	8	84748701	200	1	2.60E-07	5.529	1	0.5	Lrrc1	Unknown
DMR8:88267801	8	88267801	400	1	3.59E-07	0.504	3	0.75		
DMR8:125831901	8	125831901	400	1	3.97E-07	0.317	5	1.25		
DMR8:127473901	8	127473901	1100	3	2.12E-07	0.183	1	0.0909	Itga9	Extracellular Matrix
DMR8:130124301	8	130124301	300	1	4.38E-07	0.445	5	1.6667	Cck	Signaling
DMR9:10507301	9	10507301	400	1	9.67E-07	0.595	1	0.25		
DMR9:14792201	9	14792201	1400	1	7.65E-07	2.063	20	1.4286	Trem1	
DMR9:20296001	9	20296001	1500	1	8.21E-07	2.492	51	3.4		
DMR9:21455101	9	21455101	1700	1	1.72E-11	2.706	32	1.8824		
DMR9:21978901	9	21978901	500	1	9.26E-08	7.748	1	0.2		
DMR9:23467501	9	23467501	200	1	1.72E-08	10.164	3	1.5	Rhag	Transport
DMR9:31846801	9	31846801	100	1	2.23E-08	0.287	2	2	Adgrb3	
DMR9:37869001	9	37869001	800	1	7.06E-07	2.943	5	0.625		
DMR9:65973601	9	65973601	400	1	7.60E-07	4.92	4	1	Als2	Signaling
DMR9:97314501	9	97314501	2400	1	9.26E-07	1.645	25	1.0417		
DMR10:5412401	10	5412401	200	1	4.24E-09	0.303	0	0		
DMR10:18600401	10	18600401	1500	1	1.09E-07	2.69	7	0.4667	Kcnip1	Signaling
DMR10:27527501	10	27527501	300	1	4.47E-07	0.291	1	0.3333		
DMR10:37381101	10	37381101	500	3	7.34E-08	0.441	0	0		
DMR10:67005201	10	67005201	100	1	6.30E-07	6.065	3	3	Rab11fip4	Unknown
DMR10:80749001	10	80749001	400	1	8.69E-07	3.024	0	0		
DMR10:86380901	10	86380901	1100	1	3.39E-07	0.308	0	0	Erbb2;U6;Mien1	Receptor
DMR10:87973701	10	87973701	300	1	2.74E-07	0.353	0	0		
DMR10:88237001	10	88237001	200	1	4.56E-07	0.417	2	1	Eif1;Gast	Translation;Signaling
DMR10:95125601	10	95125601	500	1	7.62E-08	0.406	2	0.4	AABR07030593.1	
DMR10:97784701	10	97784701	300	1	2.05E-07	0.223	0	0	Arsg	Metabolism
DMR10:101715601	10	101715601	700	1	2.94E-07	0.407	4	0.5714		
DMR10:106589301	10	106589301	500	1	6.68E-08	0.424	5	1		

DMR11:65056801	11	65056801	500	1	1.20E-07	0.414	1	0.2	Nr1i2;Gsk3b	Receptor;Signaling
DMR11:81952601	11	81952601	300	1	6.94E-07	5.868	5	1.6667		
DMR12:1341101	12	1341101	600	1	7.56E-07	4.573	6	1		
DMR12:6654001	12	6654001	600	1	2.01E-07	0.376	3	0.5		
DMR12:8137201	12	8137201	200	1	4.01E-10	0.098	2	1	Mtus2	Cytoskeleton
DMR12:11273501	12	11273501	400	2	7.05E-11	0.314	9	2.25	Arpc1b;Arpc1a	Cytoskeleton
DMR12:12601401	12	12601401	1000	1	4.86E-11	4.169	4	0.4		
DMR12:21240601	12	21240601	600	1	1.47E-07	2.989	13	2.1667		
DMR12:22535701	12	22535701	1300	1	2.39E-08	0.491	16	1.2308	AABR07035790.1;AABR07035790.2;AABR07035791.1	
DMR12:38188801	12	38188801	1300	1	4.47E-07	0.137	23	1.7692		
DMR12:40097301	12	40097301	400	1	1.56E-07	0.373	1	0.25	Cux2	Development
DMR12:42433301	12	42433301	400	1	8.54E-08	0.334	0	0		
DMR12:44893501	12	44893501	400	1	6.13E-07	0.497	5	1.25	Ksr2;Rn50_12_0468.1;SNORA44	Signaling
DMR12:45279801	12	45279801	300	1	7.25E-08	3.073	2	0.6667		
DMR12:45281401	12	45281401	300	1	1.64E-09	0.093	0	0		
DMR12:45891101	12	45891101	1200	1	6.80E-07	3.376	15	1.25	Srrm4	Translation
DMR12:46622801	12	46622801	800	1	3.09E-07	0.377	11	1.375	Bicdl1	
DMR12:50526701	12	50526701	900	4	4.00E-13	2.441	9	1		
DMR12:50774501	12	50774501	2900	2	8.59E-08	3.237	47	1.6207		
DMR13:19628701	13	19628701	2400	2	3.28E-09	3.495	37	1.5417		
DMR13:19657901	13	19657901	2500	3	2.55E-08	1.863	38	1.52		
DMR13:19663701	13	19663701	2000	3	2.79E-07	2.798	9	0.45		
DMR13:50182201	13	50182201	600	1	2.14E-10	0.445	5	0.8333	Lax1	
DMR13:70572601	13	70572601	1800	1	6.87E-09	0.515	20	1.1111	Lamc2	Cytoskeleton
DMR13:75158801	13	75158801	1200	1	3.99E-07	0.327	12	1	Rn50_13_0801.2;Rn50_13_0801.3	
DMR13:75608801	13	75608801	200	1	1.35E-07	0.26	0	0		
DMR13:93431701	13	93431701	400	1	5.39E-09	0.447	15	3.75		
DMR14:1157901	14	1157901	1300	1	9.52E-07	6.297	34	2.6154		
DMR14:3415101	14	3415101	2100	1	1.03E-07	0.442	9	0.4286	Brdt	Epigenetic
DMR14:8435201	14	8435201	300	1	5.47E-07	6.423	7	2.3333	Arhgap24;Rn60_14_0085.1	Unknown
DMR14:22407301	14	22407301	2100	1	5.74E-07	2.967	12	0.5714	Ugt2a1;Ugt2b37	Metabolism
DMR14:24970001	14	24970001	1100	1	7.40E-07	3.236	37	3.3636		
DMR14:28201301	14	28201301	300	1	7.56E-07	4.341	1	0.3333		
DMR14:33641001	14	33641001	500	1	7.43E-07	2.914	12	2.4	Aasdh;RGD1311575	Metabolism
DMR14:46551601	14	46551601	4000	1	2.28E-07	0.592	60	1.5		
DMR14:46590301	14	46590301	25100	1	2.64E-07	0.61	555	2.2112	pRNA;AABR07015066.1;LOC257642;AABR07015067.1	
DMR14:94809901	14	94809901	400	1	8.25E-07	1.94	7	1.75		
DMR14:94878701	14	94878701	1200	1	4.84E-07	3.627	15	1.25		
DMR14:96914301	14	96914301	500	1	1.68E-07	2.154	15	3		
DMR15:2193401	15	2193401	300	2	6.67E-08	0.465	0	0		
DMR15:2943801	15	2943801	500	1	3.66E-08	5.511	7	1.4	Kat6b	Epigenetic
DMR15:21524001	15	21524001	2100	1	5.71E-07	2.691	19	0.9048		
DMR15:21762201	15	21762201	1200	1	4.00E-08	3.609	10	0.8333		
DMR15:23010301	15	23010301	3900	3	1.94E-08	2.027	79	2.0256		
DMR15:23024501	15	23024501	1100	2	2.03E-10	1.999	15	1.3636		

DMR15:35956601	15	35956601	200	1	9.18E-07	0.245	1	0.5	AC114070.1	
DMR15:40212401	15	40212401	1000	1	7.67E-07	0.376	2	0.2	Atp8a2	Transport
DMR15:42728801	15	42728801	900	1	2.83E-07	3.45	19	2.1111	Adam2	Protease
DMR15:51527001	15	51527001	1200	1	4.47E-07	7.678	9	0.75	Pebp4	Binding Protein
DMR15:65004501	15	65004501	2400	1	2.93E-08	3.651	34	1.4167		
DMR15:65159001	15	65159001	800	1	6.38E-08	4.464	2	0.25		
DMR15:65193301	15	65193301	300	1	2.70E-07	2.836	0	0	AABR07018574.1	
DMR15:65198801	15	65198801	5400	1	7.83E-10	2.962	63	1.1667	AABR07018574.1	
DMR15:65208101	15	65208101	1400	1	5.26E-07	2.478	25	1.7857		
DMR15:71954201	15	71954201	600	1	8.78E-07	3.648	17	2.8333		
DMR15:100725101	15	100725101	200	1	4.41E-09	0.307	2	1		
DMR15:106781201	15	106781201	600	1	7.30E-07	0.463	2	0.3333		
DMR16:1819501	16	1819501	700	1	3.10E-07	3.514	16	2.2857	Zmiz1;Mir3075	Metabolism
DMR16:17679801	16	17679801	400	1	8.45E-07	0.483	0	0	Sh2d4b	
DMR16:20898801	16	20898801	400	1	2.49E-08	8.511	2	0.5	Homer3	Signaling
DMR16:30801501	16	30801501	2000	1	9.07E-07	2.48	22	1.1		
DMR16:46792901	16	46792901	300	1	1.70E-07	4.969	4	1.3333	Tenm3	
DMR16:79332301	16	79332301	200	1	5.47E-07	0.174	4	2		
DMR16:80298801	16	80298801	100	1	5.09E-07	0.134	0	0		
DMR17:5516201	17	5516201	1700	1	7.64E-07	0.4	26	1.5294	Agtpbp1	Signaling
DMR17:23552901	17	23552901	300	1	4.83E-07	0.245	2	0.6667		
DMR17:43355501	17	43355501	300	1	1.07E-07	0.344	1	0.3333	Scgn	Signaling
DMR17:63750201	17	63750201	200	1	3.18E-07	0.32	1	0.5	Dip2c	
DMR17:72404401	17	72404401	2100	1	2.44E-07	11.509	48	2.2857		
DMR17:77710201	17	77710201	700	1	6.74E-08	15	8	1.1429		
DMR17:81182401	17	81182401	1200	1	1.35E-07	0.513	21	1.75	Hacd1	
DMR17:82084601	17	82084601	1200	1	1.37E-07	0.471	22	1.8333	Arl5b	Translation
DMR17:86588801	17	86588801	100	1	5.48E-07	0.203	2	2	AABR07028793.1	
DMR18:15602601	18	15602601	200	1	2.41E-07	3.355	8	4	Dsg2	Extracellular Matrix
DMR18:51384401	18	51384401	700	1	6.94E-07	2.088	13	1.8571		
DMR18:64122001	18	64122001	900	1	7.66E-07	0.243	9	1	Mc5r	Receptor
DMR18:72400001	18	72400001	500	1	1.40E-07	6.243	6	1.2	Zbtb7c	Transcription
DMR18:75150001	18	75150001	400	1	9.28E-07	0.382	1	0.25	Setbp1	Transcription
DMR18:78952501	18	78952501	800	1	8.24E-07	0.458	2	0.25	AABR07032747.1	
DMR18:79089601	18	79089601	800	1	4.09E-07	0.196	21	2.625		
DMR18:79299701	18	79299701	600	1	1.94E-07	0.459	1	0.1667		
DMR18:79438201	18	79438201	1100	1	2.24E-07	2.645	10	0.9091	Mbp	Unknown
DMR18:79804201	18	79804201	500	1	4.27E-08	0.377	0	0	Zfp516	Transcription
DMR18:80608001	18	80608001	2500	1	7.77E-10	0.376	71	2.84		
DMR18:87797401	18	87797401	900	1	9.01E-12	2.744	9	1		
DMR18:87820801	18	87820801	100	1	1.07E-07	9.643	1	1		
DMR18:87853601	18	87853601	900	1	3.35E-07	2.061	39	4.3333		
DMR19:3943801	19	3943801	300	1	9.85E-08	3.062	1	0.3333		
DMR19:14401801	19	14401801	100	1	3.01E-09	0.129	2	2	Tom1	Signaling
DMR19:15937901	19	15937901	400	1	2.49E-08	0.149	0	0		
DMR19:18012901	19	18012901	200	1	5.86E-07	0.323	1	0.5	AABR07043031.3	
DMR19:38934801	19	38934801	700	3	2.18E-13	0.193	3	0.4286	Tango6	
DMR19:53473901	19	53473901	500	1	6.53E-07	0.542	6	1.2	LOC687560	
DMR19:54053501	19	54053501	500	2	5.63E-07	0.513	2	0.4	Gse1	Transcription
DMR19:57373401	19	57373401	700	1	5.36E-07	0.367	4	0.5714	Capn9	Protease

DMR20:3145001	20	3145001	500	1	3.03E-09	0.448	4	0.8	Rps2-ps2;RGD1562652;AABR07044362.1;RT1-N2	Immune
DMR20:3164201	20	3164201	300	2	3.21E-10	0.35	1	0.3333	AABR07044362.1;RT1-S2;Rps2-ps1;Rn50_20_0053.5;RT1-N3;Rn60_20_0032.5	Immune
DMR20:3526801	20	3526801	500	1	1.65E-07	0.332	4	0.8	AABR07044367.1	
DMR20:3684701	20	3684701	400	1	6.73E-07	0.44	3	0.75	Mt1m;Rn50_20_0058.4	Receptor
DMR20:11692501	20	11692501	300	1	4.86E-07	9.023	4	1.3333	Ube2g2	Metabolism
DMR20:13017301	20	13017301	300	1	7.53E-07	3.413	6	2	Pcnt	Signaling
DMR20:40405401	20	40405401	300	2	1.41E-07	0.441	1	0.3333	Hs3st5	Metabolism
DMR20:40852401	20	40852401	200	1	2.34E-07	3.044	0	0		
DMR20:44787701	20	44787701	300	1	3.17E-07	0.474	0	0		
DMR20:45007301	20	45007301	200	1	3.41E-09	0.427	1	0.5	Mfsd4b;RGD1304770	
DMR20:50997701	20	50997701	400	1	1.66E-07	0.554	0	0		
DMRX:291701	X	291701	900	1	3.38E-07	2.872	10	1.1111		
DMRX:5511601	X	5511601	600	1	2.99E-08	3.732	7	1.1667		
DMRX:36455601	X	36455601	800	2	5.86E-08	2.717	17	2.125		
DMRX:114716501	X	114716501	300	1	3.59E-07	0.382	0	0		
DMRX:136769601	X	136769601	200	1	8.35E-08	0.171	0	0		

Supplemental Table S2

Prostate Stromal Cell DMR List (p<10-6)

DMR Name	Chr	Start	Length	# SigWin	minP	Ratio Vin/Con	CpG #	CpG Density	Annotation	Category
DMR1:48701	1	48701	1800	1	1.59E-07	0.611	25	1.3889		
DMR1:270401	1	270401	600	1	7.14E-07	0.63	4	0.6667		
DMR1:5738301	1	5738301	100	1	1.41E-17	0.012	0	0	SNORA17	
DMR1:6122201	1	6122201	500	2	8.72E-09	0.511	0	0		
DMR1:7175601	1	7175601	2600	1	4.79E-07	3.681	22	0.8462	AABR07000257.1	
DMR1:9030201	1	9030201	900	2	2.98E-08	2.3	2	0.2222		
DMR1:9169101	1	9169101	1200	1	9.83E-08	0.491	8	0.6667		
DMR1:11916701	1	11916701	3900	1	2.72E-08	0.619	55	1.4103	pRNA	
DMR1:11935001	1	11935001	1100	1	8.76E-08	0.631	21	1.9091		
DMR1:11963101	1	11963101	4900	3	1.92E-08	0.639	564	11.5102	pRNA;AABR07000398.1;5_8S_rRNA;AABR07000402.1;AABR07000404.1	
DMR1:15471001	1	15471001	500	1	5.47E-07	0.416	1	0.2	Map3k5	Signaling
DMR1:16370701	1	16370701	500	1	6.23E-08	0.323	10	2		
DMR1:16748201	1	16748201	400	2	1.96E-09	0.533	0	0		
DMR1:17802601	1	17802601	100	1	3.64E-07	0.186	0	0	Ptprk	Signaling
DMR1:18084901	1	18084901	1000	2	9.82E-09	0.558	7	0.7	AABR07000574.1	
DMR1:19202501	1	19202501	300	1	1.63E-08	0.329	1	0.3333		
DMR1:21732401	1	21732401	400	1	3.87E-11	0.241	1	0.25		
DMR1:21755001	1	21755001	1300	4	1.42E-09	0.648	9	0.6923	Enpp1	Signaling
DMR1:22545101	1	22545101	1100	1	3.80E-07	0.57	11	1	Taar6;Taar5	Receptor
DMR1:22736501	1	22736501	300	1	8.68E-16	0.251	0	0	Slc18b1	
DMR1:23129101	1	23129101	400	2	3.37E-10	0.486	0	0		
DMR1:23248901	1	23248901	500	1	9.90E-10	0.579	2	0.4		
DMR1:26844401	1	26844401	1500	1	5.26E-11	0.625	18	1.2		
DMR1:30195301	1	30195301	300	1	7.51E-07	0.247	5	1.6667	AABR07000968.2	
DMR1:32011001	1	32011001	400	1	5.40E-08	0.403	0	0	Trip13	Unknown
DMR1:32067401	1	32067401	400	1	8.29E-07	0.342	11	2.75	Nkd2	
DMR1:32168101	1	32168101	300	1	1.93E-08	0.43	8	2.6667		
DMR1:34883301	1	34883301	600	1	8.68E-07	0.558	5	0.8333		
DMR1:37392901	1	37392901	300	2	4.35E-07	3.991	3	1		
DMR1:39004001	1	39004001	3300	1	9.75E-07	3.764	60	1.8182	AABR07001233.1	
DMR1:39467501	1	39467501	3400	1	3.83E-07	0.648	142	4.1765		
DMR1:40429501	1	40429501	300	1	8.67E-09	0.416	0	0	Plekhg1	Signaling
DMR1:44888301	1	44888301	700	4	9.40E-17	0.534	1	0.1429		
DMR1:45091701	1	45091701	400	1	6.50E-07	0.488	1	0.25		
DMR1:52454001	1	52454001	1700	1	3.88E-07	2.405	33	1.9412	Pde10a	Signaling
DMR1:52714201	1	52714201	800	1	3.94E-11	0.64	10	1.25		
DMR1:53046001	1	53046001	600	1	2.92E-07	0.467	4	0.6667	Mpc1	
DMR1:53344501	1	53344501	600	3	2.31E-17	0.498	1	0.1667		
DMR1:53670601	1	53670601	300	1	4.40E-08	0.627	1	0.3333		
DMR1:54041301	1	54041301	100	1	4.62E-07	4.913	0	0	RGD1560718	Unknown
DMR1:54078501	1	54078501	1200	1	6.27E-07	0.199	13	1.0833		
DMR1:54088801	1	54088801	500	1	3.43E-07	0.401	3	0.6		
DMR1:54180101	1	54180101	900	1	4.91E-07	0.502	17	1.8889		
DMR1:54244401	1	54244401	800	4	1.56E-13	0.628	11	1.375	AABR07001610.1	
DMR1:54392201	1	54392201	1700	1	3.23E-07	0.365	29	1.7059		
DMR1:54417201	1	54417201	4400	1	8.35E-08	0.52	71	1.6136		
DMR1:54824701	1	54824701	1500	1	9.40E-07	0.62	18	1.2		
DMR1:56038001	1	56038001	1300	2	7.83E-09	0.648	13	1		
DMR1:57083201	1	57083201	1500	2	1.42E-09	0.585	11	0.7333		
DMR1:57456101	1	57456101	900	1	9.46E-07	0.592	0	0		
DMR1:63743701	1	63743701	400	1	5.44E-07	7.977	1	0.25	Nilr1	
DMR1:64430001	1	64430001	200	1	5.19E-07	0.484	2	1	Prkcg;Myadm	Binding Protein;Development

DMR1:65946601	1	65946601	200	1	5.52E-07	0.362	0	0	Vom2r34;Vom2r33	
DMR1:66080801	1	66080801	800	1	2.53E-08	0.505	10	1.25	Vom2r34	
DMR1:70135901	1	70135901	400	1	5.00E-07	0.616	0	0	Mir3099	
DMR1:77267901	1	77267901	1000	1	8.53E-07	0.444	4	0.4		
DMR1:91332701	1	91332701	400	1	5.12E-08	0.472	2	0.5		
DMR1:91403001	1	91403001	2100	2	1.36E-08	0.579	30	1.4286		
DMR1:92255001	1	92255001	1600	1	2.21E-07	3.854	19	1.1875		
DMR1:92860501	1	92860501	300	2	3.58E-09	0.494	3	1		
DMR1:92984301	1	92984301	300	1	3.95E-07	0.592	8	2.6667		
DMR1:94508501	1	94508501	1600	2	1.74E-09	0.43	20	1.25		
DMR1:94645701	1	94645701	200	1	1.70E-07	0.632	0	0		
DMR1:97067901	1	97067901	500	1	8.54E-07	0.45	4	0.8	AABR07003167.1	
DMR1:97414301	1	97414301	800	1	5.49E-08	0.465	7	0.875		
DMR1:98561601	1	98561601	600	1	5.72E-07	0.413	0	0	Siglec5	Immune
DMR1:101743401	1	101743401	2000	1	1.30E-07	0.477	14	0.7	Sult2b1	Metabolism
DMR1:105083701	1	105083701	300	1	8.60E-07	0.478	1	0.3333		
DMR1:105461001	1	105461001	1200	2	1.02E-07	0.516	19	1.5833	Nell1	Development
DMR1:124484201	1	124484201	1000	1	4.34E-07	0.533	12	1.2	Hmgn5b;U1	
DMR1:127163501	1	127163501	200	1	9.54E-07	3.586	0	0	Lrrk1	Unknown
DMR1:127460801	1	127460801	500	1	3.66E-07	0.562	1	0.2		
DMR1:128908001	1	128908001	300	1	7.61E-07	4.853	3	1		
DMR1:131257101	1	131257101	800	1	3.11E-07	0.587	4	0.5		
DMR1:133649201	1	133649201	400	1	2.19E-07	0.333	1	0.25		
DMR1:135570101	1	135570101	300	1	1.68E-07	0.52	0	0		
DMR1:136944501	1	136944501	300	1	6.14E-08	0.247	1	0.3333		
DMR1:139098401	1	139098401	300	1	3.13E-07	0.361	1	0.3333		
DMR1:141296301	1	141296301	400	3	9.80E-14	0.521	0	0		
DMR1:142293701	1	142293701	300	1	1.04E-07	0.65	0	0	Blm	Transcription
DMR1:142899001	1	142899001	700	1	2.49E-07	0.486	3	0.4286	Alpk3;AABR07004437.1	Cytoskeleton
DMR1:143826901	1	143826901	1600	1	3.63E-08	0.325	17	1.0625	Bnc1	Development
DMR1:145931901	1	145931901	300	2	2.66E-08	0.533	1	0.3333	Cfap161	
DMR1:147047001	1	147047001	200	1	9.75E-07	0.294	3	1.5	Xlr3a	
DMR1:148445201	1	148445201	2800	1	1.69E-07	0.357	128	4.5714	Vbp1	Protein Binding
DMR1:148461401	1	148461401	2800	2	3.07E-09	0.539	71	2.5357		
DMR1:148469601	1	148469601	1000	2	2.73E-07	0.626	20	2		
DMR1:148620501	1	148620501	1200	1	6.53E-07	0.575	28	2.3333		
DMR1:148622801	1	148622801	1900	1	1.76E-07	0.62	31	1.6316		
DMR1:148626401	1	148626401	1100	1	2.01E-08	0.617	16	1.4545		
DMR1:148632701	1	148632701	2200	8	7.70E-12	0.626	29	1.3182		
DMR1:148635901	1	148635901	1900	1	9.51E-07	0.621	44	2.3158		
DMR1:148650601	1	148650601	3200	3	1.14E-07	0.628	88	2.75		
DMR1:153395701	1	153395701	400	1	7.75E-07	0.488	2	0.5		
DMR1:160733801	1	160733801	1200	1	7.12E-07	2.592	14	1.1667	U6	
DMR1:164715401	1	164715401	200	1	5.44E-07	0.163	2	1	Olr36;Olr37	
DMR1:166547101	1	166547101	700	3	5.51E-09	0.456	2	0.2857	Pde2a	Signaling
DMR1:167953301	1	167953301	800	1	2.77E-12	0.608	0	0	Olr56;AC096030.2	
DMR1:170545801	1	170545801	900	1	3.38E-07	0.512	0	0	Dnhd1	
DMR1:178864701	1	178864701	300	1	8.22E-07	0.472	1	0.3333	Spon1	Growth Factors & Cytokines
DMR1:179353701	1	179353701	300	1	4.60E-07	4.989	2	0.6667		
DMR1:179687901	1	179687901	3700	4	1.80E-08	1.929	43	1.1622		
DMR1:179693501	1	179693501	2200	2	3.12E-07	4.289	12	0.5455		
DMR1:179846001	1	179846001	200	1	5.76E-08	9.143	0	0		
DMR1:179887701	1	179887701	1300	1	8.12E-07	3.022	6	0.4615		
DMR1:180242001	1	180242001	2300	1	1.02E-12	4.019	36	1.5652		
DMR1:181851201	1	181851201	3400	1	1.87E-08	4.13	54	1.5882		
DMR1:182797001	1	182797001	2700	1	4.18E-08	0.573	68	2.5185		
DMR1:185199001	1	185199001	400	1	5.86E-07	0.467	0	0		
DMR1:185552501	1	185552501	500	3	3.13E-11	0.618	3	0.6	RGD1311703	

DMR1:188463201	1	188463201	100	1	3.37E-07	13.102	1	1	LOC361635	Unknown
DMR1:195694701	1	195694701	400	1	4.41E-07	0.697	2	0.5		
DMR1:196430001	1	196430001	500	2	4.67E-09	0.394	4	0.8		
DMR1:197336001	1	197336001	500	1	3.84E-07	0.488	2	0.4		
DMR1:198606501	1	198606501	400	1	7.59E-09	0.552	0	0		
DMR1:202370501	1	202370501	200	1	1.80E-07	0.302	0	0		
DMR1:203068501	1	203068501	700	1	3.93E-07	0.502	2	0.2857		
DMR1:204045201	1	204045201	300	1	1.93E-07	0.33	0	0	Cpxm2	Proteolysis
DMR1:205537801	1	205537801	300	1	3.34E-07	0.571	4	1.3333		
DMR1:210195801	1	210195801	300	1	5.35E-07	0.622	0	0		
DMR1:211667001	1	211667001	300	1	1.63E-07	0.307	1	0.3333		
DMR1:214610701	1	214610701	5500	1	5.24E-07	0.698	133	2.4182	Ap2a2;Muc6	Receptor;Extracellular Matrix
DMR1:215360501	1	215360501	400	1	5.15E-09	0.36	1	0.25	Krtap5-2;Gm4559	
DMR1:215466201	1	215466201	1400	1	7.67E-07	0.559	12	0.8571	LOC685544	
DMR1:215660701	1	215660701	300	1	7.33E-08	0.282	7	2.3333	Lsp1;AC132720.1;Tnnt3	Cytoskeleton
DMR1:216242401	1	216242401	400	1	1.24E-08	0.424	1	0.25	Cd81	Signaling
DMR1:217215501	1	217215501	300	1	3.82E-09	0.341	3	1	Shank2	Protein Binding
DMR1:218338701	1	218338701	400	1	3.55E-08	0.456	4	1		
DMR1:218883301	1	218883301	400	1	6.06E-07	0.197	2	0.5	Lrp5	Receptor
DMR1:219309601	1	219309601	1100	1	1.07E-07	0.385	18	1.6364	RGD1307603	
DMR1:221469301	1	221469301	600	1	4.88E-09	0.544	2	0.3333	LOC100910252;Naaladl1	Protease
DMR1:221778701	1	221778701	800	1	7.00E-08	0.426	0	0	Pygm;Rasgrp2	Signaling
DMR1:222324301	1	222324301	400	1	6.97E-07	0.47	1	0.25	Macrod1	
DMR1:225219001	1	225219001	400	1	9.60E-08	0.591	0	0	Ahnak	Cytoskeleton
DMR1:226173301	1	226173301	300	1	6.03E-07	0.541	0	0		
DMR1:226415301	1	226415301	200	1	6.99E-07	0.503	0	0		
DMR1:238946001	1	238946001	400	1	4.91E-08	0.337	1	0.25		
DMR1:244449301	1	244449301	200	2	8.72E-11	0.55	1	0.5	AABR07006654.1;AABR07006654.2	
DMR1:247657501	1	247657501	1600	1	1.37E-07	0.564	24	1.5		
DMR1:252934301	1	252934301	500	1	2.85E-07	8.494	3	0.6	Ifit1	Immune
DMR1:253745301	1	253745301	400	1	6.32E-09	0.548	1	0.25		
DMR1:254626801	1	254626801	200	1	1.96E-07	0.294	2	1	Htr7	Receptor
DMR1:254797001	1	254797001	600	3	4.99E-11	0.534	0	0		
DMR1:254996401	1	254996401	1300	2	1.63E-07	0.266	24	1.8462	Pcgf5	Epigenetic
DMR1:255093801	1	255093801	1000	1	1.40E-09	0.379	16	1.6	Pcgf5	Epigenetic
DMR1:256444201	1	256444201	600	1	1.15E-07	0.621	2	0.3333	AC096353.1	
DMR1:256729701	1	256729701	200	1	4.51E-07	0.613	0	0	Myof	Cytoskeleton
DMR1:256983501	1	256983501	400	1	3.36E-08	0.273	3	0.75	Lgi1	Receptor
DMR1:260358101	1	260358101	1400	1	1.26E-08	0.462	14	1	Tll2	Protease
DMR1:260584201	1	260584201	500	1	1.33E-07	0.455	0	0	Pik3ap1	
DMR1:260950301	1	260950301	1500	1	1.78E-07	7.495	10	0.6667	Slit1	Receptor
DMR1:261495501	1	261495501	200	1	4.02E-07	0.547	2	1	Golga7b	
DMR1:263684301	1	263684301	800	1	5.25E-07	0.548	0	0	Dnmbp	EST
DMR1:264556301	1	264556301	1200	2	1.08E-09	0.574	12	1	Pax2	Transcription
DMR1:264588201	1	264588201	300	2	3.10E-11	0.6	1	0.3333	Pax2	Transcription
DMR1:265803801	1	265803801	400	1	7.96E-07	0.424	2	0.5	Ldb1;Pprc1	Transcription;Signaling
DMR1:266459101	1	266459101	1000	2	2.76E-09	0.49	10	1	Borcs7	
DMR1:267342101	1	267342101	300	1	3.70E-07	0.471	6	2		
DMR1:273065701	1	273065701	400	1	1.83E-07	0.395	2	0.5	AABR07006957.1	
DMR1:274268101	1	274268101	400	1	1.21E-07	0.605	2	0.5	Dusp5	Signaling
DMR1:276052201	1	276052201	400	1	5.81E-07	0.395	1	0.25		
DMR1:278286301	1	278286301	300	1	6.45E-08	0.473	1	0.3333		
DMR1:278457401	1	278457401	900	1	6.45E-08	0.531	2	0.2222		
DMR1:278891501	1	278891501	200	1	1.83E-07	0.503	0	0		
DMR1:279925401	1	279925401	400	1	2.17E-08	0.442	13	3.25	Pnliprp2	Unknown
DMR1:280030701	1	280030701	1500	1	3.79E-08	0.336	9	0.6		
DMR1:280046301	1	280046301	900	1	2.52E-07	0.284	0	0		

DMR2:185501	2	185501	500	1	4.47E-07	0.599	4	0.8		
DMR2:4064001	2	4064001	500	1	3.02E-07	0.557	0	0	Mctp1	Unknown
DMR2:4424201	2	4424201	200	1	1.71E-08	14.612	0	0	RGD1560883	Unknown
DMR2:4497101	2	4497101	2600	3	5.03E-17	0.509	3	0.1154	RGD1560883	Unknown
DMR2:7298701	2	7298701	500	1	4.52E-07	0.618	5	1		
DMR2:8942201	2	8942201	1200	2	1.70E-07	0.406	3	0.25	Adgrv1	
DMR2:9253901	2	9253901	500	1	4.41E-07	0.56	4	0.8	Adgrv1	
DMR2:9833201	2	9833201	300	1	7.58E-10	0.347	0	0		
DMR2:17316201	2	17316201	600	1	8.48E-07	0.568	3	0.5		
DMR2:20274701	2	20274701	200	1	3.73E-07	0.371	4	2		
DMR2:20578801	2	20578801	200	2	3.17E-08	0.396	0	0	Ssbp2	Transcription
DMR2:20788501	2	20788501	600	1	7.33E-07	0.336	10	1.6667		
DMR2:21025801	2	21025801	1500	1	4.34E-07	4.522	18	1.2		
DMR2:24756901	2	24756901	400	2	1.01E-12	0.593	1	0.25	Pde8b	Signaling
DMR2:28673701	2	28673701	300	2	1.88E-07	0.49	0	0		
DMR2:30978401	2	30978401	2300	1	1.90E-07	0.658	14	0.6087		
DMR2:42015701	2	42015701	1300	2	4.53E-08	0.302	22	1.6923		
DMR2:42396101	2	42396101	100	1	2.36E-07	0.195	0	0		
DMR2:44778301	2	44778301	300	1	4.61E-07	0.534	0	0	Skiv2l2;Dhx29	Transcription
DMR2:47485401	2	47485401	1100	1	9.47E-07	0.43	8	0.7273		
DMR2:48424601	2	48424601	700	1	6.47E-07	0.341	6	0.8571		
DMR2:51955201	2	51955201	300	2	1.12E-08	0.509	1	0.3333		
DMR2:52275101	2	52275101	700	1	8.54E-07	0.642	0	0	Nnt	Metabolism
DMR2:52340801	2	52340801	200	1	3.16E-08	0.28	2	1	RGD1306227	
DMR2:55009001	2	55009001	400	1	8.43E-08	0.501	3	0.75		
DMR2:67896501	2	67896501	200	1	2.59E-07	0.223	1	0.5		
DMR2:82503101	2	82503101	300	1	6.50E-07	0.49	2	0.6667	AABR07008991.1	
DMR2:83309601	2	83309601	300	1	1.02E-07	0.639	1	0.3333		
DMR2:83312901	2	83312901	300	1	3.95E-07	0.302	1	0.3333		
DMR2:93735001	2	93735001	300	1	8.12E-10	0.585	2	0.6667	Fabp12	Binding Protein
DMR2:102984201	2	102984201	400	2	1.07E-08	0.574	9	2.25		
DMR2:106884801	2	106884801	300	1	9.72E-07	0.359	0	0		
DMR2:110307101	2	110307101	400	1	4.61E-10	0.562	4	1	LOC499584	
DMR2:110836501	2	110836501	200	1	9.09E-07	0.482	0	0		
DMR2:111807901	2	111807901	400	1	8.28E-09	0.485	3	0.75		
DMR2:112504601	2	112504601	500	1	1.05E-07	0.552	0	0	Spata16	
DMR2:113199701	2	113199701	400	1	1.19E-10	0.468	0	0	Fndc3b	Cytoskeleton
DMR2:114528601	2	114528601	1500	1	2.96E-07	0.577	20	1.3333		
DMR2:114666301	2	114666301	500	1	4.19E-07	13.442	10	2		
DMR2:114752101	2	114752101	500	1	7.33E-07	6.209	5	1		
DMR2:115810301	2	115810301	100	1	3.31E-11	0	0	0	U1	
DMR2:122945101	2	122945101	400	1	6.88E-07	0.599	0	0	Qrfpr	Receptor
DMR2:141307501	2	141307501	700	2	6.92E-07	0.647	1	0.1429		
DMR2:141538601	2	141538601	600	1	9.46E-07	0.6	4	0.6667		
DMR2:143598501	2	143598501	300	1	1.54E-07	0.551	1	0.3333	Trpc4	Development
DMR2:149577301	2	149577301	600	1	4.37E-07	0.623	0	0		
DMR2:153250801	2	153250801	500	1	7.29E-07	0.254	5	1		
DMR2:156214301	2	156214301	900	1	1.95E-07	2.412	2	0.2222		
DMR2:158692301	2	158692301	400	1	1.85E-07	0.574	8	2		
DMR2:159112701	2	159112701	5600	1	1.19E-08	6.043	118	2.1071		
DMR2:160726901	2	160726901	500	2	2.35E-09	2.351	4	0.8		
DMR2:160729501	2	160729501	700	5	9.86E-10	2.626	6	0.8571		
DMR2:161037401	2	161037401	1900	2	3.97E-07	2.227	27	1.4211		
DMR2:168814701	2	168814701	300	2	1.17E-09	0.539	0	0		
DMR2:174066201	2	174066201	1500	1	3.35E-07	3.871	11	0.7333	Serpini1	Proteolysis
DMR2:175252001	2	175252001	400	1	6.52E-07	3.251	4	1		
DMR2:178451001	2	178451001	300	1	4.35E-10	0.347	0	0	Rxfp1	Receptor
DMR2:180317701	2	180317701	1300	2	2.05E-08	0.498	5	0.3846		
DMR2:183585401	2	183585401	800	1	7.68E-07	0.481	1	0.125	Arfip1	Signaling

DMR2:188766201	2	188766201	200	1	1.14E-07	0.305	1	0.5	Shc1;Pygo2;AC098750.1;Pbxip1	Signaling;Epigenetic
DMR2:193572101	2	193572101	1200	1	1.79E-08	0.571	68	5.6667	AABR07012329.1	
DMR2:194122701	2	194122701	1800	2	8.89E-09	0.576	51	2.8333		
DMR2:198844301	2	198844301	300	1	3.58E-07	0.4	5	1.6667	Pias3;Nudt17;Polr3c;SNORA5;Rnf115	Metabolism;Transcription
DMR2:199044701	2	199044701	900	5	1.69E-22	0.563	0	0	Gpr89b;Gja8	Signaling
DMR2:199069101	2	199069101	300	2	6.98E-09	0.313	2	0.6667		
DMR2:199096601	2	199096601	400	2	1.31E-07	0.394	13	3.25		
DMR2:199375201	2	199375201	400	1	1.12E-07	0.452	0	0		
DMR2:204717801	2	204717801	1500	1	9.16E-10	0.592	12	0.8		
DMR2:204924601	2	204924601	400	1	5.08E-07	0.625	2	0.5	Ngf	Signaling
DMR2:205073101	2	205073101	500	1	1.96E-07	0.56	1	0.2		
DMR2:205322301	2	205322301	100	1	2.81E-07	0.233	1	1	Sycp1;SNORA68	Cell Cycle
DMR2:206786001	2	206786001	400	1	9.90E-08	0.344	7	1.75	AABR07012762.1	
DMR2:208451501	2	208451501	200	1	4.50E-07	0.248	2	1	Tmigd3;LOC100911453;Wdr77	Epigenetic
DMR2:208620701	2	208620701	200	1	4.55E-07	0.22	4	2	Pifo	
DMR2:212654501	2	212654501	300	1	4.22E-08	0.421	0	0		
DMR2:216642901	2	216642901	800	1	8.18E-09	0.546	4	0.5		
DMR2:219875001	2	219875001	300	1	4.83E-07	0.522	0	0		
DMR2:226853001	2	226853001	500	2	9.19E-09	0.356	0	0		
DMR2:236664101	2	236664101	300	1	3.22E-08	0.308	3	1	Papss1	Metabolism
DMR2:239023001	2	239023001	300	1	8.28E-09	0.507	0	0		
DMR2:243356701	2	243356701	1000	1	6.99E-07	0.497	14	1.4	Mttp	Transport
DMR2:244095901	2	244095901	1300	1	9.70E-09	0.55	17	1.3077	Tspan5	Cytoskeleton
DMR2:244122501	2	244122501	500	1	5.39E-07	0.434	1	0.2	Tspan5	Cytoskeleton
DMR2:245726601	2	245726601	500	1	1.99E-07	0.462	0	0		
DMR2:253311201	2	253311201	800	1	7.46E-10	0.581	2	0.25		
DMR2:254331001	2	254331001	800	1	2.68E-07	0.311	7	0.875	Rn60_2_2544.1	
DMR2:254656001	2	254656001	200	1	2.58E-07	0.267	4	2		
DMR2:261369301	2	261369301	1900	1	5.71E-07	0.295	58	3.0526		
DMR2:262096401	2	262096401	200	1	9.02E-07	0.429	3	1.5		
DMR3:2305401	3	2305401	300	2	1.14E-09	0.618	1	0.3333	Entpd8;Noxa1	Signaling;Metabolism
DMR3:2901201	3	2901201	1100	1	1.55E-08	0.512	9	0.8182	Lcn6;Lcn10;Obp2a	Binding Protein
DMR3:5579901	3	5579901	300	1	5.40E-07	0.318	7	2.3333	Cacfd1;Slc2a6;AC126134.1	Transport
DMR3:5934901	3	5934901	1300	1	1.23E-07	0.547	22	1.6923	Vav2	Signaling
DMR3:9995601	3	9995601	500	1	3.59E-07	0.495	3	0.6	Fibcd1	Cytoskeleton
DMR3:10302701	3	10302701	500	1	4.14E-07	0.622	0	0	Rn60_3_0103.2	
DMR3:10505001	3	10505001	300	2	2.80E-09	0.39	2	0.6667		
DMR3:12654801	3	12654801	1200	1	4.23E-07	0.513	15	1.25	Lmx1b	
DMR3:13151001	3	13151001	400	1	8.37E-07	0.412	1	0.25		
DMR3:14959301	3	14959301	1100	1	8.69E-08	0.155	15	1.3636	Dab2ip	Signaling
DMR3:18300401	3	18300401	300	1	1.46E-08	0.303	3	1		
DMR3:23694201	3	23694201	200	2	1.05E-13	0.068	2	1		
DMR3:24674901	3	24674901	300	2	1.51E-08	0.453	2	0.6667		
DMR3:26885301	3	26885301	800	1	5.28E-08	0.558	5	0.625		
DMR3:30175001	3	30175001	200	1	1.38E-09	0.565	0	0		
DMR3:36093501	3	36093501	300	1	4.97E-09	0.366	5	1.6667		
DMR3:43814301	3	43814301	2100	1	9.53E-08	4.444	15	0.7143		
DMR3:45291601	3	45291601	300	1	9.75E-07	0.287	3	1	Pkp4	Cytoskeleton
DMR3:45446701	3	45446701	400	1	4.32E-07	0.436	3	0.75		
DMR3:47573401	3	47573401	1200	1	9.73E-08	0.406	3	0.25	AABR07052324.1;Psmd14	Protease
DMR3:48607901	3	48607901	400	1	7.51E-07	0.244	2	0.5	Ifih1	Transcription
DMR3:50156801	3	50156801	600	1	3.17E-07	0.439	2	0.3333		
DMR3:50865001	3	50865001	200	2	2.76E-07	0.437	1	0.5	AABR07052390.1	
DMR3:52065701	3	52065701	1100	1	1.04E-07	0.621	20	1.8182	Csrnp3	Unknown
DMR3:52577101	3	52577101	200	1	8.53E-08	0.521	0	0	Scn9a	Transport
DMR3:55077801	3	55077801	300	1	6.70E-09	0.568	1	0.3333		

DMR3:55603201	3	55603201	300	1	9.55E-08	0.361	1	0.3333		
DMR3:58897001	3	58897001	200	1	2.34E-07	0.35	8	4	Rapgef4	Development
DMR3:60962401	3	60962401	200	1	7.87E-07	8.291	3	1.5		
DMR3:73166501	3	73166501	300	1	2.30E-07	0.427	0	0	Olr458	
DMR3:76468901	3	76468901	300	1	3.28E-07	0.297	6	2	Olr619	
DMR3:80303701	3	80303701	400	3	8.78E-13	0.477	1	0.25	RGD1309540	Unknown
DMR3:80939101	3	80939101	400	1	3.59E-07	2.046	3	0.75	Creb3l1	Transcription
DMR3:81016901	3	81016901	400	1	5.43E-07	0.397	0	0		
DMR3:83948901	3	83948901	400	1	1.90E-08	0.536	0	0		
DMR3:84315001	3	84315001	500	1	2.00E-08	0.592	2	0.4		
DMR3:93122801	3	93122801	800	1	2.28E-07	12.87	6	0.75		
DMR3:95233501	3	95233501	200	1	1.80E-07	0.299	0	0	LOC691083	
DMR3:96883601	3	96883601	900	1	3.53E-07	0.442	8	0.8889		
DMR3:107856801	3	107856801	500	1	1.58E-07	0.458	0	0		
DMR3:109503701	3	109503701	700	1	1.40E-07	0.467	3	0.4286		
DMR3:111152601	3	111152601	700	1	1.50E-08	0.595	0	0	Dll4;Chac1	Growth Factors & Cytokines;Metabolism
DMR3:113726601	3	113726601	500	2	2.44E-11	0.563	0	0		
DMR3:116960001	3	116960001	600	1	7.21E-07	0.458	30	5	Sema6d	Growth Factors & Cytokines
DMR3:118675601	3	118675601	400	2	9.76E-12	0.368	8	2	Atp8b4	Transport
DMR3:121118401	3	121118401	300	1	1.35E-08	0.473	0	0	LOC499886	Unknown
DMR3:121323801	3	121323801	2000	1	2.92E-08	0.411	19	0.95	Mertk;SNORA25	Signaling
DMR3:122258101	3	122258101	400	1	1.88E-09	0.373	0	0		
DMR3:123634301	3	123634301	700	1	6.66E-08	0.517	8	1.1429	Siglec1;U6	Extracellular Matrix
DMR3:124844901	3	124844901	1200	2	6.88E-09	0.41	7	0.5833		
DMR3:125237201	3	125237201	500	1	1.99E-07	0.396	2	0.4	AABR07053771.1	
DMR3:125309901	3	125309901	400	2	3.23E-07	0.499	0	0		
DMR3:130687701	3	130687701	400	2	1.41E-09	0.499	1	0.25		
DMR3:138610801	3	138610801	200	2	1.44E-09	0.589	0	0	Zfp133	Transcription
DMR3:138886101	3	138886101	300	1	1.42E-07	0.487	3	1	Dtd1	Transcription
DMR3:139867001	3	139867001	400	1	1.94E-09	0.256	5	1.25		
DMR3:147099101	3	147099101	600	2	1.59E-08	0.54	0	0	Sdcbp2;Snph	Transport
DMR3:149277701	3	149277701	300	1	2.72E-07	0.207	5	1.6667	Efcab8	
DMR3:150169601	3	150169601	300	1	3.04E-08	0.585	8	2.6667		
DMR3:151812501	3	151812501	300	1	8.24E-08	0.391	0	0		
DMR3:159097301	3	159097301	500	3	4.47E-10	0.362	10	2	LOC103694889	
DMR3:160572301	3	160572301	800	2	2.50E-08	0.593	6	0.75	Wfdc5;AABR07054562.2;Wfdc12	Signaling
DMR3:160662101	3	160662101	600	2	4.54E-08	0.387	13	2.1667	LOC103691965	
DMR3:160893701	3	160893701	400	1	8.75E-07	0.512	0	0	Sdc4	Cytoskeleton
DMR3:162241101	3	162241101	1300	4	3.14E-13	0.551	1	0.0769	AABR07054583.1	
DMR3:166220501	3	166220501	1100	1	7.29E-07	0.529	2	0.1818		
DMR3:166481401	3	166481401	500	1	5.05E-07	0.508	2	0.4		
DMR3:169443601	3	169443601	700	1	3.33E-07	3.016	4	0.5714		
DMR3:169676001	3	169676001	500	1	5.25E-07	0.478	2	0.4		
DMR3:170092201	3	170092201	1600	1	7.82E-07	0.394	23	1.4375		
DMR3:170429801	3	170429801	1700	2	4.69E-09	0.57	39	2.2941	Cass4	Transcription
DMR3:171717801	3	171717801	1100	1	5.57E-07	0.385	5	0.4545		
DMR3:176733501	3	176733501	400	1	2.29E-07	0.313	14	3.5	Fndc11;Helz2	Transcription
DMR3:177220601	3	177220601	1300	1	1.52E-08	0.348	4	0.3077	Oprl1	Receptor
DMR4:798601	4	798601	600	4	5.16E-10	0.442	2	0.3333		
DMR4:6337301	4	6337301	500	2	3.23E-08	0.683	8	1.6	Galnt11;E130116L18Rik;Galntl5	Metabolism
DMR4:6487901	4	6487901	300	1	6.46E-07	0.325	5	1.6667	Rn60_4_0065.1	
DMR4:16066901	4	16066901	2000	1	7.08E-07	0.493	31	1.55	Cacna2d1	Metabolism
DMR4:21657401	4	21657401	300	2	1.63E-09	0.321	0	0	RGD1563349	EST
DMR4:25821601	4	25821601	300	1	5.32E-08	0.614	0	0	Cdk14	Cell Cycle
DMR4:28043101	4	28043101	700	2	3.85E-09	0.478	5	0.7143		

DMR4:28923301	4	28923301	200	1	5.71E-07	0.286	4	2		
DMR4:29086501	4	29086501	500	2	4.28E-10	0.539	4	0.8	Bet1	Golgi
DMR4:29447201	4	29447201	2000	1	4.57E-07	0.481	13	0.65		
DMR4:29649001	4	29649001	1500	7	8.00E-11	0.683	8	0.5333	Casd1	
DMR4:29653201	4	29653201	2300	7	2.85E-10	0.689	11	0.4783	Casd1	
DMR4:31715801	4	31715801	1700	1	1.81E-07	0.606	20	1.1765	Slc25a13	Metabolism
DMR4:39090801	4	39090801	300	2	3.95E-07	0.532	2	0.6667	Thsd7a;5S_rRNA	Extracellular Matrix
DMR4:41101901	4	41101901	700	2	1.12E-10	0.584	4	0.5714		
DMR4:41492101	4	41492101	500	1	6.89E-07	0.639	1	0.2	Foxp2	Transcription
DMR4:41669101	4	41669101	600	1	7.71E-08	0.535	1	0.1667	Foxp2	Transcription
DMR4:42693101	4	42693101	400	1	6.99E-07	0.377	13	3.25	Cftr	Transport
DMR4:45007101	4	45007101	1300	1	1.78E-07	0.381	6	0.4615	ST7	Unknown
DMR4:54827001	4	54827001	1900	1	4.36E-07	5.55	38	2	Grm8	Receptor
DMR4:54923501	4	54923501	500	2	5.94E-12	0.537	0	0	Grm8	Receptor
DMR4:55760101	4	55760101	1600	1	1.87E-07	0.379	8	0.5		
DMR4:56664901	4	56664901	200	1	1.13E-11	0.607	0	0	Opn1sw;Ccdc136	Receptor
DMR4:57908601	4	57908601	1900	1	5.38E-07	0.44	38	2	Cpa4	Proteolysis
DMR4:59070001	4	59070001	500	1	8.75E-07	0.559	1	0.2		
DMR4:63775201	4	63775201	300	1	6.65E-07	0.374	1	0.3333		
DMR4:70613401	4	70613401	400	1	2.62E-07	0.614	1	0.25	AC142181.1;Prss3	Proteolysis
DMR4:76022201	4	76022201	400	1	7.11E-08	0.428	3	0.75	Cntnap2	Receptor
DMR4:77399901	4	77399901	12400	14	1.01E-11	0.596	376	3.0323	AABR07060519.1	
DMR4:77413601	4	77413601	2900	1	1.17E-07	0.689	52	1.7931	AABR07060519.1	
DMR4:77447001	4	77447001	6300	2	6.09E-09	0.644	212	3.3651	AABR07060519.1;Y_RNA	
DMR4:78304801	4	78304801	1000	2	3.51E-09	0.637	5	0.5	Gimap8;Gimap9	Unknown
DMR4:78375301	4	78375301	700	2	1.35E-07	0.603	6	0.8571	Gimap1;Gimap5	Unknown
DMR4:83959001	4	83959001	500	1	3.07E-08	3.186	1	0.2	Tril	Receptor
DMR4:84523901	4	84523901	400	1	3.34E-07	0.552	0	0		
DMR4:95545901	4	95545901	500	2	2.32E-07	0.561	1	0.2		
DMR4:98899001	4	98899001	400	1	4.64E-07	0.489	2	0.5		
DMR4:99046901	4	99046901	400	1	4.09E-07	0.537	0	0		
DMR4:99812501	4	99812501	400	1	2.62E-07	0.414	0	0	Ptcd3	Unknown
DMR4:100356701	4	100356701	500	1	1.70E-07	0.51	0	0		
DMR4:105520501	4	105520501	2700	1	6.97E-07	4.259	16	0.5926		
DMR4:106904301	4	106904301	700	1	1.18E-08	5.752	1	0.1429		
DMR4:110176401	4	110176401	300	1	5.66E-07	28.043	0	0		
DMR4:112617501	4	112617501	200	1	3.89E-07	0.215	1	0.5		
DMR4:116072401	4	116072401	600	1	4.08E-07	0.401	5	0.8333		
DMR4:117678701	4	117678701	1200	1	4.66E-10	0.295	42	3.5	Figla	Transcription
DMR4:119307701	4	119307701	200	1	1.44E-07	0.608	0	0	Arhgap25	Unknown
DMR4:119456501	4	119456501	200	1	2.99E-07	0.525	1	0.5	Vom1r102	
DMR4:119526901	4	119526901	300	1	7.98E-07	0.522	5	1.6667	Aplf	
DMR4:121333701	4	121333701	1000	1	2.26E-07	0.46	9	0.9	Chchd6	Transcription
DMR4:121622301	4	121622301	700	1	1.90E-07	0.424	7	1	Txnrd3	Metabolism
DMR4:122649001	4	122649001	2400	1	1.89E-07	0.616	10	0.4167	Nup210	Cytoskeleton
DMR4:123261801	4	123261801	400	1	7.99E-07	0.503	5	1.25	SNORA17	
DMR4:123751401	4	123751401	1000	1	7.65E-07	0.438	10	1	Ccdc174	
DMR4:124617201	4	124617201	500	1	5.53E-07	0.496	1	0.2		
DMR4:125118701	4	125118701	200	1	6.80E-07	0.559	0	0		
DMR4:125801401	4	125801401	1200	2	9.50E-12	0.596	11	0.9167	Magi1	
DMR4:132506101	4	132506101	200	1	2.07E-07	0.554	1	0.5		
DMR4:132783301	4	132783301	1200	1	2.22E-07	0.386	13	1.0833		
DMR4:132935101	4	132935101	2300	6	2.41E-11	0.62	25	1.087		
DMR4:132968101	4	132968101	500	1	8.03E-07	0.575	0	0		
DMR4:133393601	4	133393601	600	1	7.18E-07	0.497	0	0		
DMR4:145985301	4	145985301	300	1	1.50E-08	0.502	0	0	Atp2b2	Metabolism
DMR4:146258501	4	146258501	500	2	1.19E-09	0.308	15	3		
DMR4:146538701	4	146538701	10100	2	2.22E-07	0.621	295	2.9208		
DMR4:148156101	4	148156101	600	2	1.42E-07	0.457	4	0.6667	Washc2c	

DMR4:149384301	4	149384301	200	1	9.53E-08	0.473	0	0	AABR07061848.1	
DMR4:149847601	4	149847601	200	1	7.65E-07	0.439	0	0		
DMR4:149849201	4	149849201	900	1	2.09E-08	0.549	10	1.1111		
DMR4:155617101	4	155617101	900	1	1.47E-07	0.397	13	1.4444	Slc2a3	Transport
DMR4:157106001	4	157106001	200	1	2.23E-07	0.236	7	3.5	C1rl	Protease
DMR4:157157101	4	157157101	700	1	1.25E-07	0.28	5	0.7143	C1s	Immune
DMR4:158928301	4	158928301	800	1	3.58E-08	0.367	6	0.75		
DMR4:160444901	4	160444901	700	1	9.93E-07	0.501	8	1.1429		
DMR4:162231801	4	162231801	300	1	4.51E-07	0.419	2	0.6667	LOC689757	Development
DMR4:169807501	4	169807501	1200	1	4.77E-08	0.506	21	1.75	Grin2b	Receptor
DMR4:170965501	4	170965501	400	1	7.94E-07	0.521	2	0.5	Pde6h	Metabolism
DMR4:171684301	4	171684301	500	1	7.31E-07	0.586	0	0		
DMR4:172140201	4	172140201	400	1	2.48E-07	0.52	0	0	Mgst1	Metabolism
DMR4:173488701	4	173488701	200	2	2.69E-17	0.081	5	2.5		
DMR4:173656101	4	173656101	300	1	1.48E-08	0.339	1	0.3333		
DMR4:173671101	4	173671101	900	1	9.15E-07	0.594	0	0		
DMR4:175647901	4	175647901	1200	1	6.94E-07	0.442	6	0.5	Pde3a	Signaling
DMR4:176637401	4	176637401	300	1	3.70E-07	0.413	0	0	Spx;Gys2	Metabolism
DMR4:177617801	4	177617801	400	1	7.93E-11	0.435	3	0.75		
DMR4:178003401	4	178003401	600	1	5.70E-07	3.114	6	1		
DMR4:179166901	4	179166901	300	1	7.12E-07	0.406	0	0		
DMR4:179635001	4	179635001	1600	1	1.40E-09	0.555	27	1.6875		
DMR4:179683201	4	179683201	1000	1	5.45E-07	0.437	17	1.7	Lmntd1	
DMR4:181925901	4	181925901	300	2	1.56E-07	0.547	2	0.6667	Ccdc91	Unknown
DMR4:182173101	4	182173101	200	1	7.88E-07	0.536	0	0		
DMR4:183093201	4	183093201	1200	2	1.03E-07	0.414	16	1.3333		
DMR5:4747501	5	4747501	500	1	9.37E-10	0.649	2	0.4	Rbpj	Transcription
DMR5:4789301	5	4789301	900	1	4.50E-07	4.542	6	0.6667	Rbpj	Transcription
DMR5:9061601	5	9061601	200	1	5.81E-07	0.397	4	2	Sgk3	Signaling
DMR5:9465901	5	9465901	400	1	7.31E-07	3.331	1	0.25		
DMR5:16714301	5	16714301	1100	8	2.28E-11	0.577	1	0.0909	Rps20;snoU54	Translation
DMR5:16880601	5	16880601	300	1	1.79E-08	0.616	0	0		
DMR5:18889301	5	18889301	200	1	6.56E-07	0.423	0	0		
DMR5:19536401	5	19536401	400	1	1.49E-07	0.348	9	2.25	Nsmaf	Signaling
DMR5:22790801	5	22790801	700	1	2.35E-08	0.591	3	0.4286	Asph	
DMR5:24350201	5	24350201	400	1	9.29E-07	0.327	5	1.25		
DMR5:37475901	5	37475901	400	1	3.66E-07	0.438	4	1		
DMR5:58462201	5	58462201	600	4	1.06E-14	0.447	0	0	Fancg;Pigo;Stoml2	Metabolism;Cytoskeleton
DMR5:60992101	5	60992101	700	1	4.43E-08	0.152	15	2.1429	Shb	
DMR5:61094701	5	61094701	2100	2	3.28E-09	0.507	25	1.1905		
DMR5:64470201	5	64470201	1600	3	2.45E-08	0.625	8	0.5	LOC108348074	
DMR5:78748501	5	78748501	1700	1	5.80E-07	0.468	17	1		
DMR5:81162901	5	81162901	300	1	8.97E-08	0.379	4	1.3333		
DMR5:91125501	5	91125501	12400	4	3.58E-09	0.595	1489	12.0081	pRNA;5_8S_rRNA;AABR07048791.1	
DMR5:99920301	5	99920301	1300	1	1.48E-07	0.62	9	0.6923	U4	
DMR5:100389801	5	100389801	500	1	4.83E-07	0.554	0	0		
DMR5:102239701	5	102239701	400	1	3.55E-07	0.604	3	0.75		
DMR5:103378601	5	103378601	1400	1	3.03E-08	0.533	22	1.5714		
DMR5:121133301	5	121133301	2800	1	8.05E-07	0.614	68	2.4286		
DMR5:124965301	5	124965301	100	1	3.12E-07	0.163	2	2		
DMR5:126185601	5	126185601	800	1	1.41E-07	0.154	17	2.125	Dhcr24;Lexm	Metabolism
DMR5:127601001	5	127601001	300	1	3.92E-08	0.32	2	0.6667	Slc1a7	Transport
DMR5:128332201	5	128332201	500	1	6.61E-08	0.39	0	0	Zfyve9	Transcription
DMR5:129908001	5	129908001	200	2	5.66E-10	0.545	1	0.5		
DMR5:133240801	5	133240801	500	2	9.35E-13	0.579	7	1.4	Trabd2b	
DMR5:134457801	5	134457801	500	1	2.69E-08	0.529	4	0.8		
DMR5:138190901	5	138190901	600	2	3.44E-07	0.486	3	0.5	Slc2a1	Metabolism

DMR5:138430101	5	138430101	300	2	6.36E-08	0.545	1	0.3333	Ccdc30	
DMR5:138699301	5	138699301	1700	2	6.60E-11	0.299	1	0.0588	Guca2b	Signaling
DMR5:139408901	5	139408901	1400	1	8.27E-07	3.411	11	0.7857	Scmh1	Epigenetic
DMR5:139518401	5	139518401	1800	1	1.34E-08	0.508	14	0.7778		
DMR5:144296901	5	144296901	600	4	1.18E-12	0.594	0	0	Trappc3	
DMR5:145344601	5	145344601	1300	1	2.21E-07	0.536	13	1	U6	
DMR5:145822001	5	145822001	500	1	4.59E-07	0.357	0	0		
DMR5:146403101	5	146403101	4800	23	2.79E-15	0.636	28	0.5833	Csmd2	Unknown
DMR5:149733201	5	149733201	2000	2	1.13E-08	0.522	35	1.75		
DMR5:151724301	5	151724301	1000	2	2.21E-08	0.635	0	0		
DMR5:152643201	5	152643201	1400	1	3.70E-07	0.541	8	0.5714	Pafah2	Metabolism
DMR5:152821601	5	152821601	200	1	7.56E-07	0.229	5	2.5	Man1c1	Metabolism
DMR5:153245401	5	153245401	500	1	8.77E-07	0.483	2	0.4	Tmem50a	
DMR5:154051901	5	154051901	400	1	3.70E-07	2.025	0	0	Ifnlr1	Receptor
DMR5:154184401	5	154184401	1900	1	1.04E-09	0.299	25	1.3158		
DMR5:155794701	5	155794701	1000	2	1.71E-08	0.578	1	0.1	LOC690206	Unknown
DMR5:156825501	5	156825501	300	1	8.32E-07	0.507	0	0		
DMR5:158656101	5	158656101	400	1	4.00E-07	0.603	0	0	AABR07050265.1	
DMR5:160491001	5	160491001	1100	2	3.95E-09	0.643	13	1.1818	Fhad1	
DMR5:160945901	5	160945901	700	1	2.35E-08	0.568	0	0		
DMR5:162823001	5	162823001	500	1	4.58E-07	0.357	2	0.4	Dhrs3	Metabolism
DMR5:165526301	5	165526301	800	2	2.15E-09	0.615	2	0.25		
DMR5:165581401	5	165581401	1700	1	1.82E-07	0.555	13	0.7647		
DMR5:165584401	5	165584401	900	2	5.94E-12	0.539	14	1.5556		
DMR5:165645701	5	165645701	2200	1	1.22E-09	0.47	28	1.2727		
DMR5:167424001	5	167424001	200	1	6.05E-07	0.322	1	0.5	Rere	Unknown
DMR5:167453601	5	167453601	300	1	8.41E-07	0.711	0	0	Rere	Unknown
DMR5:168387201	5	168387201	600	1	1.78E-07	8.075	3	0.5	Camta1	Transcription
DMR5:168936401	5	168936401	200	1	1.08E-07	0.499	0	0	Camta1	Transcription
DMR5:169041101	5	169041101	300	1	8.60E-08	0.362	2	0.6667	Rn60_5_1691.2	
DMR5:169788001	5	169788001	400	1	3.30E-07	0.527	0	0		
DMR5:170514901	5	170514901	500	2	3.96E-09	0.449	1	0.2		
DMR5:171079901	5	171079901	400	2	5.46E-09	0.512	0	0		
DMR5:171344401	5	171344401	600	1	4.43E-07	0.514	27	4.5		
DMR5:173227301	5	173227301	300	1	7.24E-08	0.581	0	0	Vwa1;Tmem88b	Extracellular Matrix;Unknown
DMR6:1025101	6	1025101	1400	1	5.17E-07	0.658	10	0.7143		
DMR6:5747701	6	5747701	400	1	5.05E-08	0.42	0	0		
DMR6:8305001	6	8305001	300	1	8.25E-09	0.53	1	0.3333	Slc3a1	Metabolism
DMR6:9620001	6	9620001	2000	1	2.44E-07	0.619	11	0.55	AABR07062800.1	
DMR6:9687501	6	9687501	500	1	7.51E-07	4.562	11	2.2		
DMR6:10481401	6	10481401	500	1	3.08E-10	0.557	8	1.6	Tmem247	
DMR6:10513501	6	10513501	1400	1	8.51E-08	0.565	15	1.0714	Atp6v1e2	Transport
DMR6:21304501	6	21304501	500	1	7.67E-07	0.51	1	0.2	Ltbp1	Metabolism
DMR6:27291001	6	27291001	300	1	7.48E-09	0.352	3	1	Cib4	Signaling
DMR6:28330501	6	28330501	500	2	3.17E-11	0.529	5	1	Dnmt3a	Transcription
DMR6:29281601	6	29281601	200	1	7.86E-07	0.49	0	0	Klhl29	Transcription
DMR6:30117101	6	30117101	400	1	7.04E-07	0.526	9	2.25	Itsn2	EST
DMR6:30628001	6	30628001	5800	2	3.76E-10	0.55	727	12.5345	5_8S_rRNA;AABR07063421.1;AABR07063424.1;LOC257642;AABR07063425.2;AABR07063425.1	
DMR6:31048801	6	31048801	3500	2	2.10E-07	0.434	197	5.6286	AABR07063462.1	
DMR6:31062801	6	31062801	7100	1	6.96E-07	0.704	135	1.9014		
DMR6:34163601	6	34163601	500	2	7.36E-08	0.439	6	1.2	Ttc32	
DMR6:38470301	6	38470301	600	1	4.86E-11	0.475	0	0	Nbas	Unknown
DMR6:41640801	6	41640801	900	2	3.48E-08	0.428	23	2.5556		
DMR6:44300401	6	44300401	500	1	3.36E-07	0.622	13	2.6	Kidins220	
DMR6:47511401	6	47511401	700	1	4.36E-07	5.853	5	0.7143		

DMR6:49144801	6	49144801	300	1	7.03E-07	0.535	0	0	Sntg2	Cytoskeleton
DMR6:49557001	6	49557001	600	1	3.32E-07	0.433	0	0		
DMR6:55340901	6	55340901	600	2	3.49E-08	0.446	4	0.6667		
DMR6:62044801	6	62044801	400	2	1.17E-09	0.521	2	0.5		
DMR6:63331201	6	63331201	1000	3	2.29E-10	0.567	0	0		
DMR6:75718501	6	75718501	2300	1	4.29E-09	0.367	31	1.3478		
DMR6:77548201	6	77548201	900	1	2.39E-07	0.394	24	2.6667		
DMR6:94239701	6	94239701	400	2	2.13E-07	0.661	1	0.25		
DMR6:94852401	6	94852401	3100	1	5.71E-07	0.299	40	1.2903	Jkamp;Ccdc175	Receptor
DMR6:95020101	6	95020101	300	1	6.10E-07	0.701	1	0.3333	Rtn1	Metabolism
DMR6:97274301	6	97274301	400	1	3.80E-09	0.304	2	0.5		
DMR6:99857601	6	99857601	500	1	7.11E-07	0.644	7	1.4	Rab15	Signaling
DMR6:105784801	6	105784801	400	1	1.04E-07	0.335	5	1.25		
DMR6:106942401	6	106942401	300	1	1.59E-07	0.377	3	1	Dpf3	Transcription
DMR6:108587101	6	108587101	700	4	2.50E-09	0.59	1	0.1429	Ltbp2	Receptor
DMR6:115372301	6	115372301	300	1	2.96E-08	0.276	9	3		
DMR6:117183301	6	117183301	400	1	9.55E-07	0.42	2	0.5		
DMR6:122854201	6	122854201	500	2	1.76E-08	0.652	1	0.2	Eml5	Cytoskeleton
DMR6:122977901	6	122977901	300	1	6.28E-08	4.893	3	1	Ttc8	Metabolism
DMR6:125363601	6	125363601	400	1	6.41E-07	0.633	4	1		
DMR6:125756701	6	125756701	400	3	4.57E-10	0.574	3	0.75	Trip11	Receptor
DMR6:126421401	6	126421401	300	1	9.53E-10	0.576	0	0		
DMR6:128326801	6	128326801	500	2	1.70E-12	0.484	2	0.4		
DMR6:129385801	6	129385801	500	1	1.47E-07	0.439	3	0.6	AABR07065460.1	
DMR6:131082501	6	131082501	1100	3	2.49E-11	0.497	17	1.5455		
DMR6:131886101	6	131886101	200	1	2.68E-07	0.205	10	5	Bcl11b	Transcription
DMR6:132823101	6	132823101	1800	1	9.45E-07	0.519	47	2.6111	Wdr25	
DMR6:133002701	6	133002701	700	1	6.98E-07	0.606	7	1		
DMR6:134502801	6	134502801	2300	1	2.34E-07	0.605	61	2.6522		
DMR6:134608401	6	134608401	400	1	1.04E-08	0.528	0	0		
DMR6:137062601	6	137062601	800	1	8.66E-09	0.461	2	0.25	LOC691485	Unknown
DMR6:137124701	6	137124701	400	1	9.10E-07	0.292	2	0.5	AABR07065602.2	
DMR6:138198701	6	138198701	2400	2	1.11E-08	0.498	8	0.3333	Ighm	
DMR6:139664801	6	139664801	200	1	9.69E-07	0.483	0	0	AABR07065705.1	
DMR6:141673301	6	141673301	500	1	1.86E-08	0.559	0	0		
DMR6:142065001	6	142065001	300	1	5.40E-07	0.335	5	1.6667	AABR07065811.1	
DMR6:142772501	6	142772501	200	1	2.63E-07	0.394	1	0.5		
DMR6:143220801	6	143220801	800	2	8.01E-09	10.117	5	0.625	AABR07065844.1	
DMR6:144740401	6	144740401	200	1	1.95E-07	0.227	0	0	Ptprn2	Signaling
DMR6:145445601	6	145445601	200	2	6.73E-08	0.326	0	0		
DMR7:672201	7	672201	1900	1	4.08E-07	0.553	11	0.5789		
DMR7:11717201	7	11717201	400	1	4.45E-07	0.55	5	1.25	Sppl2b;Lsm7	Proteolysis;Translation
DMR7:12072201	7	12072201	1800	1	3.33E-07	0.612	15	0.8333	Atp8b3;Onecut3	Metabolism;Transcription
DMR7:12209401	7	12209401	700	1	6.93E-08	0.622	0	0	Plk5	Unknown
DMR7:12417201	7	12417201	400	1	6.99E-07	0.371	10	2.5	Midn;Atp5d	Development;Transport
DMR7:13768501	7	13768501	2900	2	9.81E-10	0.47	28	0.9655		
DMR7:17238001	7	17238001	100	1	8.47E-07	21.885	0	0		
DMR7:24628201	7	24628201	1100	2	2.29E-08	0.636	15	1.3636	Cry1;Mterf2	DNA Repair
DMR7:26691801	7	26691801	200	1	6.46E-07	0.433	1	0.5	Chst11;U6	Metabolism
DMR7:29732201	7	29732201	500	1	3.32E-09	0.53	2	0.4		
DMR7:49253201	7	49253201	600	1	1.48E-07	0.464	1	0.1667	Acss3;SNORA17	Metabolism
DMR7:53702501	7	53702501	1400	2	3.86E-07	0.539	14	1	Zdhhc17	Transcription
DMR7:54628301	7	54628301	1500	2	1.02E-09	0.472	18	1.2		
DMR7:61909501	7	61909501	200	1	3.86E-07	0.373	0	0		
DMR7:62088001	7	62088001	400	1	1.10E-07	0.581	8	2		
DMR7:63205901	7	63205901	1100	1	1.85E-08	0.67	12	1.0909		
DMR7:63289801	7	63289801	800	1	6.72E-08	0.502	6	0.75		
DMR7:66949901	7	66949901	2000	2	1.29E-07	0.635	26	1.3	Ppm1h	Signaling

DMR7:69988101	7	69988101	1400	1	8.61E-07	0.498	25	1.7857	RGD1564306	
DMR7:70941501	7	70941501	300	3	5.22E-17	0.532	4	1.3333	Stat6	Transcription
DMR7:71980201	7	71980201	700	1	3.98E-07	0.551	0	0	Cpq	Metabolism
DMR7:77116001	7	77116001	500	1	9.47E-10	0.433	0	0		
DMR7:77321401	7	77321401	1200	2	4.00E-08	0.592	2	0.1667		
DMR7:77626801	7	77626801	600	1	3.34E-08	0.387	5	0.8333		
DMR7:80703101	7	80703101	500	3	2.11E-10	0.581	4	0.8	Oxr1	Development
DMR7:92398601	7	92398601	700	1	3.37E-08	0.403	3	0.4286		
DMR7:92791001	7	92791001	1400	1	1.17E-07	0.383	16	1.1429	Ext1	Metabolism
DMR7:94796701	7	94796701	100	1	8.52E-07	0.207	1	1	Deptor	
DMR7:95096401	7	95096401	300	1	4.21E-07	0.404	4	1.3333	Col14a1	Cytoskeleton
DMR7:97894501	7	97894501	300	1	1.04E-07	0.531	1	0.3333	RGD1310852	Unknown
DMR7:98936001	7	98936001	300	1	1.48E-07	0.3	10	3.3333	Mtss1	Cytoskeleton
DMR7:99152501	7	99152501	400	1	3.86E-07	0.617	0	0	LOC108348266	
DMR7:100014801	7	100014801	1200	1	8.26E-08	0.588	4	0.3333		
DMR7:115180001	7	115180001	1800	1	6.44E-07	0.455	25	1.3889		
DMR7:119494001	7	119494001	1000	2	2.18E-10	0.321	6	0.6	Ncf4	Development
DMR7:120415501	7	120415501	300	2	3.50E-11	0.528	1	0.3333	AC096473.3	
DMR7:121002201	7	121002201	400	1	7.31E-09	0.478	1	0.25	Dnal4;Nptxr	Cytoskeleton
DMR7:121631101	7	121631101	200	2	2.54E-13	0.262	6	3		
DMR7:121944301	7	121944301	400	1	3.75E-07	0.479	1	0.25	Tnrc6b;Rpl26-ps1	Apoptosis
DMR7:122511101	7	122511101	300	1	4.95E-07	0.396	1	0.3333		
DMR7:122971801	7	122971801	300	1	9.37E-08	0.345	1	0.3333	Rangap1;Zc3h7b	Signaling;Transcription
DMR7:123483801	7	123483801	500	2	4.93E-09	0.546	0	0	Cenpm;LOC688613;LOC108351520	
DMR7:125536301	7	125536301	1200	1	5.29E-07	0.604	15	1.25		
DMR7:126211101	7	126211101	400	1	5.43E-07	0.438	1	0.25		
DMR7:126488001	7	126488001	500	1	4.45E-07	0.164	0	0		
DMR7:128013801	7	128013801	500	1	2.23E-07	0.584	15	3	AABR07058618.1	
DMR7:129382401	7	129382401	800	1	5.18E-08	0.48	1	0.125	LOC108351524	
DMR7:129412701	7	129412701	200	1	4.35E-07	0.3	0	0	LOC108351524	
DMR7:130184701	7	130184701	300	1	7.72E-09	0.471	2	0.6667	Dennd6b	
DMR7:131253401	7	131253401	2100	1	4.30E-08	0.293	22	1.0476		
DMR7:133000201	7	133000201	200	1	1.23E-07	0.329	1	0.5	Lrrk2	Unknown
DMR7:135299101	7	135299101	1400	1	5.73E-07	0.535	14	1		
DMR7:135855601	7	135855601	1100	1	4.12E-07	0.508	5	0.4545	Twf1	Cytoskeleton
DMR7:136860401	7	136860401	1300	1	8.84E-09	5.816	4	0.3077	Nell2	Signaling
DMR7:137257401	7	137257401	800	1	4.36E-07	0.202	19	2.375	Ano6	Signaling
DMR7:137961001	7	137961001	300	1	6.47E-07	0.334	6	2		
DMR7:138069501	7	138069501	1300	1	9.03E-10	0.312	11	0.8462	U6	
DMR7:140571301	7	140571301	3900	1	7.69E-07	0.63	5	0.1282	Dhh	Signaling
DMR7:142210001	7	142210001	1800	4	4.56E-13	0.369	43	2.3889	Pou6f1	Transcription
DMR7:142476301	7	142476301	1300	2	1.79E-09	0.638	22	1.6923		
DMR7:143246701	7	143246701	600	1	1.34E-07	0.407	0	0	Rn50_7_1411.3	
DMR8:8224501	8	8224501	300	1	6.37E-07	0.374	10	3.3333	Cntn5	Extracellular Matrix
DMR8:10626201	8	10626201	3500	1	5.07E-08	2.005	41	1.1714		
DMR8:11928201	8	11928201	400	2	2.58E-08	0.547	6	1.5	Ccdc82;Maml2	Transcription
DMR8:12109101	8	12109101	900	1	7.46E-07	0.587	4	0.4444	Maml2	Transcription
DMR8:14259101	8	14259101	600	3	1.10E-22	0.455	26	4.3333	Slc36a4	Transport
DMR8:22853601	8	22853601	500	1	7.34E-07	0.379	13	2.6	Dock6;Angptl8	Signaling
DMR8:23891701	8	23891701	600	1	5.68E-08	0.507	10	1.6667	Bbs9	Development
DMR8:27896101	8	27896101	300	1	1.19E-08	0.366	0	0	Glb1l3	Golgi
DMR8:28155201	8	28155201	600	2	1.66E-08	0.61	6	1	Jam3	Cytoskeleton
DMR8:31200101	8	31200101	500	2	3.89E-10	0.6	0	0		
DMR8:33562301	8	33562301	900	1	1.32E-08	0.594	2	0.2222	Fli1	Transcription
DMR8:36532101	8	36532101	1200	2	7.01E-10	0.384	16	1.3333		
DMR8:40010701	8	40010701	200	1	5.44E-07	0.17	4	2	Esam;Vsig2;Nrgn	Cytoskeleton;Immune;Signaling
DMR8:40062201	8	40062201	300	2	1.01E-08	0.314	1	0.3333	Spa17	Immune

DMR8:46838001	8	46838001	300	1	3.22E-08	0.581	1	0.3333	Grik4	Signaling
DMR8:46877601	8	46877601	300	1	9.75E-07	0.291	11	3.6667	Grik4	Signaling
DMR8:47208201	8	47208201	500	2	2.78E-08	0.445	3	0.6		
DMR8:47813601	8	47813601	400	1	2.08E-08	0.454	2	0.5		
DMR8:47828701	8	47828701	500	1	5.15E-07	0.485	0	0		
DMR8:48584501	8	48584501	2100	2	2.87E-08	0.425	60	2.8571	Ccdc153;Pdzd3;Nlrx1	Transcription
DMR8:48794901	8	48794901	1500	1	1.61E-08	0.283	0	0	U6;AC105645.4;Bcl9l	
DMR8:49141701	8	49141701	800	1	2.00E-07	0.483	20	2.5	Kmt2a	
DMR8:50360701	8	50360701	300	2	2.92E-11	0.443	1	0.3333	Sik3	Receptor
DMR8:50629801	8	50629801	600	1	3.64E-07	0.512	0	0		
DMR8:50892501	8	50892501	200	1	5.19E-08	0.642	1	0.5		
DMR8:51622401	8	51622401	300	1	8.22E-07	0.409	0	0		
DMR8:52210401	8	52210401	1400	1	6.00E-07	0.516	33	2.3571		
DMR8:52452001	8	52452001	500	1	7.00E-08	0.456	2	0.4		
DMR8:52951601	8	52951601	500	1	9.38E-07	0.405	0	0		
DMR8:59370401	8	59370401	400	1	9.07E-07	0.653	0	0		
DMR8:59755201	8	59755201	300	1	4.40E-07	0.561	1	0.3333	Ube2q2	Metabolism
DMR8:61176301	8	61176301	600	1	4.77E-07	0.619	0	0		
DMR8:61460801	8	61460801	400	2	2.11E-09	0.55	3	0.75		
DMR8:62782201	8	62782201	100	1	8.74E-07	0.451	0	0	Cyp11a1	Metabolism
DMR8:65351301	8	65351301	1400	1	9.67E-08	0.412	28	2	Rn50_8_0651.1	
DMR8:68018601	8	68018601	1100	1	6.93E-09	0.554	12	1.0909	U6	
DMR8:68284601	8	68284601	400	1	6.98E-09	0.405	0	0	Map2k5	Signaling
DMR8:73883001	8	73883001	600	1	1.80E-07	0.569	2	0.3333		
DMR8:74490001	8	74490001	200	1	1.71E-08	0.12	2	1		
DMR8:75922801	8	75922801	400	1	8.77E-07	0.485	6	1.5		
DMR8:77727601	8	77727601	300	2	2.72E-08	0.586	0	0	Aldh1a2	Metabolism
DMR8:84899301	8	84899301	300	1	6.98E-07	0.611	10	3.3333	AABR07070801.1;AABR07070802.1	
DMR8:87257001	8	87257001	300	1	2.69E-10	0.567	0	0	Filip1	Unknown
DMR8:88267801	8	88267801	600	1	1.02E-09	0.473	3	0.5		
DMR8:96286401	8	96286401	200	1	6.21E-08	0.232	1	0.5		
DMR8:97253301	8	97253301	900	1	3.43E-07	0.399	22	2.4444		
DMR8:98971501	8	98971501	200	1	6.49E-10	0.198	1	0.5		
DMR8:102378101	8	102378101	400	1	1.39E-07	0.423	2	0.5	Slc9a9	Transport
DMR8:103346901	8	103346901	300	1	3.24E-07	0.396	6	2	Paqr9;LOC501038	Receptor
DMR8:104407201	8	104407201	300	1	2.24E-08	0.412	1	0.3333	Rasa2	
DMR8:104595001	8	104595001	1700	1	5.58E-09	0.29	13	0.7647	Zbtb38	Transcription
DMR8:106367101	8	106367101	400	3	3.58E-08	0.58	0	0	Nmnat3	Metabolism
DMR8:107637901	8	107637901	200	2	1.74E-07	0.625	0	0	Mras	
DMR8:108762401	8	108762401	1700	1	1.49E-07	0.343	33	1.9412	Il20rb	Receptor
DMR8:112313301	8	112313301	800	1	2.18E-08	6.149	10	1.25		
DMR8:116495501	8	116495501	900	1	1.60E-07	0.272	24	2.6667	Rbm5	Translation
DMR8:119071001	8	119071001	400	1	9.59E-07	0.554	2	0.5	Prss42	Protease
DMR8:119745901	8	119745901	600	1	4.97E-07	0.254	0	0	Dclk3	Cytoskeleton
DMR8:121577001	8	121577001	400	1	1.40E-07	0.646	2	0.5	AABR07071549.1	
DMR8:121929401	8	121929401	300	1	7.57E-07	3.189	7	2.3333	Pdcd6ip	Apoptosis
DMR8:124971201	8	124971201	600	1	7.87E-07	9.055	6	1	Rbms3	Epigenetic
DMR8:126283201	8	126283201	200	1	1.49E-07	0.167	8	4		
DMR8:127026801	8	127026801	1000	1	3.74E-07	0.622	6	0.6		
DMR8:127473901	8	127473901	1100	1	1.95E-07	0.422	1	0.0909	Itga9	Extracellular Matrix
DMR8:127963501	8	127963501	400	1	4.38E-07	0.343	5	1.25	Oxsr1	Signaling
DMR8:128397201	8	128397201	300	1	7.75E-07	0.493	4	1.3333	Scn10a	Transport
DMR8:129589701	8	129589701	1200	1	3.85E-07	0.421	12	1		
DMR8:131357801	8	131357801	200	1	2.31E-07	0.199	2	1		
DMR8:131661801	8	131661801	200	1	2.42E-07	0.464	2	1		
DMR8:132563001	8	132563001	400	1	2.68E-07	0.516	0	0	Limd1	Transcription
DMR9:2002401	9	2002401	2600	1	9.27E-08	0.518	17	0.6538		
DMR9:2030101	9	2030101	1100	1	9.16E-07	0.4	8	0.7273		

DMR9:10507301	9	10507301	500	3	8.34E-19	0.488	2	0.4		
DMR9:10852401	9	10852401	200	1	6.73E-07	0.665	0	0	Dpp9	Proteolysis
DMR9:12833201	9	12833201	300	1	2.55E-07	0.351	0	0	Rftn1	Unknown
DMR9:13150201	9	13150201	200	1	1.89E-08	0.336	3	1.5	Kif6	Cytoskeleton
DMR9:13406001	9	13406001	700	2	3.59E-08	0.665	5	0.7143		
DMR9:13496601	9	13496601	1000	1	1.18E-07	0.424	20	2	Daam2;Mocs1	Cytoskeleton;Unknown
DMR9:14866501	9	14866501	800	2	3.37E-09	0.413	13	1.625	LOC680920	
DMR9:15211101	9	15211101	300	1	2.48E-08	0.282	5	1.6667	Tfeb	Transcription
DMR9:16621401	9	16621401	1100	3	2.03E-09	0.489	5	0.4545	Mea1;Klhdc3;Rrp36;LOC108348250	Unknown;Transcription
DMR9:17007601	9	17007601	2000	1	7.17E-07	0.565	24	1.2		
DMR9:19377801	9	19377801	300	1	2.60E-08	0.44	0	0	Clic5	Transport
DMR9:19823001	9	19823001	800	1	2.83E-09	0.444	5	0.625	Cyp39a1	Metabolism
DMR9:20437201	9	20437201	500	1	2.46E-07	0.549	2	0.4		
DMR9:21797201	9	21797201	500	1	4.34E-07	4.563	4	0.8		
DMR9:21931301	9	21931301	400	1	7.52E-07	3.985	8	2		
DMR9:23434201	9	23434201	200	1	2.58E-07	0.263	0	0	LOC688459;Cyp2ac1	Metabolism
DMR9:30493301	9	30493301	800	2	6.09E-08	0.589	1	0.125	Col9a1	Extracellular Matrix
DMR9:30838401	9	30838401	3700	1	2.59E-07	6.014	50	1.3514	Col19a1	Cytoskeleton
DMR9:40512501	9	40512501	7500	9	2.34E-08	0.669	75	1		
DMR9:40522101	9	40522101	2700	5	1.30E-10	0.664	28	1.037		
DMR9:42428501	9	42428501	1200	1	7.48E-07	3.184	5	0.4167		
DMR9:43979301	9	43979301	900	1	5.12E-09	0.299	16	1.7778	Inpp4a	
DMR9:44068901	9	44068901	500	1	4.18E-07	6.816	11	2.2	Mgat4a	Golgi
DMR9:45398701	9	45398701	300	1	2.45E-13	0.567	0	0		
DMR9:49736101	9	49736101	300	1	4.35E-08	0.337	2	0.6667		
DMR9:53968601	9	53968601	2800	1	3.06E-07	3.868	12	0.4286		
DMR9:55375201	9	55375201	700	3	2.91E-07	0.49	17	2.4286	Tmeff2	Signaling
DMR9:65236501	9	65236501	200	1	4.10E-08	0.447	3	1.5	Aox2	
DMR9:74198001	9	74198001	400	1	1.01E-07	0.512	4	1	Cps1	Unknown
DMR9:80178301	9	80178301	600	3	1.02E-12	0.594	3	0.5		
DMR9:82134901	9	82134901	300	1	7.52E-07	0.424	1	0.3333	Fev	Signaling
DMR9:86972201	9	86972201	300	1	1.50E-07	0.471	4	1.3333	NEWGENE_1305560	
DMR9:95455901	9	95455901	400	1	3.05E-07	0.439	3	0.75	Trpm8	Receptor
DMR9:97589501	9	97589501	500	1	2.18E-07	0.514	2	0.4		
DMR9:97787401	9	97787401	300	1	2.54E-07	0.314	5	1.6667	Rn50_9_0975.2;Cops8	Signaling
DMR9:104571001	9	104571001	700	1	9.70E-08	5.533	20	2.8571	AABR07068420.1	
DMR9:105054901	9	105054901	1400	1	4.11E-08	0.558	28	2		
DMR9:110200701	9	110200701	400	1	3.78E-10	0.615	2	0.5	Efna5	Signaling
DMR9:111277601	9	111277601	200	1	4.92E-07	0.198	6	3	Ppip5k2	
DMR9:115250801	9	115250801	400	1	3.84E-08	0.231	1	0.25	Ptprm	Receptor
DMR9:115402201	9	115402201	1200	1	6.64E-07	0.449	9	0.75	Ptprm	Receptor
DMR9:116855101	9	116855101	1500	1	4.61E-07	0.531	11	0.7333	L3mbtl4	Epigenetic
DMR9:118545601	9	118545601	500	1	7.53E-07	0.568	2	0.4		
DMR9:119160101	9	119160101	1400	1	8.71E-07	0.556	15	1.0714	Dlgap1	Signaling
DMR10:5412401	10	5412401	300	1	1.42E-09	0.452	0	0		
DMR10:7144301	10	7144301	400	2	1.40E-09	0.555	3	0.75	Abat	Metabolism
DMR10:7546501	10	7546501	400	1	8.38E-09	0.582	0	0		
DMR10:8000501	10	8000501	1000	2	5.53E-07	0.559	5	0.5		
DMR10:8017301	10	8017301	400	2	1.84E-08	0.518	1	0.25		
DMR10:10140301	10	10140301	400	1	4.97E-07	0.574	1	0.25		
DMR10:10228901	10	10228901	1100	1	3.21E-08	0.607	8	0.7273		
DMR10:16136901	10	16136901	1200	1	5.51E-07	0.422	9	0.75		
DMR10:16390201	10	16390201	600	1	5.45E-07	0.491	3	0.5		
DMR10:17568501	10	17568501	300	1	6.20E-07	0.244	3	1	Fbxw11	Metabolism
DMR10:19306601	10	19306601	800	1	2.80E-07	0.303	2	0.25		
DMR10:20125801	10	20125801	400	1	3.95E-08	0.489	2	0.5		
DMR10:22353301	10	22353301	200	1	4.03E-08	0.386	2	1		
DMR10:23129501	10	23129501	400	1	5.55E-08	0.559	0	0		

DMR10:33241201	10	33241201	200	1	8.30E-07	0.465	2	1		
DMR10:38152301	10	38152301	1700	1	4.32E-08	0.512	47	2.7647	Fstl4	Hormone
DMR10:40514101	10	40514101	400	1	1.59E-08	0.497	0	0	Slc36a2	
DMR10:48893401	10	48893401	1000	1	7.56E-07	0.232	16	1.6	AABR07029741.1;SNORA29;Trpv2	Transport
DMR10:52961401	10	52961401	1800	3	3.19E-07	0.583	7	0.3889	Shisa6	Development
DMR10:53811701	10	53811701	600	1	4.27E-08	0.431	5	0.8333	Myh2;Myh8	Cytoskeleton
DMR10:58038301	10	58038301	600	1	2.84E-10	0.518	1	0.1667		
DMR10:62336301	10	62336301	300	1	4.60E-07	4.912	4	1.3333		
DMR10:63672201	10	63672201	300	1	2.03E-10	0.173	13	4.3333	Scarf1;Slc43a2	Receptor;Metabolism
DMR10:64008401	10	64008401	600	3	6.30E-13	0.636	0	0	Rph3al	Transport
DMR10:67798801	10	67798801	300	1	8.33E-07	0.642	0	0		
DMR10:67947301	10	67947301	300	1	2.70E-08	0.532	1	0.3333	Myo1d	Cytoskeleton
DMR10:68286101	10	68286101	500	2	4.07E-10	0.587	1	0.2	Asic2	Transport
DMR10:68700201	10	68700201	300	1	1.84E-07	0.379	0	0		
DMR10:69553801	10	69553801	500	1	1.69E-07	0.515	3	0.6		
DMR10:69816001	10	69816001	900	1	4.96E-07	0.42	7	0.7778		
DMR10:76771401	10	76771401	500	1	8.69E-07	3.424	12	2.4		
DMR10:77016901	10	77016901	400	1	1.24E-07	0.392	5	1.25		
DMR10:77074701	10	77074701	500	1	7.17E-10	0.501	0	0	AABR07030235.1	
DMR10:78836301	10	78836301	600	3	2.11E-07	2.184	4	0.6667		
DMR10:82168401	10	82168401	500	1	1.55E-08	0.387	1	0.2	Cacna1g	Transport
DMR10:82264401	10	82264401	300	2	9.92E-11	0.411	2	0.6667		
DMR10:82720001	10	82720001	1000	2	3.85E-09	0.476	8	0.8		
DMR10:83966701	10	83966701	800	2	1.73E-07	0.56	2	0.25	Ttll6	Cytoskeleton
DMR10:85995101	10	85995101	400	1	1.41E-07	0.551	2	0.5	Stac2	Signaling
DMR10:86617501	10	86617501	400	1	1.97E-07	0.433	0	0	Psmd3;Csf3;Med24;SNORD124	Proteolysis;Signaling;Transcription
DMR10:89143001	10	89143001	300	2	7.45E-08	0.325	1	0.3333		
DMR10:92072101	10	92072101	1200	1	1.89E-07	0.539	4	0.3333		
DMR10:92520401	10	92520401	400	1	4.30E-07	4.078	1	0.25		
DMR10:96475201	10	96475201	300	1	4.26E-07	0.332	5	1.6667	Prkca	Binding Protein
DMR10:97771501	10	97771501	500	2	5.85E-09	0.544	1	0.2	Arsg	Metabolism
DMR10:97798001	10	97798001	800	1	2.44E-07	0.545	2	0.25	Arsg	Metabolism
DMR10:98218701	10	98218701	700	3	1.29E-11	0.377	0	0		
DMR10:98912401	10	98912401	300	1	1.32E-07	0.335	6	2		
DMR10:100670901	10	100670901	400	1	4.10E-07	0.373	0	0		
DMR10:101065101	10	101065101	500	2	4.67E-09	0.435	2	0.4		
DMR10:101774201	10	101774201	1800	1	1.10E-07	0.474	31	1.7222	Slc39a11	Metabolism
DMR10:102287801	10	102287801	200	1	3.91E-08	0.609	0	0	AABR07030729.1	
DMR10:107047101	10	107047101	1400	2	1.61E-13	0.503	30	2.1429	Dnah17	Cytoskeleton
DMR10:107108001	10	107108001	800	1	2.34E-07	0.517	3	0.375	Dnah17	Cytoskeleton
DMR10:107521701	10	107521701	500	1	2.56E-07	0.661	2	0.4	Engase;Rbfox3	Metabolism
DMR10:107846401	10	107846401	400	1	3.55E-08	0.446	1	0.25		
DMR10:109393901	10	109393901	300	1	1.35E-07	0.282	4	1.3333		
DMR10:110679501	10	110679501	1600	2	5.03E-11	0.621	13	0.8125	Tbcd	EST
DMR11:9512101	11	9512101	300	1	8.36E-08	0.557	0	0		
DMR11:14683501	11	14683501	500	1	3.58E-10	0.53	3	0.6	Nrip1	Transcription
DMR11:17160601	11	17160601	300	1	9.53E-08	0.253	3	1		
DMR11:24337701	11	24337701	1200	1	2.94E-07	0.567	3	0.25		
DMR11:25708901	11	25708901	400	2	5.98E-10	0.572	1	0.25		
DMR11:28759301	11	28759301	300	1	1.44E-07	0.278	2	0.6667		
DMR11:29918901	11	29918901	1800	1	1.87E-09	0.571	24	1.3333		
DMR11:30725801	11	30725801	3000	1	7.67E-07	0.623	15	0.5		
DMR11:31010701	11	31010701	1900	2	3.04E-08	0.66	26	1.3684	Eva1c	Unknown
DMR11:31463601	11	31463601	300	1	1.51E-08	0.499	0	0		
DMR11:32361601	11	32361601	700	1	6.90E-08	0.147	3	0.4286		
DMR11:34075801	11	34075801	400	1	9.80E-09	0.404	3	0.75	Morc3	Transcription
DMR11:35060701	11	35060701	500	2	6.62E-09	0.38	0	0	Kcnj6	Metabolism

DMR11:35626801	11	35626801	400	1	4.41E-07	0.611	5	1.25	AABR07033697.2	
DMR11:36176401	11	36176401	600	1	9.09E-09	0.534	1	0.1667		
DMR11:37416501	11	37416501	500	1	4.74E-07	5.184	3	0.6		
DMR11:39099701	11	39099701	1300	1	5.25E-07	2.149	10	0.7692		
DMR11:42175701	11	42175701	500	1	8.30E-07	0.461	0	0		
DMR11:46509101	11	46509101	600	1	1.43E-09	0.386	2	0.3333		
DMR11:58659301	11	58659301	1200	1	1.05E-09	0.462	12	1	Gap43;AABR07034249.1	
DMR11:59539001	11	59539001	500	2	1.23E-07	0.462	0	0		
DMR11:64326201	11	64326201	1800	3	1.62E-07	0.488	5	0.2778	Igsf11	Extracellular Matrix
DMR11:64970101	11	64970101	500	1	2.08E-09	0.479	3	0.6	Cox17;Maats1	Binding Protein
DMR11:65684601	11	65684601	600	1	6.75E-07	0.483	0	0		
DMR11:66627601	11	66627601	800	1	6.21E-07	0.535	5	0.625	Polq	Transcription
DMR11:68367401	11	68367401	200	1	3.12E-09	0.491	0	0		
DMR11:70194801	11	70194801	800	1	3.31E-07	0.58	5	0.625	Muc13	
DMR11:71225601	11	71225601	1100	1	3.84E-08	0.514	11	1	LOC100910650;Muc20	
DMR11:74231601	11	74231601	500	1	1.49E-08	0.524	5	1	AABR07034502.1	
DMR11:78590801	11	78590801	900	1	1.60E-11	0.336	5	0.5556		
DMR11:78723501	11	78723501	700	1	6.74E-07	0.434	0	0	Tprg1	Unknown
DMR11:82688001	11	82688001	400	2	2.38E-12	0.546	3	0.75	Liph	
DMR11:86949001	11	86949001	200	1	8.78E-09	0.588	0	0		
DMR11:88400001	11	88400001	500	2	3.62E-07	0.701	0	0	Igll1;SNORA17	Immune
DMR11:88744901	11	88744901	600	3	6.17E-09	0.606	2	0.3333	Fgd4	Signaling
DMR11:90459201	11	90459201	400	2	1.49E-11	0.503	0	0		
DMR12:157501	12	157501	300	1	2.01E-07	0.477	3	1		
DMR12:1341101	12	1341101	400	1	5.32E-07	0.529	2	0.5		
DMR12:1699101	12	1699101	1300	1	5.39E-07	0.455	6	0.4615	Insr	Receptor
DMR12:1877201	12	1877201	1100	1	3.34E-07	0.547	11	1		
DMR12:2250601	12	2250601	800	1	1.09E-07	0.519	12	1.5	Fcer2	Immune
DMR12:5653001	12	5653001	1100	1	1.79E-09	0.401	20	1.8182	Fry	Development
DMR12:6944301	12	6944301	300	1	5.75E-07	0.552	4	1.3333	Uspl1	
DMR12:7352701	12	7352701	1000	1	7.39E-08	0.482	2	0.2	Metazoa_SRP;AABR07035224.1	
DMR12:8137201	12	8137201	200	1	9.70E-08	0.212	2	1	Mtus2	Cytoskeleton
DMR12:8417601	12	8417601	1600	1	1.10E-08	0.277	41	2.5625	Mtus2	Cytoskeleton
DMR12:8432501	12	8432501	2000	2	4.50E-10	0.357	39	1.95		
DMR12:9408501	12	9408501	400	2	1.19E-11	0.591	0	0	Flt3	Receptor
DMR12:10112001	12	10112001	400	3	1.01E-11	0.442	1	0.25		
DMR12:11273601	12	11273601	300	2	1.36E-11	0.306	5	1.6667	Arpc1b;Arpc1a	Cytoskeleton
DMR12:11363201	12	11363201	1000	1	3.38E-07	0.565	5	0.5	Kpna7;AABR07035368.1	Unknown
DMR12:12039301	12	12039301	600	1	4.03E-08	3.094	26	4.3333		
DMR12:12600801	12	12600801	1500	2	4.73E-12	3.677	16	1.0667		
DMR12:14024101	12	14024101	700	1	3.54E-07	0.607	6	0.8571	Mmd2	Development
DMR12:15630901	12	15630901	2000	1	6.80E-07	0.553	3	0.15		
DMR12:15954001	12	15954001	1700	1	1.82E-09	0.422	12	0.7059	Iqce;Gna12	Signaling
DMR12:16877601	12	16877601	400	1	8.10E-08	0.549	2	0.5		
DMR12:17125301	12	17125301	300	1	4.38E-07	0.521	6	2		
DMR12:17441901	12	17441901	700	1	5.25E-07	0.476	7	1	Adap1	Transcription
DMR12:17877401	12	17877401	300	2	3.00E-09	0.269	0	0	LOC498155	
DMR12:20665401	12	20665401	700	1	1.14E-08	3.537	5	0.7143	RGD1561730;RGD1560281	Immune
DMR12:20815301	12	20815301	200	1	4.69E-07	5.006	1	0.5	AABR07035650.2;AABR07035650.1	
DMR12:22523501	12	22523501	200	1	5.46E-08	0.333	1	0.5	AABR07035790.1;AABR07035790.2	
DMR12:22541701	12	22541701	1100	1	1.32E-10	0.097	25	2.2727	AABR07035791.1	
DMR12:24224901	12	24224901	200	1	6.16E-07	0.285	0	0	Hip1	Cytoskeleton
DMR12:27466001	12	27466001	1600	1	1.16E-11	0.507	15	0.9375	AABR07035916.1	
DMR12:28760101	12	28760101	600	1	7.78E-08	0.563	2	0.3333	Wbscr17	Development
DMR12:29452301	12	29452301	1000	1	5.73E-07	0.535	11	1.1	Caln1	Signaling

DMR12:34429501	12	34429501	400	1	7.03E-08	0.444	1	0.25		
DMR12:36277701	12	36277701	1300	2	3.74E-08	0.538	0	0	Tmem132b	Unknown
DMR12:36480001	12	36480001	900	2	3.27E-09	0.542	5	0.5556		
DMR12:39959401	12	39959401	500	2	2.31E-09	0.504	4	0.8	Ccdc63;Myl2	Cytoskeleton
DMR12:40970701	12	40970701	800	1	5.73E-08	0.27	20	2.5	AABR07036376.1	
DMR12:42002001	12	42002001	300	1	1.63E-07	0.388	1	0.3333	Rbm19	Transcription
DMR12:42415101	12	42415101	500	1	6.35E-08	0.414	4	0.8		
DMR12:42940401	12	42940401	300	1	1.01E-07	0.524	0	0		
DMR12:43229001	12	43229001	500	1	1.17E-08	0.481	4	0.8	AABR07036435.1	
DMR12:44161101	12	44161101	400	1	7.66E-08	0.454	5	1.25	Tesc	Signaling
DMR12:45639001	12	45639001	600	2	1.52E-14	4.049	5	0.8333		
DMR12:45946401	12	45946401	2600	1	3.43E-07	0.397	37	1.4231	AABR07036514.1	
DMR12:46240101	12	46240101	3200	1	4.06E-07	6.077	40	1.25	7SK	
DMR12:47397101	12	47397101	600	1	4.59E-07	0.61	5	0.8333		
DMR12:48790501	12	48790501	1600	1	6.88E-07	0.506	59	3.6875	Cmklr1	Growth Factors & Cytokines
DMR12:48864001	12	48864001	400	1	8.28E-07	4.615	5	1.25	Wscd2	
DMR12:49766901	12	49766901	1300	1	4.51E-09	0.207	7	0.5385	Myo18b	Cytoskeleton
DMR12:49870001	12	49870001	1600	1	2.38E-07	0.495	21	1.3125	Myo18b	Cytoskeleton
DMR12:50065601	12	50065601	500	2	1.51E-09	0.438	2	0.4		
DMR12:50526801	12	50526801	1900	4	2.49E-13	2.224	24	1.2632		
DMR12:50627401	12	50627401	600	1	8.32E-08	0.431	1	0.1667	AABR07036626.1	
DMR12:50776201	12	50776201	2100	3	2.28E-12	3.01	58	2.7619		
DMR12:50992201	12	50992201	800	1	2.59E-09	0.633	4	0.5		
DMR12:51123401	12	51123401	300	1	5.51E-07	0.508	0	0		
DMR12:51200101	12	51200101	600	1	1.23E-07	0.593	3	0.5		
DMR12:51465701	12	51465701	300	2	3.20E-08	0.496	1	0.3333	Ttc28	Unknown
DMR12:52220001	12	52220001	400	1	8.05E-08	0.436	0	0	5S_rRNA	
DMR13:10360801	13	10360801	200	1	2.10E-07	0.604	0	0		
DMR13:14487601	13	14487601	400	1	2.71E-07	0.476	4	1	LOC304725	
DMR13:17222301	13	17222301	300	1	3.28E-07	0.424	0	0		
DMR13:19426801	13	19426801	100	1	1.61E-08	28.478	0	0		
DMR13:19632601	13	19632601	2000	2	2.23E-07	2.597	14	0.7		
DMR13:19643201	13	19643201	500	1	4.79E-07	3.626	7	1.4		
DMR13:19657901	13	19657901	3800	1	1.81E-08	2.29	44	1.1579		
DMR13:19740801	13	19740801	900	1	4.19E-07	13.814	3	0.3333		
DMR13:26753401	13	26753401	300	1	3.12E-08	0.12	0	0	Bcl2	Signaling
DMR13:27069901	13	27069901	500	1	7.48E-07	0.538	1	0.2	Serpinb13	Protease
DMR13:27234001	13	27234001	400	1	3.32E-07	0.471	0	0	Serpinb11	Signaling
DMR13:30576801	13	30576801	2800	1	8.02E-07	2.836	26	0.9286		
DMR13:34021401	13	34021401	300	1	1.69E-07	0.593	2	0.6667		
DMR13:34876701	13	34876701	400	3	2.09E-08	0.358	2	0.5	Gli2	Transcription
DMR13:35179301	13	35179301	1800	1	8.78E-09	0.359	20	1.1111		
DMR13:36636901	13	36636901	600	1	8.04E-08	0.54	5	0.8333		
DMR13:41770101	13	41770101	300	1	2.38E-07	0.487	0	0		
DMR13:43068801	13	43068801	700	1	2.87E-07	0.507	2	0.2857		
DMR13:47518801	13	47518801	100	1	9.64E-09	0.098	0	0		
DMR13:48230401	13	48230401	200	1	4.06E-07	0.194	0	0	Srgap2	Signaling
DMR13:48911701	13	48911701	2500	4	8.67E-12	0.597	30	1.2	Cdk18	Cell Cycle
DMR13:49651301	13	49651301	400	1	3.25E-07	0.438	4	1	Lrrn2	Development
DMR13:51661101	13	51661101	700	3	2.12E-09	0.415	3	0.4286	Ppp1r12b	Signaling
DMR13:51672001	13	51672001	600	1	2.34E-08	0.489	3	0.5	Ppp1r12b	Signaling
DMR13:52182701	13	52182701	500	1	7.26E-10	0.473	3	0.6	Lmod1	Cytoskeleton
DMR13:68568401	13	68568401	400	1	6.95E-09	0.469	4	1		
DMR13:70467901	13	70467901	600	3	1.28E-10	0.575	1	0.1667	Nmnat2	Metabolism
DMR13:70572701	13	70572701	1600	3	3.14E-15	0.239	20	1.25	Lamc2	Cytoskeleton
DMR13:70819101	13	70819101	800	1	6.30E-07	0.453	9	1.125		
DMR13:71136201	13	71136201	300	1	1.38E-09	0.492	5	1.6667	Rgs8;AABR07021419.1	Signaling
DMR13:73174501	13	73174501	1000	1	7.38E-07	0.262	28	2.8	Rn60_13_0732.1	

DMR13:73402801	13	73402801	1200	1	2.58E-09	0.64	13	1.0833	Lhx4	Transcription
DMR13:73810501	13	73810501	1400	3	1.22E-09	0.581	14	1	Fam163a	
DMR13:75282001	13	75282001	800	1	4.04E-07	0.371	6	0.75		
DMR13:75608801	13	75608801	200	1	9.50E-07	0.363	0	0		
DMR13:79183801	13	79183801	400	1	3.90E-08	0.599	0	0		
DMR13:80392701	13	80392701	3200	1	9.35E-07	0.693	34	1.0625	Rn50_13_0853.1	
DMR13:80569301	13	80569301	500	1	6.21E-07	0.529	9	1.8		
DMR13:80581901	13	80581901	300	1	3.84E-08	0.604	6	2		
DMR13:81548701	13	81548701	600	1	9.11E-08	0.606	7	1.1667		
DMR13:83505601	13	83505601	200	1	4.76E-07	0.534	0	0	Tiprl	
DMR13:83566201	13	83566201	1200	2	8.26E-08	0.6	7	0.5833	Gpr161	Receptor
DMR13:89441701	13	89441701	6900	3	3.00E-10	0.62	213	3.087	Fcgr2b	Immune
DMR13:89986301	13	89986301	2000	2	4.68E-07	0.6	41	2.05	Cd244	Receptor
DMR13:91205301	13	91205301	3500	2	1.45E-11	0.616	92	2.6286	LOC100911825;LOC108348047	Receptor
DMR13:91520001	13	91520001	200	1	9.29E-07	0.313	2	1	AABR07021799.1	
DMR13:104082401	13	104082401	300	1	2.26E-08	0.525	6	2	Lyplal1;U7	Metabolism
DMR13:104304701	13	104304701	200	2	1.71E-08	0.546	0	0	Dusp10	Signaling
DMR13:104504901	13	104504901	400	2	1.59E-14	0.505	6	1.5		
DMR13:104867201	13	104867201	400	2	9.67E-09	0.408	1	0.25		
DMR13:105434601	13	105434601	1700	1	4.94E-08	0.699	18	1.0588		
DMR13:106427201	13	106427201	1400	1	1.38E-09	0.49	12	0.8571		
DMR13:106789401	13	106789401	500	2	7.29E-08	0.692	11	2.2	Ush2a	Extracellular Matrix
DMR13:107950601	13	107950601	500	1	5.97E-07	0.571	2	0.4		
DMR13:110121301	13	110121301	200	2	1.78E-08	0.488	0	0		
DMR13:113096501	13	113096501	2200	1	3.64E-07	6.864	12	0.5455		
DMR14:5203701	14	5203701	300	1	1.34E-07	0.412	0	0		
DMR14:6684601	14	6684601	1300	1	4.19E-07	4.349	7	0.5385	Spp1	Signaling
DMR14:6811301	14	6811301	1900	1	9.50E-08	0.322	14	0.7368	Ibsp	Signaling
DMR14:7738801	14	7738801	300	1	4.54E-07	0.393	14	4.6667	Ptpn13	Signaling
DMR14:11033201	14	11033201	500	1	2.36E-09	0.469	10	2		
DMR14:11077301	14	11077301	400	1	5.38E-07	0.414	12	3		
DMR14:11096601	14	11096601	200	1	2.69E-07	0.262	4	2	Tmem150c	Unknown
DMR14:11218801	14	11218801	1800	2	1.87E-08	0.604	0	0		
DMR14:13503001	14	13503001	300	2	3.38E-09	0.49	5	1.6667		
DMR14:13919601	14	13919601	300	1	1.61E-09	0.553	0	0		
DMR14:16748801	14	16748801	400	1	7.34E-08	0.48	2	0.5	Shroom3	Cytoskeleton
DMR14:17267501	14	17267501	2400	1	1.71E-07	0.64	49	2.0417	Sdad1	
DMR14:22404101	14	22404101	100	1	3.74E-08	14.234	0	0	Ugt2a1;Ugt2b37	Metabolism
DMR14:23498701	14	23498701	1700	2	2.36E-07	0.376	20	1.1765	Gnrhr;Uba6	Receptor;Proteolysis
DMR14:33205901	14	33205901	11500	2	2.52E-07	2.403	55	0.4783		
DMR14:34295101	14	34295101	300	1	4.58E-07	0.437	1	0.3333		
DMR14:34403601	14	34403601	500	1	9.08E-07	0.438	2	0.4	Pdcl2	
DMR14:36589601	14	36589601	300	2	2.46E-10	0.387	0	0	AC114452.1	
DMR14:37240001	14	37240001	300	1	1.81E-07	0.246	7	2.3333	Dcun1d4	Proteolysis
DMR14:42453401	14	42453401	2300	1	9.20E-07	0.475	43	1.8696		
DMR14:42869401	14	42869401	1200	1	1.52E-07	0.235	14	1.1667	Limch1	Cytoskeleton
DMR14:42956801	14	42956801	300	1	4.26E-07	0.327	4	1.3333	Limch1	Cytoskeleton
DMR14:44487601	14	44487601	400	1	7.95E-07	0.495	1	0.25	Ugdh	Metabolism
DMR14:44791201	14	44791201	600	2	1.90E-08	0.591	3	0.5	AABR07015006.1	
DMR14:46637401	14	46637401	24300	5	2.03E-08	0.606	2210	9.0947	pRNA;Rn5-8s;AABR07015078.1;AABR07015078.2;AABR07015079.1;AABR07015080.2;LOC257642;AABR07015080.1;AABR07015081.1	
DMR14:46678801	14	46678801	10300	3	1.55E-10	0.573	1068	10.3689	pRNA;5_8S_rRNA	
DMR14:52933301	14	52933301	200	1	5.38E-07	0.426	0	0		
DMR14:60463501	14	60463501	300	1	6.88E-09	0.587	1	0.3333	Anapc4	Cell Cycle

DMR14:70073901	14	70073901	2600	1	9.02E-08	5.786	19	0.7308	Fam184b	
DMR14:70659001	14	70659001	500	1	4.54E-08	0.558	4	0.8		
DMR14:77126301	14	77126301	200	1	2.77E-07	0.401	1	0.5	Slc2a9	Metabolism
DMR14:77659301	14	77659301	2500	1	5.32E-07	0.441	31	1.24	AABR07015800.1;AABR07015800.2	
DMR14:80039401	14	80039401	1200	1	3.09E-07	0.453	3	0.25	Ablim2	Cytoskeleton
DMR14:80323901	14	80323901	3900	3	3.25E-07	0.716	49	1.2564	Acox3	Metabolism
DMR14:81141901	14	81141901	600	1	7.78E-07	0.595	6	1	Htt	Development
DMR14:81457601	14	81457601	500	1	7.41E-10	0.504	1	0.2	Sh3bp2;U1	Translation
DMR14:84307201	14	84307201	600	1	4.14E-07	0.584	0	0	Sec14l4	Transport
DMR14:91808701	14	91808701	500	3	1.12E-07	0.554	1	0.2	Ikzf1	Transcription
DMR14:96400201	14	96400201	1700	1	3.07E-07	3.666	13	0.7647		
DMR14:103388301	14	103388301	300	1	7.45E-08	0.337	4	1.3333	AABR07016556.2	
DMR14:109239801	14	109239801	1200	2	1.85E-10	0.512	15	1.25		
DMR14:114713901	14	114713901	300	1	7.75E-07	0.47	1	0.3333		
DMR15:2711401	15	2711401	600	1	4.14E-07	0.643	12	2	Samd8;AABR07016873.1	Metabolism
DMR15:2929001	15	2929001	300	1	8.09E-07	0.395	1	0.3333	Kat6b	Epigenetic
DMR15:4445501	15	4445501	1800	1	3.68E-11	0.554	26	1.4444	Nudt13	Metabolism
DMR15:4586401	15	4586401	200	1	7.30E-07	0.256	7	3.5	Kcnk5	Transport
DMR15:5479301	15	5479301	400	1	1.45E-08	0.615	4	1	Spetex-2F;AABR07016950.1	Unknown
DMR15:13431401	15	13431401	800	3	8.86E-10	0.564	3	0.375		
DMR15:14111101	15	14111101	1100	2	5.41E-11	0.5	5	0.4545		
DMR15:15103101	15	15103101	1200	1	7.22E-07	0.636	14	1.1667		
DMR15:15510001	15	15510001	600	1	1.77E-07	0.344	1	0.1667	Cadps	Metabolism
DMR15:18496901	15	18496901	5400	6	9.28E-14	0.552	72	1.3333	Kctd6	Transport
DMR15:19797401	15	19797401	200	1	9.75E-07	0.291	6	3		
DMR15:19849701	15	19849701	200	1	2.80E-10	0.531	6	3	Fermt2	
DMR15:21611601	15	21611601	1300	1	3.00E-07	0.491	9	0.6923		
DMR15:23000001	15	23000001	500	1	5.58E-08	3.575	2	0.4		
DMR15:23010401	15	23010401	1700	1	3.16E-07	0.559	33	1.9412		
DMR15:23013301	15	23013301	900	2	4.58E-07	2.841	8	0.8889		
DMR15:23021801	15	23021801	3800	1	9.67E-08	2.406	79	2.0789		
DMR15:36825201	15	36825201	800	2	1.22E-07	0.559	7	0.875	Parp4	
DMR15:40212101	15	40212101	1500	2	2.40E-09	0.326	2	0.1333	Atp8a2	Transport
DMR15:44154201	15	44154201	700	1	6.18E-10	0.495	10	1.4286		
DMR15:48015801	15	48015801	2100	1	3.09E-08	0.606	23	1.0952		
DMR15:48744101	15	48744101	1200	1	1.11E-08	0.437	5	0.4167		
DMR15:51373701	15	51373701	1100	1	4.02E-08	0.502	28	2.5455	Loxl2;AABR07018321.2;R3hcc1	Metabolism
DMR15:53935701	15	53935701	1500	1	2.03E-07	0.502	8	0.5333		
DMR15:55010901	15	55010901	500	1	3.48E-07	0.497	0	0		
DMR15:56208101	15	56208101	200	1	3.61E-07	0.563	0	0		
DMR15:57730201	15	57730201	600	1	3.23E-08	0.588	2	0.3333		
DMR15:61233701	15	61233701	200	1	5.16E-07	0.383	0	0		
DMR15:62937001	15	62937001	500	3	4.12E-12	0.592	0	0		
DMR15:65188801	15	65188801	1600	1	9.81E-07	3.032	13	0.8125	AABR07018574.1	
DMR15:65285201	15	65285201	900	1	5.71E-07	3.849	7	0.7778		
DMR15:67199601	15	67199601	200	1	3.99E-07	0.667	0	0		
DMR15:76829801	15	76829801	500	2	1.21E-12	0.429	2	0.4		
DMR15:78122501	15	78122501	500	1	8.27E-09	0.656	0	0		
DMR15:79490401	15	79490401	200	1	5.13E-07	0.39	2	1		
DMR15:84328101	15	84328101	400	1	3.65E-07	0.491	0	0	Klf12	Transcription
DMR15:91578801	15	91578801	500	3	6.81E-09	0.583	2	0.4	Mycbp2	Metabolism
DMR15:104699101	15	104699101	500	1	7.87E-07	0.407	1	0.2	Hs6st3	Metabolism
DMR15:108477701	15	108477701	300	1	1.42E-09	0.487	0	0		
DMR15:108869901	15	108869901	600	1	2.90E-07	0.355	8	1.3333	AC123185.3	
DMR16:124601	16	124601	1100	1	7.08E-08	0.388	5	0.4545		
DMR16:605801	16	605801	900	1	4.14E-09	0.396	7	0.7778	AABR07024473.2	

DMR16:1427901	16	1427901	400	1	6.58E-09	0.535	3	0.75		
DMR16:1844401	16	1844401	2200	3	2.19E-10	0.58	2	0.0909	Zmiz1	Metabolism
DMR16:2068701	16	2068701	1900	1	2.47E-07	0.426	22	1.1579	AABR07024500.1	
DMR16:2185801	16	2185801	400	1	2.37E-08	0.492	0	0	Slmap;SNORA71	Protein Binding
DMR16:8717801	16	8717801	400	1	2.34E-07	0.43	0	0		
DMR16:10549601	16	10549601	500	1	2.94E-07	0.642	4	0.8	Syt15	Transport
DMR16:11166801	16	11166801	400	1	9.37E-07	0.507	1	0.25		
DMR16:11694001	16	11694001	500	1	8.78E-09	0.578	3	0.6	Grid1	Receptor
DMR16:12656501	16	12656501	500	1	9.92E-11	0.448	4	0.8	RGD1559508	Unknown
DMR16:18370601	16	18370601	300	1	2.54E-07	0.307	1	0.3333	AABR07024825.1	
DMR16:18439601	16	18439601	300	1	1.33E-07	0.308	4	1.3333	Fam60a	
DMR16:20851601	16	20851601	300	1	4.96E-09	0.516	1	0.3333	Upf1;Gdf1;Cers1	Transcription;Growth Factors & Cytokines
DMR16:21773501	16	21773501	2900	1	2.10E-08	0.668	14	0.4828	AABR07024907.1	
DMR16:22013301	16	22013301	1900	1	2.41E-07	0.562	56	2.9474	RGD1563748	
DMR16:22115001	16	22115001	4600	1	2.31E-07	0.688	51	1.1087	RGD1563748	
DMR16:26229001	16	26229001	1300	1	8.98E-07	0.294	14	1.0769		
DMR16:30802301	16	30802301	2000	1	5.21E-07	4.012	21	1.05		
DMR16:39915101	16	39915101	300	1	5.03E-07	0.501	3	1	Wdr17	
DMR16:40060601	16	40060601	700	1	2.59E-07	0.186	5	0.7143	Spcs3	Protease
DMR16:43155401	16	43155401	500	1	7.75E-07	0.484	2	0.4		
DMR16:45516901	16	45516901	500	1	4.73E-07	0.502	1	0.2		
DMR16:46280801	16	46280801	300	1	5.75E-07	0.584	0	0		
DMR16:49838201	16	49838201	1100	1	4.50E-08	0.458	6	0.5455		
DMR16:54203001	16	54203001	100	1	1.97E-07	0.26	0	0		
DMR16:60601501	16	60601501	300	1	2.10E-08	0.398	10	3.3333		
DMR16:60624601	16	60624601	1000	1	1.12E-07	0.515	11	1.1		
DMR16:63001901	16	63001901	200	1	5.01E-08	0.411	0	0		
DMR16:66360701	16	66360701	1700	1	9.18E-07	0.554	9	0.5294	LOC689479	Unknown
DMR16:68333301	16	68333301	300	1	3.18E-09	0.39	1	0.3333		
DMR16:68841301	16	68841301	300	1	8.89E-09	0.52	0	0	Poteg	
DMR16:68975401	16	68975401	300	1	8.72E-09	0.247	1	0.3333	Eif4ebp1;Rn60_16_0690.3	Transcription
DMR16:70613301	16	70613301	300	1	1.05E-10	0.537	0	0		
DMR16:71868401	16	71868401	1200	2	2.41E-09	0.502	2	0.1667	Adam9	Protease
DMR16:72106801	16	72106801	1100	2	5.10E-10	0.44	14	1.2727	Adam3a	
DMR16:72208201	16	72208201	900	1	2.61E-07	2.128	3	0.3333	Adam18;Ido1	Protease;Signaling
DMR16:72696001	16	72696001	600	1	5.79E-07	0.658	10	1.6667	AABR07026339.1	
DMR16:74336601	16	74336601	500	1	6.12E-09	0.599	3	0.6	Slc20a2	Metabolism
DMR16:75924801	16	75924801	1100	1	7.29E-07	0.529	30	2.7273	Mcph1	DNA Repair
DMR16:79083601	16	79083601	300	1	4.49E-08	0.509	1	0.3333	AABR07026499.3	
DMR16:79318001	16	79318001	300	1	4.45E-09	0.536	1	0.3333		
DMR16:80318201	16	80318201	400	1	4.62E-07	4.76	7	1.75		
DMR16:80898301	16	80898301	800	1	5.71E-07	0.297	19	2.375	Tmco3	Transport
DMR16:83696401	16	83696401	500	1	2.88E-08	0.668	7	1.4		
DMR16:84031701	16	84031701	300	1	2.55E-10	0.463	1	0.3333		
DMR16:84654401	16	84654401	700	1	9.31E-07	0.53	1	0.1429	Myo16	Cytoskeleton
DMR16:84695301	16	84695301	300	1	7.65E-07	0.435	0	0	Myo16	Cytoskeleton
DMR16:86499101	16	86499101	300	2	1.33E-07	0.538	0	0		
DMR16:88332401	16	88332401	200	1	4.57E-09	0.532	0	0		
DMR17:947401	17	947401	400	2	1.51E-11	0.559	0	0	Fancc	
DMR17:4056201	17	4056201	400	1	1.52E-07	0.489	1	0.25	LOC100364523;Ctsql2	
DMR17:5033001	17	5033001	500	2	2.67E-08	0.369	1	0.2		
DMR17:6313501	17	6313501	400	2	6.42E-07	0.361	0	0		
DMR17:6565801	17	6565801	400	1	9.16E-07	0.413	7	1.75		
DMR17:7167901	17	7167901	1300	2	4.28E-10	0.573	18	1.3846		
DMR17:7190501	17	7190501	2000	2	2.97E-11	0.608	23	1.15		
DMR17:8593301	17	8593301	800	2	1.55E-08	0.458	6	0.75	Slc25a48	
DMR17:9742701	17	9742701	600	1	1.46E-07	0.58	2	0.3333	F12;Pfn3;Slc34a1	Protease;Cytoskeleton; Metabolism

DMR17:10243501	17	10243501	400	2	2.36E-10	0.536	1	0.25		
DMR17:11754001	17	11754001	600	2	1.34E-07	0.633	4	0.6667		
DMR17:11760201	17	11760201	1900	4	9.98E-12	0.582	18	0.9474		
DMR17:11799301	17	11799301	1800	1	4.77E-09	0.651	22	1.2222		
DMR17:11925001	17	11925001	1300	1	1.30E-07	0.229	24	1.8462	RGD1561671	
DMR17:11929101	17	11929101	200	1	2.74E-07	0.441	0	0		
DMR17:12606501	17	12606501	500	2	1.57E-08	0.491	4	0.8	Syk	Signaling
DMR17:12998401	17	12998401	400	1	7.39E-07	0.557	2	0.5		
DMR17:13169501	17	13169501	300	1	6.77E-07	0.516	4	1.3333		
DMR17:14004201	17	14004201	1200	1	2.28E-07	13.93	13	1.0833		
DMR17:15685001	17	15685001	600	2	1.91E-12	0.552	3	0.5	Bicd2	
DMR17:17142201	17	17142201	700	1	2.36E-08	0.461	9	1.2857	AABR07027157.1	
DMR17:17600401	17	17600401	900	1	3.23E-07	0.47	11	1.2222		
DMR17:19522801	17	19522801	1500	2	2.90E-15	0.564	11	0.7333	Atxn1	Transcription
DMR17:19760201	17	19760201	1300	2	1.98E-09	0.406	22	1.6923		
DMR17:19878401	17	19878401	400	1	4.55E-11	0.598	0	0		
DMR17:21143301	17	21143301	500	1	3.76E-08	0.608	8	1.6		
DMR17:21456901	17	21456901	300	1	9.85E-08	0.422	7	2.3333	Sycp2l	
DMR17:25019601	17	25019601	400	1	8.66E-07	0.529	7	1.75		
DMR17:29837701	17	29837701	600	3	6.85E-11	0.586	0	0	Cdyl	Metabolism
DMR17:39442601	17	39442601	200	1	6.97E-07	4.335	2	1	Prl8a4	
DMR17:43752701	17	43752701	200	1	2.11E-08	0.492	0	0		
DMR17:54612301	17	54612301	500	1	5.44E-07	0.42	1	0.2		
DMR17:56190501	17	56190501	700	2	9.16E-09	0.454	7	1		
DMR17:69602501	17	69602501	400	1	1.39E-07	0.62	2	0.5	Akr1c12l1	Metabolism
DMR17:72872601	17	72872601	600	1	1.99E-07	0.545	4	0.6667		
DMR17:74005801	17	74005801	300	1	4.14E-07	0.622	3	1	AABR07028534.1;AABR07028535.1	
DMR17:76302801	17	76302801	900	2	4.43E-08	0.412	6	0.6667	AC141220.1;Dhtkd1	Metabolism
DMR17:81281701	17	81281701	400	1	2.32E-07	0.567	3	0.75	Tmem236	
DMR17:82084501	17	82084501	400	2	2.37E-11	0.403	1	0.25	Arl5b	Translation
DMR17:85010901	17	85010901	200	1	8.87E-08	0.222	5	2.5	Dnajc1	Protein Binding
DMR17:85992701	17	85992701	300	1	2.23E-07	0.389	0	0	Armc3	
DMR17:86187001	17	86187001	300	1	5.43E-07	0.448	1	0.3333		
DMR17:89432501	17	89432501	1200	1	1.70E-08	0.481	5	0.4167		
DMR17:89452301	17	89452301	6000	1	1.26E-07	0.672	54	0.9	RGD1561231	
DMR17:90299401	17	90299401	1400	1	3.36E-08	0.347	21	1.5	Gng4	Signaling
DMR18:4167301	18	4167301	300	1	4.04E-08	0.618	1	0.3333	Osbpl1a	Binding Protein
DMR18:6587101	18	6587101	500	1	1.95E-08	0.576	4	0.8	LOC103694404	
DMR18:13487301	18	13487301	600	4	7.66E-10	0.534	0	0	Asxl3	
DMR18:17176801	18	17176801	600	3	1.77E-09	0.54	0	0	Fhod3;Rn60_18_0172.2	Cytoskeleton
DMR18:24409901	18	24409901	5100	2	2.68E-07	0.526	42	0.8235	Sap130	Transcription
DMR18:26894101	18	26894101	600	1	1.49E-08	0.6	4	0.6667		
DMR18:31973301	18	31973301	600	4	1.78E-13	0.531	0	0	Arhgap26	Signaling
DMR18:34631101	18	34631101	300	1	4.62E-07	4.92	1	0.3333		
DMR18:40305301	18	40305301	300	1	3.92E-07	3.455	2	0.6667	AABR07031952.1	
DMR18:40520801	18	40520801	300	1	6.40E-08	0.592	0	0	Fem1c	Unknown
DMR18:43677601	18	43677601	300	1	2.64E-07	0.474	2	0.6667		
DMR18:51234801	18	51234801	200	1	1.03E-07	0.463	1	0.5		
DMR18:56335001	18	56335001	300	1	2.18E-07	0.536	1	0.3333	Slc6a7;Cdx1	Metabolism;Transcription
DMR18:57175801	18	57175801	2000	2	1.15E-10	0.589	4	0.2	Ablim3	Cytoskeleton
DMR18:57229901	18	57229901	1400	1	3.17E-08	0.559	12	0.8571	Ablim3	Cytoskeleton
DMR18:57310901	18	57310901	600	1	1.41E-07	0.371	6	1	Sh3tc2	Development
DMR18:57540701	18	57540701	1300	2	1.42E-07	0.559	3	0.2308	AABR07032261.3;AABR07032261.4	
DMR18:59926501	18	59926501	600	1	6.63E-07	0.467	2	0.3333		
DMR18:70151701	18	70151701	300	1	5.09E-07	0.648	1	0.3333		
DMR18:70943501	18	70943501	600	2	5.25E-09	0.494	11	1.8333		

DMR18:70979701	18	70979701	300	1	5.40E-07	0.321	3	1	SNORD58;Rpl17;RGD1562987	Translation;EST
DMR18:71357801	18	71357801	900	1	2.46E-07	0.352	44	4.8889		
DMR18:72887701	18	72887701	1200	1	1.61E-07	0.691	20	1.6667		
DMR18:73614601	18	73614601	400	2	8.36E-12	0.315	1	0.25	St8sia5	Metabolism
DMR18:73747801	18	73747801	400	1	5.56E-11	0.547	0	0	Loxhd1	
DMR18:76894201	18	76894201	300	1	5.57E-12	0.497	0	0		
DMR18:77234301	18	77234301	1400	1	2.13E-07	0.315	38	2.7143	Nfatc1	Transcription
DMR18:77395601	18	77395601	400	1	1.14E-07	0.624	2	0.5	Atp9b	Transport
DMR18:78869201	18	78869201	400	1	8.64E-09	0.614	0	0		
DMR18:79438101	18	79438101	2700	3	1.04E-08	2.591	28	1.037	Mbp	Unknown
DMR18:79732701	18	79732701	2000	1	4.04E-09	0.518	40	2		
DMR18:79924301	18	79924301	200	2	8.48E-09	0.206	1	0.5		
DMR18:79927201	18	79927201	1100	1	4.73E-07	0.577	13	1.1818		
DMR18:80084401	18	80084401	1000	2	4.48E-12	0.275	15	1.5		
DMR18:80191401	18	80191401	100	1	4.73E-07	5.112	3	3	AABR07032758.1	
DMR18:80608901	18	80608901	3200	1	2.19E-11	0.357	88	2.75		
DMR18:81005401	18	81005401	700	3	2.32E-10	0.472	2	0.2857		
DMR18:81383001	18	81383001	1400	1	9.56E-08	0.558	12	0.8571	Zfp407	Transcription
DMR18:81612501	18	81612501	900	1	2.44E-07	0.492	3	0.3333		
DMR18:81672901	18	81672901	200	1	1.69E-07	0.222	4	2	LOC100359752;7SK	
DMR18:87797401	18	87797401	1300	2	4.93E-11	2.31	24	1.8462		
DMR19:306401	19	306401	5100	2	2.16E-13	0.585	33	0.6471	AABR07042611.1	
DMR19:3444301	19	3444301	300	1	3.86E-07	0.442	1	0.3333		
DMR19:5829101	19	5829101	6400	1	8.74E-08	0.633	121	1.8906		
DMR19:8323201	19	8323201	800	1	9.48E-10	0.566	12	1.5		
DMR19:13937101	19	13937101	800	1	1.90E-07	0.517	2	0.25		
DMR19:14401801	19	14401801	100	1	1.30E-17	0.063	2	2	Tom1	Signaling
DMR19:15148701	19	15148701	300	1	6.09E-08	0.625	4	1.3333	Ces1d;Ces1f	Metabolism
DMR19:15937901	19	15937901	600	1	2.60E-10	0.184	3	0.5		
DMR19:19075301	19	19075301	1800	2	5.72E-08	0.459	21	1.1667		
DMR19:19943401	19	19943401	300	1	5.25E-07	0.291	0	0		
DMR19:22137001	19	22137001	300	1	3.42E-08	2.612	3	1	Phkb	Signaling
DMR19:23362801	19	23362801	400	1	2.12E-09	0.543	4	1		
DMR19:26331701	19	26331701	400	1	9.81E-07	0.344	2	0.5		
DMR19:28131101	19	28131101	400	1	1.65E-07	0.519	2	0.5		
DMR19:28160901	19	28160901	1500	1	4.19E-07	13.116	12	0.8		
DMR19:38934801	19	38934801	600	3	3.87E-10	0.295	3	0.5	Tango6	
DMR19:39266801	19	39266801	1900	1	7.53E-07	0.585	16	0.8421	Cog8;Nip7;Tmed6;Terf2	Golgi;Transcription;Binding Protein
DMR19:46136101	19	46136101	800	1	8.41E-09	0.476	2	0.25		
DMR19:47274401	19	47274401	500	1	7.36E-07	0.49	2	0.4		
DMR19:48069901	19	48069901	1100	1	1.81E-07	0.398	4	0.3636		
DMR19:48943901	19	48943901	300	1	4.41E-07	0.479	1	0.3333		
DMR19:49747901	19	49747901	500	1	5.14E-09	0.534	5	1	Gan	Cytoskeleton
DMR19:52295401	19	52295401	800	1	5.60E-07	0.517	0	0		
DMR19:52419301	19	52419301	2900	3	5.12E-08	0.461	38	1.3103	Tldc1	Unknown
DMR19:53316601	19	53316601	400	2	1.47E-08	0.551	4	1		
DMR19:55152301	19	55152301	700	1	2.19E-07	0.339	14	2	Zfpm1	Transcription
DMR19:55973501	19	55973501	300	1	4.37E-07	0.198	6	2	Sult5a1;Dpep1	Metabolism
DMR19:56336201	19	56336201	400	2	1.42E-09	0.565	19	4.75	Gas8;5S_rRNA	Cell Cycle
DMR19:56337701	19	56337701	1700	6	9.04E-14	0.625	85	5	Gas8;5S_rRNA	Cell Cycle
DMR20:2584701	20	2584701	600	2	8.16E-08	0.53	2	0.3333	Rn50_20_0026.4	
DMR20:4024901	20	4024901	500	1	3.83E-07	0.515	7	1.4	RT1-DOb	Immune
DMR20:4765201	20	4765201	1400	1	8.49E-08	0.669	16	1.1429	RT1-CE1;RT1-CE4;RT1-CE6;RT1-CE7	Immune
DMR20:6324401	20	6324401	400	2	1.07E-07	0.343	8	2	Rn60_20_0064.1	
DMR20:6709201	20	6709201	900	2	8.42E-10	0.287	34	3.7778	Ppil1	Immune
DMR20:8044901	20	8044901	1300	3	7.93E-11	0.595	12	0.9231		

DMR20:9543801	20	9543801	300	1	1.23E-07	0.379	6	2	Dnah8	Cytoskeleton
DMR20:9580701	20	9580701	900	1	2.14E-08	0.583	9	1	Glp1r	Receptor
DMR20:10179301	20	10179301	500	1	3.43E-08	0.414	3	0.6	Pde9a	Signaling
DMR20:10284401	20	10284401	500	1	6.30E-07	0.442	2	0.4	Ndufv3;SNORA36	Metabolism
DMR20:12157201	20	12157201	1000	1	3.38E-08	0.358	26	2.6		
DMR20:13070801	20	13070801	600	1	3.58E-07	0.687	10	1.6667	Dip2a	
DMR20:13447801	20	13447801	700	1	2.12E-07	0.401	2	0.2857	Slc5a4	Transport
DMR20:14403201	20	14403201	700	2	9.87E-11	0.499	0	0	Specc1l	
DMR20:19259201	20	19259201	600	1	4.31E-07	0.589	5	0.8333		
DMR20:20501301	20	20501301	1800	1	1.21E-08	0.484	13	0.7222		
DMR20:23391301	20	23391301	1300	3	1.63E-11	0.622	1	0.0769		
DMR20:23907401	20	23907401	300	1	4.66E-08	0.291	2	0.6667		
DMR20:26002301	20	26002301	500	1	3.71E-07	0.3	1	0.2	Lrrtm3	Receptor
DMR20:26919601	20	26919601	600	2	2.91E-07	0.543	3	0.5	Dnajc12	Protein Binding
DMR20:28594801	20	28594801	1200	1	7.33E-07	0.205	9	0.75	Sh3rf3	
DMR20:29846101	20	29846101	2700	1	1.10E-07	0.571	61	2.2593	Psap;Cdh23	Signaling;Extracellular Matrix
DMR20:30473701	20	30473701	300	1	1.25E-08	0.398	0	0		
DMR20:31823501	20	31823501	400	1	7.06E-09	0.461	3	0.75	Tspan15	Extracellular Matrix
DMR20:42391801	20	42391801	400	1	7.22E-07	0.389	3	0.75	AABR07045313.1	
DMR20:44324301	20	44324301	1500	1	4.92E-07	0.669	10	0.6667		
DMR20:45007301	20	45007301	200	1	2.09E-09	0.509	1	0.5	Mfsd4b;RGD1304770	
DMR20:46358701	20	46358701	2400	1	2.84E-08	0.366	36	1.5		
DMR20:46777401	20	46777401	400	1	2.53E-07	0.463	3	0.75	Armc2	Transcription
DMR20:47157401	20	47157401	600	1	3.54E-09	0.469	6	1	Lace1;AABR07045427.1	Metabolism
DMR20:47664601	20	47664601	200	1	2.93E-08	3.312	5	2.5	Scml4	Transcription
DMR20:48143701	20	48143701	1700	2	9.40E-11	0.608	13	0.7647	Pdss2	Metabolism
DMR20:50334101	20	50334101	300	1	3.20E-09	0.539	0	0		
DMRX:143301	X	143301	3200	1	2.44E-09	3.909	42	1.3125		
DMRX:2200201	X	2200201	6000	2	1.83E-08	0.677	32	0.5333		
DMRX:2207901	X	2207901	800	1	3.35E-08	0.683	9	1.125		
DMRX:2211801	X	2211801	10100	10	2.69E-10	0.687	78	0.7723		
DMRX:2341701	X	2341701	4600	5	1.37E-08	0.663	29	0.6304		
DMRX:2347501	X	2347501	2800	1	2.64E-07	0.658	17	0.6071		
DMRX:2383801	X	2383801	1700	3	1.47E-07	0.673	8	0.4706		
DMRX:11117101	X	11117101	200	1	7.33E-07	0.327	1	0.5	5S_rRNA	
DMRX:14652501	X	14652501	300	1	7.65E-09	11.362	2	0.6667		
DMRX:14951401	X	14951401	4200	16	1.13E-12	0.625	21	0.5	SNORA26;Slc38a5	Unknown
DMRX:18223701	X	18223701	1900	1	8.29E-07	0.614	21	1.1053		
DMRX:18891501	X	18891501	300	1	7.43E-09	0.457	8	2.6667	Klf8	Transcription
DMRX:25932601	X	25932601	200	1	9.80E-07	0.346	1	0.5		
DMRX:35089101	X	35089101	300	1	7.88E-07	0.538	0	0		
DMRX:35262601	X	35262601	3300	4	1.78E-10	0.677	18	0.5455		
DMRX:35266901	X	35266901	2700	12	3.06E-11	0.679	20	0.7407		
DMRX:35270701	X	35270701	500	2	4.52E-10	0.66	2	0.4		
DMRX:35272601	X	35272601	600	1	9.61E-07	0.691	15	2.5		
DMRX:36455401	X	36455401	1200	2	8.42E-08	0.881	28	2.3333		
DMRX:49376001	X	49376001	300	1	1.38E-08	11.3	1	0.3333		
DMRX:73094101	X	73094101	2300	3	5.79E-15	0.59	27	1.1739	Rn50_X_0740.2	
DMRX:106167001	X	106167001	1900	7	5.43E-09	0.661	13	0.6842		
DMRX:106172001	X	106172001	2500	3	2.18E-11	0.666	12	0.48		
DMRX:106175701	X	106175701	5700	12	1.10E-10	0.67	30	0.5263		
DMRX:109827101	X	109827101	200	1	1.96E-08	0.567	0	0	Il1rapl2	Receptor
DMRX:112386401	X	112386401	8200	3	9.69E-08	0.587	58	0.7073	Atg4a	Metabolism
DMRX:120821201	X	120821201	200	2	6.32E-10	0.41	5	2.5		
DMRX:140094601	X	140094601	2200	1	8.35E-07	2.843	22	1		
DMRX:150002401	X	150002401	200	1	1.80E-07	3.731	2	1		
DMRX:150165301	X	150165301	400	1	1.63E-07	5.677	0	0		
DMRX:150557901	X	150557901	500	1	4.95E-07	2.62	4	0.8		

DMRX:153076201	X	153076201	300	1	2.80E-07	0.294	3	1	Pnma3;Xlr4a	
DMRX:154665601	X	154665601	300	1	3.32E-07	0.376	6	2		
DMRX:155840101	X	155840101	4200	2	4.10E-08	0.559	102	2.4286	Dkc1	Transcription
DMRX:157278701	X	157278701	200	1	1.35E-07	0.285	12	6	Atp2b3	Transport

Supplemental Table S3
(A) DMR Site Table Vinclozolin Prostate Epithelial Epi Str Overlap (p<1e-06)

DMR Name	Chr	Start	Length	# SigWin	Min P-value	CpG #	CpG Density	Gene Annotation	Gene Category
DMR1:5738301	1	5738301	200	2	3.26E-15	0	0	SNORA17	
DMR1:22736601	1	22736601	400	1	2.19E-19	2	0.5	Slc18b1	
DMR1:179687901	1	179687901	2500	2	2.76E-07	38	1.52		
DMR1:180242101	1	180242101	2300	1	4.83E-09	36	1.565217391		
DMR1:263684201	1	263684201	900	1	1.88E-07	1	0.111111111	Dnmbp	EST
DMR1:280046301	1	280046301	700	1	1.44E-08	0	0		
DMR10:5412401	10	5412401	200	1	4.24E-09	0	0		
DMR12:1341101	12	1341101	600	1	7.56E-07	6	1		
DMR12:8137201	12	8137201	200	1	4.01E-10	2	1	Mtus2	Cytoskeleton
DMR12:11273501	12	11273501	400	2	7.05E-11	9	2.25	Arpc1b;Arpc1a	Cytoskeleton
DMR12:12601401	12	12601401	1000	1	4.86E-11	4	0.4		
DMR12:50526701	12	50526701	900	4	4.00E-13	9	1		
DMR12:50774501	12	50774501	2900	2	8.59E-08	47	1.620689655		
DMR13:19657901	13	19657901	2500	3	2.55E-08	38	1.52		
DMR13:70572601	13	70572601	1800	1	6.87E-09	20	1.111111111	Lamc2	Cytoskeleton
DMR13:75608801	13	75608801	200	1	1.35E-07	0	0		
DMR15:23010301	15	23010301	3900	3	1.94E-08	79	2.025641026		
DMR15:23024501	15	23024501	1100	2	2.03E-10	15	1.363636364		
DMR15:40212401	15	40212401	1000	1	7.67E-07	2	0.2	Atp8a2	Transport
DMR16:30801501	16	30801501	2000	1	9.07E-07	22	1.1		
DMR17:82084601	17	82084601	1200	1	1.37E-07	22	1.833333333	Arl5b	Translation
DMR18:79438201	18	79438201	1100	1	2.24E-07	10	0.909090909	Mbp	Unknown
DMR18:80608001	18	80608001	2500	1	7.77E-10	71	2.84		
DMR18:87797401	18	87797401	900	1	9.01E-12	9	1		
DMR19:14401801	19	14401801	100	1	3.01E-09	2	2	Tom1	Signaling
DMR19:15937901	19	15937901	400	1	2.49E-08	0	0		
DMR19:38934801	19	38934801	700	3	2.18E-13	3	0.428571429	Tango6	
DMR2:161037301	2	161037301	700	2	3.55E-11	11	1.571428571		
DMR2:226852901	2	226852901	1400	1	6.20E-08	5	0.357142857		
DMR20:45007301	20	45007301	200	1	3.41E-09	1	0.5	Mfsd4b;RGD1304770	
DMR3:23694201	3	23694201	200	2	7.25E-15	2	1		
DMR3:159097301	3	159097301	500	1	2.82E-09	10	2	LOC103694889	
DMR4:77401101	4	77401101	11200	1	1.10E-08	368	3.285714286	AABR07060519.1	
DMR4:158928401	4	158928401	600	2	2.53E-10	5	0.833333333		
DMR4:173488601	4	173488601	300	3	4.62E-13	9	3		
DMR5:121133101	5	121133101	3000	1	6.67E-07	72	2.4		
DMR5:138699301	5	138699301	2700	1	6.51E-07	15	0.555555556	Guca2b	Signaling
DMR6:31062601	6	31062601	9300	2	3.77E-08	169	1.817204301		
DMR6:134504101	6	134504101	1100	2	3.28E-09	26	2.363636364		
DMR7:119494001	7	119494001	900	1	3.38E-07	4	0.444444444	Ncf4	Development
DMR7:121631101	7	121631101	200	2	1.59E-08	6	3		
DMR8:8224501	8	8224501	300	1	2.88E-07	10	3.333333333	Cntn5	Extracellular Matrix
DMR8:10628701	8	10628701	900	1	1.15E-07	8	0.888888889		
DMR8:12109201	8	12109201	800	1	1.46E-07	2	0.25	Maml2	Transcription
DMR8:14259101	8	14259101	600	2	8.22E-13	26	4.333333333	Slc36a4	Transport
DMR8:88267801	8	88267801	400	1	3.59E-07	3	0.75		
DMR8:127473901	8	127473901	1100	3	2.12E-07	1	0.090909091	Itga9	Extracellular Matrix
DMR9:10507301	9	10507301	400	1	9.67E-07	1	0.25		
DMRX:36455601	X	36455601	800	2	5.86E-08	17	2.125		

(B) DMR Site Table Vinclozolin Prostate Stromal Epi Str Overlap (p<1e-06)

DMR Name	Chr	Start	Length	# Sig Win	Min P-value	CpG #	CpG Density	Gene Annotation	Gene Category
DMR1:5738301	1	5738301	100	1	1.41E-17	0	0	SNORA17	
DMR1:22736501	1	22736501	300	1	8.68E-16	0	0	Slc18b1	
DMR1:179687901	1	179687901	3700	4	1.80E-08	43	1.162162162		
DMR1:180242001	1	180242001	2300	1	1.02E-12	36	1.565217391		
DMR1:263684301	1	263684301	800	1	5.25E-07	0	0	Dnmbp	EST
DMR1:280046301	1	280046301	900	1	2.52E-07	0	0		
DMR10:5412401	10	5412401	300	1	1.42E-09	0	0		
DMR12:1341101	12	1341101	400	1	5.32E-07	2	0.5		
DMR12:8137201	12	8137201	200	1	9.70E-08	2	1	Mtus2	Cytoskeleton
DMR12:11273601	12	11273601	300	2	1.36E-11	5	1.666666667	Arpc1b;Arpc1a	Cytoskeleton
DMR12:12600801	12	12600801	1500	2	4.73E-12	16	1.066666667		
DMR12:50526801	12	50526801	1900	4	2.49E-13	24	1.263157895		
DMR12:50776201	12	50776201	2100	3	2.28E-12	58	2.761904762		
DMR13:19657901	13	19657901	3800	1	1.81E-08	44	1.157894737		
DMR13:70572701	13	70572701	1600	3	3.14E-15	20	1.25	Lamc2	Cytoskeleton
DMR13:75608801	13	75608801	200	1	9.50E-07	0	0		
DMR15:23010401	15	23010401	1700	1	3.16E-07	33	1.941176471		
DMR15:23013301	15	23013301	900	2	4.58E-07	8	0.888888889		
DMR15:23021801	15	23021801	3800	1	9.67E-08	79	2.078947368		
DMR15:40212101	15	40212101	1500	2	2.40E-09	2	0.133333333	Atp8a2	Transport
DMR16:30802301	16	30802301	2000	1	5.21E-07	21	1.05		
DMR17:82084501	17	82084501	400	2	2.37E-11	1	0.25	Arl5b	Translation
DMR18:79438101	18	79438101	2700	3	1.04E-08	28	1.037037037	Mbp	Unknown
DMR18:80608901	18	80608901	3200	1	2.19E-11	88	2.75		
DMR18:87797401	18	87797401	1300	2	4.93E-11	24	1.846153846		
DMR19:14401801	19	14401801	100	1	1.30E-17	2	2	Tom1	Signaling
DMR19:15937901	19	15937901	600	1	2.60E-10	3	0.5		
DMR19:38934801	19	38934801	600	3	3.87E-10	3	0.5	Tango6	
DMR2:161037401	2	161037401	1900	2	3.97E-07	27	1.421052632		
DMR2:226853001	2	226853001	500	2	9.19E-09	0	0		
DMR20:45007301	20	45007301	200	1	2.09E-09	1	0.5	Mfsd4b;RGD1304770	
DMR3:23694201	3	23694201	200	2	1.05E-13	2	1		
DMR3:159097301	3	159097301	500	3	4.47E-10	10	2	LOC103694889	
DMR4:77399901	4	77399901	12400	14	1.01E-11	376	3.032258065	AABR07060519.1	
DMR4:158928301	4	158928301	800	1	3.58E-08	6	0.75		
DMR4:173488701	4	173488701	200	2	2.69E-17	5	2.5		
DMR5:121133301	5	121133301	2800	1	8.05E-07	68	2.428571429		
DMR5:138699301	5	138699301	1700	2	6.60E-11	1	0.058823529	Guca2b	Signaling
DMR6:31062801	6	31062801	7100	1	6.96E-07	135	1.901408451		
DMR6:134502801	6	134502801	2300	1	2.34E-07	61	2.652173913		
DMR7:119494001	7	119494001	1000	2	2.18E-10	6	0.6	Ncf4	Development
DMR7:121631101	7	121631101	200	2	2.54E-13	6	3		
DMR8:8224501	8	8224501	300	1	6.37E-07	10	3.333333333	Cntn5	Extracellular Matrix
DMR8:10626201	8	10626201	3500	1	5.07E-08	41	1.171428571		
DMR8:12109101	8	12109101	900	1	7.46E-07	4	0.444444444	Maml2	Transcription
DMR8:14259101	8	14259101	600	3	1.10E-22	26	4.333333333	Slc36a4	Transport
DMR8:88267801	8	88267801	600	1	1.02E-09	3	0.5		
DMR8:127473901	8	127473901	1100	1	1.95E-07	1	0.090909091	Itga9	Extracellular Matrix
DMR9:10507301	9	10507301	500	3	8.34E-19	2	0.4		
DMRX:36455401	X	36455401	1200	2	8.42E-08	28	2.333333333		

Supplemental Table S4
Site Table sncRNA Prostate Epithelial (p<0.001)

id	Chr	Start	log2FoldChange	pvalue	padj
rno-miR-450a-3p:MIMAT0017183	X	158589811	3.32	5.50E-04	1.45E-02
rno-miR-450b-5p:MIMAT0035746	X	158589644	1.78	1.31E-05	9.41E-04
rno-miR-503-5p:MIMAT0003213	X	158584862	3.37	1.89E-14	6.53E-11
rno-miR-322-3p:MIMAT0000547	X	158584602	2.66	1.39E-10	1.59E-07
rno-miR-201-5p:MIMAT0012846	X	155339151	-2.45	3.45E-04	1.09E-02
rno-miR-135b-5p:MIMAT0000611	13	48977001	-5.55	9.55E-05	4.57E-03
rno-miR-503-3p:MIMAT0017224	X	158148521	2.05	2.15E-04	8.23E-03
rno-miR-322-5p:MIMAT0001619	X	158584564	2	1.45E-06	2.28E-04
rno-miR-202-5p:MIMAT0012822	1	212295675	-1.68	1.70E-04	7.00E-03
rno-miR-450b-3p:MIMAT0035747	X	158153296	3.67	4.72E-06	4.52E-04
rno-miR-547-3p:MIMAT0012851	X	155338889	-3.22	6.47E-08	2.79E-05
rno-miR-129-5p:MIMAT0000600	4	56301731	-4.54	6.79E-04	1.66E-02
rno-miR-450a-5p:MIMAT0001547	X	158153394	1.99	2.89E-06	3.44E-04
rno-miR-201-3p:MIMAT0017364	X	155339186	-3.83	8.05E-06	6.78E-04
rno-miR-351-5p:MIMAT0000608	X	158149221	2.36	2.81E-07	7.46E-05
piR-rno-220	3	120268801	-6.49	1.08E-05	8.69E-04
piR-rno-372	5	78076115	-5.63	6.03E-04	1.54E-02
piR-rno-451	20	6680118	-6.55	1.20E-05	9.27E-04
piR-rno-623	1	135182573	-6.64	8.06E-06	6.78E-04
piR-rno-1013	20	6662285	-5.6	9.91E-04	2.08E-02
piR-rno-1195	20	6651490	-6.07	8.64E-05	4.26E-03
piR-rno-1709	20	6648907	-5.37	1.86E-04	7.37E-03
piR-rno-1833	20	6630280	-5.33	4.39E-04	1.26E-02
piR-rno-1938	20	6684687	-5.82	2.68E-04	9.07E-03
piR-rno-1949	15	245749	-5.11	7.15E-04	1.73E-02
piR-rno-2490	3	146945235	-6.17	5.57E-05	2.78E-03
piR-rno-3102	20	6634461	-5.31	3.79E-04	1.16E-02
piR-rno-3337	20	6639763	-6.63	4.45E-06	4.39E-04
piR-rno-3623	20	6680109	-6.21	4.64E-05	2.42E-03
piR-rno-3686	1	131021248	-6.26	4.20E-05	2.30E-03
piR-rno-3988	6	122382919	-8.24	3.93E-10	3.10E-07
piR-rno-4043	1	135146666	-5.88	2.31E-04	8.32E-03
piR-rno-4065	11	87120655	-5.79	3.63E-04	1.12E-02
piR-rno-4301	20	6660191	-6.96	8.40E-07	1.53E-04
piR-rno-5960	20	6653039	-5.15	5.09E-04	1.39E-02
piR-rno-5970	20	6639416	-5.53	9.70E-04	2.07E-02
piR-rno-6124	20	6643420	-5.69	4.47E-04	1.28E-02
piR-rno-6623	15	276897	-6.11	1.17E-04	5.46E-03
piR-rno-6676	20	6626739	-6.35	2.14E-05	1.34E-03
piR-rno-6777	20	6679528	-7.47	4.12E-08	2.03E-05

piR-rno-6999	20	6634562	-5.54	9.98E-04	2.08E-02
piR-rno-7000	4	160380812	-6.43	1.48E-05	1.02E-03
piR-rno-7161	1	135146667	-5.77	4.16E-04	1.23E-02
piR-rno-7819	20	6623847	-6.85	2.73E-06	3.37E-04
piR-rno-7850	20	6651613	-5.56	8.39E-04	1.88E-02
piR-rno-8155	20	6645194	-5.6	7.41E-04	1.73E-02
piR-rno-8940	20	6680121	-7.6	2.73E-08	1.57E-05
piR-rno-9032	15	245751	-5.56	7.55E-04	1.73E-02
piR-rno-9247	1	54889999	-5.59	9.33E-04	2.01E-02
piR-rno-9474	20	6630799	-5.63	5.82E-04	1.50E-02
piR-rno-9804	X	155338889	-2.5	1.24E-05	9.27E-04
piR-rno-9874	20	6655630	-5.79	4.69E-04	1.32E-02
piR-rno-9899	NA	NA	-6.94	1.70E-06	2.49E-04
piR-rno-10003	20	6631677	-5.5	9.79E-04	2.07E-02
piR-rno-10535	20	6653153	-5.73	4.33E-04	1.26E-02
piR-rno-10554	12	49067299	-5.15	6.12E-04	1.55E-02
piR-rno-10929	20	6652053	-9.04	3.13E-12	5.39E-09
piR-rno-10962	20	6642039	-5.53	8.91E-04	1.95E-02
piR-rno-10980	20	6626741	-6.05	1.43E-04	6.30E-03
piR-rno-11689	20	6642860	-5.82	3.23E-04	1.04E-02
piR-rno-12125	NA	NA	-5.74	4.07E-04	1.21E-02
piR-rno-12143	20	6660023	-6.05	1.28E-04	5.82E-03
piR-rno-12297	20	6654839	-5.95	1.56E-04	6.64E-03
piR-rno-12739	20	6680786	-6.59	6.37E-06	5.63E-04
piR-rno-13064	4	117651976	-6.6	5.81E-06	5.28E-04
piR-rno-13457	20	6653039	-5.58	7.86E-04	1.77E-02
piR-rno-13695	8	73555829	-5.61	7.58E-04	1.73E-02
piR-rno-14106	5	153978738	-4.67	7.50E-04	1.73E-02
piR-rno-14184	20	6662987	-7.23	4.66E-07	1.00E-04
piR-rno-14382	X	107054233	-6.41	1.83E-05	1.17E-03
piR-rno-14406	20	6669159	-4.21	2.23E-04	8.23E-03
piR-rno-14617	20	6623914	-6.65	4.34E-06	4.39E-04
piR-rno-14798	X	107054233	-5.3	2.75E-04	9.21E-03
piR-rno-14879	3	120268801	-5.69	5.28E-04	1.41E-02
piR-rno-14881	7	99560734	-6.01	1.35E-04	6.05E-03
piR-rno-15105	X	151274374	-1.63	6.76E-04	1.66E-02
piR-rno-15550	X	155330302	-3.12	1.63E-04	6.75E-03
piR-rno-15619	20	6668525	-6.84	2.59E-06	3.31E-04
piR-rno-15683	12	6521839	-6.33	3.33E-05	1.98E-03
piR-rno-15704	20	6672989	-5.19	1.49E-07	5.15E-05
piR-rno-16346	20	6642863	-6.72	3.22E-06	3.70E-04
piR-rno-16585	20	6680101	-6.9	2.35E-06	3.24E-04
piR-rno-16699	20	6676695	-5.74	4.07E-04	1.21E-02

piR-rno-16818	20	6635475	-7.11	4.31E-07	9.92E-05
piR-rno-17331	8	73566343	-6.05	1.19E-04	5.46E-03
piR-rno-17387	20	6679199	-6.85	1.74E-06	2.49E-04
piR-rno-17402	10	91522069	-5.83	2.34E-04	8.32E-03
piR-rno-17998	20	6668528	-7.11	3.31E-07	8.14E-05
piR-rno-18085	18	60914182	-5.8	3.93E-04	1.19E-02
piR-rno-18254	20	6623524	-5.81	3.58E-04	1.11E-02
piR-rno-19037	20	6651886	-5.67	6.17E-04	1.55E-02
piR-rno-19617	7	119885479	-6.26	5.39E-05	2.73E-03
piR-rno-20568	20	6651884	-5.95	1.65E-05	1.10E-03
piR-rno-21165	20	6680490	-5.91	2.54E-04	8.92E-03
piR-rno-21623	20	6632039	-7.02	5.21E-07	1.06E-04
piR-rno-22267	20	6659331	-6.07	8.98E-06	7.37E-04
piR-rno-22887	20	6632151	-5.7	5.29E-04	1.41E-02
piR-rno-22906	20	6622830	-6.05	1.47E-04	6.37E-03
piR-rno-22908	20	6623920	-5.9	2.01E-04	7.89E-03
piR-rno-23207	20	6635476	-5.95	1.79E-04	7.25E-03
piR-rno-23823	20	6638130	-5.72	5.01E-05	2.58E-03
piR-rno-24292	20	6636383	-5.61	7.58E-04	1.73E-02
piR-rno-24907	20	6676886	-5.26	3.24E-04	1.04E-02
piR-rno-25584	20	6678933	-5.92	2.66E-04	9.07E-03
piR-rno-25698	20	6637849	-5.91	3.47E-04	1.09E-02
piR-rno-25913	20	6630085	-5.55	8.48E-04	1.89E-02
piR-rno-26293	4	160352079	-5.66	5.31E-04	1.41E-02
piR-rno-26309	20	6680341	-6.17	1.10E-04	5.19E-03
piR-rno-26408	20	6675345	-6.22	4.60E-05	2.42E-03
piR-rno-26467	20	6630259	-7.01	4.30E-04	1.26E-02
piR-rno-26564	20	6627709	-5.84	2.56E-04	8.92E-03
piR-rno-27441	20	6630414	-5.37	2.86E-04	9.47E-03
piR-rno-27656	3	81482722	-6.47	1.26E-05	9.27E-04
piR-rno-27796	15	245746	-6.11	5.54E-06	5.16E-04
piR-rno-27923	9	114168972	-5.56	8.66E-04	1.92E-02
piR-rno-27989	1	135182573	-5.59	7.30E-04	1.73E-02
piR-rno-28264	20	6627707	-7.19	2.73E-07	7.46E-05
piR-rno-28549	20	6672641	-6.5	1.43E-05	1.01E-03
piR-rno-29588	20	6644630	-5.66	5.05E-04	1.39E-02
piR-rno-29714	7	99563060	-5.64	7.04E-04	1.71E-02
piR-rno-29849	1	134436389	-5.04	7.48E-04	1.73E-02
piR-rno-29859	20	6653567	-5.52	9.76E-04	2.07E-02
piR-rno-29885	20	6674880	-6.4	1.70E-05	1.10E-03
piR-rno-30402	5	91131795	1.83	1.16E-06	2.00E-04
piR-rno-31427	6	122382879	-6.07	8.82E-05	4.29E-03
piR-rno-31568	4	160354342	-5.78	4.63E-04	1.31E-02

piR-rno-31788	20	6631493	-5.6	7.44E-04	1.73E-02
piR-rno-31810	X	155339186	-4.88	2.24E-07	7.03E-05
piR-rno-32475	3	81472305	-5.7	4.95E-04	1.38E-02
piR-rno-32845	20	6664106	-5.57	9.11E-04	1.98E-02
piR-rno-33223	15	245748	-6.23	3.88E-05	2.23E-03
piR-rno-33464	1	135195735	-5.59	6.61E-04	1.64E-02
piR-rno-34239	3	81476825	-6.4	1.70E-05	1.10E-03
piR-rno-34653	20	6628538	-5.27	3.08E-04	1.01E-02
piR-rno-34667	20	6671547	-6.29	2.50E-06	3.31E-04
piR-rno-36144	X	155338889	-2.8	3.62E-06	3.91E-04
piR-rno-36179	20	6676627	-7.35	4.49E-10	3.10E-07
piR-rno-36660	1	131021292	-5.59	8.89E-04	1.95E-02
piR-rno-37046	20	6680490	-6.67	4.01E-06	4.19E-04
piR-rno-38236	20	6680786	-6.26	4.61E-05	2.42E-03
piR-rno-40563	1	131025168	-5.56	7.66E-04	1.74E-02
piR-rno-41278	NA	NA	-5.45	2.22E-04	8.23E-03
piR-rno-41664	11	81378400	-1.83	1.62E-04	6.75E-03
piR-rno-44184	4	117830858	-5.61	6.25E-04	1.56E-02
piR-rno-45634	NA	NA	-5.86	2.66E-04	9.07E-03
piR-rno-45755	20	6673141	-6.31	2.92E-05	1.77E-03
piR-rno-50608	12	6521838	-6.29	4.01E-05	2.25E-03
piR-rno-50675	NA	NA	-6.28	3.54E-05	2.07E-03
piR-rno-53124	NA	NA	-5.67	5.64E-04	1.47E-02
piR-rno-54005	5	78078495	-6	2.10E-04	8.13E-03
piR-rno-54090	20	6669070	-6.36	2.22E-05	1.37E-03
piR-rno-54872	NA	NA	-5.11	5.74E-04	1.49E-02
piR-rno-55529	7	99564978	-7.06	7.02E-07	1.35E-04
piR-rno-56024	20	6647108	-6.92	1.36E-06	2.24E-04
piR-rno-56843	6	122382920	-6.31	8.52E-08	3.27E-05
piR-rno-57230	20	6680110	-5.75	3.41E-04	1.09E-02
piR-rno-57549	NA	NA	-5.69	5.22E-04	1.41E-02
piR-rno-57625	18	60904360	-5.35	2.24E-04	8.23E-03
piR-rno-60510	20	6673738	-5.98	1.48E-04	6.37E-03
piR-rno-61093	NA	NA	-5.89	1.81E-04	7.25E-03
piR-rno-61203	1	109521611	-5.71	4.04E-05	2.25E-03
piR-rno-62794	5	129142658	1.93	2.32E-04	8.32E-03
piR-rno-62997	10	65291234	-2.51	2.23E-04	8.23E-03
piR-rno-63055	X	158153394	1.87	1.26E-05	9.27E-04
piR-rno-63080	X	158584564	2	3.38E-06	3.76E-04

Supplemental Table S5
Site Table sncRNA Prostate Stromal (p<0.001)

id	Chr	Start	log2FoldChange	pvalue	padj
rno-miR-204-3p:MIMAT0004739	1	240403071	3.15	3.45E-04	2.30E-02
rno-miR-136-5p:MIMAT0000842	6	133716775	2.6	3.09E-04	2.25E-02
rno-miR-3473:MIMAT0024853	10	75205084	3.2	4.95E-05	6.62E-03
rno-miR-342-5p:MIMAT0004652	6	132561187	2.26	9.17E-04	4.20E-02
Rattus_norvegicus_chr17.trna1606-AlaAGC:(45815808	17	43962065	-3.12	1.17E-06	3.56E-04
Rattus_norvegicus_chr1.trna6805-AlaAGC:(225683168	1	218810648	-3.09	3.61E-04	2.32E-02
Rattus_norvegicus_chr17.trna3936-AlaAGC:(45793269	17	43938525	-3.22	3.30E-04	2.25E-02
Rattus_norvegicus_chr2.trna2176-AlaAGC:(124127980	2	104404891	-3.07	2.69E-06	7.48E-04
Rattus_norvegicus_chr6.trna7740-AlaAGC:(36671663-	2	104404891	-2.3	1.96E-04	1.72E-02
Rattus_norvegicus_chr4.trna1517-ArgCCT:(66087628-	4	66276179	-1.66	9.77E-04	4.30E-02
Rattus_norvegicus_chr10.trna13306-ArgCCT:(1269871	10	12877346	-3.39	2.41E-07	1.15E-04
Rattus_norvegicus_chrX.trna1999-ArgCCT:(82600032-	X	82631971	-3.46	8.76E-05	9.75E-03
Rattus_norvegicus_chr10.trna13315-ArgCCT:(1267121	10	12849847	-3.3	4.07E-04	2.52E-02
Rattus_norvegicus_chr2.trna4358-GlyCCC:(217947735	2	198461976	-2.15	1.20E-04	1.22E-02
Rattus_norvegicus_chr13.trna3830-LeuCAG:(96493702	13	91978414	-3.6	1.41E-09	4.72E-06
Rattus_norvegicus_chrM.trna1-LeuTAA:(2665-2739)::	MT	2665	-2.17	2.61E-05	4.15E-03
Rattus_norvegicus_chr17.trna1587-SerAGA:(45707665	17	43851269	1.89	8.74E-04	4.20E-02
Rattus_norvegicus_chr5.trna455-SerAGA:(28086029-2	17	44674536	1.86	9.64E-04	4.29E-02
piR-rno-220	3	120268801	-5.98	1.19E-04	1.22E-02
piR-rno-2490	3	146945235	-5.61	4.88E-04	2.72E-02
piR-rno-3102	20	6634461	-5.72	3.17E-04	2.25E-02
piR-rno-3686	1	131021248	-5.48	8.82E-04	4.20E-02
piR-rno-3988	6	122382919	-7.49	7.33E-08	6.85E-05
piR-rno-4301	20	6660191	-6.5	1.05E-05	2.18E-03
piR-rno-6777	20	6679528	-7.21	3.61E-07	1.51E-04
piR-rno-7819	20	6623847	-5.75	2.91E-04	2.25E-02
piR-rno-8940	20	6680121	-5.73	6.26E-05	7.75E-03
piR-rno-10929	20	6652053	-5.67	8.61E-08	6.85E-05
piR-rno-11689	20	6642860	-5.56	6.65E-04	3.32E-02
piR-rno-12189	20	6683531	-5.59	5.38E-04	2.81E-02
piR-rno-13064	4	117651976	-4.62	4.88E-04	2.72E-02
piR-rno-14184	20	6662987	-6.06	8.12E-05	9.68E-03
piR-rno-14406	20	6669159	-5.94	1.30E-04	1.28E-02
piR-rno-14617	20	6623914	-5.15	5.25E-04	2.81E-02
piR-rno-14798	X	107054233	-5.62	5.32E-04	2.81E-02
piR-rno-15619	20	6668525	-5.89	1.60E-04	1.48E-02
piR-rno-15704	20	6672989	-6.72	3.56E-06	8.48E-04
piR-rno-16346	20	6642863	-6.05	8.75E-05	9.75E-03
piR-rno-16585	20	6680101	-5.78	2.59E-04	2.11E-02
piR-rno-16818	20	6635475	-6.52	1.12E-05	2.21E-03
piR-rno-19617	7	119885479	-5.44	9.54E-04	4.29E-02
piR-rno-20568	20	6651884	-6.21	4.14E-05	6.02E-03
piR-rno-21623	20	6632039	-6.15	5.77E-05	7.41E-03
piR-rno-23823	20	6638130	-5.57	5.79E-04	2.93E-02
piR-rno-27656	3	81482722	-5.82	2.55E-04	2.11E-02
piR-rno-27796	15	245746	-6.6	7.98E-06	1.78E-03

piR-rno-30402	5	91131795	2.45	8.23E-07	2.75E-04
piR-rno-34667	20	6671547	-6.76	3.21E-06	8.24E-04
piR-rno-36179	20	6676627	-6.81	5.31E-07	1.97E-04
piR-rno-40536	10	65443464	-1.74	3.97E-04	2.50E-02
piR-rno-40648	2	189865693	-2.07	1.88E-04	1.70E-02
piR-rno-40649	NA	NA	-2	9.12E-04	4.20E-02
piR-rno-41278	NA	NA	-5.6	5.31E-04	2.81E-02
piR-rno-41525	5	91134984	2.27	4.56E-04	2.72E-02
piR-rno-41721	4	127537621	4.33	5.70E-04	2.93E-02
piR-rno-41910	14	46646166	2.27	4.89E-04	2.72E-02
piR-rno-50642	NA	NA	2.16	2.75E-04	2.19E-02
piR-rno-50788	NA	NA	2.25	9.07E-04	4.20E-02
piR-rno-54090	20	6669070	-5.79	2.42E-04	2.07E-02
piR-rno-55529	7	99564978	-5.19	4.70E-04	2.72E-02
piR-rno-56843	6	122382920	-7.38	1.36E-07	7.55E-05
piR-rno-61203	1	109521611	-5.27	3.53E-04	2.31E-02
piR-rno-62771	17	44660505	-1.69	8.99E-04	4.20E-02
piR-rno-62883	14	46646165	2.24	2.56E-05	4.15E-03
piR-rno-62898	1	11970092	2.23	2.83E-05	4.29E-03
piR-rno-62912	14	46646165	2.1	2.42E-05	4.15E-03
piR-rno-63032	6	133716775	2.58	3.25E-04	2.25E-02
ENSRNOT00000088337.1:ncrna:chromosome:Rnor_6.0:10	10	34151395	-2.25	3.13E-04	2.25E-02
ENSRNOT00000081514.1:ncrna:chromosome:Rnor_6.0:1:	1	100222088	-5.24	4.95E-05	6.62E-03
ENSRNOT00000086797.1:ncrna:chromosome:Rnor_6.0:2:	2	75154117	-2.43	1.42E-04	1.35E-02
ENSRNOT00000077870.1:ncrna:chromosome:Rnor_6.0:6:	6	81835387	-2.37	3.21E-04	2.25E-02
ENSRNOT00000091975.1:ncrna:chromosome:Rnor_6.0:1:	1	135581305	-1.96	4.46E-04	2.71E-02
ENSRNOT00000045072.3:ncrna:chromosome:Rnor_6.0:MT	MT	1026	-1.98	9.45E-05	1.02E-02
ENSRNOT00000049156.3:ncrna:chromosome:Rnor_6.0:MT	MT	2665	-2.17	2.11E-05	3.91E-03
ENSRNOT00000051008.3:ncrna:chromosome:Rnor_6.0:MT	MT	3695	-3.08	1.02E-07	6.85E-05
ENSRNOT00000044639.3:ncrna:chromosome:Rnor_6.0:MT	MT	15348	-3.5	4.17E-08	6.85E-05

Supplemental Table S6

Site Table sncRNA Prostate Strromal and Epithelial Overlaps 0.001

id	chr	start
piR-rno-220	3	120268801
piR-rno-2490	3	146945235
piR-rno-3102	20	6634461
piR-rno-3686	1	131021248
piR-rno-3988	6	122382919
piR-rno-4301	20	6660191
piR-rno-6777	20	6679528
piR-rno-7819	20	6623847
piR-rno-8940	20	6680121
piR-rno-10929	20	6652053
piR-rno-11689	20	6642860
piR-rno-13064	4	117651976
piR-rno-14184	20	6662987
piR-rno-14406	20	6669159
piR-rno-14617	20	6623914
piR-rno-14798	X	107054233
piR-rno-15619	20	6668525
piR-rno-15704	20	6672989
piR-rno-16346	20	6642863
piR-rno-16585	20	6680101
piR-rno-16818	20	6635475
piR-rno-19617	7	119885479
piR-rno-20568	20	6651884
piR-rno-21623	20	6632039
piR-rno-23823	20	6638130
piR-rno-27656	3	81482722
piR-rno-27796	15	245746
piR-rno-30402	5	91131795
piR-rno-34667	20	6671547
piR-rno-36179	20	6676627
piR-rno-41278	NA	NA
piR-rno-54090	20	6669070
piR-rno-55529	7	99564978
piR-rno-56843	6	122382920
piR-rno-61203	1	109521611

Supplemental Table S7
Site Table lncRNA Prostate Epithelial 0.001

test_id	Chr	Start	mRNA_size	ORF_size	log2.fold_change.	p_value	q_value	Gene	Category
TCONS_00000097	1	8126655	18935	327	-0.985	0.0001	1.72E-03	-	
TCONS_00000104	1	8255913	48739	453	-0.716	0.00005	9.26E-04	-	
TCONS_00000109	1	8774090	17523	405	-0.764	0.00015	2.46E-03	-	
TCONS_00001530	1	78112558	1279	135	0.985	0.00015	2.46E-03	AABR07002627.1	
TCONS_00003723	1	175995019	12898	672	-0.971	0.00005	9.26E-04	-	
TCONS_00005960	1	249591548	4727	276	-2.519	0.00005	9.26E-04	-	
TCONS_00005961	1	249596765	18434	324	-2.559	0.00005	9.26E-04	-	
TCONS_00005962	1	249645239	20247	288	-2.509	0.00005	9.26E-04	-	
TCONS_00005963	1	249695987	13730	264	-1.793	0.00005	9.26E-04	-	
TCONS_00005965	1	249735066	39387	402	-2.067	0.00005	9.26E-04	-	
TCONS_00006476	1	274168224	959	204	1.219	0.00005	9.26E-04	-	
TCONS_00006563	1	276895960	7303	294	0.73	0.0005	6.80E-03	-	
TCONS_00006920	1	14132251	27804	375	-0.977	0.00005	9.26E-04	-	
TCONS_00006935	1	14180975	16524	513	-0.945	0.00005	9.26E-04	-	
TCONS_00007020	1	20966066	23066	396	-0.861	0.0002	3.15E-03	-	
TCONS_00007021	1	20989996	18996	621	-0.794	0.00005	9.26E-04	-	
TCONS_00009855	1	162054045	20511	405	-0.751	0.00025	3.82E-03	-	
TCONS_00010873	1	198719161	2315	996	0.726	0.00095	1.14E-02	Sephs2	Metabolism
TCONS_00012575	1	249789640	24387	666	-1.757	0.00085	1.05E-02	AABR07006724.1	
TCONS_00012578	1	249789640	95933	864	-2.081	0.00005	9.26E-04	-	
TCONS_00012829	1	261854669	10541	405	-1.202	0.00005	9.26E-04	-	
TCONS_00012833	1	261854669	14019	351	-1.707	0.0001	1.72E-03	-	
TCONS_00013202	1	279623621	2652	306	1.477	0.0001	1.72E-03	-	
TCONS_00013806	1	23237904	33124	306	-1.431	0.00005	9.26E-04	-	
TCONS_00013989	1	31465283	2317	198	-1.095	0.00005	9.26E-04	-	
TCONS_00014677	1	62084377	629	78		0.00005	9.26E-04	-	
TCONS_00017668	1	249628639	8117	393	-2.412	0.00005	9.26E-04	-	
TCONS_00017669	1	249665572	29495	375	-1.921	0.00005	9.26E-04	-	
TCONS_00017670	1	249722776	10728	273	-1.248	0.0001	1.72E-03	-	
TCONS_00017762	1	254204088	2168	225	-1.221	0.00055	7.37E-03	-	
TCONS_00017895	1	262780800	583	72		0.0002	3.15E-03	-	
TCONS_00023730	10	56527084	1197	465	0.761	0.00005	9.26E-04	Eif5a	Translation
TCONS_00025682	10	101696383	10842	375	-2.451	0.00005	9.26E-04	-	
TCONS_00025683	10	101708032	3355	294	-2.272	0.00005	9.26E-04	-	
TCONS_00025957	10	107939842	13912	456	-3.29	0.00005	9.26E-04	-	
TCONS_00028247	10	107926884	3921	213	-2.691	0.00035	5.07E-03	-	
TCONS_00028466	11	16062031	72436	570	-1.481	0.00005	9.26E-04	Mir3588	
TCONS_00030661	11	57505004	31425	324	-1.223	0.00005	9.26E-04	-	
TCONS_00030911	11	69426747	25703	942	-2.338	0.00005	9.26E-04	-	
TCONS_00030912	11	69452557	12817	387	-2.378	0.00005	9.26E-04	-	
TCONS_00032673	11	58056659	18666	261	-0.763	0.00015	2.46E-03	-	
TCONS_00033925	12	15362330	21863	420	-1.09	0.00005	9.26E-04	-	
TCONS_00033927	12	15400657	18505	711	-1.294	0.00005	9.26E-04	-	
TCONS_00035672	12	15180762	14879	393	-1.394	0.00005	9.26E-04	-	
TCONS_00035673	12	15259395	14164	450	-1.517	0.00005	9.26E-04	-	
TCONS_00035675	12	15275102	44482	462	-1.274	0.00005	9.26E-04	-	
TCONS_00036032	12	28381981	37213	912	-2.328	0.00005	9.26E-04	Rn60_12_0305.1	
TCONS_00037142	12	15423659	2876	327	-1.163	0.00035	5.07E-03	-	
TCONS_00037143	12	15426684	14460	234	-0.838	0.00005	9.26E-04	-	
TCONS_00037352	12	30959997	1359	204	-1.537	0.00005	9.26E-04	-	

TCONS_00038987	13	96534691	35239	564	-0.836	0.00005	9.26E-04	-	
TCONS_00039674	13	50599107	27309	480	-1.544	0.00005	9.26E-04	-	
TCONS_00042412	13	96618810	13300	339	-0.898	0.0003	4.46E-03	-	
TCONS_00042525	13	97519370	17013	366	-0.839	0.00005	9.26E-04	-	
TCONS_00043898	14	54648921	53978	420	-1.566	0.00005	9.26E-04	-	
TCONS_00044457	14	87147950	7515	564	-1.211	0.00005	9.26E-04	-	
TCONS_00044564	14	101135664	2253	378	-1.25	0.00005	9.26E-04	-	
TCONS_00045419	14	31252929	14388	426	-1.142	0.00005	9.26E-04	-	
TCONS_00046098	14	79919716	26839	324	-1.605	0.0002	3.15E-03	-	
TCONS_00047033	14	13399769	8677	528	-1.198	0.00005	9.26E-04	-	
TCONS_00047173	14	21222284	793	99		0.00005	9.26E-04	-	
TCONS_00048726	14	115451889	6701	276	-3.086	0.00005	9.26E-04	-	
TCONS_00048755	15	2294539	43495	408	-1.097	0.00005	9.26E-04	-	
TCONS_00048980	15	15985366	40115	432	-0.644	0.0003	4.46E-03	-	
TCONS_00048981	15	16035004	18862	435	-0.895	0.00005	9.26E-04	-	
TCONS_00048984	15	16159303	38165	579	-0.781	0.00005	9.26E-04	-	
TCONS_00048986	15	16224034	2547	234	-1.154	0.00085	1.05E-02	-	
TCONS_00048987	15	16226726	6492	201	-0.907	0.00085	1.05E-02	-	
TCONS_00048991	15	16337989	41650	489	-1.002	0.00005	9.26E-04	-	
TCONS_00050618	15	16055517	40094	354	-0.854	0.0002	3.15E-03	-	
TCONS_00050621	15	16233519	49038	348	-0.751	0.00005	9.26E-04	-	
TCONS_00050918	15	30541238	7072	327	-1.128	0.00005	9.26E-04	-	
TCONS_00051909	15	102119617	1731	171	-2.234	0.00005	9.26E-04	-	
TCONS_00051910	15	102123213	2775	297	-2.166	0.0001	1.72E-03	-	
TCONS_00052109	15	2218730	15018	273	-1.101	0.00005	9.26E-04	-	
TCONS_00052114	15	2259144	35237	492	-1.085	0.00005	9.26E-04	-	
TCONS_00052397	15	16283172	9700	297	-0.864	0.00065	8.43E-03	-	
TCONS_00052538	15	17401165	5145	303	-1.389	0.00005	9.26E-04	-	
TCONS_00055305	16	65575959	19796	483	-1.034	0.00005	9.26E-04	-	
TCONS_00057028	16	366041	540	18		0.00005	9.26E-04	-	
TCONS_00058585	16	89060609	602	108		0.00005	9.26E-04	-	
TCONS_00059107	17	16958074	6337	285	-1.047	0.0001	1.72E-03	-	
TCONS_00060189	17	81633170	18346	294	-2.092	0.00025	3.82E-03	-	
TCONS_00062030	17	81633170	45251	492	-2.415	0.00005	9.26E-04	-	
TCONS_00063573	17	60572282	5526	402	-1.496	0.00005	9.26E-04	-	
TCONS_00064358	18	16107176	8910	549	-0.797	0.00045	6.24E-03	-	
TCONS_00066402	18	56957425	21792	486	-1.314	0.00065	8.43E-03	Mir143	
TCONS_00066718	18	75090475	98877	450	-1.67	0.00015	2.46E-03	-	
TCONS_00066721	18	75443433	7656	285	-1.243	0.0001	1.72E-03	-	
TCONS_00066948	18	3095598	3967	189	-1.485	0.00065	8.43E-03	-	
TCONS_00067197	18	16981547	567	93		0.00005	9.26E-04	-	
TCONS_00067543	18	32633113	5971	225	-0.945	0.00005	9.26E-04	-	
TCONS_00067544	18	32639353	1570	255	-1.154	0.00055	7.37E-03	-	
TCONS_00067547	18	32645002	8801	417	-0.691	0.00045	6.24E-03	-	
TCONS_00068076	18	55255692	661	78		0.00005	9.26E-04	-	
TCONS_00068567	18	87128831	1446	279	-1.109	0.00085	1.05E-02	-	
TCONS_00068955	19	23542467	42803	606	-1.03	0.00015	2.46E-03	-	
TCONS_00069177	19	31615785	5178	252	-1.006	0.00005	9.26E-04	uc_338	
TCONS_00069597	19	47159236	115423	516	-0.725	0.00005	9.26E-04	-	
TCONS_00069603	19	47313675	52382	486	-0.684	0.0001	1.72E-03	-	
TCONS_00069606	19	47411063	9976	360	-0.957	0.00025	3.82E-03	-	
TCONS_00069607	19	47421312	50570	618	-0.733	0.00005	9.26E-04	-	
TCONS_00070929	19	47143859	10152	378	-0.856	0.00005	9.26E-04	-	

TCONS_00071909	19	47155012	4141	330	-1.321	0.00005	9.26E-04	-	
TCONS_00071911	19	47373166	7495	363	-0.865	0.0005	6.80E-03	-	
TCONS_00071938	19	47558544	2543	228	-2.221	0.00075	9.47E-03	-	
TCONS_00072378	2	123551	4978	285	-0.88	0.0001	1.72E-03	-	
TCONS_00073301	2	73232062	7773	360	-1.436	0.00005	9.26E-04	-	
TCONS_00073349	2	79067914	54298	369	-1.77	0.00005	9.26E-04	AABR07008916.1	
TCONS_00073420	2	88734941	6595	348	-1.533	0.00005	9.26E-04	-	
TCONS_00074148	2	150879847	3405	213	-0.684	0.0002	3.15E-03	-	
TCONS_00076924	2	39135042	10052	351	-0.664	0.0003	4.46E-03	-	
TCONS_00077952	2	153743389	6691	249	-0.957	0.0003	4.46E-03	-	
TCONS_00082071	2	103149264	5174	294	-1.095	0.00015	2.46E-03	-	
TCONS_00084182	2	211762403	3839	198	-0.887	0.00035	5.07E-03	-	
TCONS_00086062	20	18688024	6833	216	-0.927	0.00025	3.82E-03	-	
TCONS_00086065	20	18738902	14112	1059	-1.175	0.00005	9.26E-04	-	
TCONS_00086066	20	18753079	11326	645	-1.062	0.0009	1.10E-02	-	
TCONS_00086633	20	50439739	4382	531	-0.837	0.00095	1.14E-02	Bves	
TCONS_00090340	3	38392701	28689	414	-1.83	0.00005	9.26E-04	-	
TCONS_00090349	3	38392701	49324	378	-1.712	0.00005	9.26E-04	-	
TCONS_00093005	3	166467656	66872	582	-1.801	0.00005	9.26E-04	-	
TCONS_00094627	3	60110393	5720	519	-0.93	0.00005	9.26E-04	-	
TCONS_00096278	3	139333856	51531	447	-2.046	0.00005	9.26E-04	-	
TCONS_00096918	3	161180132	8924	303	2.441	0.00005	9.26E-04	-	
TCONS_00097078	3	165360737	2114	330	-2.245	0.00065	8.43E-03	-	
TCONS_00097079	3	165365024	9676	528	-2.033	0.00005	9.26E-04	-	
TCONS_00097213	3	175763331	5806	354	-1.283	0.00045	6.24E-03	Mir1b	
TCONS_00098084	3	60106633	3212	330	-1.095	0.00035	5.07E-03	-	
TCONS_00098794	3	107381335	1527	147	-1.973	0.00065	8.43E-03	-	
TCONS_00099751	3	144080680	1190	234	-1.156	0.00005	9.26E-04	-	
TCONS_00100113	3	161190694	984	234	1.28	0.0007	8.95E-03	-	
TCONS_00100514	3	175775624	3045	228	-2.193	0.0006	7.89E-03	-	
TCONS_00103433	4	168527521	11300	432	-0.723	0.0004	5.66E-03	-	
TCONS_00103434	4	168539534	10532	480	-0.85	0.00005	9.26E-04	-	
TCONS_00103517	4	171595598	13375	354	-1.36	0.00005	9.26E-04	-	
TCONS_00103765	4	182620091	4890	486	-0.764	0.00095	1.14E-02	-	
TCONS_00104577	4	66957436	4332	294	-0.925	0.00045	6.24E-03	-	
TCONS_00104966	4	90923522	2508	270	-1.306	0.0003	4.46E-03	-	
TCONS_00105564	4	125684691	160392	495	-0.935	0.00005	9.26E-04	-	
TCONS_00105927	4	150635805	77082	687	-2.733	0.00005	9.26E-04	-	
TCONS_00105928	4	150635805	46413	678	-2.642	0.00005	9.26E-04	-	
TCONS_00106422	4	168072953	4757	228	-1.218	0.00035	5.07E-03	-	
TCONS_00106491	4	171614193	19991	495	-0.72	0.00035	5.07E-03	-	
TCONS_00106492	4	171634259	11204	294	-0.838	0.0001	1.72E-03	-	
TCONS_00107493	4	35002322	700	90		0.00005	9.26E-04	-	
TCONS_00107498	4	35015107	920	147		0.00005	9.26E-04	-	
TCONS_00107513	4	35046517	793	192		0.00005	9.26E-04	-	
TCONS_00109686	4	159670366	1657	225	-0.896	0.00005	9.26E-04	-	
TCONS_00110713	5	40318425	4874	411	-0.86	0.00005	9.26E-04	-	
TCONS_00110742	5	47465739	42927	342	-1.136	0.0005	6.80E-03	-	
TCONS_00111201	5	71682087	60432	549	-0.591	0.00055	7.37E-03	-	
TCONS_00116678	5	162804976	2276	219	1.329	0.0004	5.66E-03	-	
TCONS_00116878	5	166934541	17141	519	-0.763	0.00005	9.26E-04	-	
TCONS_00120432	6	4255516	40312	399	-1.763	0.00005	9.26E-04	AABR07062708.1	
TCONS_00120542	6	9711793	24250	516	-0.889	0.00005	9.26E-04	-	

TCONS_00121897	6	103364181	39015	348	-1.318	0.00015	2.46E-03	AC118496.1	
TCONS_00122514	6	133658880	5400	372	-1.04	0.00005	9.26E-04	AABR07065531.1	
TCONS_00122519	6	133667221	24841	390	-1.451	0.00005	9.26E-04	AABR07065531.5	
TCONS_00123074	6	6584464	12059	255	-0.718	0.0004	5.66E-03	-	
TCONS_00124428	6	104410955	970	219	0.966	0.0005	6.80E-03	-	
TCONS_00126199	6	30528636	954	207	1.518	0.0004	5.66E-03	-	
TCONS_00126475	6	43193065	2368	222	-2.468	0.00065	8.43E-03	-	
TCONS_00129503	7	42570940	21509	399	-1.314	0.00025	3.82E-03	-	
TCONS_00129504	7	42592577	8013	264	-1.297	0.00005	9.26E-04	-	
TCONS_00130142	7	98059904	3804	282	-0.87	0.00005	9.26E-04	-	
TCONS_00131723	7	3051684	1103	348	0.61	0.00015	2.46E-03	-	
TCONS_00133328	7	77456799	7606	258	2.119	0.00005	9.26E-04	-	
TCONS_00133701	7	114851069	18280	429	-0.854	0.0003	4.46E-03	-	
TCONS_00135033	7	412395	873	240	-1.302	0.00005	9.26E-04	-	
TCONS_00135094	7	1453382	752	84	-0.964	0.0003	4.46E-03	-	
TCONS_00135847	7	42600680	1249	117	-1.531	0.00055	7.37E-03	-	
TCONS_00135853	7	42617467	2548	240	-1.753	0.0001	1.72E-03	-	
TCONS_00135889	7	42710730	2425	186	-1.669	0.00075	9.47E-03	-	
TCONS_00135890	7	42713344	4832	195	-1.259	0.00015	2.46E-03	-	
TCONS_00136983	7	92497283	1949	279	-1.139	0.00005	9.26E-04	-	
TCONS_00137269	7	102932754	469	45		0.0004	5.66E-03	-	
TCONS_00138040	8	4292229	5956	570	-0.66	0.00045	6.24E-03	-	
TCONS_00138121	8	13037264	10110	309	-2.811	0.0001	1.72E-03	-	
TCONS_00138742	8	44354220	10763	405	-2.288	0.00005	9.26E-04	-	
TCONS_00138772	8	45753253	14593	237	-1.457	0.00005	9.26E-04	Mir3596a	
TCONS_00138773	8	45768032	20225	528	-1.828	0.00005	9.26E-04	-	
TCONS_00139337	8	64811940	32314	429	-2.232	0.00005	9.26E-04	-	
TCONS_00139923	8	85186549	36463	438	-0.84	0.00005	9.26E-04	-	
TCONS_00141837	8	44381195	7946	273	-2.061	0.00005	9.26E-04	-	
TCONS_00141838	8	44389523	9962	330	-1.679	0.00005	9.26E-04	-	
TCONS_00142489	8	64811940	24227	486	-2.201	0.00055	7.37E-03	Rn60_8_0651.1	
TCONS_00142735	8	73195276	61117	516	-0.741	0.00025	3.82E-03	-	
TCONS_00142874	8	81797817	2405	225	1.119	0.00065	8.43E-03	-	
TCONS_00144892	8	44350342	3588	267	-2.146	0.00025	3.82E-03	-	
TCONS_00145836	8	85256907	2317	204	-1.155	0.00015	2.46E-03	-	
TCONS_00149700	9	27925341	13514	426	-2.328	0.00005	9.26E-04	-	
TCONS_00149701	9	27943951	35453	477	-2.112	0.00005	9.26E-04	-	
TCONS_00149702	9	27943951	19746	387	-2.051	0.00005	9.26E-04	-	
TCONS_00149703	9	28005854	12540	369	-1.803	0.00005	9.26E-04	-	
TCONS_00149704	9	28018578	28068	294	-2.19	0.00005	9.26E-04	-	
TCONS_00149705	9	28046888	79518	570	-2.159	0.00005	9.26E-04	-	
TCONS_00149707	9	28046888	25120	468	-2.165	0.00005	9.26E-04	-	
TCONS_00149828	9	40512446	1756	222	-2.039	0.00005	9.26E-04	-	
TCONS_00149837	9	40512446	2978	339	-2.215	0.00005	9.26E-04	-	
TCONS_00150219	9	62015613	43917	678	-1.601	0.00055	7.37E-03	-	
TCONS_00150404	9	72573005	2501	207	-0.807	0.0001	1.72E-03	-	
TCONS_00151721	9	27916744	8490	417	-1.668	0.00005	9.26E-04	-	
TCONS_00153827	X	13804971	2304	417	-1.259	0.00055	7.37E-03	-	
TCONS_00153830	X	13815921	14331	507	-1.593	0.00005	9.26E-04	-	
TCONS_00154183	X	26675170	20874	273	-0.973	0.00005	9.26E-04	-	
TCONS_00154297	X	34313311	15948	735	-1.938	0.00005	9.26E-04	-	
TCONS_00155926	X	14644275	7343	354	-1.283	0.00005	9.26E-04	-	
TCONS_00156113	X	26696434	11083	261	-1.136	0.00005	9.26E-04	-	

TCONS_00156114	X	26707992	60343	564	-0.828	0.00005	9.26E-04	-	
TCONS_00157666	X	3258006	10062	270	-0.904	0.00065	8.43E-03	-	
TCONS_00158248	X	26592619	28218	399	-0.825	0.00005	9.26E-04	-	
TCONS_00158319	X	32910305	938	165	-2.826	0.0005	6.80E-03	-	
TCONS_00158350	X	34329793	7684	354	-1.777	0.00005	9.26E-04	-	
TCONS_00160334	X	156075167	6124	639	-1.708	0.00005	9.26E-04	-	

Supplemental Table S8
Site Table lncRNA Prostate Stromal (p<0.001)

test_id	Chr	Start	mRNA_size	ORF_size	log2.fold_change.	p_value	q_value	Gene	Category
TCONS_00000047	1	3587953	12978	417	-1.249	0.00005	9.26E-04	-	
TCONS_00000097	1	8126655	18935	327	-0.988	0.0001	1.72E-03	-	
TCONS_00000104	1	8255913	48739	453	-0.793	0.00005	9.26E-04	-	
TCONS_00000223	1	14161063	7776	273	-1.46	0.00005	9.26E-04	-	
TCONS_00001754	1	81947578	1752	348	-1.193	0.00005	9.26E-04	-	
TCONS_00002846	1	130032606	18105	495	-1.031	0.00005	9.26E-04	-	
TCONS_00005960	1	249591548	4727	276	-1.421	0.00005	9.26E-04	-	
TCONS_00005961	1	249596765	18434	324	-1.276	0.00005	9.26E-04	-	
TCONS_00005962	1	249645239	20247	288	-1.156	0.00005	9.26E-04	-	
TCONS_00005963	1	249695987	13730	264	-1	0.00005	9.26E-04	-	
TCONS_00005965	1	249735066	39387	402	-0.991	0.00005	9.26E-04	-	
TCONS_00006754	1	3170237	14471	318	-0.994	0.00005	9.26E-04	-	
TCONS_00006755	1	3255211	18084	744	-0.92	0.00005	9.26E-04	-	
TCONS_00006756	1	3316977	31542	546	-1.015	0.00005	9.26E-04	-	
TCONS_00006759	1	3622310	5355	285	-1.348	0.00005	9.26E-04	-	
TCONS_00006760	1	3627977	18966	342	-0.942	0.00005	9.26E-04	-	
TCONS_00006762	1	3668722	11002	399	-0.958	0.00005	9.26E-04	-	
TCONS_00006828	1	8232208	9050	486	-1.055	0.00005	9.26E-04	-	
TCONS_00006829	1	8241669	4228	414	-1.628	0.00005	9.26E-04	-	
TCONS_00006920	1	14132251	27804	375	-1.178	0.00005	9.26E-04	-	
TCONS_00006935	1	14180975	16524	513	-1.263	0.00015	2.46E-03	-	
TCONS_00007020	1	20966066	23066	396	-0.805	0.00005	9.26E-04	-	
TCONS_00007021	1	20989996	18996	621	-0.761	0.00005	9.26E-04	-	
TCONS_00007027	1	21054482	13222	420	-0.571	0.00075	9.47E-03	-	
TCONS_00011365	1	215744373	2376	261	0.646	0.0009	1.10E-02	Mir675	
TCONS_00011931	1	221177114	5204	255	-1.477	0.00005	9.26E-04	-	
TCONS_00012571	1	249564179	8180	459	-0.91	0.0001	1.72E-03	-	
TCONS_00012578	1	249789640	95933	864	-0.927	0.00015	2.46E-03	-	
TCONS_00012833	1	261854669	14019	351	-1.117	0.0001	1.72E-03	-	
TCONS_00013106	1	276098598	31529	372	-0.897	0.00005	9.26E-04	-	
TCONS_00013127	1	276575086	24344	456	-0.637	0.0007	8.95E-03	-	
TCONS_00013329	1	3053887	6282	255	-1.2	0.00005	9.26E-04	-	
TCONS_00013339	1	3075204	678	249		0.00005	9.26E-04	-	
TCONS_00013342	1	3077782	4158	192	-1.264	0.0004	5.66E-03	-	
TCONS_00013349	1	3099614	5966	270	-1.313	0.0001	1.72E-03	-	
TCONS_00013357	1	3116495	12314	405	-0.995	0.00005	9.26E-04	-	
TCONS_00013362	1	3142003	3614	309	-1.544	0.00005	9.26E-04	-	
TCONS_00013368	1	3154719	14566	342	-0.907	0.0002	3.15E-03	-	
TCONS_00013371	1	3192333	35811	375	-0.666	0.00045	6.24E-03	-	
TCONS_00013372	1	3228229	3930	285	-1.525	0.00005	9.26E-04	-	
TCONS_00013373	1	3232230	22878	345	-0.74	0.0002	3.15E-03	-	
TCONS_00013374	1	3274961	8798	231	-0.881	0.00055	7.37E-03	-	
TCONS_00013376	1	3299260	8646	300	-0.889	0.0002	3.15E-03	-	
TCONS_00013377	1	3310148	2851	231	-1.202	0.00045	6.24E-03	-	
TCONS_00013378	1	3313153	3764	372	-1.303	0.00005	9.26E-04	-	
TCONS_00013417	1	3484509	3173	234	-1.25	0.0007	8.95E-03	-	
TCONS_00013441	1	3545446	4207	234	-1.071	0.0003	4.46E-03	-	
TCONS_00013444	1	3562441	3212	216	-1.274	0.0003	4.46E-03	-	
TCONS_00013447	1	3571432	3001	213	-1.588	0.0001	1.72E-03	-	

TCONS_00013449	1	3574890	2772	180	-1.43	0.0002	3.15E-03	-	
TCONS_00013450	1	3577971	2400	183	-1.618	0.0004	5.66E-03	-	
TCONS_00013451	1	3580434	7439	297	-1.017	0.00005	9.26E-04	-	
TCONS_00013452	1	3601454	7060	249	-1.265	0.00005	9.26E-04	-	
TCONS_00013463	1	3679857	3097	147	-1.288	0.00055	7.37E-03	-	
TCONS_00013561	1	8229651	2411	198	-1.424	0.00005	9.26E-04	-	
TCONS_00013761	1	21887063	2061	294	-1.885	0.0008	9.98E-03	-	
TCONS_00015663	1	130142041	640	84		0.00005	9.26E-04	-	
TCONS_00015982	1	146742910	572	93		0.00005	9.26E-04	-	
TCONS_00016088	1	155618861	6846	420	-0.973	0.0005	6.80E-03	-	
TCONS_00016089	1	155626242	29961	282	-0.989	0.00005	9.26E-04	-	
TCONS_00016094	1	155693063	2772	168	-1.684	0.0004	5.66E-03	-	
TCONS_00016240	1	163745774	604	75		0.00005	9.26E-04	-	
TCONS_00017668	1	249628639	8117	393	-1.372	0.00005	9.26E-04	-	
TCONS_00017669	1	249665572	29495	375	-1.109	0.00005	9.26E-04	-	
TCONS_00017670	1	249722776	10728	273	-0.682	0.0005	6.80E-03	-	
TCONS_00021686	10	101604294	17737	483	-1.617	0.00005	9.26E-04	-	
TCONS_00021689	10	101696383	11501	630	-1.922	0.00035	5.07E-03	-	
TCONS_00025209	10	90153200	19876	405	-1.082	0.00005	9.26E-04	-	
TCONS_00025259	10	90769554	2679	435	-2.805	0.00005	9.26E-04	AABR07030501.1	
TCONS_00025260	10	90773658	2272	177	-1.268	0.0008	9.98E-03	-	
TCONS_00025677	10	101595358	4892	369	-1.91	0.00005	9.26E-04	-	
TCONS_00025678	10	101623862	62673	618	-1.705	0.00005	9.26E-04	-	
TCONS_00025679	10	101687933	5729	378	-1.828	0.00005	9.26E-04	-	
TCONS_00025682	10	101696383	10842	375	-1.476	0.00005	9.26E-04	-	
TCONS_00025683	10	101708032	3355	294	-1.038	0.00005	9.26E-04	-	
TCONS_00025955	10	107515532	11552	399	-2.944	0.00005	9.26E-04	-	
TCONS_00025956	10	107515532	5317	276	-3.001	0.00005	9.26E-04	-	
TCONS_00025957	10	107939842	13912	456	-2.427	0.00005	9.26E-04	-	
TCONS_00027174	10	48873181	580	126		0.00005	9.26E-04	-	
TCONS_00028099	10	101588731	3791	357	-1.575	0.00045	6.24E-03	-	
TCONS_00028101	10	101600496	3644	441	-1.877	0.00005	9.26E-04	-	
TCONS_00028246	10	107919555	6385	345	-2.603	0.00005	9.26E-04	-	
TCONS_00028247	10	107926884	3921	213	-3.128	0.00005	9.26E-04	-	
TCONS_00028249	10	107935089	2795	240	-2.221	0.00005	9.26E-04	-	
TCONS_00028591	11	31523886	9383	339	-0.84	0.0003	4.46E-03	-	
TCONS_00030695	11	60961711	2448	153	-1.619	0.0001	1.72E-03	-	
TCONS_00030911	11	69426747	25703	942	-1.329	0.00005	9.26E-04	-	
TCONS_00030912	11	69452557	12817	387	-1.499	0.00005	9.26E-04	-	
TCONS_00031647	11	12792816	658	147		0.00005	9.26E-04	-	
TCONS_00032188	11	32317755	2926	300	-1.609	0.0001	1.72E-03	-	
TCONS_00032198	11	32337306	6755	300	-1.411	0.00005	9.26E-04	-	
TCONS_00032211	11	32367372	4620	531	-1.956	0.00005	9.26E-04	-	
TCONS_00032735	11	59344103	598	135		0.00005	9.26E-04	-	
TCONS_00032820	11	60948838	556	93		0.00005	9.26E-04	-	
TCONS_00032827	11	60964299	2225	162	-1.584	0.0004	5.66E-03	-	
TCONS_00033925	12	15362330	21863	420	-0.758	0.0006	7.89E-03	-	
TCONS_00033927	12	15400657	18505	711	-0.831	0.00005	9.26E-04	-	
TCONS_00034366	12	29251656	5483	309	-1.102	0.00015	2.46E-03	-	
TCONS_00034785	12	43585245	5068	285	-1.02	0.00005	9.26E-04	-	
TCONS_00035673	12	15259395	14164	450	-0.932	0.00005	9.26E-04	-	
TCONS_00036032	12	28381981	37213	912	-1.298	0.00005	9.26E-04	Rn60_12_0305.1	
TCONS_00036443	12	43590676	12324	315	-0.909	0.00005	9.26E-04	-	

TCONS_00036444	12	43604705	8692	234	-0.896	0.00005	9.26E-04	-	
TCONS_00037136	12	15359153	2770	408	-0.94	0.00005	9.26E-04	-	
TCONS_00037143	12	15426684	14460	234	-0.61	0.00055	7.37E-03	-	
TCONS_00037328	12	29258634	2826	252	-1.415	0.00075	9.47E-03	-	
TCONS_00038987	13	96534691	35239	564	-0.781	0.00005	9.26E-04	-	
TCONS_00038989	13	96600561	14313	345	-0.746	0.0009	1.10E-02	-	
TCONS_00039674	13	50599107	27309	480	-0.8	0.0001	1.72E-03	-	
TCONS_00040321	13	83220252	18495	336	-1.028	0.0002	3.15E-03	-	
TCONS_00040647	13	96570192	30070	435	-0.828	0.0001	1.72E-03	-	
TCONS_00041524	13	43037730	4612	609	-1.45	0.0004	5.66E-03	-	
TCONS_00042411	13	96497261	37282	393	-0.765	0.00005	9.26E-04	-	
TCONS_00042412	13	96618810	13300	339	-0.924	0.00015	2.46E-03	-	
TCONS_00042413	13	96632166	6889	186	-0.976	0.0002	3.15E-03	-	
TCONS_00042415	13	96647161	9293	441	-0.93	0.0003	4.46E-03	-	
TCONS_00042936	13	113673075	506	72		0.00005	9.26E-04	-	
TCONS_00044564	14	101135664	2253	378	-1.092	0.00005	9.26E-04	-	
TCONS_00047657	14	59815944	549	36		0.0006	7.89E-03	-	
TCONS_00047802	14	61495690	4285	213	-1.419	0.00055	7.37E-03	-	
TCONS_00048750	15	2034397	25001	432	-1.349	0.00005	9.26E-04	-	
TCONS_00048751	15	2136151	7485	249	-1.087	0.00015	2.46E-03	-	
TCONS_00048752	15	2185121	12237	375	-1.067	0.00005	9.26E-04	-	
TCONS_00048754	15	2247245	11831	339	-1.12	0.00005	9.26E-04	-	
TCONS_00048755	15	2294539	43495	408	-1.15	0.00005	9.26E-04	-	
TCONS_00048980	15	15985366	40115	432	-0.615	0.0005	6.80E-03	-	
TCONS_00048981	15	16035004	18862	435	-0.746	0.00005	9.26E-04	-	
TCONS_00048984	15	16159303	38165	579	-0.736	0.00005	9.26E-04	-	
TCONS_00048987	15	16226726	6492	201	-0.957	0.00005	9.26E-04	-	
TCONS_00048990	15	16299385	13265	249	-0.906	0.00005	9.26E-04	-	
TCONS_00048991	15	16337989	41650	489	-0.845	0.00005	9.26E-04	-	
TCONS_00050273	15	106428599	5396	423	-1.012	0.0007	8.95E-03	-	
TCONS_00050325	15	344359	44256	375	-1.428	0.00005	9.26E-04	U6	
TCONS_00050332	15	2143726	35079	531	-0.974	0.00005	9.26E-04	-	
TCONS_00050618	15	16055517	40094	354	-0.727	0.00065	8.43E-03	-	
TCONS_00050621	15	16233519	49038	348	-0.835	0.00005	9.26E-04	-	
TCONS_00050622	15	16312721	19918	294	-0.699	0.0002	3.15E-03	-	
TCONS_00050623	15	16332697	4974	357	-1.046	0.00005	9.26E-04	-	
TCONS_00051962	15	106679271	36629	348	-0.679	0.0003	4.46E-03	-	
TCONS_00052098	15	2059448	4037	207	-1.282	0.00075	9.47E-03	-	
TCONS_00052101	15	2077751	17357	282	-1.255	0.00005	9.26E-04	-	
TCONS_00052103	15	2105398	20273	282	-1.096	0.00005	9.26E-04	-	
TCONS_00052104	15	2125728	9248	555	-1.356	0.00005	9.26E-04	-	
TCONS_00052108	15	2209564	9082	324	-0.932	0.00055	7.37E-03	-	
TCONS_00052109	15	2218730	15018	273	-1.179	0.00005	9.26E-04	-	
TCONS_00052114	15	2259144	35237	492	-1.025	0.00005	9.26E-04	-	
TCONS_00052397	15	16283172	9700	297	-0.8	0.00015	2.46E-03	-	
TCONS_00052398	15	16294935	3345	240	-1.18	0.0001	1.72E-03	-	
TCONS_00052400	15	16379758	27166	300	-0.674	0.0003	4.46E-03	-	
TCONS_00052417	15	16443872	5597	231	-1.12	0.0001	1.72E-03	-	
TCONS_00052427	15	16466301	3567	264	-1.288	0.00025	3.82E-03	-	
TCONS_00052437	15	16487739	2935	120	-1.278	0.0008	9.98E-03	-	
TCONS_00057346	16	20471466	10347	573	-1.168	0.00005	9.26E-04	-	
TCONS_00057603	16	36450175	858	201	-1.057	0.00005	9.26E-04	-	
TCONS_00062001	17	80327861	4776	381	-1.432	0.00005	9.26E-04	-	

TCONS_00062002	17	80342001	2456	195	-1.57	0.00005	9.26E-04	-	
TCONS_00062003	17	80344523	18622	330	-1.329	0.00005	9.26E-04	-	
TCONS_00062004	17	80372233	12019	315	-1.223	0.00005	9.26E-04	-	
TCONS_00062030	17	81633170	45251	492	-1.117	0.0004	5.66E-03	-	
TCONS_00062412	17	6566077	4466	408	-1.32	0.0001	1.72E-03	-	
TCONS_00062779	17	19120143	4915	297	-1.144	0.00005	9.26E-04	-	
TCONS_00062797	17	22265797	27900	591	-0.776	0.00035	5.07E-03	-	
TCONS_00063022	17	36943674	3102	240	-1.175	0.0008	9.98E-03	-	
TCONS_00063667	17	66062646	461	21		0.0006	7.89E-03	-	
TCONS_00063910	17	78013543	6770	483	-1.196	0.0001	1.72E-03	-	
TCONS_00063921	17	78040824	2998	300	-1.626	0.00045	6.24E-03	-	
TCONS_00063927	17	78056421	5354	207	-1.076	0.00065	8.43E-03	-	
TCONS_00063931	17	78069790	6441	342	-1.209	0.0005	6.80E-03	-	
TCONS_00063977	17	80332736	7186	387	-1.14	0.00005	9.26E-04	-	
TCONS_00066314	18	52689366	9224	324	-0.914	0.00055	7.37E-03	-	
TCONS_00066402	18	56957425	21792	486	-1.037	0.00005	9.26E-04	Mir143	
TCONS_00066726	18	76653223	8032	594	-0.821	0.0006	7.89E-03	-	
TCONS_00067195	18	16968572	5282	252	-1.103	0.00005	9.26E-04	-	
TCONS_00067196	18	16973961	3657	282	-0.95	0.0004	5.66E-03	-	
TCONS_00068281	18	61982507	3399	321	-1.398	0.00065	8.43E-03	-	
TCONS_00069596	19	47082273	61310	615	-0.936	0.00015	2.46E-03	-	
TCONS_00069597	19	47159236	115423	516	-0.928	0.00005	9.26E-04	-	
TCONS_00069599	19	47279272	16275	450	-0.85	0.0005	6.80E-03	-	
TCONS_00069601	19	47306274	4969	453	-0.912	0.0007	8.95E-03	-	
TCONS_00069603	19	47313675	52382	486	-0.93	0.00005	9.26E-04	-	
TCONS_00069604	19	47366559	6199	369	-1.279	0.00005	9.26E-04	-	
TCONS_00069606	19	47411063	9976	360	-1.019	0.00005	9.26E-04	-	
TCONS_00069607	19	47421312	50570	618	-0.878	0.00005	9.26E-04	-	
TCONS_00069614	19	49249926	11954	315	-0.846	0.0003	4.46E-03	-	
TCONS_00070400	19	25774142	1473	303	0.602	0.00035	5.07E-03	AC120246.2	
TCONS_00070730	19	40408425	14471	357	-0.957	0.00005	9.26E-04	-	
TCONS_00070731	19	40423412	28237	306	-0.772	0.00005	9.26E-04	-	
TCONS_00070929	19	47143859	10152	378	-0.936	0.00005	9.26E-04	-	
TCONS_00071634	19	32047934	6625	312	-1.7	0.00005	9.26E-04	-	
TCONS_00071635	19	32057417	2651	156	-2.615	0.00075	9.47E-03	-	
TCONS_00071636	19	32060963	7336	465	-1.934	0.00005	9.26E-04	-	
TCONS_00071908	19	47071818	10064	291	-0.934	0.00005	9.26E-04	-	
TCONS_00071911	19	47373166	7495	363	-1.257	0.00005	9.26E-04	-	
TCONS_00071915	19	47394238	3640	243	-1.348	0.00005	9.26E-04	-	
TCONS_00071916	19	47398093	9387	393	-1.25	0.00005	9.26E-04	-	
TCONS_00071919	19	47483896	15262	462	-1.143	0.00005	9.26E-04	-	
TCONS_00071924	19	47512002	9522	405	-1.03	0.00005	9.26E-04	-	
TCONS_00071925	19	47521592	3833	282	-1.287	0.00055	7.37E-03	-	
TCONS_00076924	2	39135042	10052	351	-0.759	0.00005	9.26E-04	-	
TCONS_00079779	2	242954128	4605	273	-1.985	0.00005	9.26E-04	-	
TCONS_00080582	2	13546549	1357	132	-1.396	0.00005	9.26E-04	-	
TCONS_00080922	2	33870358	493	51		0.0001	1.72E-03	-	
TCONS_00082260	2	110470653	4324	243	-1.061	0.0008	9.98E-03	-	
TCONS_00083214	2	155586709	7051	399	-1.046	0.00045	6.24E-03	-	
TCONS_00083231	2	155620619	3717	297	-1.877	0.0004	5.66E-03	-	
TCONS_00083235	2	155628719	9654	294	-1.524	0.00005	9.26E-04	-	
TCONS_00083236	2	155638881	2744	228	-1.467	0.0006	7.89E-03	-	
TCONS_00083266	2	155680959	4398	261	-1.421	0.00055	7.37E-03	-	

TCONS_00083681	2	184309976	3031	192	-1.302	0.00055	7.37E-03	-	
TCONS_00083838	2	193541171	468	87		0.0002	3.15E-03	-	
TCONS_00083945	2	197939916	1518	204	-0.656	0.00065	8.43E-03	-	
TCONS_00084590	2	239325332	524	75		0.00005	9.26E-04	-	
TCONS_00084638	2	242930076	3410	306	-2.261	0.0001	1.72E-03	-	
TCONS_00084644	2	242940054	563	69		0.00005	9.26E-04	-	
TCONS_00084647	2	242949566	3523	171	-1.607	0.00085	1.05E-02	-	
TCONS_00084656	2	242976396	6990	414	-1.333	0.00005	9.26E-04	-	
TCONS_00084657	2	242983908	2233	144	-1.245	0.0005	6.80E-03	-	
TCONS_00086062	20	18688024	6833	216	-0.809	0.0001	1.72E-03	-	
TCONS_00086065	20	18738902	14112	1059	-0.841	0.00005	9.26E-04	-	
TCONS_00086066	20	18753079	11326	645	-0.772	0.0007	8.95E-03	-	
TCONS_00088710	20	26214326	3173	216	-1.47	0.00035	5.07E-03	-	
TCONS_00089908	3	10705386	5160	291	-1.131	0.00095	1.14E-02	-	
TCONS_00092768	3	158600785	5547	300	-1.119	0.00005	9.26E-04	-	
TCONS_00092769	3	158607066	14301	309	-0.796	0.0003	4.46E-03	-	
TCONS_00092921	3	162285259	24535	537	-0.973	0.0004	5.66E-03	-	
TCONS_00093005	3	166467656	66872	582	-0.929	0.0002	3.15E-03	-	
TCONS_00094099	3	21953114	2985	198	-1.193	0.0002	3.15E-03	-	
TCONS_00096278	3	139333856	51531	447	-0.964	0.0002	3.15E-03	-	
TCONS_00097078	3	165360737	2114	330	-1.704	0.00005	9.26E-04	-	
TCONS_00097079	3	165365024	9676	528	-1.328	0.00005	9.26E-04	-	
TCONS_00097213	3	175763331	5806	354	-1.174	0.00005	9.26E-04	Mir1b	
TCONS_00100042	3	158462506	4184	438	-1.232	0.00055	7.37E-03	-	
TCONS_00100048	3	158476564	6827	438	-0.978	0.00045	6.24E-03	-	
TCONS_00100051	3	158486557	7142	270	-1.196	0.0001	1.72E-03	-	
TCONS_00100072	3	158585562	6544	243	-0.884	0.00065	8.43E-03	-	
TCONS_00100197	3	165363063	1732	300	-1.7	0.00005	9.26E-04	-	
TCONS_00100455	3	174997178	791	168		0.00005	9.26E-04	-	
TCONS_00100513	3	175773797	1551	447	-1.726	0.00085	1.05E-02	-	
TCONS_00100514	3	175775624	3045	228	-1.682	0.00005	9.26E-04	-	
TCONS_00100516	3	175789571	4526	261	-2.183	0.00005	9.26E-04	-	
TCONS_00101393	4	58340153	33512	399	-1.171	0.00035	5.07E-03	-	
TCONS_00101398	4	58631346	8794	270	-0.92	0.0006	7.89E-03	-	
TCONS_00102703	4	140623983	5316	246	-1.389	0.00005	9.26E-04	-	
TCONS_00102882	4	146374809	8132	375	-1.353	0.00005	9.26E-04	-	
TCONS_00103030	4	152026384	16545	627	-0.994	0.00005	9.26E-04	-	
TCONS_00103433	4	168527521	11300	432	-0.788	0.00005	9.26E-04	-	
TCONS_00103434	4	168539534	10532	480	-0.808	0.00005	9.26E-04	-	
TCONS_00103517	4	171595598	13375	354	-0.878	0.00005	9.26E-04	-	
TCONS_00103518	4	171609077	4832	264	-1.24	0.00005	9.26E-04	-	
TCONS_00105564	4	125684691	160392	495	-0.942	0.00025	3.82E-03	-	
TCONS_00106489	4	171591955	3545	243	-1.306	0.0002	3.15E-03	-	
TCONS_00106491	4	171614193	19991	495	-0.832	0.00005	9.26E-04	-	
TCONS_00106492	4	171634259	11204	294	-0.769	0.0001	1.72E-03	-	
TCONS_00107906	4	58644743	8474	441	-1.027	0.00005	9.26E-04	-	
TCONS_00107907	4	58653486	7065	261	-1.043	0.00035	5.07E-03	-	
TCONS_00109024	4	128224313	3780	183	-1.286	0.00075	9.47E-03	-	
TCONS_00109042	4	128289254	3007	318	-2.204	0.00065	8.43E-03	-	
TCONS_00109043	4	128292323	4205	282	-1.607	0.00045	6.24E-03	-	
TCONS_00109044	4	128296579	8144	333	-1.377	0.00005	9.26E-04	-	
TCONS_00109046	4	128309564	5631	414	-1.435	0.00005	9.26E-04	-	
TCONS_00109048	4	128315750	8091	351	-1.133	0.0003	4.46E-03	-	

TCONS_00109582	4	151959192	834	189		0.00005	9.26E-04	-	
TCONS_00109614	4	152042984	11907	408	-1.007	0.00005	9.26E-04	-	
TCONS_00109691	4	160663181	3404	318	-0.947	0.0005	6.80E-03	-	
TCONS_00109693	4	160668191	5654	468	-0.894	0.0002	3.15E-03	-	
TCONS_00109739	4	162573744	1285	240	-1.576	0.00005	9.26E-04	-	
TCONS_00109861	4	167676992	2722	228	-1.128	0.0001	1.72E-03	-	
TCONS_00110396	5	8215405	19572	432	-1.334	0.00005	9.26E-04	-	
TCONS_00110510	5	21769344	22967	480	-0.941	0.00035	5.07E-03	-	
TCONS_00110511	5	21769344	27403	603	-1.129	0.0006	7.89E-03	-	
TCONS_00111588	5	113458805	812	99		0.00005	9.26E-04	-	
TCONS_00111662	5	117862703	7319	240	-1.027	0.0005	6.80E-03	-	
TCONS_00112434	5	142118250	6188	474	-0.947	0.00025	3.82E-03	-	
TCONS_00112435	5	142140448	15871	294	-0.697	0.00075	9.47E-03	-	
TCONS_00113300	5	160939712	4868	282	-1.212	0.00045	6.24E-03	-	
TCONS_00113306	5	160954079	9771	570	-0.965	0.0003	4.46E-03	-	
TCONS_00113308	5	160969418	21754	390	-0.747	0.00025	3.82E-03	-	
TCONS_00113309	5	160991323	12596	354	-0.779	0.0009	1.10E-02	-	
TCONS_00113310	5	161004033	15448	522	-0.872	0.00005	9.26E-04	-	
TCONS_00114812	5	74584886	10260	591	-0.953	0.0005	6.80E-03	-	
TCONS_00115076	5	91124669	3947	726	0.841	0.00005	9.26E-04	5_8S_rRNA	
TCONS_00116878	5	166934541	17141	519	-0.87	0.00005	9.26E-04	-	
TCONS_00118195	5	63127856	608	75		0.00005	9.26E-04	-	
TCONS_00119583	5	142124505	8266	303	-0.915	0.00005	9.26E-04	-	
TCONS_00119584	5	142158770	31395	390	-0.905	0.00005	9.26E-04	-	
TCONS_00119587	5	142203501	8871	291	-1.062	0.00015	2.46E-03	-	
TCONS_00120451	6	6570377	11593	540	-0.649	0.00035	5.07E-03	-	
TCONS_00120903	6	30627930	1598	189	0.733	0.00005	9.26E-04	5_8S_rRNA	
TCONS_00121897	6	103364181	39015	348	-1.249	0.00005	9.26E-04	AC118496.1	
TCONS_00123074	6	6584464	12059	255	-0.702	0.0001	1.72E-03	-	
TCONS_00123279	6	25262284	3839	228	-1.251	0.0009	1.10E-02	-	
TCONS_00123625	6	43185055	3997	282	-0.925	0.0008	9.98E-03	-	
TCONS_00125707	6	9736700	21652	741	-0.898	0.00005	9.26E-04	-	
TCONS_00126696	6	61180390	6096	333	-0.932	0.0009	1.10E-02	-	
TCONS_00126700	6	61202188	5375	207	-0.942	0.0007	8.95E-03	-	
TCONS_00127431	6	95163447	13970	573	-0.73	0.0006	7.89E-03	-	
TCONS_00127816	6	105160707	8232	342	-0.753	0.00095	1.14E-02	-	
TCONS_00130846	7	123786501	3439	435	-1.332	0.00075	9.47E-03	-	
TCONS_00133701	7	114851069	18280	429	-0.981	0.00005	9.26E-04	-	
TCONS_00136140	7	52612694	561	141		0.00015	2.46E-03	-	
TCONS_00136728	7	75184464	5754	258	-0.774	0.00005	9.26E-04	-	
TCONS_00136983	7	92497283	1949	279	-0.959	0.00005	9.26E-04	-	
TCONS_00137802	7	137645621	2653	237	-1.974	0.00075	9.47E-03	-	
TCONS_00137869	7	140662782	558	90		0.00005	9.26E-04	-	
TCONS_00138740	8	44342449	7157	288	-1.151	0.00005	9.26E-04	-	
TCONS_00138742	8	44354220	10763	405	-1.359	0.00005	9.26E-04	-	
TCONS_00138761	8	45607484	16808	471	-0.799	0.0002	3.15E-03	-	
TCONS_00139274	8	63758031	37388	345	-0.69	0.0002	3.15E-03	-	
TCONS_00139337	8	64811940	32314	429	-1.16	0.0004	5.66E-03	-	
TCONS_00139650	8	75108985	2152	189	-1.306	0.00035	5.07E-03	-	
TCONS_00139757	8	77826298	1823	156	-0.912	0.0009	1.10E-02	-	
TCONS_00139923	8	85186549	36463	438	-0.739	0.0001	1.72E-03	-	
TCONS_00141112	8	162675	1272	210	1.09	0.00005	9.26E-04	-	
TCONS_00141592	8	26651222	52493	3591	-0.862	0.0001	1.72E-03	AABR07069524.1	

TCONS_00141835	8	44328120	14029	300	-0.869	0.00005	9.26E-04	-	
TCONS_00141837	8	44381195	7946	273	-0.924	0.00005	9.26E-04	-	
TCONS_00141838	8	44389523	9962	330	-0.937	0.00005	9.26E-04	-	
TCONS_00142601	8	69466800	8263	351	-1.026	0.00025	3.82E-03	-	
TCONS_00142735	8	73195276	61117	516	-0.705	0.00005	9.26E-04	-	
TCONS_00142764	8	75439357	16113	429	-0.695	0.0006	7.89E-03	-	
TCONS_00142765	8	75455714	12509	345	-0.715	0.0008	9.98E-03	-	
TCONS_00142766	8	75469543	29463	453	-0.809	0.00005	9.26E-04	-	
TCONS_00142869	8	80742773	59339	423	-0.638	0.00035	5.07E-03	-	
TCONS_00143785	8	127603421	11038	285	-0.793	0.00005	9.26E-04	-	
TCONS_00144469	8	30650546	1085	84		0.00005	9.26E-04	-	
TCONS_00144892	8	44350342	3588	267	-1.275	0.00005	9.26E-04	-	
TCONS_00145149	8	52794526	4958	231	-1.043	0.0009	1.10E-02	-	
TCONS_00145643	8	75117588	4254	462	-1.219	0.00005	9.26E-04	-	
TCONS_00145675	8	77805548	12687	315	-0.878	0.0002	3.15E-03	-	
TCONS_00145677	8	77820507	4275	348	-0.898	0.0009	1.10E-02	-	
TCONS_00145836	8	85256907	2317	204	-1.07	0.00005	9.26E-04	-	
TCONS_00146188	8	105742920	5151	309	-1.107	0.0005	6.80E-03	-	
TCONS_00146193	8	105760458	6804	291	-0.95	0.0003	4.46E-03	-	
TCONS_00149694	9	27844925	45506	459	-1.132	0.00005	9.26E-04	-	
TCONS_00149700	9	27925341	13514	426	-1.249	0.00005	9.26E-04	-	
TCONS_00149701	9	27943951	35453	477	-0.973	0.00005	9.26E-04	-	
TCONS_00149702	9	27943951	19746	387	-1.1	0.00005	9.26E-04	-	
TCONS_00149703	9	28005854	12540	369	-1.268	0.00005	9.26E-04	-	
TCONS_00149704	9	28018578	28068	294	-1.223	0.00005	9.26E-04	-	
TCONS_00149705	9	28046888	79518	570	-1.076	0.00005	9.26E-04	-	
TCONS_00150429	9	76031683	6590	267	-0.996	0.00065	8.43E-03	-	
TCONS_00151710	9	27821863	5744	285	-1.634	0.00005	9.26E-04	-	
TCONS_00151721	9	27916744	8490	417	-1.005	0.00005	9.26E-04	-	
TCONS_00151722	9	27939236	2498	327	-1.053	0.00005	9.26E-04	-	
TCONS_00152523	9	76015427	15849	267	-0.853	0.00035	5.07E-03	-	
TCONS_00153022	9	99513680	2596	162	-1.427	0.0001	1.72E-03	-	
TCONS_00154182	X	26635190	30840	555	-0.669	0.00015	2.46E-03	-	
TCONS_00154183	X	26675170	20874	273	-0.66	0.00015	2.46E-03	-	
TCONS_00154297	X	34313311	15948	735	-0.676	0.0003	4.46E-03	-	
TCONS_00154299	X	34367098	29916	387	-0.643	0.00035	5.07E-03	-	
TCONS_00154300	X	34399276	26918	423	-0.754	0.00005	9.26E-04	-	
TCONS_00154302	X	34426464	41510	300	-0.712	0.00005	9.26E-04	-	
TCONS_00154303	X	34468103	23150	480	-0.91	0.00005	9.26E-04	-	
TCONS_00156113	X	26696434	11083	261	-1.014	0.00005	9.26E-04	-	
TCONS_00156114	X	26707992	60343	564	-0.76	0.00005	9.26E-04	-	
TCONS_00156115	X	26773818	16368	507	-0.93	0.00005	9.26E-04	-	
TCONS_00156116	X	26791349	15715	309	-0.755	0.0002	3.15E-03	-	
TCONS_00156117	X	26827030	18213	720	-0.738	0.00005	9.26E-04	-	
TCONS_00158250	X	26768514	5087	207	-0.772	0.00095	1.14E-02	-	
TCONS_00158251	X	26807137	18856	306	-0.643	0.00065	8.43E-03	-	
TCONS_00158354	X	34517348	20425	351	-0.744	0.00025	3.82E-03	-	
TCONS_00159070	X	75048034	13903	297	-0.953	0.00005	9.26E-04	-	
TCONS_00159564	X	105611105	980	117		0.00005	9.26E-04	-	

Supplemental Table S9
Site Table lncRNA Prostate Stromal Epithelial Overlaps (p<0.001)

test_id	Chr	Start	Gene	Category
TCONS_00000097	1	8126655	-	
TCONS_00000104	1	8255913	-	
TCONS_00005960	1	249591548	-	
TCONS_00005961	1	249596765	-	
TCONS_00005962	1	249645239	-	
TCONS_00005963	1	249695987	-	
TCONS_00005965	1	249735066	-	
TCONS_00006920	1	14132251	-	
TCONS_00006935	1	14180975	-	
TCONS_00007020	1	20966066	-	
TCONS_00007021	1	20989996	-	
TCONS_00012578	1	249789640	-	
TCONS_00012833	1	261854669	-	
TCONS_00017668	1	249628639	-	
TCONS_00017669	1	249665572	-	
TCONS_00017670	1	249722776	-	
TCONS_00025682	10	101696383	-	
TCONS_00025683	10	101708032	-	
TCONS_00025957	10	107939842	-	
TCONS_00028247	10	107926884	-	
TCONS_00030911	11	69426747	-	
TCONS_00030912	11	69452557	-	
TCONS_00033925	12	15362330	-	
TCONS_00033927	12	15400657	-	
TCONS_00035673	12	15259395	-	
TCONS_00036032	12	28381981	Rn60_12_0305.1	
TCONS_00037143	12	15426684	-	
TCONS_00038987	13	96534691	-	
TCONS_00039674	13	50599107	-	
TCONS_00042412	13	96618810	-	
TCONS_00044564	14	101135664	-	
TCONS_00048755	15	2294539	-	
TCONS_00048980	15	15985366	-	
TCONS_00048981	15	16035004	-	
TCONS_00048984	15	16159303	-	
TCONS_00048987	15	16226726	-	
TCONS_00048991	15	16337989	-	
TCONS_00050618	15	16055517	-	
TCONS_00050621	15	16233519	-	
TCONS_00052109	15	2218730	-	

TCONS_00052114	15	2259144	-	
TCONS_00052397	15	16283172	-	
TCONS_00062030	17	81633170	-	
TCONS_00066402	18	56957425	Mir143	
TCONS_00069597	19	47159236	-	
TCONS_00069603	19	47313675	-	
TCONS_00069606	19	47411063	-	
TCONS_00069607	19	47421312	-	
TCONS_00070929	19	47143859	-	
TCONS_00071911	19	47373166	-	
TCONS_00076924	2	39135042	-	
TCONS_00086062	20	18688024	-	
TCONS_00086065	20	18738902	-	
TCONS_00086066	20	18753079	-	
TCONS_00093005	3	166467656	-	
TCONS_00096278	3	139333856	-	
TCONS_00097078	3	165360737	-	
TCONS_00097079	3	165365024	-	
TCONS_00097213	3	175763331	Mir1b	
TCONS_00100514	3	175775624	-	
TCONS_00103433	4	168527521	-	
TCONS_00103434	4	168539534	-	
TCONS_00103517	4	171595598	-	
TCONS_00105564	4	125684691	-	
TCONS_00106491	4	171614193	-	
TCONS_00106492	4	171634259	-	
TCONS_00116878	5	166934541	-	
TCONS_00121897	6	103364181	AC118496.1	
TCONS_00123074	6	6584464	-	
TCONS_00133701	7	114851069	-	
TCONS_00136983	7	92497283	-	
TCONS_00138742	8	44354220	-	
TCONS_00139337	8	64811940	-	
TCONS_00139923	8	85186549	-	
TCONS_00141837	8	44381195	-	
TCONS_00141838	8	44389523	-	
TCONS_00142735	8	73195276	-	
TCONS_00144892	8	44350342	-	
TCONS_00145836	8	85256907	-	
TCONS_00149700	9	27925341	-	
TCONS_00149701	9	27943951	-	
TCONS_00149702	9	27943951	-	
TCONS_00149703	9	28005854	-	

TCONS_00149704	9	28018578	-	
TCONS_00149705	9	28046888	-	
TCONS_00151721	9	27916744	-	
TCONS_00154183	X	26675170	-	
TCONS_00154297	X	34313311	-	
TCONS_00156113	X	26696434	-	
TCONS_00156114	X	26707992	-	

Supplemental Table S10
Site Table mRNA Prostate Epithelial (p<0.001)

test_id	Chr	Start	log2.fold_change.	p_value	q_value	Gene	Category
TCONS_00000077	1	7101714	0.778	2.00E-04	3.15E-03	Sf3b5	Translation
TCONS_00000193	1	13164649		5.00E-05	9.26E-04	-	
TCONS_00000239	1	14224392	0.661	2.00E-04	3.15E-03	Perp	Apoptosis
TCONS_00000693	1	39729055	0.917	5.00E-04	6.80E-03	Ppp1r14c	Signaling
TCONS_00001123	1	59700063	-1.006	9.00E-04	1.10E-02	LOC688452	Unknown
TCONS_00001353	1	70210799	-1.59	6.00E-04	7.89E-03	Peg3	Transcription
TCONS_00001407	1	72624704	1.442	5.00E-05	9.26E-04	Tmem238	
TCONS_00001542	1	78417718	1.103	6.50E-04	8.43E-03	Tmem160	Unknown
TCONS_00001546	1	78671120	0.708	8.50E-04	1.05E-02	Ap2s1	Transport
TCONS_00001571	1	78818398	-1.417	5.00E-05	9.26E-04	Gng8	Signaling
TCONS_00001738	1	81750927	0.651	2.00E-04	3.15E-03	Rps19	Translation
TCONS_00002540	1	101692972	0.754	7.00E-04	8.95E-03	Rpl18	Transcription
TCONS_00002547	1	101859345	0.694	2.50E-04	3.82E-03	Kdelr1	
TCONS_00002873	1	134699031	-1.671	4.50E-04	6.24E-03	Rgma	
TCONS_00002934	1	139890559	-2.088	5.50E-04	7.37E-03	Ntrk3	Receptor
TCONS_00003148	1	154371720	-0.82	3.00E-04	4.46E-03	Picalm	Transport
TCONS_00003328	1	165549343	1.132	4.00E-04	5.66E-03	Coa4	
TCONS_00003512	1	168945448	-1.687	5.00E-05	9.26E-04	AC113925.2	
TCONS_00004364	1	199412833	-1.027	2.00E-04	3.15E-03	Fus	
TCONS_00004389	1	199941160	-1.155	1.50E-04	2.46E-03	Bag3	Apoptosis
TCONS_00004790	1	214375514	0.709	2.50E-04	3.82E-03	Taldo1	Metabolism
TCONS_00005019	1	219439952	0.705	2.00E-04	3.15E-03	Ppp1ca	Signaling
TCONS_00005400	1	225163390	0.82	5.00E-05	9.26E-04	Eef1g	Translation
TCONS_00005597	1	228142777	0.898	1.50E-04	2.46E-03	Mrpl16	Transcription
TCONS_00005858	1	246954979	0.773	7.50E-04	9.47E-03	Slc1a1	Metabolism
TCONS_00005982	1	251145252	1.425	7.50E-04	9.47E-03	Papss2	Metabolism
TCONS_00006008	1	252894662	-0.899	2.00E-04	3.15E-03	Ifit2	Immune
TCONS_00006143	1	259739912	-1.342	5.00E-05	9.26E-04	Entpd1	Metabolism
TCONS_00006190	1	261158260	0.648	9.50E-04	1.14E-02	Pgam1	Metabolism
TCONS_00006293	1	264738844	0.98	8.00E-04	9.98E-03	Sema4g	Signaling
TCONS_00007156	1	31527645	0.915	5.00E-05	9.26E-04	Rpl26-ps2	
TCONS_00007360	1	41608165	-1.033	5.00E-05	9.26E-04	Syne1	Development
TCONS_00007820	1	65660469	0.61	6.00E-04	7.89E-03	Rps5	Translation
TCONS_00008249	1	80549042	0.773	5.00E-05	9.26E-04	Clptm1	Development
TCONS_00008810	1	92703079	-0.76	9.50E-04	1.14E-02	Tshz3	Transcription
TCONS_00008855	1	98472744	0.932	5.00E-05	9.26E-04	Etfb	
TCONS_00009561	1	141561333	0.913	5.00E-05	9.26E-04	Anpep	Metabolism
TCONS_00009675	1	143661520	-1.374	5.00E-05	9.26E-04	Hdgfrp3	
TCONS_00009724	1	148469770		5.00E-05	9.26E-04	-	
TCONS_00009919	1	164435877	0.627	1.50E-04	2.46E-03	Rps3	Translation
TCONS_00010120	1	168971273	-1.94	5.00E-05	9.26E-04	Hbb	Metabolism
TCONS_00010229	1	170652293	0.843	5.00E-05	9.26E-04	Mrpl17	Transcription
TCONS_00010546	1	188394813	0.911	3.00E-04	4.46E-03	Gde1	Metabolism
TCONS_00011118	1	207887208	-1.706	5.00E-05	9.26E-04	Ptpre	Signaling
TCONS_00011166	1	209522876	-1.221	2.00E-04	3.15E-03	Ebf3	
TCONS_00011360	1	215541541	0.636	5.00E-05	9.26E-04	Ctsd	Protease
TCONS_00011656	1	219254278	0.718	9.50E-04	1.14E-02	Ndufv1	Metabolism
TCONS_00012084	1	222466574	0.697	2.50E-04	3.82E-03	Cox8a	Electron Transport
TCONS_00012495	1	242493522	-1.961	6.00E-04	7.89E-03	Pgm5	Metabolism

TCONS_00012534	1	246380829	-2.248	5.00E-05	9.26E-04	Glis3	Transcription
TCONS_00012622	1	252154183	-2.218	5.00E-05	9.26E-04	LOC100360690	
TCONS_00012623	1	252154183	-2.155	5.00E-05	9.26E-04	LOC100360690	
TCONS_00012629	1	252429676	-1.507	5.00E-05	9.26E-04	Ankrd22	Transcription
TCONS_00012909	1	264160128	0.862	7.50E-04	9.47E-03	Scd1	Metabolism
TCONS_00013007	1	266422131	2.446	5.00E-05	9.26E-04	Cyp17a1	Metabolism
TCONS_00013033	1	267039345	-1.438	5.00E-05	9.26E-04	Sh3pxd2a	Development
TCONS_00013253	1	282238772	0.769	3.50E-04	5.07E-03	Prdx3	Metabolism
TCONS_00014701	1	62401784		5.00E-05	9.26E-04	-	
TCONS_00015104	1	92076800		1.00E-04	1.72E-03	-	
TCONS_00015595	1	126359019		3.50E-04	5.07E-03	-	
TCONS_00016339	1	169135347		5.00E-05	9.26E-04	-	
TCONS_00016498	1	178433655		9.50E-04	1.14E-02	-	
TCONS_00017144	1	221170983		1.50E-04	2.46E-03	-	
TCONS_00017291	1	225803232		1.00E-04	1.72E-03	-	
TCONS_00017936	1	264824102		5.00E-05	9.26E-04	-	
TCONS_00018040	1	276942527		5.00E-05	9.26E-04	-	
TCONS_00018377	10	5344802		5.00E-05	9.26E-04	-	
TCONS_00018677	10	14105749	0.805	5.00E-04	6.80E-03	Msrb1	Metabolism
TCONS_00018875	10	18558127	-2.42	5.00E-05	9.26E-04	Kcnmb1	Metabolism
TCONS_00018897	10	19924555	-1.691	3.00E-04	4.46E-03	SNORA17	
TCONS_00018908	10	23658713	-1.42	5.00E-05	9.26E-04	Ebf1	Transcription
TCONS_00019004	10	34149716	0.74	5.00E-05	9.26E-04	Gnb2l1	Signaling
TCONS_00019192	10	37215936	0.803	2.00E-04	3.15E-03	Sar1b	Development
TCONS_00019197	10	37422646	0.684	9.00E-04	1.10E-02	Cdkn2aipnl	Cell Cycle
TCONS_00019212	10	37724914	0.757	5.00E-05	9.26E-04	Vdac1	Transport
TCONS_00019287	10	39786637	-1.137	5.00E-05	9.26E-04	Fnip1	
TCONS_00019590	10	49000972	-3.856	4.50E-04	6.24E-03	LOC102553715	
TCONS_00019843	10	56601287	0.765	5.00E-05	9.26E-04	Gabarap	Cytoskeleton
TCONS_00019897	10	57131385	0.836	4.50E-04	6.24E-03	Psmb6	Proteolysis
TCONS_00020306	10	66099530	1.282	7.50E-04	9.47E-03	Lyrm9	
TCONS_00020839	10	82352389	0.921	5.00E-05	9.26E-04	Lrrc59	Development
TCONS_00020878	10	83476106	0.669	9.50E-04	1.14E-02	Phb	Transcription
TCONS_00020917	10	84031954	0.762	4.00E-04	5.66E-03	Hoxb13	Transcription
TCONS_00021025	10	85978690	0.612	3.00E-04	4.46E-03	Rpl19	Translation
TCONS_00021455	10	91710494	0.616	2.50E-04	3.82E-03	Rprml	
TCONS_00022022	10	109658031	0.773	7.50E-04	9.47E-03	Mrpl12	Translation
TCONS_00022716	10	15577248	-1.785	1.00E-04	1.72E-03	Hba2	
TCONS_00022718	10	15577248	-1.727	5.00E-05	9.26E-04	Hba1	
TCONS_00022822	10	19715274	0.834	9.50E-04	1.14E-02	RGD1564698	
TCONS_00022864	10	25903910	0.625	8.50E-04	1.05E-02	Ccng1	Signaling
TCONS_00022909	10	32055192	-1.993	2.00E-04	3.15E-03	Sgcd	Cytoskeleton
TCONS_00023109	10	38779131	0.831	4.50E-04	6.24E-03	Uqcrq	Proteolysis
TCONS_00023189	10	40790844	0.712	5.50E-04	7.37E-03	Atox1	Metabolism
TCONS_00023287	10	45559577	0.614	2.50E-04	3.82E-03	Arf1	Signaling
TCONS_00023554	10	51679225	-0.923	5.00E-05	9.26E-04	Myocd	Transcription
TCONS_00023770	10	56953690	-1.44	5.00E-05	9.26E-04	Alox15	Metabolism
TCONS_00024157	10	65839897	-0.588	8.00E-04	9.98E-03	Nlk	Signaling
TCONS_00024545	10	78992579	0.73	2.00E-04	3.15E-03	LOC100363469	
TCONS_00024559	10	81648215	0.66	1.50E-04	2.46E-03	Nme2	Metabolism
TCONS_00024700	10	83895946	0.938	4.50E-04	6.24E-03	Atp5g1	Metabolism
TCONS_00025229	10	90351899	0.735	5.00E-05	9.26E-04	Slc25a39	Binding Protein
TCONS_00025764	10	104521699	0.68	8.50E-04	1.05E-02	LOC100360679	Translation

TCONS_00025900	10	106817600	0.699	7.50E-04	9.47E-03	Tk1	Signaling	
TCONS_00026041	10	109736457	0.699	5.00E-05	9.26E-04	P4hb	Metabolism	
TCONS_00026157	10	110251362	0.883	8.50E-04	1.05E-02	Sectm1b	Development	
TCONS_00026341	10	6831349		7.00E-04	8.95E-03	-		
TCONS_00026626	10	27629913		8.00E-04	9.98E-03	-		
TCONS_00026937	10	37259498		9.50E-04	1.14E-02	-		
TCONS_00027245	10	53569623		4.00E-04	5.66E-03	-		
TCONS_00028056	10	101271075		2.00E-04	3.15E-03	-		
TCONS_00028206	10	105495218		7.00E-04	8.95E-03	-		
TCONS_00028520	11	27004319	1.353	2.00E-04	3.15E-03	N6amt1	Transcription	
TCONS_00028698	11	33909438	0.795	5.00E-05	9.26E-04	Cbr3	Metabolism	
TCONS_00029227	11	61028596	-0.922	5.50E-04	7.37E-03	Boc	Receptor	
TCONS_00029332	11	66316605	2.065	1.00E-04	1.72E-03	Stxbp5l	Transcription	
TCONS_00029430	11	70034138	1.047	6.50E-04	8.43E-03	Umps	Metabolism	
TCONS_00029649	11	82465916	-1.029	2.00E-04	3.15E-03	Igf2bp2	Transcription	
TCONS_00029856	11	88047831	0.606	6.00E-04	7.89E-03	Ube2l3	Metabolism	
TCONS_00029860	11	88122270	0.744	8.00E-04	9.98E-03	Sdf2l1	Transcription	
TCONS_00030788	11	64962665	0.62	9.50E-04	1.14E-02	Cox17	Binding Protein	
TCONS_00030834	11	67004036	0.757	4.50E-04	6.24E-03	Ildr1		
TCONS_00031122	11	78015459	-1.526	5.00E-05	9.26E-04	P3h2		
TCONS_00031486	11	90403332	-1.354	2.00E-04	3.15E-03	Snai2	Transcription	
TCONS_00032978	11	68854216		1.00E-04	1.72E-03	-		
TCONS_00033057	11	74247681		8.00E-04	9.98E-03	-		
TCONS_00033213	11	80177406		7.50E-04	9.47E-03	-		
TCONS_00034231	12	23544286	1.051	5.00E-05	9.26E-04	Orai2	Metabolism	
TCONS_00034684	12	40466494	1.037	5.00E-05	9.26E-04	Aldh2	Metabolism	
TCONS_00034915	12	47667555	0.754	1.00E-04	1.72E-03	Gltp	Metabolism	
TCONS_00035179	12	2452760		5.00E-05	9.26E-04	-		
TCONS_00035920	12	23839398	-1.013	3.00E-04	4.46E-03	Hspb1	Signaling	
TCONS_00035923	12	23941447	0.635	3.50E-04	5.07E-03	Mdh2	Metabolism	
TCONS_00036076	12	30532648	0.621	6.00E-04	7.89E-03	Gbas	Transport	
TCONS_00036104	12	31134800	-2.253	5.00E-05	9.26E-04	Adgrd1		
TCONS_00036135	12	36512392	0.885	8.50E-04	1.05E-02	Aacs	Metabolism	
TCONS_00036169	12	37471259	0.668	5.00E-05	9.26E-04	Tmed2	Metabolism	
TCONS_00037229	12	19545265		4.00E-04	5.66E-03	-		
TCONS_00037347	12	30679048		9.50E-04	1.14E-02	-		
TCONS_00037405	12	31503205		4.50E-04	6.24E-03	-		
TCONS_00037440	12	37912394		5.00E-05	9.26E-04	-		
TCONS_00037981	13	47564129	0.98	5.00E-05	9.26E-04	Pigr	Receptor	
TCONS_00038555	13	77940453	0.876	8.50E-04	1.05E-02	Mrps14	Translation	
TCONS_00038759	13	87966747	-0.938	4.00E-04	5.66E-03	Rgs5	Signaling	
TCONS_00039281	13	1931824	-0.925	8.00E-04	9.98E-03	Dsel	Metabolism	
TCONS_00039358	13	26605425	-1.098	5.00E-05	9.26E-04	Bcl2	Signaling	
TCONS_00039401	13	34829128	-1.195	5.00E-05	9.26E-04	Gli2	Transcription	
TCONS_00039995	13	70566320	-1.44	3.50E-04	5.07E-03	Lamc2	Cytoskeleton	
TCONS_00040274	13	81147347	-1.009	8.50E-04	1.05E-02	Prrx1	Transcription	
TCONS_00040333	13	83434957	0.694	8.50E-04	1.05E-02	Sft2d2		
TCONS_00040426	13	88311637	-1.255	2.50E-04	3.82E-03	Ddr2	Receptor	
TCONS_00040598	13	92894430	-0.686	7.50E-04	9.47E-03	Grem2		
TCONS_00041085	13	12298490	-1.611	7.50E-04	9.47E-03	-		
TCONS_00041125	13	20113463		7.50E-04	9.47E-03	-		
TCONS_00041184	13	24984331		2.50E-04	3.82E-03	-		
TCONS_00041637	13	47214749		2.50E-04	3.82E-03	-		

TCONS_00041936	13	70653858		7.00E-04	8.95E-03	-	
TCONS_00042643	13	102101365		4.00E-04	5.66E-03	-	
TCONS_00043358	14	17143002	-1	3.00E-04	4.46E-03	Cxcl10	Growth Factors & Cytokines
TCONS_00043458	14	22091776	-1.324	3.00E-04	4.46E-03	Sult1d1	Metabolism
TCONS_00043474	14	22706900	0.799	5.00E-05	9.26E-04	LOC100360977	
TCONS_00043619	14	37113209	-0.75	5.50E-04	7.37E-03	Sgcb	Cytoskeleton
TCONS_00043758	14	44413635	0.638	2.00E-04	3.15E-03	Smim14	
TCONS_00044431	14	86673774	0.614	3.00E-04	4.46E-03	Ppia	
TCONS_00044606	14	106008085	-1.25	2.00E-04	3.15E-03	Peli1	Signaling
TCONS_00044851	14	2860964	0.561	6.00E-04	7.89E-03	Rpl5	Translation
TCONS_00045119	14	14912342	-1.373	6.50E-04	8.43E-03	Mrpl1	Translation
TCONS_00045209	14	18743684	-1.305	9.50E-04	1.14E-02	Cxcl1	Growth Factors & Cytokines
TCONS_00045210	14	18743684	-1.288	5.00E-05	9.26E-04	Cxcl1	Growth Factors & Cytokines
TCONS_00045933	14	63004406	-1.1	5.00E-05	9.26E-04	Ppargc1a	Receptor
TCONS_00046118	14	80678466	-1.859	9.50E-04	1.14E-02	ENSRNOG00000054319	
TCONS_00046389	14	85947764	0.747	8.50E-04	1.05E-02	Mrps24	Translation
TCONS_00046585	14	103181280	-1.258	5.00E-05	9.26E-04	Meis1	Transcription
TCONS_00046636	14	106378941	0.759	5.00E-05	9.26E-04	Mdh1	Metabolism
TCONS_00046703	14	108485712	-1.505	2.00E-04	3.15E-03	Rel	Transcription
TCONS_00046982	14	10945101		5.00E-05	9.26E-04	-	
TCONS_00046989	14	10988205		5.00E-05	9.26E-04	-	
TCONS_00047184	14	21279479		1.50E-04	2.46E-03	-	
TCONS_00047300	14	31407992		7.00E-04	8.95E-03	-	
TCONS_00047306	14	31421935		4.00E-04	5.66E-03	-	
TCONS_00047493	14	45399028		1.00E-04	1.72E-03	-	
TCONS_00047555	14	51599869		2.00E-04	3.15E-03	-	
TCONS_00048902	15	8188608	0.832	9.50E-04	1.14E-02	Rpl15	Translation
TCONS_00049249	15	30555098	-1.334	5.00E-05	9.26E-04	AABR07017745.4	
TCONS_00049624	15	38340990	-1.058	3.00E-04	4.46E-03	Fgf9	Growth Factors & Cytokines
TCONS_00049693	15	42640145	-1.212	5.00E-05	9.26E-04	Clu	Apoptosis
TCONS_00049733	15	44799333	-0.978	2.00E-04	3.15E-03	Nefl	Cytoskeleton
TCONS_00049823	15	51065315	1.859	5.00E-05	9.26E-04	Nkx3-1	
TCONS_00049912	15	57175880	0.728	5.00E-05	9.26E-04	Lcp1	Metabolism
TCONS_00050210	15	104026600	1.798	5.00E-05	9.26E-04	Cldn10	Cell Junction
TCONS_00050293	15	108526013	0.602	5.00E-04	6.80E-03	Tm9sf2	Unknown
TCONS_00050638	15	18532360	0.717	6.00E-04	7.89E-03	AABR07072440.1	
TCONS_00050924	15	32868086	0.627	4.50E-04	6.24E-03	Dad1	Metabolism
TCONS_00050951	15	33259042	0.675	7.00E-04	8.95E-03	Psmb5	Protease
TCONS_00051893	15	95507639	-0.922	5.00E-05	9.26E-04	Slitrk6	Receptor
TCONS_00052874	15	42559007		2.00E-04	3.15E-03	-	
TCONS_00053510	15	71737620		6.50E-04	8.43E-03	-	
TCONS_00053523	15	73654074		8.00E-04	9.98E-03	-	
TCONS_00053879	15	92038576		8.00E-04	9.98E-03	-	
TCONS_00054393	16	7212487	0.909	5.00E-04	6.80E-03	Nt5dc2	Metabolism
TCONS_00054435	16	8280274	1.22	5.00E-05	9.26E-04	Msmb	
TCONS_00054716	16	20293228	0.697	5.00E-05	9.26E-04	Rpl18a	Translation
TCONS_00055236	16	61954589	-1.685	5.00E-05	9.26E-04	Rbpms	Transcription
TCONS_00055242	16	61954589	-1.366	5.00E-05	9.26E-04	Rbpms	Transcription
TCONS_00055635	16	83824429	-1.962	5.00E-05	9.26E-04	Irs2	Unknown
TCONS_00056465	16	49543141	-1.558	5.00E-05	9.26E-04	Pdlim3	Cytoskeleton
TCONS_00056633	16	62452521	-0.745	1.00E-04	1.72E-03	Purg	Signaling
TCONS_00056664	16	68954860	0.649	7.00E-04	8.95E-03	Eif4ebp1	Transcription
TCONS_00058904	17	9653560	-1.056	7.00E-04	8.95E-03	Pdlim7	Receptor

TCONS_00058989	17	11953521	-1.64	1.00E-04	1.72E-03	Ror2	Signaling
TCONS_00059391	17	32902964	0.813	5.00E-05	9.26E-04	Serpinb1a	Proteolysis
TCONS_00059444	17	35677983	0.622	4.50E-04	6.24E-03	Uqcrfs1	Metabolism
TCONS_00060183	17	81352699	-1.114	9.50E-04	1.14E-02	Mrc1	Receptor
TCONS_00060191	17	81633170	-2.194	5.00E-05	9.26E-04	Cacnb2	Transport
TCONS_00060201	17	82065936	-0.856	4.50E-04	6.24E-03	Arl5b	Translation
TCONS_00060701	17	10558713	0.805	5.00E-04	6.80E-03	Higd2a	Apoptosis
TCONS_00060921	17	22136756	-1.218	5.00E-05	9.26E-04	Edn1	Signaling
TCONS_00061033	17	29672817	-0.661	7.00E-04	8.95E-03	Cdyl	Metabolism
TCONS_00061190	17	36713017	1.723	6.00E-04	7.89E-03	Rn60_17_0381.1	
TCONS_00061332	17	43807129	0.93	5.00E-05	9.26E-04	LOC102549173	
TCONS_00061334	17	43821224	1.076	5.00E-05	9.26E-04	LOC680097	
TCONS_00061485	17	54652450	-1.399	1.00E-04	1.72E-03	Zeb1	Transcription
TCONS_00062023	17	81171353	-1.451	5.00E-05	9.26E-04	Hacd1	
TCONS_00062706	17	15656404	0.807	1.00E-04	1.72E-03	-	
TCONS_00062743	17	16954675		6.50E-04	8.43E-03	-	
TCONS_00065054	18	52917123	0.767	3.00E-04	4.46E-03	Slc12a2	Transport
TCONS_00065110	18	56042378	0.571	7.50E-04	9.47E-03	Rps14	Translation
TCONS_00065278	18	61490030	0.607	4.50E-04	6.24E-03	Sec11c	Protease
TCONS_00065335	18	64113931	0.804	1.00E-04	1.72E-03	Mc5r	Receptor
TCONS_00065435	18	71395829	-0.99	2.50E-04	3.82E-03	Smad7	Signaling
TCONS_00065476	18	74156552	0.707	1.00E-04	1.72E-03	Atp5a1	Metabolism
TCONS_00065566	18	81694807	0.703	2.00E-04	3.15E-03	Cyb5a	Metabolism
TCONS_00065704	18	4365428	0.879	5.00E-05	9.26E-04	LOC100910109	
TCONS_00065731	18	6833410	0.659	3.00E-04	4.46E-03	Chst9	Metabolism
TCONS_00065768	18	15063078	0.997	2.50E-04	3.82E-03	Mep1b	Protease
TCONS_00066131	18	31728372	-0.964	9.00E-04	1.10E-02	Nr3c1	Transcription
TCONS_00066269	18	47500329	-0.653	8.50E-04	1.05E-02	Lox	Metabolism
TCONS_00066306	18	51619006	0.834	5.00E-05	9.26E-04	Aldh7a1	Metabolism
TCONS_00066702	18	74461054	-1.113	1.00E-04	1.72E-03	Slc14a1	Metabolism
TCONS_00067153	18	14754736		5.00E-05	9.26E-04	-	
TCONS_00067797	18	40803184		9.50E-04	1.14E-02	-	
TCONS_00067819	18	41275399		2.00E-04	3.15E-03	-	
TCONS_00068175	18	57420276		5.00E-05	9.26E-04	-	
TCONS_00068660	19	9587652	0.757	5.00E-05	9.26E-04	Got2	Metabolism
TCONS_00069071	19	26084902	0.73	1.00E-04	1.72E-03	Prdx2	Electron Transport
TCONS_00069185	19	32112950	-0.957	1.50E-04	2.46E-03	Smad1	Signaling
TCONS_00069245	19	34109736	-1.44	5.00E-05	9.26E-04	Arhgap10	Signaling
TCONS_00069443	19	39357724	1.204	9.00E-04	1.10E-02	Cyb5b	Metabolism
TCONS_00069659	19	52258946	1.052	4.50E-04	6.24E-03	Adad2	Transcription
TCONS_00069938	19	61332350	-1.072	7.00E-04	8.95E-03	Nrp1	Receptor
TCONS_00070100	19	11300083	-0.856	7.50E-04	9.47E-03	Mt1	
TCONS_00070103	19	11307966	-1.177	5.00E-05	9.26E-04	Mt2A	Receptor
TCONS_00071448	19	22010372		5.00E-05	9.26E-04	-	
TCONS_00071987	19	47629583		2.50E-04	3.82E-03	-	
TCONS_00072409	2	2605995	0.925	5.00E-05	9.26E-04	Glrx	Metabolism
TCONS_00072610	2	20416754	-0.87	6.50E-04	8.43E-03	Ssbp2	Transcription
TCONS_00072976	2	45668968	-0.726	5.50E-04	7.37E-03	Arl15	Signaling
TCONS_00073373	2	84275883	0.744	5.00E-05	9.26E-04	Dap	Signaling
TCONS_00073383	2	85377317	-0.766	5.00E-05	9.26E-04	Sema5a	Signaling
TCONS_00073602	2	115928376		5.00E-05	9.26E-04	-	
TCONS_00073976	2	142262235	-1.442	5.00E-05	9.26E-04	Lhfp	Transcription
TCONS_00074414	2	180857148		5.00E-05	9.26E-04	-	

TCONS_00074652	2	187951315	0.69	5.00E-05	9.26E-04	Ssr2	Receptor
TCONS_00075171	2	198380886	0.785	2.00E-04	3.15E-03	AABR07012582.2	
TCONS_00075181	2	198528001		7.00E-04	8.95E-03	U1	
TCONS_00075242	2	199775695	-0.941	5.00E-05	9.26E-04	Fmo5	Metabolism
TCONS_00075350	2	205159922	-1.683	5.00E-05	9.26E-04	Tspan2	Signaling
TCONS_00075420	2	206314212	1.478	5.00E-05	9.26E-04	Bcl2l15	
TCONS_00075501	2	209097926	1.378	1.00E-04	1.72E-03	Dennd2d	
TCONS_00075645	2	224851382	0.925	3.50E-04	5.07E-03	Alg14	Metabolism
TCONS_00075677	2	226899438	-1.211	6.00E-04	7.89E-03	Pde5a	Metabolism
TCONS_00075680	2	227255106	-2.144	5.00E-05	9.26E-04	Synpo2	Development
TCONS_00075973	2	251533646	-1.286	9.50E-04	1.14E-02	AABR07013701.1	
TCONS_00075978	2	251634430	1.051	5.00E-05	9.26E-04	Ddah1	Metabolism
TCONS_00076056	2	256609691	-1.165	6.00E-04	7.89E-03	Adgrl4	
TCONS_00076655	2	30175017	0.771	5.00E-05	9.26E-04	Mccc2	Metabolism
TCONS_00077359	2	88097719	1.806	5.00E-05	9.26E-04	Car2	Metabolism
TCONS_00077401	2	93574558	1.112	5.00E-05	9.26E-04	Chmp4c	Epigenetic
TCONS_00077509	2	105016625	-0.763	3.00E-04	4.46E-03	Cpa3	Proteolysis
TCONS_00077562	2	115862455	-1.925	7.50E-04	9.47E-03	Skil	Development
TCONS_00077897	2	148718279	0.735	1.50E-04	2.46E-03	Serp1	Transcription
TCONS_00078524	2	186330297	0.636	5.50E-04	7.37E-03	Cd1d1	Receptor
TCONS_00078601	2	187903300	0.93	5.00E-05	9.26E-04	Rab25	Signaling
TCONS_00079403	2	212696609	-1.699	5.00E-05	9.26E-04	-	
TCONS_00079573	2	227255106	-2.147	2.50E-04	3.82E-03	Synpo2	Development
TCONS_00079681	2	235850333	0.881	5.00E-04	6.80E-03	Rpl34	Translation
TCONS_00079763	2	240773455	-1.926	1.50E-04	2.46E-03	Nfkb1	Transcription
TCONS_00080468	2	7778858		5.00E-05	9.26E-04	-	
TCONS_00080531	2	11876743		2.00E-04	3.15E-03	-	
TCONS_00081296	2	53031029		5.00E-05	9.26E-04	-	
TCONS_00081341	2	56424619		7.50E-04	9.47E-03	-	
TCONS_00081525	2	65085570		9.00E-04	1.10E-02	-	
TCONS_00082380	2	112859219		9.50E-04	1.14E-02	-	
TCONS_00082792	2	128317812		5.00E-05	9.26E-04	-	
TCONS_00082810	2	128458061		4.00E-04	5.66E-03	-	
TCONS_00082906	2	139569408		8.50E-04	1.05E-02	-	
TCONS_00082916	2	139617773		9.00E-04	1.10E-02	-	
TCONS_00084676	2	243349916		6.50E-04	8.43E-03	-	
TCONS_00085430	20	4593317	0.754	5.00E-05	9.26E-04	Slc44a4	Metabolism
TCONS_00085914	20	11393876	0.769	5.50E-04	7.37E-03	Pfkl	Signaling
TCONS_00085999	20	13729562	0.876	5.00E-05	9.26E-04	Mif	Growth Factors & Cytokines
TCONS_00086278	20	28920615	-1.745	5.00E-05	9.26E-04	P4ha1	Metabolism
TCONS_00086321	20	30682658	1.692	3.00E-04	4.46E-03	Pcbd1	Metabolism
TCONS_00086653	20	100942		5.00E-05	9.26E-04	-	
TCONS_00087297	20	7215674	0.908	5.00E-05	9.26E-04	Rps10	Translation
TCONS_00087573	20	13665030	1.055	5.00E-05	9.26E-04	Chchd10	
TCONS_00087662	20	19358734	-0.851	8.50E-04	1.05E-02	-	
TCONS_00087908	20	31456304	-1.358	5.00E-05	9.26E-04	Col13a1	Extracellular Matrix
TCONS_00088030	20	34571647	-0.908	9.00E-04	1.10E-02	Cep85l	Epigenetic
TCONS_00089020	20	34702546		6.00E-04	7.89E-03	-	
TCONS_00089167	20	46872158		7.00E-04	8.95E-03	-	
TCONS_00089549	3	2781168	0.765	5.00E-05	9.26E-04	Edf1	Transcription
TCONS_00089872	3	9044956		6.00E-04	7.89E-03	-	
TCONS_00090393	3	45211078	-1.029	9.00E-04	1.10E-02	-	
TCONS_00090407	3	45538796	1.179	5.50E-04	7.37E-03	Dapl1	Signaling

TCONS_00090788	3	67538288	0.824	5.00E-05	9.26E-04	Dnajc10	Transcription
TCONS_00091282	3	94010474	-0.938	4.50E-04	6.24E-03	Cd59	Signaling
TCONS_00092417	3	148259595	0.997	1.50E-04	2.46E-03	AABR07054332.1	
TCONS_00092653	3	153333218	0.587	5.00E-04	6.80E-03	Rpn2	Golgi
TCONS_00092858	3	161018510	1.653	5.00E-05	9.26E-04	Wfdc2	Signaling
TCONS_00092993	3	164424514	0.765	5.50E-04	7.37E-03	Cebpb	Transcription
TCONS_00093385	3	2438088	0.808	7.00E-04	8.95E-03	Tubb4b	Cytoskeleton
TCONS_00093584	3	4341248	0.91	5.00E-05	9.26E-04	LOC684988	Translation
TCONS_00093774	3	9858629	-1.181	5.00E-04	6.80E-03	Fnbp1	Signaling
TCONS_00095212	3	82924686	0.829	1.50E-04	2.46E-03	Hsd17b12	Metabolism
TCONS_00095292	3	93379873	0.755	4.50E-04	6.24E-03	Cat	Metabolism
TCONS_00095364	3	94990067	0.643	1.00E-04	1.72E-03	Eif3m	Translation
TCONS_00095973	3	121147546	1.896	3.50E-04	5.07E-03	Anapc1	Cell Cycle
TCONS_00096581	3	150587832	0.786	2.00E-04	3.15E-03	Ahcy	
TCONS_00096711	3	152381664	0.884	5.00E-05	9.26E-04	Scand1	Transcription
TCONS_00097431	3	5154295		5.00E-05	9.26E-04	-	
TCONS_00097743	3	30019196		9.50E-04	1.14E-02	-	
TCONS_00097784	3	35539509		8.50E-04	1.05E-02	-	
TCONS_00097857	3	37920298		7.50E-04	9.47E-03	-	
TCONS_00098613	3	93172252		5.00E-05	9.26E-04	-	
TCONS_00098977	3	117510450	0.896	6.00E-04	7.89E-03	-	
TCONS_00099581	3	136572223		9.50E-04	1.14E-02	-	
TCONS_00099860	3	149039192		9.50E-04	1.14E-02	-	
TCONS_00099964	3	156275632		1.00E-04	1.72E-03	-	
TCONS_00100103	3	160926496		5.00E-05	9.26E-04	-	
TCONS_00100206	3	165669981		5.00E-05	9.26E-04	-	
TCONS_00100515	3	175788540		5.00E-05	9.26E-04	-	
TCONS_00100528	3	176668514		6.00E-04	7.89E-03	-	
TCONS_00100703	4	6022893	0.78	5.00E-04	6.80E-03	LOC100363537	
TCONS_00101075	4	27175242	0.951	3.00E-04	4.46E-03	Cyp51	Electron Transport
TCONS_00101242	4	44321882	-0.933	5.50E-04	7.37E-03	Tes	Cytoskeleton
TCONS_00101454	4	62103561	-1.523	5.00E-05	9.26E-04	Cald1	Metabolism
TCONS_00101856	4	84478838	1.131	5.00E-05	9.26E-04	Prr15	
TCONS_00101993	4	96659083	-1.96	3.50E-04	5.07E-03	Prdm5	Transcription
TCONS_00102436	4	118852061	0.694	4.50E-04	6.24E-03	Gfpt1	Metabolism
TCONS_00102456	4	119997267	0.586	8.00E-04	9.98E-03	Rpn1	Proteolysis
TCONS_00102744	4	144192988	-1.042	6.50E-04	8.43E-03	Lmcd1	Development
TCONS_00103175	4	157452606	0.82	5.00E-05	9.26E-04	Mlf2	Signaling
TCONS_00103218	4	157563989	0.992	4.50E-04	6.24E-03	Acrbp	
TCONS_00103532	4	172119330	0.88	5.00E-05	9.26E-04	Mgst1	Metabolism
TCONS_00103617	4	175431903	-1.182	1.50E-04	2.46E-03	Pde3a	Signaling
TCONS_00103974	4	10295121	-1.128	5.00E-05	9.26E-04	Fgl2	Cytoskeleton
TCONS_00103997	4	14318273	-0.796	5.00E-05	9.26E-04	Sema3c	Development
TCONS_00104183	4	30311737	1.049	5.00E-05	9.26E-04	Pon2	Metabolism
TCONS_00104702	4	77470635	0.593	6.00E-04	7.89E-03	Pdia4	Metabolism
TCONS_00105787	4	146922084	1.089	9.00E-04	1.10E-02	Tamm41	
TCONS_00105878	4	150202057	0.945	1.00E-04	1.72E-03	Ret	Signaling
TCONS_00107279	4	24082582		9.00E-04	1.10E-02	-	
TCONS_00107320	4	24179198		1.00E-04	1.72E-03	-	
TCONS_00107492	4	35001455		5.00E-05	9.26E-04	-	
TCONS_00107503	4	35021942		5.00E-05	9.26E-04	-	
TCONS_00107520	4	35069188		5.00E-05	9.26E-04	-	
TCONS_00107531	4	35100755		5.00E-04	6.80E-03	-	

TCONS_00107541	4	35115969		5.00E-05	9.26E-04	-	
TCONS_00107553	4	35147121		5.00E-05	9.26E-04	-	
TCONS_00107654	4	44424092		2.50E-04	3.82E-03	-	
TCONS_00108304	4	87025051		5.00E-05	9.26E-04	-	
TCONS_00109683	4	159399732		6.00E-04	7.89E-03	-	
TCONS_00109920	4	170430048		6.50E-04	8.43E-03	-	
TCONS_00111073	5	62109327	0.864	5.00E-05	9.26E-04	Nans	Metabolism
TCONS_00111171	5	69833224	0.935	5.00E-05	9.26E-04	Nipsnap3b	Transport
TCONS_00111644	5	116394670	-0.832	5.00E-05	9.26E-04	Nfia	Transcription
TCONS_00111711	5	119596076	-1.597	3.50E-04	5.07E-03	-	
TCONS_00112157	5	135536412	0.663	1.50E-04	2.46E-03	Prdx1	Electron Transport
TCONS_00112571	5	145188322	0.906	5.00E-04	6.80E-03	Zmym6nb	
TCONS_00112647	5	147288722	0.985	5.00E-04	6.80E-03	Tmem54	
TCONS_00112974	5	154269117	0.679	1.00E-04	1.72E-03	Fuca1	Metabolism
TCONS_00113137	5	157759415	1.139	5.00E-05	9.26E-04	Akr7a2	Metabolism
TCONS_00113190	5	159428514	1.374	5.00E-05	9.26E-04	Padi2	Translation
TCONS_00113871	5	5999474	-0.779	4.50E-04	6.24E-03	Sulf1	Metabolism
TCONS_00114248	5	39560452	-1.429	5.00E-05	9.26E-04	Fhl5	Transcription
TCONS_00114480	5	58197679	-0.871	1.00E-04	1.72E-03	Ccl21	Growth Factors & Cytokines
TCONS_00114497	5	58472549	0.916	5.00E-04	6.80E-03	Stoml2	Cytoskeleton
TCONS_00114620	5	60969740	-0.64	1.00E-04	1.72E-03	Shb	
TCONS_00114816	5	74997842	0.648	7.00E-04	8.95E-03	LOC685849	
TCONS_00114995	5	78380815	0.768	8.50E-04	1.05E-02	Pole3	Transcription
TCONS_00115189	5	102407422	-1.438	2.50E-04	3.82E-03	Bnc2	Development
TCONS_00115196	5	102407422	-2.089	5.00E-05	9.26E-04	Bnc2	Development
TCONS_00115459	5	127505613	0.741	1.00E-04	1.72E-03	Cpt2	Metabolism
TCONS_00115657	5	136020940	0.604	4.50E-04	6.24E-03	Rps8	Transcription
TCONS_00116294	5	152195360	0.688	5.00E-05	9.26E-04	Hmgn2	Transcription
TCONS_00116785	5	164886812	0.732	1.00E-04	1.72E-03	Agtrap	Receptor
TCONS_00117899	5	44419469		5.00E-05	9.26E-04	-	
TCONS_00118564	5	86111304		1.50E-04	2.46E-03	-	
TCONS_00118574	5	86153100		5.00E-05	9.26E-04	-	
TCONS_00118576	5	86165730		6.50E-04	8.43E-03	-	
TCONS_00118585	5	86192772		9.50E-04	1.14E-02	-	
TCONS_00118593	5	86229557		1.00E-04	1.72E-03	-	
TCONS_00120363	6	788547	-1.977	5.00E-05	9.26E-04	Crim1	Development
TCONS_00120364	6	788547	-1.465	8.00E-04	9.98E-03	Crim1	Development
TCONS_00120494	6	7793734	-1.879	8.50E-04	1.05E-02	Plekhh2	Signaling
TCONS_00121498	6	80159363	-0.794	5.00E-05	9.26E-04	Pnn	Translation
TCONS_00121815	6	99282849	0.665	6.50E-04	8.43E-03	Mthfd1	Metabolism
TCONS_00122392	6	128738387	0.823	1.00E-04	1.72E-03	AABR07065438.1	
TCONS_00123280	6	25267969	-1.881	2.50E-04	3.82E-03	Srd5a2	Metabolism
TCONS_00123449	6	29178857	-1.777	5.00E-05	9.26E-04	Klhl29	Transcription
TCONS_00123532	6	31049747	0.987	5.00E-05	9.26E-04	-	
TCONS_00124247	6	92597705	1.066	5.00E-05	9.26E-04	Pygl	Metabolism
TCONS_00124346	6	99845953	0.955	5.00E-05	9.26E-04	Rab15	Signaling
TCONS_00124532	6	108467409	1.063	5.00E-05	9.26E-04	Npc2	
TCONS_00124558	6	109169740	0.728	1.00E-04	1.72E-03	Tmed10	Metabolism
TCONS_00126300	6	32161840		5.00E-05	9.26E-04	-	
TCONS_00127125	6	80440633		2.50E-04	3.82E-03	-	
TCONS_00128265	6	137253247		7.00E-04	8.95E-03	-	
TCONS_00128575	7	2504694	0.717	5.00E-05	9.26E-04	Atp5b	Transport
TCONS_00128825	7	11321056	0.811	5.00E-05	9.26E-04	Mrpl54	Translation

TCONS_00128838	7	11545023	0.78	5.50E-04	7.37E-03	Slc39a3	Transport
TCONS_00129316	7	29282304	0.634	4.00E-04	5.66E-03	Arl1	Signaling
TCONS_00129441	7	38742050	-0.805	5.00E-05	9.26E-04	Dcn	Cytoskeleton
TCONS_00129443	7	38819770	-0.86	5.00E-05	9.26E-04	Lum	Receptor
TCONS_00129498	7	42269783	-0.946	5.00E-04	6.80E-03	Kitlg	Growth Factors & Cytokines
TCONS_00129632	7	58446923	-1.843	5.00E-05	9.26E-04	A930009A15Rik	
TCONS_00129638	7	58814529	0.62	4.50E-04	6.24E-03	Tspan8	Cytoskeleton
TCONS_00129659	7	60654349	-0.911	2.00E-04	3.15E-03	Cpm	Proteolysis
TCONS_00129694	7	63094910	-0.762	5.00E-05	9.26E-04	Wif1	Signaling
TCONS_00129720	7	65616176	1.32	5.00E-05	9.26E-04	Gns	Metabolism
TCONS_00129908	7	72924798	0.72	3.00E-04	4.46E-03	Laptm4b	Proteolysis
TCONS_00130101	7	94130851	0.749	2.50E-04	3.82E-03	Mal2	Immune
TCONS_00130103	7	94375019	0.942	1.50E-04	2.46E-03	Nov	Development
TCONS_00130447	7	117304741	0.705	8.00E-04	9.98E-03	Grina	Receptor
TCONS_00130530	7	118507223	-2.923	5.00E-05	9.26E-04	Rpl8	Transcription
TCONS_00130657	7	121107530	0.907	7.50E-04	9.47E-03	Apobec3b	Metabolism
TCONS_00130667	7	121311014	-1.856	5.00E-05	9.26E-04	Syngr1	Development
TCONS_00131068	7	130472550	-1.385	6.50E-04	8.43E-03	Shank3	Signaling
TCONS_00131249	7	141249043	1.134	5.00E-04	6.80E-03	Aqp5	Transport
TCONS_00131259	7	141378535	0.817	4.50E-04	6.24E-03	Cox14	
TCONS_00131306	7	141973552	0.814	2.00E-04	3.15E-03	Mettl7a	Epigenetic
TCONS_00131445	7	143881999	0.759	9.00E-04	1.10E-02	Mfsd5	Unknown
TCONS_00131710	7	2972899	0.612	1.00E-04	1.72E-03	Rpl41	
TCONS_00131932	7	11401500	0.656	2.50E-04	3.82E-03	Eef2	Translation
TCONS_00132131	7	12875536	0.745	5.00E-05	9.26E-04	Bsg	Unknown
TCONS_00132377	7	23403890	-1.26	5.00E-05	9.26E-04	Timp3	Proteolysis
TCONS_00132712	7	51404918	-1.105	3.00E-04	4.46E-03	Ppp1r12a	Signaling
TCONS_00132929	7	62850514	-1.983	5.00E-05	9.26E-04	Msrb3	Metabolism
TCONS_00132947	7	63513761	-0.702	5.50E-04	7.37E-03	Rassf3	Signaling
TCONS_00133396	7	90085213	-1.196	3.50E-04	5.07E-03	Trps1	Development
TCONS_00133409	7	92605727	-1.2	5.00E-05	9.26E-04	Ext1	Metabolism
TCONS_00133474	7	98063734	-0.738	5.00E-05	9.26E-04	Fbxo32	Transcription
TCONS_00133621	7	107734131	-1.473	5.00E-05	9.26E-04	Ndrg1	Transcription
TCONS_00133785	7	116949878	1.009	5.00E-05	9.26E-04	Pycrl	Metabolism
TCONS_00133977	7	118685021	-0.724	2.00E-04	3.15E-03	LOC100911597	Cytoskeleton
TCONS_00133998	7	119616323	0.829	1.00E-04	1.72E-03	Tst	Metabolism
TCONS_00135646	7	32540611		3.50E-04	5.07E-03	-	
TCONS_00137627	7	121648876		1.50E-04	2.46E-03	-	
TCONS_00138079	8	6013206	-1.861	5.50E-04	7.37E-03	Birc3	Apoptosis
TCONS_00138778	8	45788896	-1.379	5.00E-05	9.26E-04	AABR07070046.1	
TCONS_00138969	8	52829084	0.926	6.00E-04	7.89E-03	RGD1563941	
TCONS_00139015	8	55178288	-1.947	1.50E-04	2.46E-03	Cryab	Development
TCONS_00139224	8	62298357	0.789	2.00E-04	3.15E-03	Cox5a	Metabolism
TCONS_00139426	8	69121681	0.638	2.00E-04	3.15E-03	Rpl4	Unknown
TCONS_00139536	8	71786309	0.854	5.00E-05	9.26E-04	Fam96a	Unknown
TCONS_00139565	8	72741154	0.695	5.00E-05	9.26E-04	Rps27l	Transcription
TCONS_00139812	8	79722333	0.641	6.00E-04	7.89E-03	Rab27a	Signaling
TCONS_00140002	8	91464228	0.626	4.50E-04	6.24E-03	Bckdhb	Metabolism
TCONS_00140332	8	115069094	1.059	3.50E-04	5.07E-03	Dusp7	Signaling
TCONS_00140335	8	115131270	0.794	5.00E-05	9.26E-04	Rpl29	Translation
TCONS_00140924	8	128806128	0.608	3.00E-04	4.46E-03	Rpsa	Translation
TCONS_00141219	8	6749743	0.829	5.00E-05	9.26E-04	LOC100360491	
TCONS_00141289	8	13838571	0.83	2.50E-04	3.82E-03	RGD1309534	

TCONS_00141410	8	21911624	0.674	1.00E-04	1.72E-03	Eif3g	Translation
TCONS_00141518	8	22918698	0.748	5.00E-04	6.80E-03	Rab3d	Signaling
TCONS_00141627	8	31977000	0.815	5.00E-05	9.26E-04	Adamts15	Protease
TCONS_00142262	8	58166989	0.795	3.00E-04	4.46E-03	Acat1	Metabolism
TCONS_00142609	8	69568515	0.898	4.50E-04	6.24E-03	Tmem185a	
TCONS_00142718	8	72771472	-1.036	1.50E-04	2.46E-03	Tpm1	Cytoskeleton
TCONS_00143735	8	122954947	0.762	5.00E-05	9.26E-04	Gpd1l	Metabolism
TCONS_00143753	8	124661838	-1.421	5.00E-05	9.26E-04	Rbms3	Epigenetic
TCONS_00144202	8	21001817		9.50E-04	1.14E-02	-	
TCONS_00145869	8	88603071		2.50E-04	3.82E-03	-	
TCONS_00146441	8	116066597		9.50E-04	1.14E-02	-	
TCONS_00146461	8	118327654		5.00E-05	9.26E-04	-	
TCONS_00146880	9	10339074	0.788	3.00E-04	4.46E-03	Vmac	
TCONS_00146891	9	10446698	0.654	6.00E-04	7.89E-03	LOC301124	
TCONS_00146949	9	10773333		4.00E-04	5.66E-03	-	
TCONS_00147043	9	12740884	-1.166	1.00E-04	1.72E-03	Rftn1	Unknown
TCONS_00147589	9	43259708	0.613	9.00E-04	1.10E-02	Cox5b	Electron Transport
TCONS_00147678	9	46086901	0.862	5.00E-05	9.26E-04	Rpl31	Translation
TCONS_00147716	9	46656032	-0.879	4.00E-04	5.66E-03	Map4k4	Signaling
TCONS_00148136	9	69497120	-1.551	2.00E-04	3.15E-03	Nrp2	Receptor
TCONS_00148149	9	69953250	1.205	5.00E-04	6.80E-03	Eef1b2	Translation
TCONS_00149070	9	119349252	-1.945	5.00E-05	9.26E-04	Myom1	Unknown
TCONS_00149271	9	10441258	0.666	8.00E-04	9.98E-03	Rpl36	Translation
TCONS_00149314	9	10738221	0.717	8.00E-04	9.98E-03	Uhrf1	Transcription
TCONS_00149521	9	16845524	1.184	1.00E-04	1.72E-03	Dnph1	
TCONS_00149558	9	17211718	0.846	5.00E-05	9.26E-04	Rps2-ps6	
TCONS_00149596	9	19469705	0.707	2.50E-04	3.82E-03	Enpp5	
TCONS_00150369	9	70735847	-1.308	5.00E-05	9.26E-04	Klf7	Transcription
TCONS_00151032	9	110052592	-0.948	4.00E-04	5.66E-03	Efna5	Signaling
TCONS_00154225	X	28593404	0.562	8.00E-04	9.98E-03	Tmsb4x	Cytoskeleton
TCONS_00154257	X	32232141	0.768	3.00E-04	4.46E-03	Car5b	Metabolism
TCONS_00154417	X	48779109	-0.974	5.00E-05	9.26E-04	Tmem47	Unknown
TCONS_00154695	X	71272041	0.849	5.00E-05	9.26E-04	Gjb1	
TCONS_00155329	X	123404569	0.827	5.00E-05	9.26E-04	Slc25a5	Transport
TCONS_00155661	X	159112879	-1.833	5.50E-04	7.37E-03	Fhl1	Transcription
TCONS_00156277	X	45244037	1.003	2.00E-04	3.15E-03	Pbsn	
TCONS_00156606	X	72074107	0.538	6.50E-04	8.43E-03	LOC100362640	Translation
TCONS_00156829	X	88312674	1.135	5.00E-05	9.26E-04	M6pr	Receptor
TCONS_00157258	X	138224039	-0.695	4.50E-04	6.24E-03	Rap2c	Signaling
TCONS_00157389	X	156392629	0.963	9.00E-04	1.10E-02	Fam50a	Unknown
TCONS_00157405	X	156438250	0.541	8.00E-04	9.98E-03	RGD1563861	Transcription
TCONS_00158875	X	65918687		3.50E-04	5.07E-03	-	
TCONS_00159167	X	76092278		5.00E-04	6.80E-03	-	
TCONS_00159797	X	115408841		6.50E-04	8.43E-03	-	
TCONS_00160196	X	146983066		4.50E-04	6.24E-03	-	
TCONS_00160217	X	151255741		8.50E-04	1.05E-02	-	
TCONS_00160331	X	155865956		8.00E-04	9.98E-03	-	

Supplemental Table S11
Site Table mRNA Prostate Stromal (p<0.001)

test_id	Chr	Start	log2.fold_change.	p_value	q_value	Gene	Category
TCONS_00001571	1	78818398	-1.091	5.00E-05	9.26E-04	Gng8	Signaling
TCONS_00001738	1	81750927	0.802	5.00E-05	9.26E-04	Rps19	Translation
TCONS_00003148	1	154371720	-0.807	5.00E-05	9.26E-04	Picalm	Transport
TCONS_00003629	1	173532802	0.582	9.00E-04	1.10E-02	Eif3f	Transcription
TCONS_00004791	1	214428219	5.084	2.00E-04	3.15E-03	LOC100911575	Transcription
TCONS_00005019	1	219439952	0.704	2.00E-04	3.15E-03	Ppp1ca	Signaling
TCONS_00005504	1	225753749		5.00E-05	9.26E-04	-	
TCONS_00006143	1	259739912	-0.798	5.00E-05	9.26E-04	Entpd1	Metabolism
TCONS_00006550	1	276617120	-1.299	3.50E-04	5.07E-03	Tcf7l2	Transcription
TCONS_00007666	1	57318707	1.01	3.50E-04	5.07E-03	Dll1	Receptor
TCONS_00008079	1	75151947	1.44	4.00E-04	5.66E-03	RGD1564801	Signaling
TCONS_00008105	1	77829710	0.914	5.00E-05	9.26E-04	AABR07002624.1	
TCONS_00008259	1	80618153	0.948	4.00E-04	5.66E-03	Tomm40	Metabolism
TCONS_00008328	1	81885520	-0.957	9.50E-04	1.14E-02	Grik5	Receptor
TCONS_00008518	1	87065900	-0.943	5.00E-05	9.26E-04	Actn4	Cytoskeleton
TCONS_00008821	1	94346513	2.016	5.00E-05	9.26E-04	Uri1	
TCONS_00009139	1	103897040	-1.219	3.00E-04	4.46E-03	LOC688010	
TCONS_00009403	1	128692111	-1.735	1.50E-04	2.46E-03	Synm	Cytoskeleton
TCONS_00009561	1	141561333	0.705	4.00E-04	5.66E-03	Anpep	Metabolism
TCONS_00009697	1	146069770	0.881	7.50E-04	9.47E-03	Cemip	
TCONS_00009724	1	148469770		5.00E-05	9.26E-04	-	
TCONS_00010637	1	192233909	-1.667	5.00E-04	6.80E-03	Prkcb	Signaling
TCONS_00011838	1	220727291	0.855	9.00E-04	1.10E-02	Cst6	Signaling
TCONS_00012534	1	246380829	-0.947	5.00E-05	9.26E-04	Glis3	Transcription
TCONS_00012629	1	252429676	-0.749	1.00E-04	1.72E-03	Ankrd22	Transcription
TCONS_00012641	1	252976071	-0.884	9.00E-04	1.10E-02	Slc16a12	Metabolism
TCONS_00012725	1	259373520	-1.585	7.00E-04	8.95E-03	Sorbs1	Cytoskeleton
TCONS_00012751	1	259373520	-1.35	8.00E-04	9.98E-03	Sorbs1	Cytoskeleton
TCONS_00013102	1	275920418	-0.979	1.00E-04	1.72E-03	AABR07007000.1	
TCONS_00013111	1	276168629	-1.468	5.00E-05	9.26E-04	Gucy2g	
TCONS_00013779	1	21926037		7.50E-04	9.47E-03	-	
TCONS_00013913	1	29571894		6.00E-04	7.89E-03	-	
TCONS_00013960	1	30982079		6.50E-04	8.43E-03	-	
TCONS_00014089	1	38564539		5.00E-04	6.80E-03	-	
TCONS_00014189	1	41041331		5.00E-05	9.26E-04	-	
TCONS_00014915	1	81761684		4.50E-04	6.24E-03	-	
TCONS_00015089	1	91285268		5.50E-04	7.37E-03	-	
TCONS_00015347	1	104486176		9.00E-04	1.10E-02	-	
TCONS_00015352	1	104501749		9.00E-04	1.10E-02	-	
TCONS_00015825	1	134697025		6.00E-04	7.89E-03	-	
TCONS_00015881	1	137511778		6.00E-04	7.89E-03	-	
TCONS_00015924	1	142521201		5.00E-04	6.80E-03	-	
TCONS_00016895	1	207882902		2.50E-04	3.82E-03	-	
TCONS_00017207	1	225224397		1.00E-04	1.72E-03	-	
TCONS_00017229	1	225358161		5.00E-05	9.26E-04	-	
TCONS_00017440	1	237780513		5.00E-04	6.80E-03	-	
TCONS_00017678	1	250652788		9.50E-04	1.14E-02	-	

TCONS_00017708	1	252790440		3.50E-04	5.07E-03	-	
TCONS_00017785	1	255534407		1.50E-04	2.46E-03	-	
TCONS_00017811	1	258180320		6.00E-04	7.89E-03	-	
TCONS_00018725	10	14722582	-1.433	5.00E-05	9.26E-04	Tpsb2	
TCONS_00018875	10	18558127	-1.657	5.00E-05	9.26E-04	Kcnmb1	Metabolism
TCONS_00018885	10	18558127	-1.258	5.00E-05	9.26E-04	Kcnmb1	Metabolism
TCONS_00019004	10	34149716	0.629	2.50E-04	3.82E-03	Gnb2l1	Signaling
TCONS_00019591	10	49020453		5.00E-05	9.26E-04	LOC100911746	Development
TCONS_00020917	10	84031954	0.9	1.00E-04	1.72E-03	Hoxb13	Transcription
TCONS_00021455	10	91710494	0.726	2.00E-04	3.15E-03	Rprml	
TCONS_00024396	10	72556081	1.269	1.50E-04	2.46E-03	LOC681195	
TCONS_00024545	10	78992579	0.741	1.00E-04	1.72E-03	LOC100363469	
TCONS_00025764	10	104521699	0.736	2.00E-04	3.15E-03	LOC100360679	Translation
TCONS_00025929	10	107291934	-0.859	8.00E-04	9.98E-03	Usp36	
TCONS_00026041	10	109736457	0.585	5.00E-04	6.80E-03	P4hb	Metabolism
TCONS_00026336	10	6820815		4.00E-04	5.66E-03	-	
TCONS_00026472	10	15284952		5.50E-04	7.37E-03	-	
TCONS_00026752	10	32782120		2.00E-04	3.15E-03	-	
TCONS_00027453	10	64677863		2.00E-04	3.15E-03	-	
TCONS_00027734	10	83343820		3.00E-04	4.46E-03	-	
TCONS_00027769	10	84834950		5.00E-04	6.80E-03	-	
TCONS_00027968	10	94965953		6.50E-04	8.43E-03	-	
TCONS_00028698	11	33909438	0.868	5.00E-05	9.26E-04	Cbr3	Metabolism
TCONS_00029316	11	64959951		4.00E-04	5.66E-03	-	
TCONS_00029405	11	69481933	-1.465	5.00E-05	9.26E-04	Kalrn	Signaling
TCONS_00030268	11	32378796		1.00E-04	1.72E-03	-	
TCONS_00030788	11	64962665	0.834	3.00E-04	4.46E-03	Cox17	Binding Protein
TCONS_00031020	11	71397382	1.763	5.00E-05	9.26E-04	Tfrc	Receptor
TCONS_00031103	11	74834049	0.849	7.00E-04	8.95E-03	AABR07034532.1	
TCONS_00031740	11	13049113		5.00E-05	9.26E-04	-	
TCONS_00031802	11	13281689		5.00E-04	6.80E-03	-	
TCONS_00031992	11	20329695		1.50E-04	2.46E-03	-	
TCONS_00032046	11	25306961		8.00E-04	9.98E-03	-	
TCONS_00032670	11	57878551		6.00E-04	7.89E-03	-	
TCONS_00032672	11	57893345		9.50E-04	1.14E-02	-	
TCONS_00032821	11	60950483		5.00E-05	9.26E-04	-	
TCONS_00032826	11	60958801		9.00E-04	1.10E-02	-	
TCONS_00032838	11	60990223		4.50E-04	6.24E-03	-	
TCONS_00033108	11	75655640		8.00E-04	9.98E-03	-	
TCONS_00033381	11	89596595		2.00E-04	3.15E-03	-	
TCONS_00033400	11	90043603		9.50E-04	1.14E-02	-	
TCONS_00033671	12	5360100	-1.011	6.50E-04	8.43E-03	-	
TCONS_00034868	12	47179663	-1.357	5.00E-05	9.26E-04	Cabp1	Signaling
TCONS_00035425	12	8746854	1.041	4.00E-04	5.66E-03	Pomp	Proteolysis
TCONS_00035920	12	23839398	-1.05	1.50E-04	2.46E-03	Hspb1	Signaling
TCONS_00035971	12	24796271	0.689	6.00E-04	7.89E-03	Metazoa_SRP	
TCONS_00036104	12	31134800	-1.511	5.00E-05	9.26E-04	Adgrd1	
TCONS_00036548	12	47138345	1.304	1.00E-04	1.72E-03	Pop5	Metabolism
TCONS_00036971	12	6397165		5.00E-05	9.26E-04	-	
TCONS_00037043	12	10493442		7.50E-04	9.47E-03	-	
TCONS_00037100	12	14263223		5.00E-05	9.26E-04	-	

TCONS_00037185	12	18454144		5.00E-04	6.80E-03	-	
TCONS_00037324	12	29246573		5.00E-05	9.26E-04	-	
TCONS_00037463	12	38697437		6.00E-04	7.89E-03	-	
TCONS_00038224	13	52662982	-1.795	4.00E-04	5.66E-03	Tnnt2	Cytoskeleton
TCONS_00038788	13	89524328	0.96	7.00E-04	8.95E-03	Mpz	Extracellular Matrix
TCONS_00039254	13	111870120	0.866	3.50E-04	5.07E-03	Irf6	Transcription
TCONS_00039358	13	26605425	-0.642	1.00E-04	1.72E-03	Bcl2	Signaling
TCONS_00041002	13	2329893		9.50E-04	1.14E-02	-	
TCONS_00041223	13	26427798		5.00E-05	9.26E-04	-	
TCONS_00041326	13	35137415		5.00E-05	9.26E-04	-	
TCONS_00041408	13	41848378		6.00E-04	7.89E-03	-	
TCONS_00041664	13	48027009		9.00E-04	1.10E-02	-	
TCONS_00041876	13	66251982		1.00E-04	1.72E-03	-	
TCONS_00042372	13	92731065		5.00E-04	6.80E-03	-	
TCONS_00042545	13	97606869		5.00E-05	9.26E-04	-	
TCONS_00042940	13	113680061		2.00E-04	3.15E-03	-	
TCONS_00044322	14	84482673	-1.462	3.50E-04	5.07E-03	Lif	Growth Factors & Cytokines
TCONS_00044326	14	84482673	-1.292	7.50E-04	9.47E-03	Lif	Growth Factors & Cytokines
TCONS_00044606	14	106008085	-0.791	9.00E-04	1.10E-02	Peli1	Signaling
TCONS_00045933	14	63004406	-0.914	2.50E-04	3.82E-03	Ppargc1a	Receptor
TCONS_00046118	14	80678466	-1.179	5.50E-04	7.37E-03	ENSRNOG00000054319	
TCONS_00047080	14	16311532		6.00E-04	7.89E-03	-	
TCONS_00047229	14	24370425		9.00E-04	1.10E-02	-	
TCONS_00047325	14	32776221		8.00E-04	9.98E-03	-	
TCONS_00047401	14	36686359		3.00E-04	4.46E-03	-	
TCONS_00047768	14	61433497		5.00E-04	6.80E-03	-	
TCONS_00047835	14	61563019		2.00E-04	3.15E-03	-	
TCONS_00047844	14	61583588		1.00E-04	1.72E-03	-	
TCONS_00048112	14	76853470		5.00E-05	9.26E-04	-	
TCONS_00048182	14	83538394		5.00E-05	9.26E-04	-	
TCONS_00048187	14	83548191		5.00E-04	6.80E-03	-	
TCONS_00048402	14	103175771		5.00E-04	6.80E-03	-	
TCONS_00048487	14	107520799		1.00E-04	1.72E-03	-	
TCONS_00048493	14	107530134		5.00E-05	9.26E-04	-	
TCONS_00048562	14	109947780		7.50E-04	9.47E-03	-	
TCONS_00048577	14	110056442		1.00E-04	1.72E-03	-	
TCONS_00048621	14	112530865		5.00E-05	9.26E-04	-	
TCONS_00049693	15	42640145	-0.746	8.00E-04	9.98E-03	Clu	Apoptosis
TCONS_00049710	15	43293879	-1.124	2.00E-04	3.15E-03	Adra1a	Receptor
TCONS_00051793	15	87389609	1.07	3.00E-04	4.46E-03	AABR07019083.1	
TCONS_00052044	15	1787998		5.00E-05	9.26E-04	-	
TCONS_00052148	15	4778309		5.00E-05	9.26E-04	-	
TCONS_00052459	15	16571278		4.50E-04	6.24E-03	-	
TCONS_00052761	15	34295686		5.00E-05	9.26E-04	-	
TCONS_00052951	15	45817340		5.00E-05	9.26E-04	-	
TCONS_00053185	15	57673747		9.00E-04	1.10E-02	-	
TCONS_00053536	15	75056704		5.00E-05	9.26E-04	-	
TCONS_00053902	15	94769729		5.00E-05	9.26E-04	-	
TCONS_00054025	15	104587288		5.00E-04	6.80E-03	-	
TCONS_00054068	15	104671781		5.00E-05	9.26E-04	-	
TCONS_00054100	15	104757628		9.00E-04	1.10E-02	-	

TCONS_00054112	15	104801347		5.00E-05	9.26E-04	-	
TCONS_00054128	15	104935757		5.00E-04	6.80E-03	-	
TCONS_00054140	15	104982744		6.00E-04	7.89E-03	-	
TCONS_00054155	15	105132343		5.00E-05	9.26E-04	-	
TCONS_00054716	16	20293228	0.599	5.50E-04	7.37E-03	Rpl18a	Translation
TCONS_00055273	16	63837102	-0.971	3.00E-04	4.46E-03	Nrg1	Signaling
TCONS_00055635	16	83824429	-0.966	5.00E-05	9.26E-04	Irs2	Unknown
TCONS_00057143	16	3685776		2.00E-04	3.15E-03	-	
TCONS_00057383	16	21547985		5.00E-05	9.26E-04	-	
TCONS_00057539	16	33266384		2.00E-04	3.15E-03	-	
TCONS_00057687	16	41198094		5.00E-05	9.26E-04	-	
TCONS_00058319	16	73301943		6.50E-04	8.43E-03	-	
TCONS_00058424	16	77605481		9.50E-04	1.14E-02	-	
TCONS_00058750	17	4846788	0.74	3.00E-04	4.46E-03	Gas1	
TCONS_00058784	17	5739655		6.50E-04	8.43E-03	-	
TCONS_00058987	17	11953521	-1.263	6.50E-04	8.43E-03	Ror2	Signaling
TCONS_00058989	17	11953521	-1.001	4.50E-04	6.24E-03	Ror2	Signaling
TCONS_00060265	17	86066832	1.104	9.50E-04	1.14E-02	Msrb2	
TCONS_00060788	17	14606269	3.617	5.00E-05	9.26E-04	Ogn	Growth Factors & Cytokines
TCONS_00061492	17	55230297	-0.89	8.50E-04	1.05E-02	Svil	Cytoskeleton
TCONS_00062266	17	1512315		1.50E-04	2.46E-03	-	
TCONS_00062390	17	5474139		5.00E-05	9.26E-04	-	
TCONS_00062404	17	5831342		5.00E-04	6.80E-03	-	
TCONS_00063321	17	50651510		4.50E-04	6.24E-03	-	
TCONS_00063332	17	50676052		1.50E-04	2.46E-03	-	
TCONS_00063356	17	50716474		4.00E-04	5.66E-03	-	
TCONS_00063484	17	56647724		5.00E-05	9.26E-04	-	
TCONS_00063678	17	66082118		5.00E-05	9.26E-04	-	
TCONS_00063742	17	69723855		1.50E-04	2.46E-03	-	
TCONS_00063749	17	70071649		5.00E-04	6.80E-03	-	
TCONS_00063907	17	78007100		4.00E-04	5.66E-03	-	
TCONS_00064070	17	86413285		2.00E-04	3.15E-03	-	
TCONS_00064915	18	40542093	0.863	5.00E-05	9.26E-04	7SK	
TCONS_00064992	18	48434651	-0.998	3.50E-04	5.07E-03	Prdm6	Transcription
TCONS_00065110	18	56042378	0.7	5.00E-05	9.26E-04	Rps14	Translation
TCONS_00065278	18	61490030	0.637	5.00E-04	6.80E-03	Sec11c	Protease
TCONS_00065435	18	71395829	-0.764	4.00E-04	5.66E-03	Smad7	Signaling
TCONS_00065476	18	74156552	0.604	3.00E-04	4.46E-03	Atp5a1	Metabolism
TCONS_00065704	18	4365428	0.771	2.50E-04	3.82E-03	LOC100910109	
TCONS_00066969	18	3700072		3.50E-04	5.07E-03	-	
TCONS_00066985	18	3834245		5.00E-05	9.26E-04	-	
TCONS_00067094	18	12696703		5.00E-05	9.26E-04	-	
TCONS_00067400	18	29877964		4.00E-04	5.66E-03	-	
TCONS_00067484	18	31725695		3.00E-04	4.46E-03	-	
TCONS_00067717	18	39288777		3.00E-04	4.46E-03	-	
TCONS_00068264	18	61956613		5.00E-05	9.26E-04	-	
TCONS_00068528	18	81888511		7.00E-04	8.95E-03	-	
TCONS_00069185	19	32112950	-0.902	5.00E-05	9.26E-04	Smad1	Signaling
TCONS_00069411	19	38669229	0.702	1.00E-04	1.72E-03	Cdh3	Cytoskeleton
TCONS_00069768	19	55917735	0.633	1.50E-04	2.46E-03	Rpl13	Translation
TCONS_00070817	19	42753983	-1.03	1.00E-04	1.72E-03	Zfhx3	Transcription

TCONS_00071028	19	54693958	-1.222	5.00E-05	9.26E-04	Slc7a5	Metabolism
TCONS_00071401	19	18886915		6.50E-04	8.43E-03	-	
TCONS_00071561	19	29188465		5.00E-05	9.26E-04	-	
TCONS_00071583	19	30155543		9.50E-04	1.14E-02	-	
TCONS_00071586	19	30165798		5.00E-05	9.26E-04	-	
TCONS_00072027	19	47908556		5.00E-05	9.26E-04	-	
TCONS_00072052	19	48158021		5.00E-05	9.26E-04	-	
TCONS_00072164	19	54895418		4.00E-04	5.66E-03	-	
TCONS_00072216	19	55015138		2.00E-04	3.15E-03	-	
TCONS_00072313	19	59489821		4.00E-04	5.66E-03	-	
TCONS_00072341	19	59845171		5.00E-05	9.26E-04	-	
TCONS_00072955	2	44664096	-0.803	4.50E-04	6.24E-03	Ppap2a	Metabolism
TCONS_00075497	2	208738131	0.91	6.00E-04	7.89E-03	AABR07012795.1	
TCONS_00075593	2	216863427	0.596	7.50E-04	9.47E-03	Col11a1	Cytoskeleton
TCONS_00075680	2	227255106	-1.122	7.50E-04	9.47E-03	Synpo2	Development
TCONS_00075973	2	251533646	-0.823	3.00E-04	4.46E-03	AABR07013701.1	
TCONS_00078601	2	187903300	0.755	8.00E-04	9.98E-03	Rab25	Signaling
TCONS_00078869	2	196105568	1.435	4.00E-04	5.66E-03	Selenbp1	Receptor
TCONS_00080106	2	28028		8.50E-04	1.05E-02	-	
TCONS_00080120	2	161986		4.00E-04	5.66E-03	-	
TCONS_00080183	2	2118771		8.00E-04	9.98E-03	-	
TCONS_00080274	2	4549916		7.50E-04	9.47E-03	-	
TCONS_00080578	2	13440313		4.50E-04	6.24E-03	-	
TCONS_00080961	2	35612208		7.50E-04	9.47E-03	-	
TCONS_00081233	2	51358178		9.00E-04	1.10E-02	-	
TCONS_00081251	2	51384540		5.00E-05	9.26E-04	-	
TCONS_00081739	2	83295503		5.00E-05	9.26E-04	-	
TCONS_00081809	2	88366541		9.50E-04	1.14E-02	-	
TCONS_00082149	2	109078882		9.00E-04	1.10E-02	-	
TCONS_00082354	2	111425609		8.00E-04	9.98E-03	-	
TCONS_00082374	2	111792992		1.00E-04	1.72E-03	-	
TCONS_00082654	2	123976687		1.00E-04	1.72E-03	-	
TCONS_00083263	2	155673380		5.50E-04	7.37E-03	-	
TCONS_00083278	2	155707144		5.00E-05	9.26E-04	-	
TCONS_00083431	2	166283371		5.50E-04	7.37E-03	-	
TCONS_00083543	2	178949844		5.00E-04	6.80E-03	-	
TCONS_00083576	2	180594585		5.00E-04	6.80E-03	-	
TCONS_00083627	2	182688467		5.00E-05	9.26E-04	-	
TCONS_00084228	2	218356580		8.50E-04	1.05E-02	-	
TCONS_00084309	2	225433104		6.00E-04	7.89E-03	-	
TCONS_00084381	2	230555141		1.00E-04	1.72E-03	-	
TCONS_00084432	2	231778688		4.50E-04	6.24E-03	-	
TCONS_00084914	2	260797950		3.00E-04	4.46E-03	-	
TCONS_00086458	20	44679486	-1.726	1.00E-04	1.72E-03	Traf3ip2	Apoptosis
TCONS_00086475	20	45138362	-1.391	5.00E-05	9.26E-04	Slc16a10	Transport
TCONS_00086591	20	48330286	0.613	2.50E-04	3.82E-03	Cd24	
TCONS_00087281	20	6515587	1.404	9.00E-04	1.10E-02	Ppil1	Immune
TCONS_00087640	20	18457309	-1.159	2.00E-04	3.15E-03	Ipmk	Signaling
TCONS_00088619	20	20628272		1.50E-04	2.46E-03	-	
TCONS_00088769	20	28493471		4.00E-04	5.66E-03	-	
TCONS_00088861	20	31432612		1.50E-04	2.46E-03	-	

TCONS_00089033	20	34742410		9.00E-04	1.10E-02	-	
TCONS_00090694	3	62444295		3.00E-04	4.46E-03	-	
TCONS_00092999	3	165242117	-2.043	5.00E-05	9.26E-04	Nfatc2	Transcription
TCONS_00093584	3	4341248	0.903	5.00E-05	9.26E-04	LOC684988	Translation
TCONS_00095120	3	80844004	-0.847	5.00E-05	9.26E-04	Dgkz	Signaling
TCONS_00097407	3	3974121		9.00E-04	1.10E-02	-	
TCONS_00098077	3	59073141		3.50E-04	5.07E-03	-	
TCONS_00098113	3	60637054		5.00E-04	6.80E-03	-	
TCONS_00098578	3	91066774		5.00E-04	6.80E-03	-	
TCONS_00098594	3	91959860		4.50E-04	6.24E-03	-	
TCONS_00098935	3	113975978		5.00E-05	9.26E-04	-	
TCONS_00098983	3	118636876		6.00E-04	7.89E-03	-	
TCONS_00099147	3	124223669		9.50E-04	1.14E-02	-	
TCONS_00099287	3	134578614		5.00E-05	9.26E-04	-	
TCONS_00099325	3	134679512		5.00E-05	9.26E-04	-	
TCONS_00099338	3	134854973		6.50E-04	8.43E-03	-	
TCONS_00099521	3	135887818		7.00E-04	8.95E-03	-	
TCONS_00099612	3	136635070		9.50E-04	1.14E-02	-	
TCONS_00100055	3	158503019		4.00E-04	5.66E-03	-	
TCONS_00100260	3	166830991		5.00E-05	9.26E-04	-	
TCONS_00100744	4	6559544	-0.796	6.50E-04	8.43E-03	Rheb	Signaling
TCONS_00101242	4	44321882	-0.788	1.00E-04	1.72E-03	Tes	Cytoskeleton
TCONS_00102744	4	144192988	-1.24	5.00E-05	9.26E-04	Lmcd1	Development
TCONS_00103532	4	172119330	0.808	5.00E-05	9.26E-04	Mgst1	Metabolism
TCONS_00104054	4	22133520	-0.935	6.50E-04	8.43E-03	Abcb1a	Transport
TCONS_00105703	4	139165423	1.229	5.00E-05	9.26E-04	7SK	
TCONS_00105823	4	147686489	0.592	5.50E-04	7.37E-03	Rpl32	Translation
TCONS_00106099	4	157285178	1.521	5.00E-05	9.26E-04	Eno2	Metabolism
TCONS_00106887	4	8398453		9.00E-04	1.10E-02	-	
TCONS_00106957	4	11782586		9.50E-04	1.14E-02	-	
TCONS_00106976	4	11818378		2.00E-04	3.15E-03	-	
TCONS_00107169	4	12475478		4.50E-04	6.24E-03	-	
TCONS_00107204	4	18470228		1.00E-04	1.72E-03	-	
TCONS_00107997	4	66150235		3.00E-04	4.46E-03	-	
TCONS_00108026	4	67387719		9.00E-04	1.10E-02	-	
TCONS_00108242	4	85355078		7.50E-04	9.47E-03	-	
TCONS_00108785	4	122520636		2.00E-04	3.15E-03	-	
TCONS_00108864	4	124117266		9.50E-04	1.14E-02	-	
TCONS_00108895	4	124425470		5.00E-05	9.26E-04	-	
TCONS_00109487	4	146405192		9.50E-04	1.14E-02	-	
TCONS_00109589	4	151966771		3.50E-04	5.07E-03	-	
TCONS_00110490	5	19284547	-1.483	2.50E-04	3.82E-03	Ubxn2b	Proteolysis
TCONS_00111139	5	64476316	1.228	5.00E-05	9.26E-04	Col15a1	Extracellular Matrix
TCONS_00113162	5	158041815	-1.28	5.00E-05	9.26E-04	Tas1r2	Receptor
TCONS_00113202	5	159534201	0.696	5.00E-05	9.26E-04	Mfap2	Extracellular Matrix
TCONS_00113263	5	160821952		5.00E-05	9.26E-04	-	
TCONS_00114620	5	60969740	-0.679	2.00E-04	3.15E-03	Shb	
TCONS_00115576	5	133708227	1.642	6.00E-04	7.89E-03	Foxd2	Transcription
TCONS_00115609	5	135019777	0.931	2.00E-04	3.15E-03	Tspan1	Cytoskeleton
TCONS_00116353	5	153564437	-0.808	5.00E-04	6.80E-03	Clic4	Transport
TCONS_00116393	5	153893038	-1.281	5.00E-05	9.26E-04	Grhl3	Transcription

TCONS_00117268	5	3103793		4.00E-04	5.66E-03	-	
TCONS_00117622	5	22304456		5.00E-05	9.26E-04	-	
TCONS_00117848	5	39883798		2.00E-04	3.15E-03	-	
TCONS_00118396	5	74600931		5.00E-05	9.26E-04	-	
TCONS_00118440	5	75685189		5.00E-05	9.26E-04	-	
TCONS_00119135	5	118344641		2.00E-04	3.15E-03	-	
TCONS_00119178	5	122162124		7.50E-04	9.47E-03	-	
TCONS_00119257	5	125873613		2.00E-04	3.15E-03	-	
TCONS_00119821	5	152952593		5.00E-05	9.26E-04	-	
TCONS_00119834	5	153473890		1.50E-04	2.46E-03	-	
TCONS_00120246	5	171772123		5.00E-05	9.26E-04	-	
TCONS_00120310	5	172012190		8.00E-04	9.98E-03	-	
TCONS_00120343	5	173278982		9.50E-04	1.14E-02	-	
TCONS_00120362	6	788547	-1.394	5.00E-05	9.26E-04	Crim1	Development
TCONS_00120363	6	788547	-1.576	5.00E-05	9.26E-04	Crim1	Development
TCONS_00121595	6	91456907	0.976	5.00E-05	9.26E-04	LOC102723236	
TCONS_00122477	6	132282741	-1.881	5.00E-05	9.26E-04	Eml1	
TCONS_00123449	6	29178857	-0.715	1.50E-04	2.46E-03	Klhl29	Transcription
TCONS_00124145	6	91455332	0.653	5.00E-05	9.26E-04	Rps29	Translation
TCONS_00125506	6	6171876		4.50E-04	6.24E-03	-	
TCONS_00125564	6	6679262		9.00E-04	1.10E-02	-	
TCONS_00125791	6	14971107		9.00E-04	1.10E-02	-	
TCONS_00125812	6	15052827		2.00E-04	3.15E-03	-	
TCONS_00125823	6	15113279		1.00E-04	1.72E-03	-	
TCONS_00125852	6	15323865		4.00E-04	5.66E-03	-	
TCONS_00125969	6	22480631		8.00E-04	9.98E-03	-	
TCONS_00126245	6	31229345		2.00E-04	3.15E-03	-	
TCONS_00126514	6	45141204		5.00E-05	9.26E-04	-	
TCONS_00126964	6	72216384		7.00E-04	8.95E-03	-	
TCONS_00126999	6	72289812		5.00E-05	9.26E-04	-	
TCONS_00127328	6	92964584		5.00E-05	9.26E-04	-	
TCONS_00127462	6	96457810		5.00E-05	9.26E-04	-	
TCONS_00127632	6	102646701		4.50E-04	6.24E-03	-	
TCONS_00127665	6	102709701		6.50E-04	8.43E-03	-	
TCONS_00127721	6	102884187		1.50E-04	2.46E-03	-	
TCONS_00128100	6	128836337		9.50E-04	1.14E-02	-	
TCONS_00128249	6	135890523		6.00E-04	7.89E-03	-	
TCONS_00129632	7	58446923	-1.01	5.00E-05	9.26E-04	A930009A15Rik	
TCONS_00129657	7	60282325	1.238	1.50E-04	2.46E-03	Lyc2	
TCONS_00129720	7	65616176	1.248	5.00E-05	9.26E-04	Gns	Metabolism
TCONS_00130101	7	94130851	0.944	5.00E-05	9.26E-04	Mal2	Immune
TCONS_00130637	7	120579959	-0.81	5.00E-05	9.26E-04	Maff	Transcription
TCONS_00130981	7	128500010	1.013	5.50E-04	7.37E-03	Fam19a5	
TCONS_00131132	7	137141660	-1.234	1.00E-04	1.72E-03	Ano6	Signaling
TCONS_00131304	7	141973009		6.50E-04	8.43E-03	-	
TCONS_00131534	7	318307		5.00E-05	9.26E-04	-	
TCONS_00131710	7	2972899	0.604	7.50E-04	9.47E-03	Rpl41	
TCONS_00131970	7	11777825	-1.318	5.00E-05	9.26E-04	Dot1l	Epigenetic
TCONS_00132929	7	62850514	-1.084	8.50E-04	1.05E-02	Msrb3	Metabolism
TCONS_00132947	7	63513761	-1.064	5.00E-05	9.26E-04	Rassf3	Signaling
TCONS_00133131	7	71472482	0.791	5.50E-04	7.37E-03	Metazoa_SRP	

TCONS_00133409	7	92605727	-0.79	5.00E-05	9.26E-04	Ext1	Metabolism
TCONS_00133621	7	107734131	-0.733	5.00E-04	6.80E-03	Ndrg1	Transcription
TCONS_00133864	7	117566367	-0.898	5.00E-05	9.26E-04	Dgat1	Metabolism
TCONS_00133977	7	118685021	-0.688	3.00E-04	4.46E-03	LOC100911597	Cytoskeleton
TCONS_00133990	7	119188184	0.947	5.50E-04	7.37E-03	Eif3d	Transcription
TCONS_00134996	7	144970443	-1.133	7.50E-04	9.47E-03	Itga5	Signaling
TCONS_00135013	7	20892	1.112	4.50E-04	6.24E-03	-	
TCONS_00135336	7	12837650		5.00E-05	9.26E-04	-	
TCONS_00135503	7	18731550		5.50E-04	7.37E-03	-	
TCONS_00135586	7	23052184		5.00E-04	6.80E-03	-	
TCONS_00135753	7	36462286		5.00E-05	9.26E-04	-	
TCONS_00136173	7	52656341		5.00E-05	9.26E-04	-	
TCONS_00136513	7	62701029		8.50E-04	1.05E-02	-	
TCONS_00136686	7	71475182		8.50E-04	1.05E-02	-	
TCONS_00137471	7	113250699		4.00E-04	5.66E-03	-	
TCONS_00137501	7	116644512		5.00E-04	6.80E-03	-	
TCONS_00137659	7	122523760		5.00E-05	9.26E-04	-	
TCONS_00137796	7	137627454		5.00E-05	9.26E-04	-	
TCONS_00138912	8	50287949	-0.959	5.00E-05	9.26E-04	Sik3	Receptor
TCONS_00139015	8	55178288	-1.035	5.00E-05	9.26E-04	Cryab	Development
TCONS_00139097	8	59278261	-1.588	1.00E-04	1.72E-03	Dnaja4	Protein Binding
TCONS_00139565	8	72741154	0.727	1.50E-04	2.46E-03	Rps27l	Transcription
TCONS_00140335	8	115131270	0.639	2.00E-04	3.15E-03	Rpl29	Translation
TCONS_00140924	8	128806128	0.587	5.50E-04	7.37E-03	Rpsa	Translation
TCONS_00142534	8	66862142	0.674	5.00E-05	9.26E-04	Rplp1	Transcription
TCONS_00142718	8	72771472	-0.942	5.00E-05	9.26E-04	Tpm1	Cytoskeleton
TCONS_00144219	8	21627131		4.00E-04	5.66E-03	-	
TCONS_00144434	8	30550227		5.00E-05	9.26E-04	-	
TCONS_00144938	8	45573455		9.50E-04	1.14E-02	-	
TCONS_00145000	8	45823702		5.00E-05	9.26E-04	-	
TCONS_00145167	8	55018307		8.00E-04	9.98E-03	-	
TCONS_00145294	8	63094263		5.00E-05	9.26E-04	-	
TCONS_00145495	8	70438960		7.00E-04	8.95E-03	-	
TCONS_00145847	8	86753224		8.50E-04	1.05E-02	-	
TCONS_00146147	8	105554247		5.00E-05	9.26E-04	-	
TCONS_00146374	8	113062811		5.00E-05	9.26E-04	-	
TCONS_00146379	8	113069151		2.00E-04	3.15E-03	-	
TCONS_00146576	8	126116812		1.50E-04	2.46E-03	-	
TCONS_00147678	9	46086901	1.188	5.00E-05	9.26E-04	Rpl31	Translation
TCONS_00148043	9	66057925	-1.251	5.00E-05	9.26E-04	Cdk15	Signaling
TCONS_00148344	9	82053580	1.117	9.00E-04	1.10E-02	Wnt10a	Signaling
TCONS_00148697	9	94702107	-1.573	2.00E-04	3.15E-03	Inpp5d	Signaling
TCONS_00148776	9	98072989	1.348	2.00E-04	3.15E-03	Mlph	Metabolism
TCONS_00149271	9	10441258	0.775	1.00E-04	1.72E-03	Rpl36	Translation
TCONS_00149558	9	17211718	0.927	5.00E-05	9.26E-04	Rps2-ps6	
TCONS_00151443	9	14635314		7.00E-04	8.95E-03	-	
TCONS_00151623	9	18832171		6.00E-04	7.89E-03	-	
TCONS_00151703	9	27363255		8.00E-04	9.98E-03	-	
TCONS_00151954	9	51181029		4.50E-04	6.24E-03	-	
TCONS_00152192	9	60787372		1.50E-04	2.46E-03	-	
TCONS_00152614	9	80797029		5.00E-05	9.26E-04	-	

TCONS_00152731	9	81072298		5.00E-05	9.26E-04	-	
TCONS_00152817	9	88886524		1.00E-04	1.72E-03	-	
TCONS_00152829	9	89455768		2.00E-04	3.15E-03	-	
TCONS_00152998	9	98386163		6.50E-04	8.43E-03	-	
TCONS_00153757	X	10430846	0.906	5.00E-05	9.26E-04	LOC100361811	
TCONS_00154313	X	35488135	1.346	5.00E-05	9.26E-04	Metazoa_SRP	
TCONS_00155329	X	123404569	0.671	2.00E-04	3.15E-03	Slc25a5	Transport
TCONS_00156829	X	88312674	1.02	5.00E-05	9.26E-04	M6pr	Receptor
TCONS_00157369	X	154916378	0.917	6.50E-04	8.43E-03	AABR07042397.1	
TCONS_00157739	X	6035468		5.00E-05	9.26E-04	-	
TCONS_00158293	X	31185816		2.00E-04	3.15E-03	-	
TCONS_00158381	X	35989333		4.50E-04	6.24E-03	-	
TCONS_00159017	X	73079436		5.00E-04	6.80E-03	-	
TCONS_00159600	X	106407775		5.00E-05	9.26E-04	-	
TCONS_00159953	X	135292414		7.50E-04	9.47E-03	-	
TCONS_00160185	X	145278738		9.00E-04	1.10E-02	-	
TCONS_00160351	X	156489842		6.00E-04	7.89E-03	-	

Supplemental Table S12

Site Table mRNA Prostate Stromal - Epithelial Overlaps (p<0.001)

test_id	Chr	Start	Gene	Category
TCONS_00001571	1	78818398	Gng8	Signaling
TCONS_00001738	1	81750927	Rps19	Translation
TCONS_00003148	1	154371720	Picalm	Transport
TCONS_00005019	1	219439952	Ppp1ca	Signaling
TCONS_00006143	1	259739912	Entpd1	Metabolism
TCONS_00009561	1	141561333	Anpep	Metabolism
TCONS_00009724	1	148469770	-	
TCONS_00012534	1	246380829	Glis3	Transcription
TCONS_00012629	1	252429676	Ankrd22	Transcription
TCONS_00018875	10	18558127	Kcnmb1	Metabolism
TCONS_00019004	10	34149716	Gnb2l1	Signaling
TCONS_00020917	10	84031954	Hoxb13	Transcription
TCONS_00021455	10	91710494	Rprml	
TCONS_00024545	10	78992579	LOC100363469	
TCONS_00025764	10	104521699	LOC100360679	Translation
TCONS_00026041	10	109736457	P4hb	Metabolism
TCONS_00028698	11	33909438	Cbr3	Metabolism
TCONS_00030788	11	64962665	Cox17	Binding Protein
TCONS_00035920	12	23839398	Hspb1	Signaling
TCONS_00036104	12	31134800	Adgrd1	
TCONS_00039358	13	26605425	Bcl2	Signaling
TCONS_00044606	14	106008085	Peli1	Signaling
TCONS_00045933	14	63004406	Ppargc1a	Receptor
TCONS_00046118	14	80678466	ENSRNOG00000054319	
TCONS_00049693	15	42640145	Clu	Apoptosis
TCONS_00054716	16	20293228	Rpl18a	Translation
TCONS_00055635	16	83824429	Irs2	Unknown
TCONS_00058989	17	11953521	Ror2	Signaling
TCONS_00065110	18	56042378	Rps14	Translation
TCONS_00065278	18	61490030	Sec11c	Protease
TCONS_00065435	18	71395829	Smad7	Signaling
TCONS_00065476	18	74156552	Atp5a1	Metabolism
TCONS_00065704	18	4365428	LOC100910109	
TCONS_00069185	19	32112950	Smad1	Signaling
TCONS_00075680	2	227255106	Synpo2	Development
TCONS_00075973	2	251533646	AABR07013701.1	
TCONS_00078601	2	187903300	Rab25	Signaling
TCONS_00093584	3	4341248	LOC684988	Translation
TCONS_00101242	4	44321882	Tes	Cytoskeleton
TCONS_00102744	4	144192988	Lmcd1	Development

TCONS_00103532	4	172119330	Mgst1	Metabolism
TCONS_00114620	5	60969740	Shb	
TCONS_00120363	6	788547	Crim1	Development
TCONS_00123449	6	29178857	Klhl29	Transcription
TCONS_00129632	7	58446923	A930009A15Rik	
TCONS_00129720	7	65616176	Gns	Metabolism
TCONS_00130101	7	94130851	Mal2	Immune
TCONS_00131710	7	2972899	Rpl41	
TCONS_00132929	7	62850514	Msrb3	Metabolism
TCONS_00132947	7	63513761	Rassf3	Signaling
TCONS_00133409	7	92605727	Ext1	Metabolism
TCONS_00133621	7	107734131	Ndrg1	Transcription
TCONS_00133977	7	118685021	LOC100911597	Cytoskeleton
TCONS_00139015	8	55178288	Cryab	Development
TCONS_00139565	8	72741154	Rps27l	Transcription
TCONS_00140335	8	115131270	Rpl29	Translation
TCONS_00140924	8	128806128	Rpsa	Translation
TCONS_00142718	8	72771472	Tpm1	Cytoskeleton
TCONS_00147678	9	46086901	Rpl31	Translation
TCONS_00149271	9	10441258	Rpl36	Translation
TCONS_00149558	9	17211718	Rps2-ps6	
TCONS_00155329	X	123404569	Slc25a5	Transport
TCONS_00156829	X	88312674	M6pr	Receptor

Supplemental Table S13
Genes previously associated with prostate disease

Gene	Description
Ager	advanced glycosylation end product-specific receptor
Akt1	AKT serine/threonine kinase 1
AMACR	alpha-methylacyl-CoA racemase
ANO1	anoctamin 1
APC	APC; WNT signaling pathway regulator
AR	androgen receptor
ATM	ATM serine/threonine kinase
Axin2	axin 2
Bcl2	BCL2, apoptosis regulator
Bmp2	bone morphogenetic protein 2
Bmp7	bone morphogenetic protein 7
BRCA2	BRCA2; DNA repair associated
CBX7	chromobox 7
CCND2	cyclin D2
CCR2	C-C motif chemokine receptor 2
CDC27	cell division cycle 27
CDH1	cadherin 1
CHD1	chromodomain helicase DNA binding protein 1
Ctnnb1	catenin beta 1
CXCL1	C-X-C motif chemokine ligand 1
CXCL12	C-X-C motif chemokine ligand 12
CXCL2	C-X-C motif chemokine ligand 2
CXCL5	C-X-C motif chemokine ligand 5
CXCL6	C-X-C motif chemokine ligand 6
CXCR1	C-X-C motif chemokine receptor 1
CXCR2	C-X-C motif chemokine receptor 2
DGKZ	diacylglycerol kinase zeta
DLEC1	deleted in lung and esophageal cancer 1
EEF1A1	eukaryotic translation elongation factor 1 alpha 1
EGF	epidermal growth factor
Erbb2	erb-b2 receptor tyrosine kinase 2
ERG	ERG; ETS transcription factor
ETS1	ETS proto-oncogene 1, transcription factor
ETV1	ets variant 1
ETV4	ets variant 4
ETV5	ets variant 5
Exo1	exonuclease 1
FGF1	fibroblast growth factor 1
FGF10	fibroblast growth factor 10
FGF2	fibroblast growth factor 2
Fgf3	fibroblast growth factor 3
FGF7	fibroblast growth factor 7

FGF9	fibroblast growth factor 9
FGFR2	fibroblast growth factor receptor 2
FLI1	Fli-1 proto-oncogene; ETS transcription factor
FolH1	folate hydrolase 1
FOXA1	forkhead box A1
FRG1	FSHD region gene 1
FSTL5	follistatin-like 5
GSTP1	glutathione S-transferase pi 1
HES5	hes family bHLH transcription factor 5
HIF1A	hypoxia inducible factor 1 subunit alpha
HIPK2	homeodomain interacting protein kinase 2
HMGA1	high mobility group AT-hook 1
HMGA2	high mobility group AT-hook 2
HMGB1	high mobility group box 1
HNRNPA2B1	heterogeneous nuclear ribonucleoprotein A2/B1
HOXC6	homeo box C6
HOXD3	homeo box D3
HRAS	rat sarcoma viral oncogene
IDH1	isocitrate dehydrogenase (NADP(+)) 1; cytosolic
IGF1	insulin-like growth factor 1
IGF1R	insulin-like growth factor 1 receptor
IGF2	insulin-like growth factor 2
IGFBP2	insulin-like growth factor binding protein 2
IGFBP5	insulin-like growth factor binding protein 5
IL15	interleukin 15
IL17	interleukin17
IL1A	interleukin 1 alpha
IL2	interleukin 2
Il4	interleukin 4
IL6	interleukin 6
IL8	interleukin 8
Ipcef1	interaction protein for cytohesin exchange factors 1
IQGAP2	IQ motif containing GTPase activating protein 2
Jak1	Janus kinase 1
Jak2	Janus kinase 2
KDM6A	lysine demethylase 6A
KIF5A	kinesin family member 5A
Klf4	Kruppel like factor 4
KLK3	kallikrein related peptidase 3 (PSA)
KMT2C	lysine methyltransferase 2C
KRAS	rat sarcoma viral oncogene
Lef1	lymphoid enhancer binding factor 1
Lin28a	lin-28 homolog A
Lin28c	lin-28 homolog C
MAP2K1	mitogen-activated protein kinase kinase 1

MAP2K2	mitogen-activated protein kinase kinase 2
MAPK1	mitogen activated protein kinase 1
miR-156	microRNA 156
miR-181b	microRNA 181b
miR-196a-2	microRNA 196a-2
miR-21	microRNA 21
MIRLET7A1	microRNA let-7a-1
Mirlet7c1	microRNA let-7c-1
MOB3B	MOB kinase activator 3B
MT-TC	mitochondrially encoded tRNA cysteine
MYC	MYC proto-oncogene; bHLH transcription factor
NRAS	rat sarcoma viral oncogene
Nfkb1	nuclear factor kappa B subunit 1
Nkx3-1	NK3 homeobox 1
Nme1	NME/NM23 nucleoside diphosphate kinase 1
Nme2	NME/NM23 nucleoside diphosphate kinase 2
OR10H3	olfactory receptor family 10 subfamily H member 3
OR1S2	olfactory receptor family 1 subfamily S member 2
OR2T35	olfactory receptor family 2 subfamily T member 35
PCA3	prostate cancer associated 3
PDGFA	platelet derived growth factor subunit A
PIK3CA	phosphatidylinositol-4;5-bisphosphate 3-kinase; catalytic subunit alpha
Pik3cg	phosphatidylinositol-4,5-bisphosphate 3-kinase, catalytic subunit gamma
PITX2	paired-like homeodomain 2
PTEN	phosphatase and tensin homolog
Ptn	pleiotrophin (HARP)
PYHIN1	pyrin and HIN domain family member 1
Raf1	Raf-1 proto-oncogene; serine/threonine kinase
RARB	retinoic acid receptor; beta
RASSF1	Ras association domain family member 1
RB1	RB transcriptional corepressor 1
SFRP1	secreted frizzled-related protein 1
Shh	sonic hedgehog
SLC45A3	solute carrier family 45; member 3
SNAI1	snail family transcriptional repressor 1
SNAI2	snail family transcriptional repressor 2
SOX9	SRY box 9
SPINK1	serine peptidase inhibitor; Kazal type 1
SPOP	speckle type BTB/POZ protein
Stat1	signal transducer and activator of transcription 1
Stat2	signal transducer and activator of transcription 2
Stat3	signal transducer and activator of transcription 3
Stat4	signal transducer and activator of transcription 4
Stat5a	signal transducer and activator of transcription 5a
Stat5b	signal transducer and activator of transcription 5b

Stat6	signal transducer and activator of transcription 6
TACC2	transforming; acidic coiled-coil containing protein 2
TDRD1	tudor domain containing 1
TGFA	transforming growth factor alpha
TGFB1	transforming growth factor beta 1
TGFB2	transforming growth factor beta 2
TGFB3	transforming growth factor beta 3
TGFBR2	transforming growth factor, beta receptor 2
Thbs1	Thrombospondin-1
TLR2	toll-like receptor 2
TLR3	toll-like receptor 3
TLR4	toll-like receptor 4
TMPRSS2	transmembrane serine protease 2
TP53	tumor protein p53
VEGFA	vascular endothelial growth factor A
Wnt1	Wnt family member 1
Wnt10a	Wnt family member 10A
Wnt10b	Wnt family member 10B
Wnt11	Wnt family member 11
Wnt16	Wnt family member 16
Wnt2	Wnt family member 2
Wnt2b	Wnt family member 2B
Wnt3	Wnt family member 3
Wnt3a	Wnt family member 3A
Wnt4	Wnt family member 4
Wnt5a	Wnt family member 5A
Wnt5b	Wnt family member 5B
Wnt6	Wnt family member 6
Wnt7a	Wnt family member 7A
Wnt7b	Wnt family member 7B
Wnt8a	Wnt family member 8A
Wnt8b	Wnt family member 8B
Wnt9a	Wnt family member 9A
Wnt9b	Wnt family member 9B
Wt1	Wilms tumor 1
ZFP91	zinc finger protein 91
